# A Bottom-up Computational Study of Doped Amorphous Carbon Clusters as Precursors of Carbon Nanodots

**DOI:** 10.1021/acs.jpca.5c03577

**Published:** 2025-07-21

**Authors:** Francesca D’Ambrosio, Alice Frustaci, Enrico Bodo

**Affiliations:** Chemistry Department, 9311 University of Rome “La Sapienza”, P. Aldo Moro 5, Rome 00185, Italy

## Abstract

In this work, we present a bottom-up computational study of unsaturated amorphous carbon clusters partially doped with nitrogen and other typical chemical groups. We focus on their structure and electronic properties with the aim of providing possible models of the amorphous portions of the core of carbon nanodots, a class of fluorescent carbon-based nanoparticles whose complex atomistic structure is not yet fully understood. Using the GOAT-EXPLORE algorithm in combination with semiempirical and DFT methods, we generated and optimized a broad ensemble of structural isomers for C_*n*_X clusters of up to 35 atoms of carbon, with X including nitrogen and typical functional groups found in the carbon nanodots (X = OH, NH_2_, COOH, and CONH_2_). Structural and electronic parameters were evaluated to understand how size, doping, and functionalization impact electronic delocalization, stability, and potential reactivity. Our findings highlight a structural evolution from linear to polycyclic and cage-like motifs with increasing size and demonstrate that functionalization mainly influences local electronic environments without drastically affecting the overall architecture.

## Graphical abstract

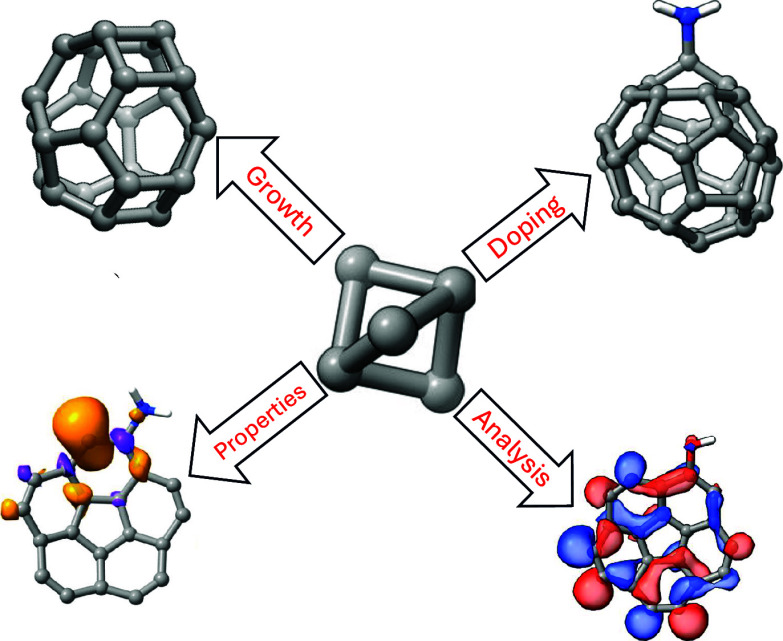

## 1 Introduction

Xu and colleagues^1,2^ discovered carbon dots (CDs) in 2004 as one of the byproducts of the purification of single-walled carbon nanotubes. Since then, carbon dots (CDs) have emerged as a distinctive class of carbon-based nanoparticles characterized by intense photoluminescence, excellent biocompatibility, and easy surface functionalization. Thanks to their versatility, CDs have found applications in various fields, including biomedicine,^3–5^ and have shown promising results when used as sensors and catalysts.^6–12^ They can be obtained from easy synthetic procedures, which either follow a bottom-up approach—starting from small organic molecules such as amino acids, sugars, or natural polymers—or a top-down approach, involving carbon-based materials such as graphite, graphene sheets, or carbon nanotubes.^13–17^ The choice of the synthetic method influences the functional groups on the surface, the size, the morphology, and the optical properties of the resulting CDs, leading to nanoparticles with a wide range of characteristics.

CDs can be classified into four main categories, from well-ordered stacked graphene layers to highly disordered phases resembling amorphous solid carbon. From least to most disordered, we have (i) graphene quantum dots (GQDs) that are composed of one or more layers of graphene, with edge functional groups and interlayer defects whose emission is influenced by quantum confinement effects (QCE); (ii) carbon quantum dots (CQDs) that are structurally ordered nanoparticles with crystalline lattices and surface functional groups and that exhibit QCE in the emission spectra; (iii) carbon nanodots (CNDs) that are spherical nanoparticles lacking short-range order consisting in an amorphous carbonaceous core with sp^2^-ordered domains and surface functionalization; and (iv) carbon polymer dots (CPDs), containing cross-linked polymer chains variously functionalized.^18^

In recent years, CDs have received significant attention, leading to an increase in experimental and computational studies about them. Experimental structural studies show that CNDs are three-dimensional objects with scales below tens of nanometers with a mixture of allotropic carbon forms that include variably extended sp^2^ domains and sp^3^ clusters.^19–21^ However, a comprehensive understanding of their atomic-level structure and its correlation with their properties remains incomplete.^22^

In 2022, an extensive review was published by Carbonaro’s group^23^ underlining the methods and models employed for *in silico* studies of CNDs. As stated there, an effective computation model should capture all the experimental observed features of CNDs, including isolated sp^2^ domains, surface functional groups, and the possibility of doping the core itself—these are all necessary for a realistic atomistic model. If one is interested in the correlation between the structure and the optical properties, then the adopted computational model must include, at a given point, an explicit treatment of the electronic degree of freedom. This requires the use of ab initio methods and, in turn, mandates to choose a model system of reduced size to keep the computation feasible and the associated cost manageable.

Most of the initial attempts at modeling CNDs focused on small aromatic molecules, polynuclear aromatic hydrocarbons (PAHs), sharing similar characteristics to those exhibited by CDs.^24^ Specifically, the model included anthracene, pyrene, and perylene embedded in an amorphous polymeric matrix. Kundelev et al.^25^ investigated the structure of amino-functionalized perylene and pyrene molecules (−NH_2_). Hola et al.^26^ used coronene and its derivatives as small model systems to simulate the effect of surface groups in the core of CDs. Other research groups used graphene as a building block to model CNDs.^27^ They considered amide-capped graphene ribbons and their corresponding bilayers. Sheardy et al.^28^ reported several models based on neat and functionalized graphitic sheets to which they added structural defects, epoxides, and amines. Otyepka et al.^29^ developed a builder for creating CDs with different sizes and various functional groups linked to the surface. The model consists of a stack of graphenic layers of gradually decreasing size with an overall spherical shape with randomly positioned functional groups at the edge of the layers.

In addition to the models mentioned so far, there have been some efforts from various groups over the years focusing on the modeling of the amorphous core of nanodots. McCulloch et al.^30^ performed *ab-initi*o simulations of amorphous carbon, generating random carbon networks of 125 atoms at various densities. Also, Margraf at al.^31^ focused on hydrogen-passivated structures of amorphous carbon using Monte techniques. Other works explored the possibility of modeling CNDs as polymer-like nanoparticles^32^ where sp^3^-hybridized amorphous carbon cores coexist with small domains of partially sp^2^-hybridized carbon atoms.^33^

Computational approaches for simulating amorphous nanoparticles vary based on the chosen system size. For smaller structures, quantum mechanical methods—particularly DFT—are preferred due to their accuracy in electronic property predictions. In contrast, larger systems need molecular dynamics (MD) or related techniques to balance computational cost. Recently, tentative works using a machine learning (ML) approach were published^34,35^ where a carbon interatomic potential was generated and was able to reproduce properties and geometries of bulk and nanosized carbon structures. Yet, ML-based approaches that can deal with the mechanism of photoluminescence or its relationship with the atomic composition are missing.

With a few exceptions, many of the models reviewed above seem to neglect the amorphous component of the CND core. To address this gap, we propose a novel approach to modeling the amorphous region of CNDs that is based on the structural features of carbon clusters. Amorphous carbon clusters are known to act as precursors for the transformation into fullerenes under high temperature^36^ where the initial structures transform into a hollow sp^2^ shell where the carbon chains gradually insert themselves. Also, fullerene at high temperature loses fragments that can be assimilated to amorphous carbon clusters.^37^ Starting from these studies, we propose that progressively larger carbon clusters could serve as an effective model for the CND-unsaturated portions of their carbonaceous core. As their size increases, they gradually adopt cage-like structures that might further assemble within the core of nanodots during the energetic phases of their synthesis.

Here, we explore the most stable isomers of the structures of singly doped neutral, unsaturated carbon clusters. These structures are essentially C_*n*_ clusters with *n* ranging from 5 to 35 where one carbon atom has been substituted with a skeletal nitrogen, or has been attached to an −NH_2_, −OH, −COOH, and −CONH_2_ group. The choice can be justified if we consider the most common reagents used in bottom-up synthetic procedures of carbon dots, which usually employ amino acids or amine-based systems.^19^

We point out that these clusters are not meant to be fully formed structures of CNDs. They lack the saturation that is needed to provide a complete model of them. However, we think that the exploration—at a fundamental level—of these unsaturated carbon clusters is still relevant for advancing our understanding of the nanoparticles due to their possible role as building blocks or aggregation centers during their synthetic stages. For instance, the transformation of acetaldehyde into carbon dots highlights how undirected aggregation of unsaturated aldehydes can lead to the formation of carbon chains and clusters, ultimately yielding carbon nanodots.^38^ Additionally, the investigation into their structure, electronic properties, and reactivity could be of use for exploiting them to provide specific functionalities to CNDs. Their unsaturation provides these structures with unique electronic characteristics, allowing them to play a role in the rather entangled set of carbon chain and structure growth reactions.^39^ Moreover, the chemically active surface area and active sites associated with unsaturated carbon structures allow for improved adsorption properties, particularly in environmental applications where CNDs can be used as adsorbents for pollutants.^40^ Also, unsaturated carbon clusters, with a propensity for forming various structural defects, can influence the charge dynamics within CNDs by acting as sites for electron trapping and recombination processes.^41–43^

We conclude by mentioning the fact that unsaturated carbon clusters are significant not only as structural units in nanoscience but also as key intermediates in complex chemical reactions. As radicals, they are essential in the synthesis of more complex carbonaceous materials, including fullerenes and polycyclic aromatic hydrocarbons^44,45^ and they can undergo various chemical transformations that yield a plethora of functionalized carbon materials, essential for applications in nanotechnology.^46^

## 2 Methods

For the choice of method, it was essential to balance an acceptable computational cost with the accuracy of an *ab initio* calculation. The GOAT algorithm implemented in the ORCA program (version 6)^47–50^ seemed the ideal solution, allowing to run an isomer and conformer search on a given initial structure using the GFN2-xTB developed by Grimme’s group.^49,50^ Subsequently, only the low-lying conformers underwent geometry optimization at the DFT level to refine the computational accuracy.

The initial model structures containing from 5 to 35 atoms were generated with the aid of a utility developed by us. This procedure randomly places a defined number of carbon atoms within a box and generates many tentative structures with different geometries—including linear, monocyclic, polycyclic, and cage-like. Each of these structures was optimized with the GFN2-xTB method using the software XTB.^51^ The same approach was applied to the doped and functionalized clusters with the additional enforcement of appropriate bond constraints to prevent the dissociation or modification of the selected functional group. These structures were subjected to a minimum structure search using the variable topology GOAT algorithm at the GFN2-xTB level. From the GOAT ensemble of isomers, we handpicked the five lowest-energy conformers for each cluster that exhibited significantly different geometries. These last set of structures was optimized employing the ωB97X-D3/def2-TZVP level of theory. The entire process is summarized in Figure 1.

**Figure 1:**
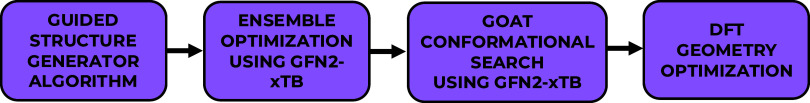
Algorithm procedure for generation of the structural ensemble.

According to previous studies on the spin multiplicity of linear carbon clusters, it is well-known that there exists an alternation between singlet and triplet states for C_*n*_ clusters based on the even–odd number of atoms.^52^ When cyclic structures begin to emerge, however, electronic delocalization favors the singlet ground state, which is consistent with the findings of Afshar et al.^53^ who explored the magnetic properties of C_*n*_ with *n* = 2–12, without finding significant magnetic behavior. Furthermore, Khavryuchenko et al. considered small models of amorphous carbon, showing that the high multiplicity states are energetically close to the singlet.^54^ On our side, a preliminary study was conducted to compare the energy difference between singlet and triplet states for a few selected small C_*n*_ clusters. In all cases, the low-lying energy states exhibited no significant differences in structure. For these reasons, here we have limited our study to singlet states for all of the C_*n*_ clusters. For the doped C_*n*_N clusters and the functionalized ones (C_*n*_–OH, C_*n*_-NH_2_, C_*n*_-COOH, and C_*n*_-CONH_2_), instead, owing to the odd number of electrons, we treated them as a doublet state. This is consistent with what has been reported in older studies.^55^ The unrestricted or spin-polarized version of the SCF procedures have been used for the open shell system.^48,56^

## 3 Discussion and Results

The structural analysis was performed on neat carbon clusters (neat C_*n*_), nitrogen-doped ones (N-doped C_*n*–1_N), and clusters functionalized with a hydroxyl (C_*n*_-OH), amino (C_*n*_-NH_2_), carboxylic (C_*n*_-COOH), or amide group (C_*n*_-CONH_2_). The chemical composition of all investigated systems is summarized in Table 1.

**Table 1:** Chemical Composition of the Carbon Clusters

C_*n*_	C_*n*–1_N	C_*n*_-OH	C_*n*_-NH_2_	C_*n*_-COOH	C_*n*_-CONH_2_
C_5_	C_4_N	C_5_OH	C_5_NH_2_	C_6_O_2_H	C_6_ONH_2_
C_6_	C_5_N	C_6_OH	C_6_NH_2_	C_7_O_2_H	C_7_ONH_2_
C_7_	C_6_N	C_7_OH	C_7_NH_2_	C_8_O_2_H	C_8_ONH_2_
C_8_	C_7_N	C_8_OH	C_8_NH_2_	C_9_O_2_H	C_9_ONH_2_
C_9_	C_8_N	C_9_OH	C_9_NH_2_	C_10_O_2_H	C_10_ONH_2_
C_10_	C_9_N	C_10_OH	C_10_NH_2_	C_11_O_2_H	C_11_ONH_2_
C_15_	C_14_N	C_15_OH	C_15_NH_2_	C_16_O_2_H	C_16_ONH_2_
C_20_	C_19_N	C_20_OH	C_20_NH_2_	C_21_O_2_H	C_21_ONH_2_
C_25_	C_24_N	C_25_OH	C_25_NH_2_	C_26_O_2_H	C_26_ONH_2_
C_30_	C_29_N	C_30_OH	C_30_NH_2_	C_31_O_2_H	C_31_ONH_2_
C_35_	C_34_N	C_35_OH	C_35_NH_2_	C_36_O_2_H	C_36_ONH_2_

Figures 2 provides an overview of all conformers obtained for the cluster variants without functionalization, that is, C_*n*_ and C_*n*–1_N. The final structures show a wide variety of topologies, including linear, monocyclic, polycyclic, and cage structures. It is worth pointing out that the structures of C_*n*_ clusters have already been studied and characterized several times before.^57–61^ Linear and monocyclic structures have already been found as a peculiar feature of these species comprising a relatively small number of atoms. Here, we have recomputed the structures of the C_*n*_ to provide a test of our algorithm and to ensure the reliability of the results pertaining to the doped ones. Our results are consistent with previous findings.

**Figure 2:**
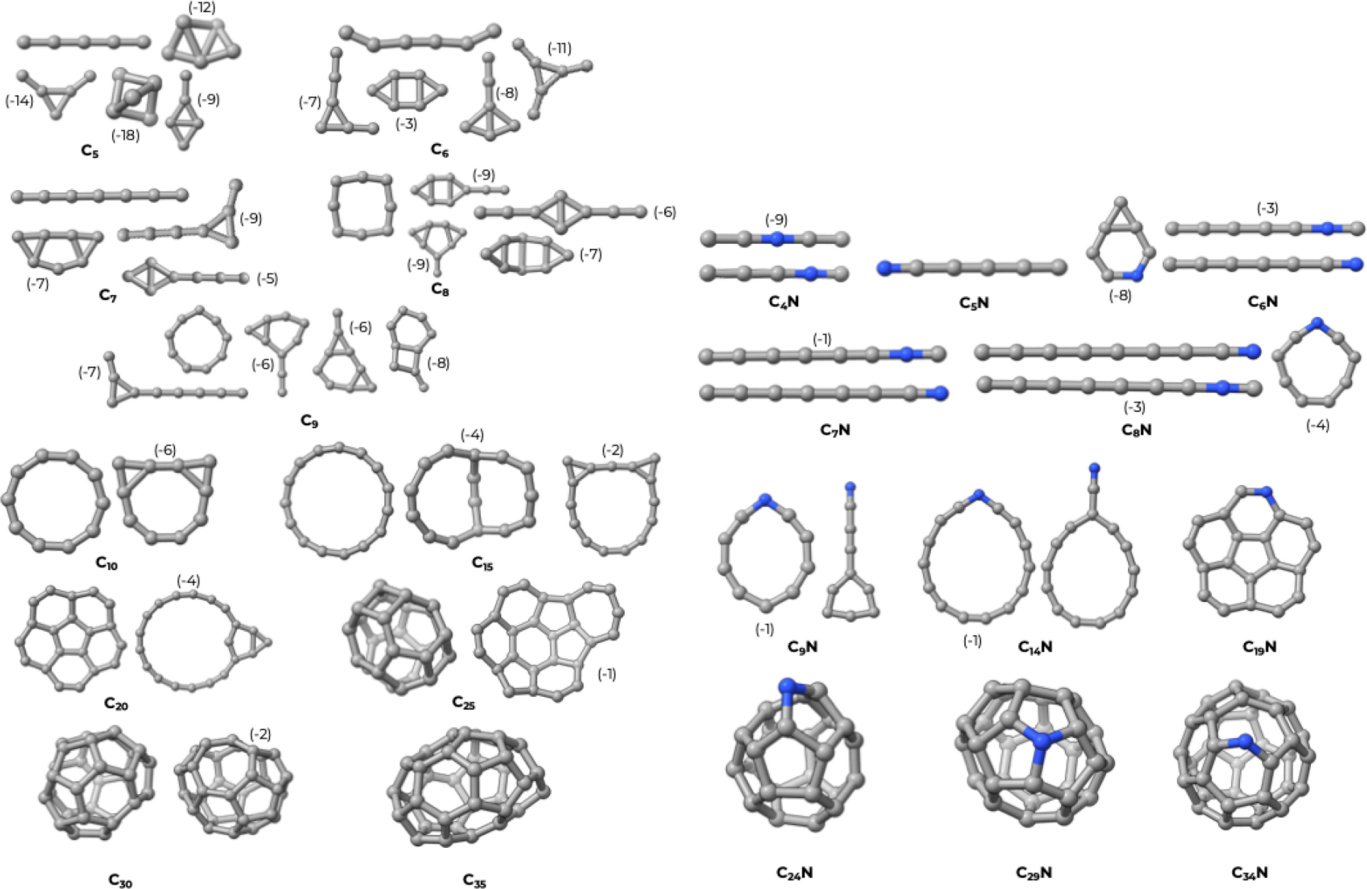
3D structures for neat C_*n*_ and C_*n*–1_N clusters. The negative numbers in parentheses indicate the relative atomization energies per atom in kcal/mol, calculated with respect to the most stable isomer (the more negative the number, the less stable is the cluster). In the few cases where the difference in atomization energy was less than 1 kcal/mol, we omitted the energy indicator.

Generally, for *n* < 7, the C_*n*_ clusters (up to seven carbon atoms) present a most stable isomer that has a linear structure. Higher-energy isomers show triangular and other cyclic shapes. As size increases beyond eight to nine carbon atoms, the linear structure is not stable anymore, and the carbon chain tends to fold on itself forming regular monocyclic structures such as those of C_10_ and C_15_. The presence of an N atom within the carbon cluster tends to stabilize the linear geometries and make the formation of small cycles energetically penalizing. Loss of linear geometry occurs only upon reaching 10 atoms, i.e., for the C_9_N cluster.

As the number of atoms moves from 10 to 15, for both C_*n*_ and C_*n*–1_N families, the monocyclic form is still predominant (C_15_ and C_20_), although in the case of N-doping, we notice the appearance of ″pendant″ structures where a portion of the cluster assumes a linear configuration (C_9_N and C_14_N). At C_20_ and C_19_N, we have the onset of the transition to a fullerene-like structure, at least partially. Both clusters show the formation of a partial cage with six- and five-membered rings reminiscent of a graphenic wall.

As the number of atoms increases to about 25–30 atoms, the polycyclic structures containing five- and six-membered rings begin to emerge as the most stable ones, forming partially closed and closed cages. These larger structures follow the pentane rule, which explains why five- and six-membered rings are energetically favored in amorphous carbonaceous materials. The formation of such rings relieves strain while preserving a degree of sp^2^ conjugation. However, the presence of pentagonal rings induces curvature, leading to the formation of buckyball-like cages, which do not resemble typical aromatic systems, a feature expected from the lack of border saturation.

The structures of the C_*n*_-NH_2_ and C_*n*_-OH clusters families are shown in Figure 3. The functional group has been held together using suitable bond constraints during the many optimization steps of the conformer search algorithms. However, in a single case (C_15_-NH_2_), we were unable to keep the NH_2_ group intact and the only conformers that emerged show the formation of pyrrolic-like or pyridinic-like function. The general features already described for the C_*n*_ and C_*n*–1_N classes are also present with NH_2_ and OH. The onset of fullerene formation is at 20 carbon atoms, when hexagonal and pentagonal structures appear. The first cage-like structures appear at 25 carbon atoms. The dopant group remains on the surface of the cage pointing outward because of the constraints and of its steric hindrance.

**Figure 3:**
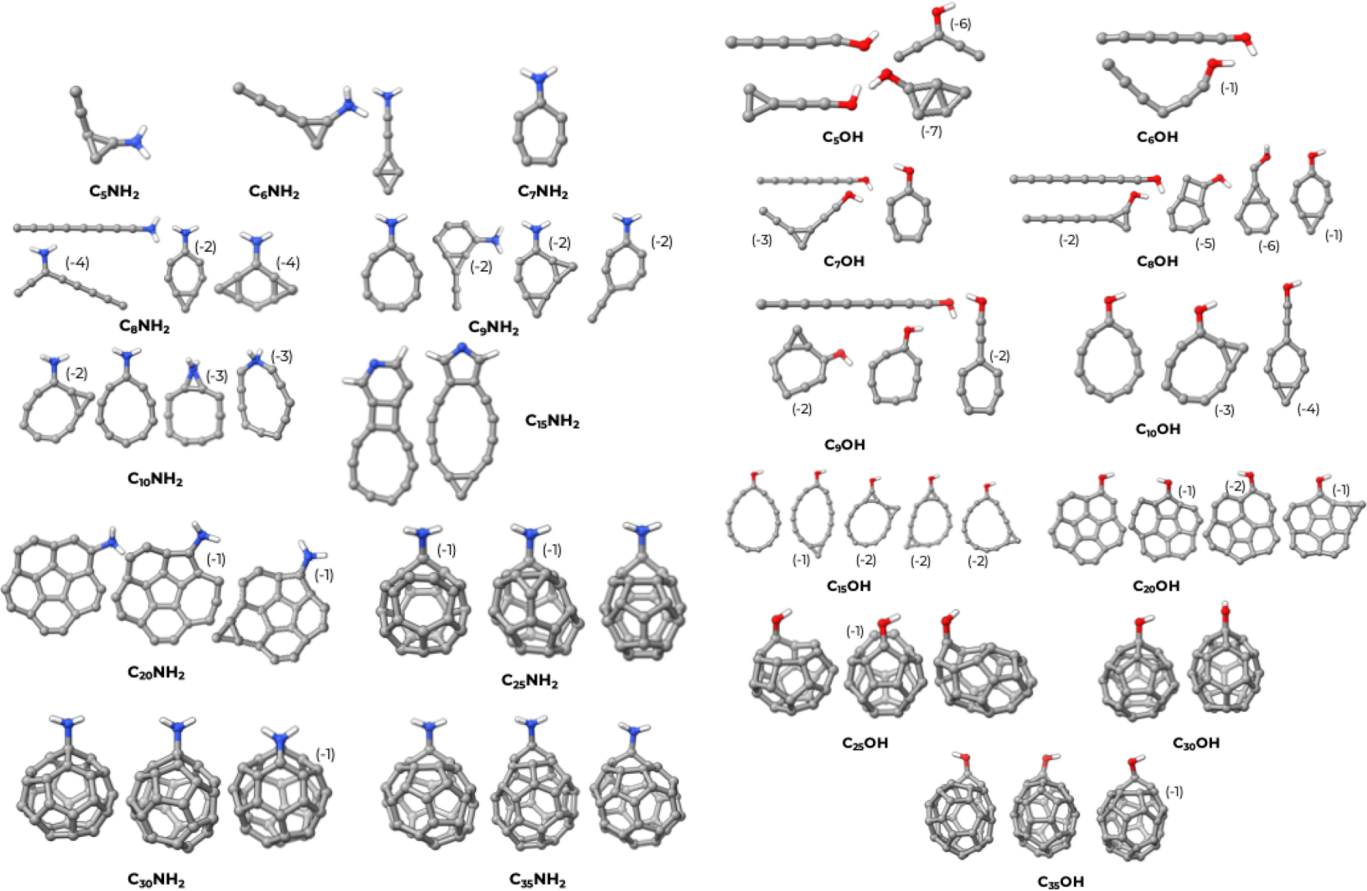
3D structures for C_*n*_-NH_2_ and C_*n*_-OH clusters. The numbers in parentheses indicate the relative atomization energies per atom of the structures in kcal/mol, calculated with respect to the most stable isomer. In the few cases where the difference in atomization energy was less than 1 kcal/mol, we omitted the energy indicator.

A similar description can be applied to the structures of the C_*n*_-COOH and C_*n*_-CONH_2_ families, as shown in Figure 4. Despite the use of constraints, in both families, the carbonyl oxygen tends to attach itself to the carbon skeleton, giving rise to rings of different sizes and fusing into the cage.

**Figure 4:**
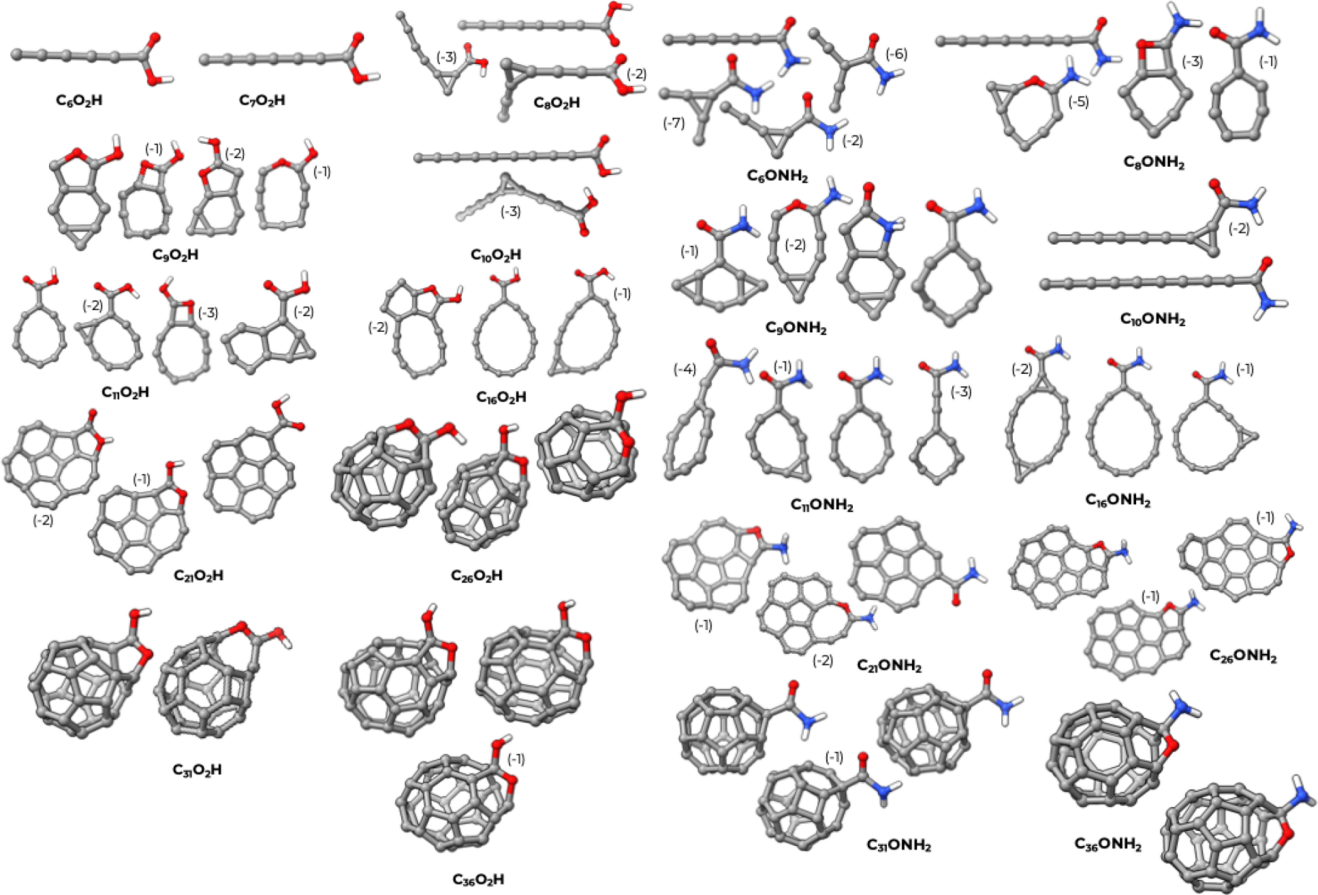
3D structures for the C_*n*_-COOH and C_*n*_-CONH_2_ clusters. The numbers in brackets indicate the relative atomization energies per atom of the structures in kcal/mol, calculated with respect to the most stable isomer. In the few cases where the difference in atomization energy was less than 1 kcal/mol, we omitted the energy indicator.

### 3.1 Atomization Energies

To assess the stability of the analyzed clusters and the effect of functionalization or doping, the atomization energy was calculated. Atomization energy was defined as the difference between the total energy of the individual atoms in vacuum—each appropriately scaled by the number of atoms present in the cluster—and the total energy of the cluster itself, normalized by the number of atoms in the cluster. The atomization energy was computed by using the following equation:

$$E_{\mathrm{atom}}=\frac{\sum_{i}N_{i}E_{i}-E_{\mathrm{cluster}}}{\sum_{i}N_{i}}$$ 1

where *N*_*i*_ and *E*_*i*_ represent the number and energy of atom type *i*. For clusters with a functional group, the atoms belonging to the group are included in eq 1; therefore, the atomization energy also accounts for the atomization of the functional group. With this choice, the atomization energy is simply proportional to the stability of the cluster. Figure 5 reports the atomization energies for all clusters reported whose structures are in Figures 2, 3, and 4. Each class of different size has been marked by a different color, and each family has its own energy plot. For all families, the atomization energy grows almost linearly with the cluster size. This trend is expected since larger structures tend to form more stable allotropic carbon phases increasingly similar to fullerenes. Compared to neat carbon clusters, functionalized and doped clusters generally exhibit slightly higher atomization energy, indicating greater stability. However, the overall stabilization effect introduced by functional groups or doping is relatively modest, with energy differences between clusters of the same atomic size, but different functionalization, reaching only a few kcal/mol.

**Figure 5:**
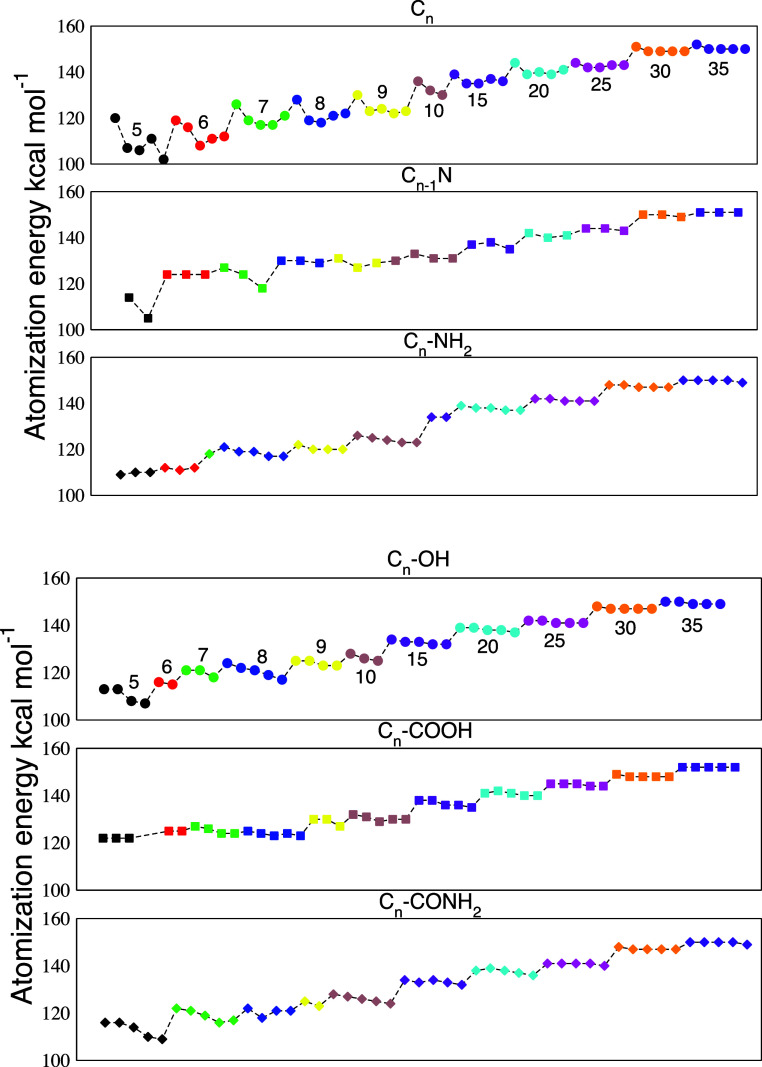
Atomization energy trends for all six classes considered. From top to bottom: C_*n*_, C_*n*–1_N, C_*n*_-NH_2_, C_*n*_-OH, C_*n*_-COOH, and C_*n*_-CONH_2_. Each value of *n* is identified by a different color consistently across the classes. The correspondence between color and *n* is explicitly reported in the top panels.

We remark that in this study, we have limited the space of possible functionalization to a single functional group per cluster. In real carbon nanodots synthesized via bottom-up approaches, functionalization is much richer, with a variety of surface groups typically present. This would likely have a much more pronounced impact on the overall structure and stability. We think, however, that isolating the effect of a single dopant or substituent is useful to understanding the intricacies of more complex setups. For example, despite the single functional group, we can clearly see a difference in the pattern of stabilities across the cluster growth. The neat carbon clusters show the most pronounced energetic variation across isomers of a given *n*. This happens to such an extent that isomeric structures for a given *n* can be less stable than those with *n–*1. This effect can be clearly grasped by the presence of peculiarly stable structures (reminiscent of magic numbers) corresponding to distinct peaks in the energy profile in Figure 3. By contrast, all other classes of clusters show a more uniform pattern of stability within a class of size *n* and a smooth scaling with it. With sporadic exceptions pertaining to small *n* values and for the C_*n*–1_N family, all of the energic variations among isomers of the doped/functionalized clusters are quite limited and much less pronounced than for neat C_*n*_. This implies less variability of the isomers across a family of size *n.* Rather than providing an increase stability in terms of cohesive energy, doping or functionalization reduces the energy variation among the available isomeric forms at low energies.

### 3.2 Hybridization Ratios

Hybridization ratios were calculated for the six families of clusters and are shown in Figure 6. These ratios were obtained using RDKit, a tool that employs graph-based heuristics to determine hybridization states. The data in each column correspond to the average ratios across all identified isomers. Hence, the data are representative of the entire set of isomeric structures for a given *n*. These data show clearly the coexistence of different carbon hybridization patterns within the same structure that makes these clusters different from the pure allotropic forms of carbon.

**Figure 6:**
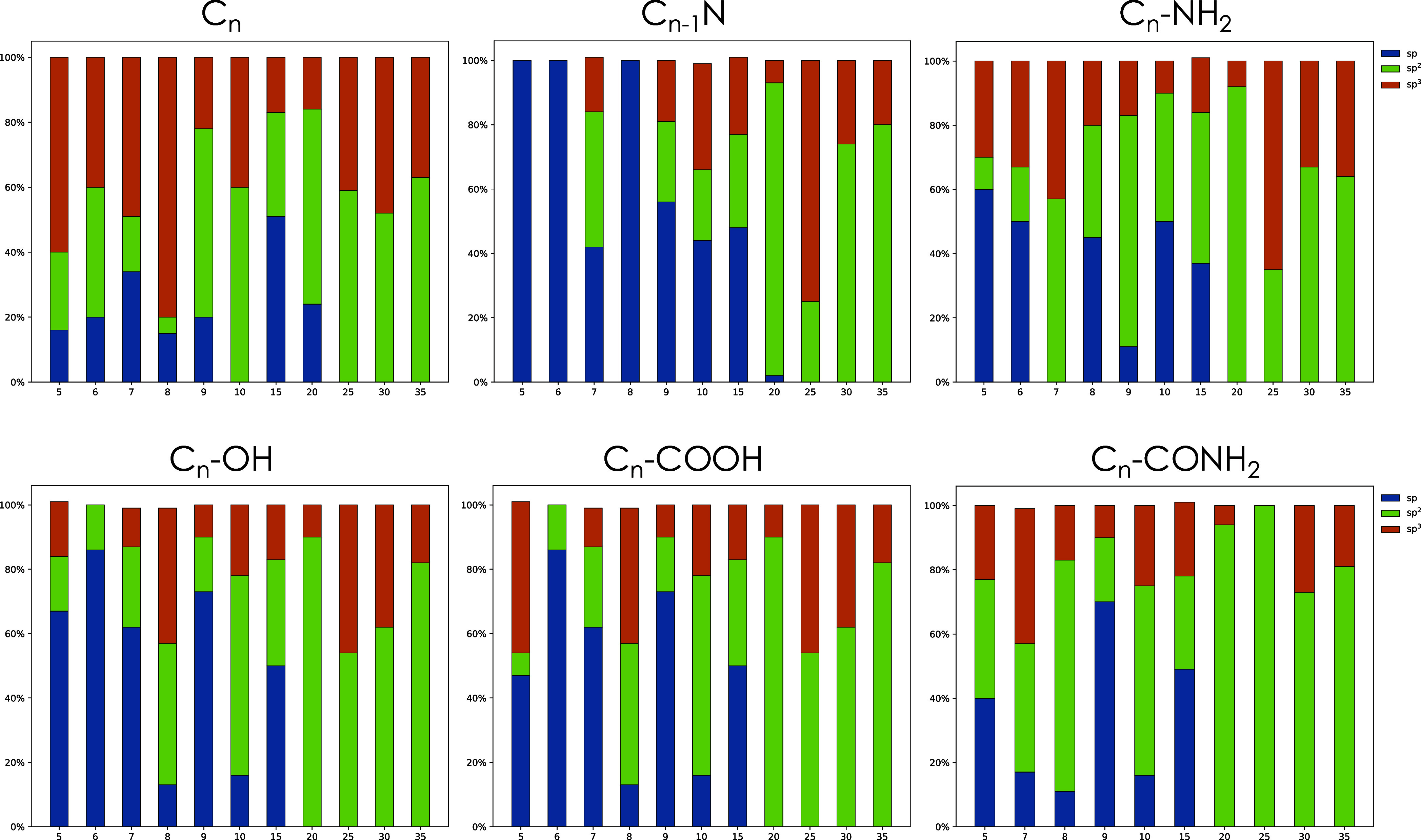
Hybridization patterns (as percentile) of the carbon atoms in each cluster of the six families. The hybridization pattern was analyzed for each structure, and average values were reported for clusters of the same size. In blue the sp content, in green the sp^2^, and in orange the sp^3^.

This analysis reveals some general trends in the hybridization percentages. In small clusters—particularly those with five, six, or seven atoms—the sp fraction is very high, which is expected given their predominantly linear structures. As monocyclic and polycyclic structures form, the sp content decreases to zero, while a mixed sp^2^ and sp^3^ hybridization emerges. This trend is consistent with data reported in the following section regarding average bond lengths for the structures considered. In larger clusters when the cages are the main structural motif, sp^2^ hybridization becomes dominant.

The clusters corresponding to a carbon core of 20 atoms (C_19_N for the N-doped ones) mark the transition to a cage-like structure. Except for the C_*n*_ case where the average includes the contribution of a cyclic structure (Figure 2), all C_20_ clusters show a nearly complete sp^2^ carbon composition. Their structure is, in fact, nearly planar, but the lack of saturation prevents them from acquiring the aromatic configuration typical of PAHs. This happens also for another large structure pertaining to the C_25_ series: the open cage C_26_ONH_2_ specie (see Figure 4). The lack of saturation on the edges prevents these quasi-planar structures from growing further.

### 3.3 Average Bond Lengths

The average carbon–carbon bond lengths extracted from the clusters are shown in Figure 7. The color circles identify the average values for a given dimension *n*, and the open circles are the average values for each isomer. In accordance with the structural characterization above, one can notice how the C_*n*_ family of clusters (red circles) shows the widest variation of C–C distances, while the presence of a single dopant group or atom significantly inhibits it by reducing the structural variety. A limiting case is represented for this aspect by the C_*n*–1_N cluster (green circles) that seems to induce the presence of a very limited number of combinations among of the C–C bonds, especially for small *n* where the doping nitrogen forces the clusters to assume a linear geometry. The results for the small cluster with *n* up to 15 indicate an intermediate situation between sp, sp^2^, and sp^3^ hybridization, consistent with the previous analysis on hybridization ratios. Clusters with the highest sp content, predominantly the smaller ones, exhibit the shortest bonds, as expected. In contrast, larger clusters display average bond lengths between those of single and double carbon–carbon bonds. As the size of the cluster grows, independent of the doping agent, the carbon bonds tend to the geometry of graphene according to the gradually dominant sp^2^ hybridization pattern. The computed bond lengths for *n* > 20 are comparable to those observed in graphene-based structures, which are commonly used as models for carbon nanodots. Specifically, in graphene, the average bond length for C–C is 1.42 Å.

**Figure 7:**
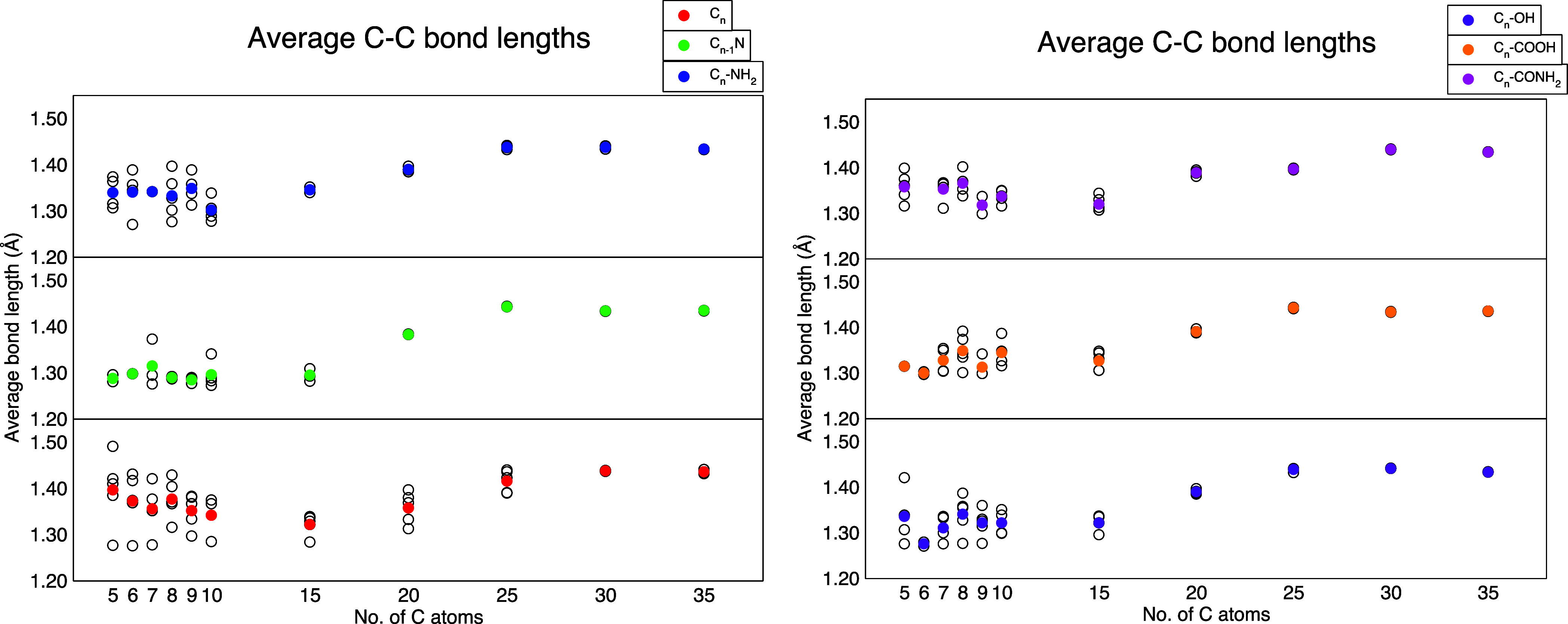
Average (colored circles) and isomer-specific (white circles) C–C bond lengths. The *x*-axis refers to the number of carbon atoms in the corresponding neat C_*n*_ clusters before doping or functionalization.

### 3.4 Localized Orbitals and HOMO–LUMO Energy Gap

To investigate the reactivity of these systems, we have computed the localized HOMO and LUMO orbitals for one representative cluster of each class derived from the corresponding C_20_, that is, for the cluster C_19_N, C_20_OH, C_20_NH_2_, C_21_O_2_H, and C_21_ONH_2_. The localized versions of the HOMO and LUMO orbitals are reported in Figure 8. From this kind of analysis, one can qualitatively grasp the stability of the clusters and, therefore, their potential reactivity. In general, when the LUMO is localized on the functionalized part of the cluster, the functional group itself could easily promote interactions with other nucleophilic species, like biomolecules. Plus, if the HOMO is localized on the nonfunctionalized part, then it means the functionalization does not significantly alter the electron density.

**Figure 8:**
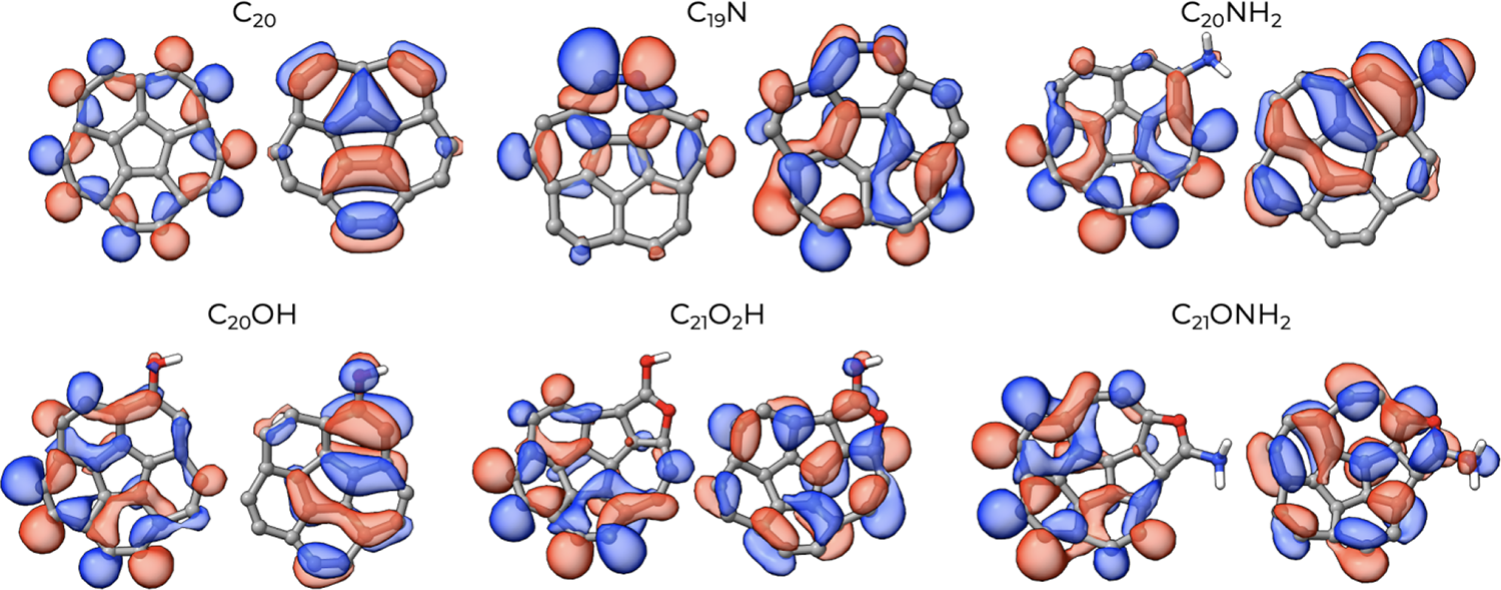
Localized HOMO (left) and LUMO (right) orbitals for the six clusters of size *n* = 20.

In neat carbon cluster C_20_, the HOMO is localized along the edges of the structure. The structure is not perfectly planar (like in coronene), thus hindering a classification by symmetry, but nevertheless the HOMO does not present a nodal structure traversing the carbon “plane”. On the contrary, the LUMO that is localized also in the inner region of the molecule has clearly a symmetry that resembles a π orbital with a nodal surface lying on the carbon “plane”. In C_20_, both the edges and the center ring are likely sites of a nucleophilic attack.

In the corresponding N-doped C_19_N, the HOMO is strongly asymmetric and entirely shifted toward the portion of the molecule where N resides. Also in this case, the orbital does not have a nodal surface lying on the nearly planar carbon structure. The corresponding LUMO is entirely delocalized across the whole structure and shows (at least partially) a change in phase when crossing the nuclear region. In C_20_-NH_2_, the situation is reversed due to the saturation of the amino group: The HOMO is localized far from the functional group, while the LUMO (of nearly π symmetry) encompasses it. These orbital shapes are similar to those shown by the C_20_-OH clusters. In C_20_-COOH and in C_20_-CONH_2_, there is a marked delocalization of both HOMO and LUMO across the whole polycyclic structures, but the functional group does not appear to be directly involved.

To further investigate the possible reactivity of the structures considered, the energy of the HOMO–LUMO (HL) gap was computed and is reported in Figure 9. For open shell species, we used the Fock eigenvalue of the SOMO and the one corresponding the first virtual β orbital. The HL gap of neat carbon clusters (C_*n*_) decreases with increasing size, ranging from 8 eV for small clusters (C_5_–C_1__0_) to 5 eV gaps in larger ones (C_3__0_–C_3__5_). This trend is consistent with a more pronounced π-electron delocalization, which leads to a denser orbital energies. When a heteroatom is introduced in the structure, as is the case of nitrogen doping (C_*n*__–1_N), this causes a notable reduction in the gap for most structures. A simple relation between the energetic stability of the clusters (Figure 5, where their atomization energies are displayed in the same order) and the HL gap is not systematic, if it exists at all. One common feature to all families is a drop in the variance of HL gaps across the isomeric class of a given *n* when their structure assumes a cage-like shape. This happens at *n* = 25 for most of the families except for C_*n*_-CONH_2_.

**Figure 9:**
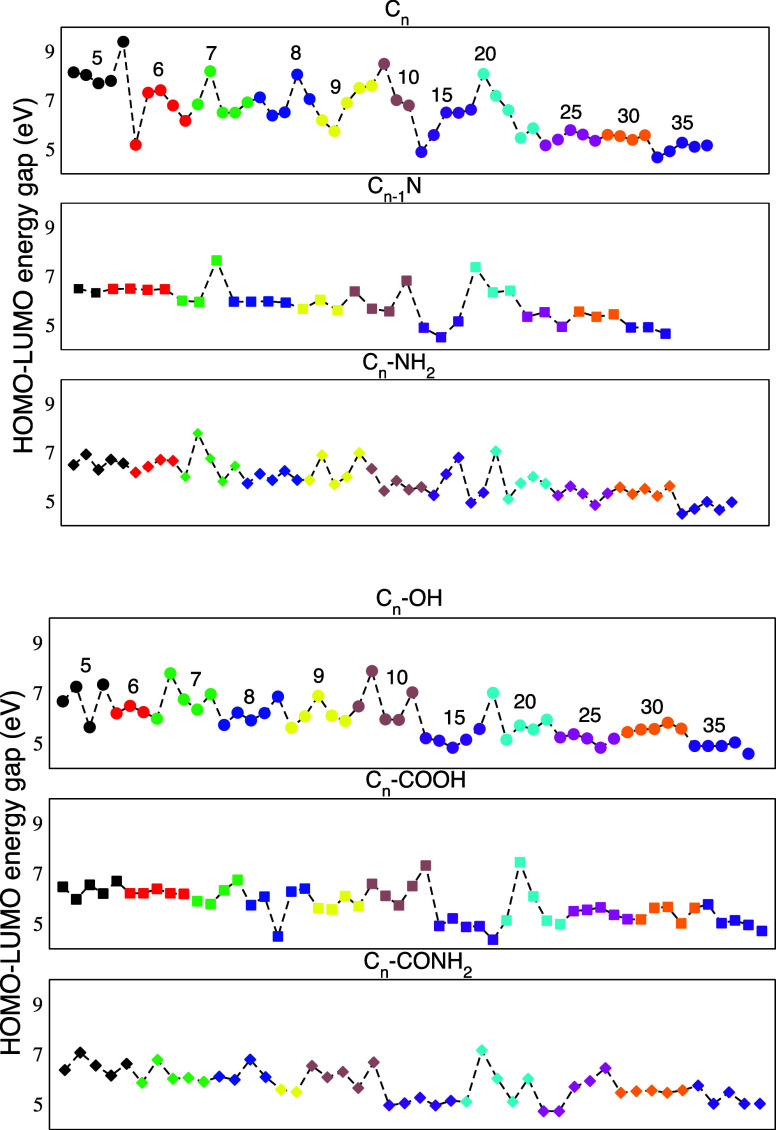
Energy of the HOMO–LUMO gap for all six classes of clusters considered (C_*n*_, C_*n*–1_N, C_*n*_-NH_2_, C_*n*_-OH, C_*n*_-COOH, and C_*n*_-CONH_2_). Each value of *n* is identified by a different color consistently across the classes. The correspondence between color and *n* is explicitly reported in the top panels. The order for the structures is the same as that in Figure 5.

Clusters functionalized with NH_2_ (C_*n*_-NH_2_) show a moderate decrease in the gap compared with neat systems, but the reduction is less pronounced than in the N-doped clusters. This is probably caused by the −NH_2_ group, which, being a mild electron donor, primarily raises the HOMO level, resulting in a smaller gap. All of the other functionalized systems exhibit a lower HOMO–LUMO gap compared to neat carbon clusters. Among them, the C_*n*_-OH clusters show a relatively small reduction, while C_*n*_-COOH and C_*n*_-CONH_2_ systems lead to a more pronounced gap narrowing, especially in midsized clusters (C_1__0_–C_2__5_). These trends can be explained by considering the electronic nature of the functional groups considered. The −OH group acts as a weak electron donor, mainly affecting the HOMO level, with a limited influence on the LUMO. In contrast, the −COOH and −CONH_2_ groups are electron-withdrawing: they tend to lower the LUMO, effectively reducing the HL gap. These effects suggest increased electronic activity and potential for enhanced conductivity or catalytic behavior in functionalized systems.

**Figure 10:**
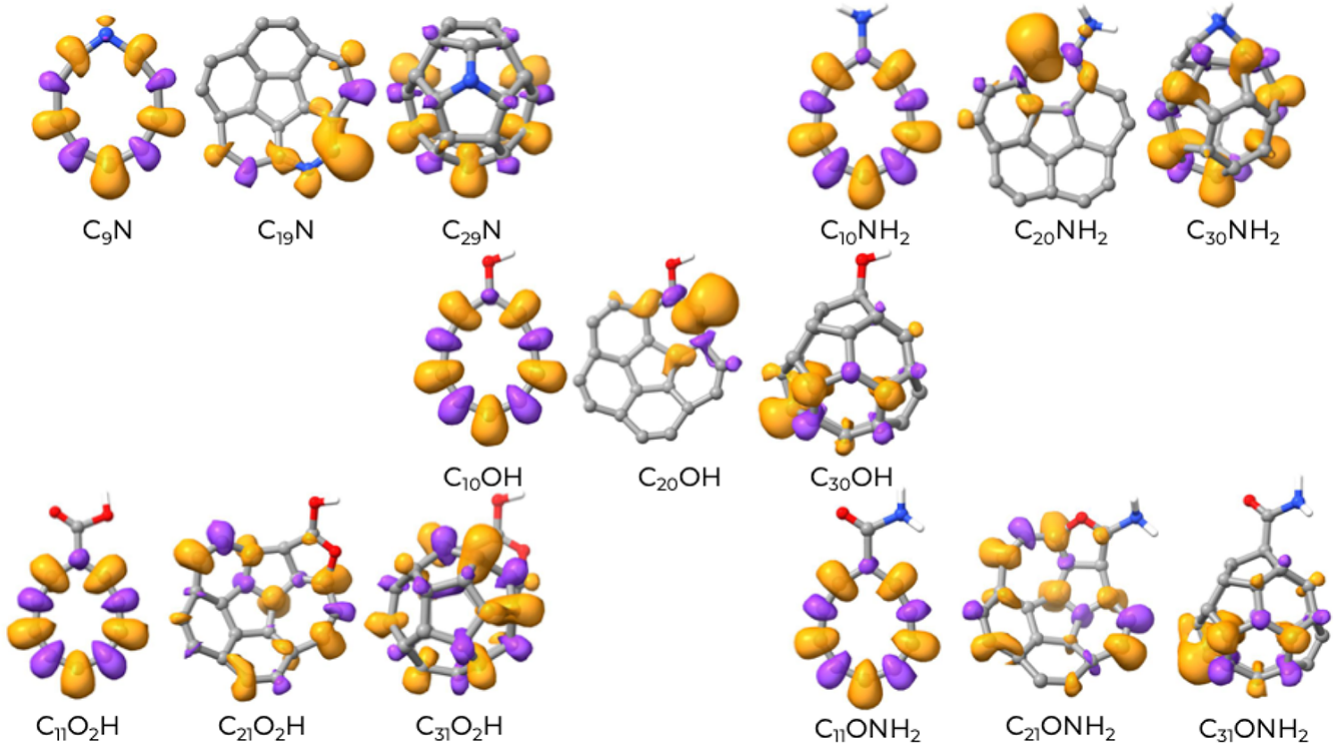
Spin-density maps for the five classes are reported here.

### 3.5 Spin Density

Except the pure C_*n*_ ones, the other families of clusters have an odd number of electrons and were treated as doublet states. The radical nature warrants an investigation of their spin density that provide insights into the distribution of the spin polarization within the molecular system. A highly localized spin density, concentrated on a few atoms, is characteristic of isolated radicals or strongly localized electronic states. Conversely, if the spin density is distributed across multiple atoms, then this suggests spin delocalization, which may arise from π conjugation or exchange interactions with neighboring atoms. The spin density is shown in Figure 10 for the series of clusters derived from the neat structures C_10_, C_20_, and C_30_. Certain atoms may exhibit negative (purple) or positive (orange) spin density, indicating respectively a deficit or an excess of spin α polarization. In the N-doped clusters, a marked difference in spin excess distribution can be noted and linked to the structural features. In the small monocyclic cluster (C_9_N), contrary to our intuition, the excess of spin α is on alternating positions on the carbon ring but not on the nitrogen. The prevalence of sp hybridization (Figure 6) allows for a redistribution of the spin excess along the carbon chain. In C_19_N, which has a nearly planar structure almost entirely sp^2^ in nature, the unpaired spin density is localized almost completely on the carbon adjacent to the nitrogen atom. In C_29_N, which is an sp^2^-dominated cage-like structure, the unpaired spin density is delocalized across the cage, but far from the doping nitrogen. Interestingly, a saturated nitrogen (NH_2_) produces almost the same effect as a skeletal nitrogen. As shown by Figure 10, the excess spin either delocalizes over all carbon structures or it localizes on the carbon site adjacent to the substituent also for −OH. It appears that the specific nature (N, −NH_2_, or −OH) of the doping or the functionalization has no peculiar effects on the distribution of the spin density, which is essentially determined by the shape of the carbon structure.

Few relevant differences can be noted for −COOH and −CONH_2_ functionalization. While the monocyclic and cage-like structure appears to be in line with what we have seen, the planar structures C_21_O_2_H and C_21_ONH_2_ present a much more pronounced delocalization of the excess spin over the carbon nearly planar structure.

## 4 Conclusions

In this study, we proposed a bottom-up computational approach to model the amorphous carbon core of carbon nanodots (CNDs), based on unsaturated and partially functionalized carbon clusters. Our results reveal a structural evolution that reproduces the known formation mechanisms of fullerene-like materials and other carbonaceous nanoparticles, starting from linear and monocyclic units in small clusters to larger polycyclic and cage-like motifs in larger ones.

Partial substitution with one nitrogen atom or functionalization with a single functional group (−NH_2_, −OH, −COOH, −CONH_2_) significantly modifies the local electronic features—particularly the spin density and the distribution of frontier orbitals—while largely preserving the overall carbon geometry and energy. Actually, both doping and functionalization tend to reduce the conformational freedom of the resulting structures, making the identification of the lowest-lying isomers easier and almost unambiguous. The structural rigidity, combined with variable HL gaps, hint at the role of such structures as realistic building blocks for the disordered carbon domains within CNDs.

Moreover, the nature and shape of the localized frontier orbitals and a certain control over the unpaired spin delocalization that appears to depend more on the structure of the carbon framework than on the peculiar substituent, highlight the possible roles of hypothetic amorphous domains analogous to these clusters in charge trapping, interlayer reactivity, and photophysical behavior. These are all crucial issues in understanding the optoelectronic and catalytic performances of CNDs.

Although the clusters studied in this paper are still far from representing a viable model of an entire CND, we think that the study of relatively small units amenable to an investigation at the highest level of theory is important to understand the behavior of more complex and large systems where such studies are limited by computational costs and efficiency. This methodology can be further extended to explore multisite functionalization, partial and progressive saturation, larger cluster assemblies, and aggregation processes, thus aiming at understanding from first-principles part of the structure and functionality of carbon nanodots in specific applications.

## Supplementary Material



## Notes

All cluster structures in “xyz” format; electronic energies of the clusters and those of the isolated atoms are also reported; S1, C_*n*_N family; S2, C_*n*_ family; S3, C_*n*_NH_2_ family; S4, C_*n*_CONH_2_ family; S5, C_*n*_COOH family; and S6, C_*n*_-OH family (PDF)
