# Supplementary material for: A Bottom-up Computational Study of Doped Amorphous Carbon Clusters as Precursors of Carbon Nanodots
Source: J Phys Chem A. 2025 Jul 21;129(30):6825–36. doi: 10.1021/acs.jpca.5c03577 (PMC12319916; doi:10.1021/acs.jpca.5c03577)
Supplement: Supplementary file 1 [file jp5c03577_si_001.pdf]

A Bottom-up Computational Study of Doped Amorphous Carbon Clusters as Precursors of Carbon Nano-dots.

Francesca D'Ambrosio[1]*, Alice Frustaci*[1]*, Enrico Bodo*[1]

[1]Chemistry Department, University of Rome "La Sapienza", P. Aldo Moro 5, 00185, Rome Italy.

Supporting information

S1 C_nN family: Energies (Hartrees) and xyz coordinates (Angstroms)

```
C4N_a -206,88374
C4N_b -206,81162
C5N_a -245,00798
C5N_b -245,00796
C5N_c -245,00799
C6N_a -283,08024
C6N_b -283,04266
C6N_c -282,98481
C7N_a -321,16587
C7N_b -321,16589
C7N_c -321,15466
C8N_a -359,23777
C8N_b -359,17306
C8N_c -359,19967
C9N_a -397,27780
C9N_b -397,32194
C9N_c -397,28382
C9N_d -397,28787
C14N_a -587,69614
C14N_b -587,73190
C14N_c -587,65773
C19N_a -778,18178
C19N_b -778,13706
C19N_c -778,15832
C24N_a -968,61507
C24N_b -968,62423
C24N_c -968,59582
C29N_a -1159,27634
C29N_b -1159,26977
C29N_c -1159,25970
C34N_a -1349,79627
C34N_b -1349,79643
C34N_c -1349,77565

C -37,84496
N -54,58957

Structure: C14N_a.xyz
15
Coordinates from ORCA-job 15N.globalminimum E -587.696146163941
  N          3.60519590710520     -0.00002004390627      0.00802704247260
  C          2.87373486031325      1.07209903056148      0.00621073553102
  C          2.11028277231185      2.03397207244213      0.00485700510730
  C          0.90285005416226      2.62848227978829      0.00205799784792
  C         -0.29389900987114      2.87645060721981     -0.00052735701412
  C         -1.60380946848812      2.53489817253876     -0.00352934528347
  C         -2.59372007809844      1.82492053533645     -0.00587184801897
  C         -3.19819732086103      0.60849765397119     -0.00722617429962
  C         -3.19810215162620     -0.60846771625733     -0.00718993705066
  C         -2.59374293969203     -1.82494331728828     -0.00577713319631
  C         -1.60373209012788     -2.53478173904223     -0.00343735400551
  C         -0.29386547373311     -2.87644171571475     -0.00050534440094
  C          0.90287564871613     -2.62844112330815      0.00200744646347
  C          2.11034888204174     -2.03404992194646      0.00475035882882
  C          2.87378040784752     -1.07215477439465      0.00615390701846


Structure: C14N_b.xyz
15
```

```
Coordinates from ORCA-job 15N.struttura2 E -587.731898308764
  N           4.75417402887395      1.87116796458021      0.00869524897790
  C           3.68360504139267      1.44923806598061      0.00758418884900
  C           2.36220169094469      0.92855974423652      0.00557851870755
  C           1.22996475291067      1.74559721306284      0.00348511742600
  C           0.06739019245448      2.11161841417052     -0.00046666198740
  C          -1.29221937391651      2.09703714815320     -0.00264341552849
  C          -2.40209056187176      1.60056827806478     -0.00626678561816
  C          -3.28129328105786      0.55653867127936     -0.00657161428285
  C          -3.49492805906083     -0.63922764050804     -0.00857354850263
  C          -2.99333604233387     -1.91170619018192     -0.00521954238227
  C          -2.02422297208869     -2.64396344274307     -0.00502966820359
  C          -0.66889983196767     -2.80744109598314     -0.00012169360687
  C           0.48235697585371     -2.41658919917220      0.00049559122028
  C           1.48737160981113     -1.50043772425219      0.00318673111755
  C           2.08992582476412     -0.44094019320546      0.00586753381396
```

```
Structure: C14N_c.xyz
15
Coordinates from ORCA-job 15N.struttura9 E -587.657735968391
  N           3.00498928347034     -0.13666275696107      0.00900508372875
  C           2.68212529388761      0.99354864685127      0.00766632456028
  C           1.95249628866571      2.11706327541643      0.00542500070914
  C           0.87901697886255      2.70753474894577      0.00240695168184
  C          -0.42564643534169      3.06408735502159     -0.00062380981260
  C          -1.57697760512388      2.65309675387478     -0.00449676630326
  C          -2.69864301889213      1.88908536183987     -0.00878691864631
  C          -3.05377182443137      0.71878785028818     -0.01107845416285
  C          -3.12969163615462     -0.63434903788929     -0.01236914430048
  C          -2.43379835743356     -1.64265434066282     -0.00747260829582
  C          -1.46580284140726     -2.58296686969463     -0.00415520538029
  C          -0.25170165808352     -2.78149743072122      0.00331744781474
  C           1.08780295667727     -2.79815982370975      0.00467863888064
  C           2.14932888695270     -2.15708899117957      0.00754710133450
  C           3.28027368835186     -1.40980473852106      0.00893635289996
```

```
Structure: C19N_a.xyz
20
Coordinates from ORCA-job 20N1.globalminimum E -778.181772440130
  C          -0.80975146737792      0.27067290223225      0.46150811483158
  C           1.12769058086296      1.20853381812322     -0.46379038687503
  C           0.56002213099056      0.06238062320140      0.08552916129053
  C           2.84364494235250     -0.63544595109008      0.29038309714559
  C           1.62957765561325     -0.83028566535832      0.34920390563875
  C           4.68112073853215      1.32305130799676      0.16932235753123
  N          -1.43539038772259      1.36563803482383      0.48463221858167
  C           0.43652465910046      2.44287884838772     -0.46620247132580
  C           2.52581625308048      1.47511874350491     -0.53529705122125
  C           2.69062734573342      2.89252421573740     -0.56499681171438
  C           3.49995030581328      0.57498077499346     -0.04884081250335
  C           4.82553177935897      2.54487811651015      0.14329058276170
  C          -0.81811292071472      2.53920661203334      0.11461985319348
  C           1.14124004815512      4.78628764949040     -0.01619719576239
  C           1.39351647184673      3.49205092779437     -0.52225669429880
  C           2.39777221293989      5.39666428702216      0.20688808186045
  C           3.86286569519866      3.54741541355375     -0.12363107208446
  C           3.51789836010923      4.88936476530680      0.16109777392545
  C          -1.04186606991979      3.91361028060223      0.39949593093513
  C          -0.22781833770570      4.83589429615218      0.34620141278142
```

Structure: C19N_b.xyz
20
Coordinates from ORCA-job 20N1.struttura3 E -778.137059673446

```
  C          -0.06514977670009      0.58247992027542      0.13246121315938
  C           0.94403108678010      1.35531495635770     -0.60671074931964
  C           0.36847773072411     -0.65873998041387      0.56444768653207
  C           2.82891564763575     -0.64098198969888      0.33868737565907
  C           1.62886533112361     -0.99692434845401      0.48867079452984
  C           4.56177715421368      1.29498402817447      0.15187961366452
  N          -1.13767625428974      1.34667247049089      0.48087141309234
  C           0.38295684002187      2.60788185036881     -0.69861348597297
  C           2.37012823422107      1.50395843325147     -0.55903216693659
  C           2.63085699461341      2.93241609596212     -0.62026887480904
  C           3.34812739015344      0.58817985844676     -0.06650225620126
  C           4.74230061351477      2.50582627041525      0.14881444372528
  C          -0.80860774778492      2.58390224597642      0.06952328786710
  C           1.14247346976712      4.85189254717451     -0.00362115737463
  C           1.37746601954416      3.61050047203152     -0.63646195857936
  C           2.40592854056217      5.38693922138189      0.30026579695914
  C           3.81036528629425      3.54230924177288     -0.11653914655386
  C           3.52569918641579      4.87314323446933      0.22629534464159
  C          -1.03379978106776      3.95038202356267      0.44091693428165
  C          -0.22227596949586      4.87528344418082      0.43587588103514
```

Structure: C19N_c.xyz
20
Coordinates from ORCA-job 20N1.struttura5 E -778.158316754152

```
  C           0.42103811781472     -0.01913281386213      0.11332799508639
  C           0.84840977902451      1.27743757112605      0.07639634457411
  C           0.56270040760329     -1.36807493352219      0.12222482059087
  C           2.87873487814372     -0.05499329392965     -0.00965152539250
  C           1.83375662380024     -0.97968742314240      0.05399944418949
  C           4.83332330586072      1.32769633407559     -0.12547623525401
  N          -2.96003308088506      2.59256511714162      0.25651131127107
  C           0.55788400983647      2.75046648300988      0.07250648356352
  C           2.24783047178850      1.18969608697189      0.00700373667003
  C           2.86970374175381      2.43621204879483     -0.04085891339505
  C           4.27574325359776      0.23007941346280     -0.08382936161458
  C           4.25073951657175      2.63053209092420     -0.11268523332545
  C          -1.90262256894451      3.03510072053849      0.19598033316654
  C           0.90646073727326      5.44523053236399      0.02088738457469
  C           1.84821998419911      3.39393622252151     -0.00149777616567
  C           2.14003163868527      4.78230945812211     -0.03340088868351
  C           4.41455354918181      4.03986265083812     -0.13835437712537
  C           3.54896688257110      4.91691062339971     -0.10589684439011
  C          -0.57472237681086      3.55744203254874      0.12008429051957
  C          -0.19985887481867      4.91183107963475      0.08368900583152
```

Structure: C24N_a.xyz
25
Coordinates from ORCA-job 25N1.globalminimum E -968.615069276778

```
  C           1.13609787634765      1.57428735975872     -1.53582713252935
  C           0.53394065375900      2.38993737729965      2.20882553169093
  C           3.43333530660477      2.30017574423510     -1.84933219629243
  C          -0.69623647385934      3.28449588503969      0.48903445244979
  C           3.32226473017741      1.52351965169244     -0.68981343843978
  C           1.89719161670178      2.52036098560739      2.30175793431259
  C           0.05614129950548      3.64989152488745      1.57597306478382
  C           3.22613335879500      4.25971969969103     -1.55143970757841
  C          -0.08332803491388      2.06859657792696     -1.08596884342281
```

```
  C         -0.53973549349087      1.80847605752544      0.26458963217990
  C          3.48124872045046      2.19105414941683      0.58828680963661
  C          2.36528412345814      3.82110071292936      1.86514585298004
  C          1.60875235230463      0.81319518115221      0.77024792540394
  C          1.97493692561276      0.89257505823995     -0.58872835769665
  C          2.60798737967691      1.53002374414440      1.51698201994166
  C          1.54999315920493      4.90496885737545      0.02138194557895
  C         -0.23866022590602      3.53233567018368     -0.86363916126154
  C          3.32666324229944      3.65590524953016      0.81115592423821
  C          2.00667480843399      2.53139438651455     -2.23533023497764
  C          0.83990796391692      4.37537913199963     -1.10966578074466
  C          1.86543488319943      3.86515010412444     -2.03241041582655
  C          0.30504016090736      1.30181043254583      1.21867057540783
  C          2.99381388647747      4.62144138444711     -0.21896188019124
  N          4.21687305378704      3.38036455645402     -1.74316494696199
  C          1.21762472806857      4.50492051579155      1.33161040748547
```

```
Structure: C24N_b.xyz
25
Coordinates from ORCA-job 25N1.struttura2 E -968.624228026335
  C          1.84155822001305      2.82068906856798     -2.34671296640543
  C          0.50269168677721      2.45598464972564      2.24705419430271
  C          3.13815913824184      2.27742238451936     -1.85844698034205
  C         -0.45279393370715      3.10612771159318      0.21819409575373
  C          3.03037449778303      1.43272637888367     -0.69843891198060
  C          1.88929021435657      2.57624680142579      2.42284112573069
  C          0.07223086925134      3.63368214750876      1.41185457247680
  C          3.29299052307438      4.32202955999455     -1.57228387200925
  C          0.60065214996550      1.28715652987521     -0.88675437246308
  C         -0.26336777321446      1.63974202325469      0.27049147487241
  C          3.34740832278829      2.13238332080612      0.54735961322541
  C          2.32687224651691      3.78301361636710      1.86167244597703
  C          1.80132129477907      0.74694680286044     -0.39174408040129
  C          1.62541991789877      0.71071974344160      1.08723348268721
  C          2.57293754476385      1.50374638807562      1.64509668051241
  C          1.60613934667769      4.98010241727666      0.02570649842126
  C          0.02703902028804      3.54897345539823     -1.04278846780616
  C          3.28543901005676      3.56375225367160      0.77389189951496
  C          0.63535437394838      2.39946159333311     -1.76555458350675
  C          0.93210797056331      4.61996716884442     -1.16370729837131
  C          1.98303574811438      4.20917976504789     -2.12525490225383
  C          0.29464335931409      1.25427991726774      1.46637328146882
  C          3.03858670675234      4.58297110663210     -0.22278577232815
  N          4.08620488621099      3.19878849096031     -1.72556757033156
  C          1.19308466030485      4.51498670318131      1.28165040929806
```

```
Structure: C24N_c.xyz
25
Coordinates from ORCA-job 25N1.struttura6 E -968.595814287739
  C         -0.30338487210520      2.64180590137027     -0.96587635528007
  C          0.18380974917515      4.44572728344921      1.43864967471214
  C          3.90236255770480      1.69873466635528     -0.81457258210997
  C          0.44158238456266      1.44540324290815     -0.78042481559230
  C          2.74667461862042      0.82315291574321     -0.92978206858392
  C          1.98801322406521      2.34018156010927      1.96400402832859
  C         -0.28134976646117      3.04801936429841      1.42363965522728
  C          3.10963332524604      3.44759685267930     -1.70046867730781
  C         -0.90396068601854      3.27972578049712      0.10897807982174
  C          0.60093348134445      1.99464665841526      1.62663109354142
  C          3.97429040859286      2.13789084897347      0.56996828852945
```

C	2.46749635444614	3.61702964012317	1.69521175462876
C	2.20253584792133	0.64157364283682	0.43986964372350
C	1.71059657073097	1.51234275546876	-1.57410203425777
C	2.91598774076953	1.40582774951768	1.31124349589073
C	1.55130525556231	4.62997978255061	1.30131887343892
C	1.80696694005828	2.91094900745032	-2.05445002964252
C	1.87906773931200	4.91057168632680	-0.12342329975402
C	3.07570528508221	4.27120798564648	-0.50437410076280
C	-0.41799026797845	4.65419456907632	0.12735258801631
C	0.63571044216890	3.57772746900241	-1.64348649912698
C	0.79656853092486	1.06311015834594	0.50839622183806
C	3.53316777130047	3.46575613097171	0.65293738666020
N	4.12010116867390	2.58015373039657	-1.76331379324415
C	0.67155619781990	4.75777061600052	-0.85454653265276

Structure: C29N_a.xyz
30
Coordinates from ORCA-job 30N1.globalminimum E -1159.276337151731

C	0.03616686779893	2.56013985434541	1.60430244183892
C	-0.15077686044753	2.01268864218501	-0.63993597774057
C	0.20651153731321	4.91662523867819	0.86473496038868
C	1.45779719543192	5.03278236759386	1.55165932745146
N	-0.87312366774667	3.23310431014587	-0.36534886009303
C	-0.38058159895503	4.15258675869473	-1.20605102282507
C	0.75478281253057	2.24348357966267	-1.73816228572974
C	2.49397876765257	1.17761735795813	-0.33760877585167
C	2.15562336262201	1.66719431461281	1.88057310151339
C	1.31256125766716	5.61566070156466	-1.24869549960906
C	0.49837159994609	3.59847220504355	-2.18852791591403
C	2.86513291039937	4.13182551395038	-2.20037218181200
C	2.52404019250538	5.79465343684007	-0.58128560729643
C	0.25410033401177	1.49646019471895	0.56847208676274
C	3.72276802534170	4.59308604911595	1.01759254297666
C	3.70053251661923	1.86989953212289	0.20658874729176
C	1.58031547958010	1.02196082282960	0.71537046863253
C	2.11485483428605	1.82860360877119	-1.56784164390522
C	1.23787523639903	2.69181436227000	2.34150825198304
C	-0.48784831912975	3.65671316121865	0.96118179369143
C	1.93346946231218	3.90994795097434	2.32277879769324
C	3.34101180144659	3.62304264112627	1.91364366396133
C	4.08635044122423	2.82029947906721	-0.70851864876867
C	1.53399527930415	4.53600314472501	-2.29951594241998
C	0.14960670798432	5.29788125480024	-0.53371996170270
C	2.59843436037181	5.51880077384098	0.81420908933212
C	3.41392043444756	2.22929945862099	1.56856336008238
C	3.54360506225449	4.99601708232560	-1.24548004437473
C	3.15941707952773	2.78301069839823	-1.84946337975541
C	4.19494691021489	4.21390549167452	-0.30523087693244

Structure: C29N_b.xyz
30
Coordinates from ORCA-job 30N1.struttura2 E -1159.269772916173

C	0.10869644423123	2.65400380953367	1.58303810149212
C	-0.14921536398204	2.01998036846138	-0.62888515588447
C	0.02585012919522	5.01125538986383	0.82126823041724
C	1.80386443631613	4.20773388846661	2.36373532957818
N	-0.86470819567514	3.22032532210650	-0.39228274547709
C	-0.39615872661655	4.15279784162958	-1.28172894091027
C	0.74335663224569	2.22592071174925	-1.74145564024147
C	2.46973501328630	1.07696009503074	-0.39573282784711

C	2.30437595058609	1.91091594770598	1.78967410975643
C	1.36511545571061	5.56199709870172	-1.18324432307067
C	0.51012741025857	3.59172471231517	-2.19900547954070
C	2.89234619624996	4.05578547325164	-2.10054199251501
C	2.55642964260420	5.67348918925533	-0.38991374108380
C	0.31688907452331	1.56941315062974	0.60670812709444
C	2.41700229242690	5.63878130983580	1.00150869698625
C	3.75010458367764	1.65327720023229	0.03757728069530
C	1.63196187466449	1.10494255381178	0.72483376745340
C	2.07458953412106	1.74939092026878	-1.61977948838870
C	1.36333496790843	2.88879813325407	2.27215293236420
C	-0.52854922066038	3.67507925980066	0.91868511427723
C	1.13733899973941	5.26109698378184	1.60220256292153
C	3.93431456039350	3.68486289968805	1.21291131748538
C	4.14990028287386	2.58317427740853	-0.87143578071533
C	1.59896515896296	4.51328275457194	-2.22900115337779
C	0.12519708277951	5.30782775895911	-0.59868120362261
C	3.09637221978828	4.60399955329056	1.79220823508995
C	3.57337467896238	2.27545730871760	1.33860267016279
C	3.54912832988843	4.79334929378555	-0.96969938915794
C	3.16257735767220	2.67147803875993	-1.93645407742326
C	4.25552321019826	3.88647874830011	-0.21184452764933

Structure: C29N_c.xyz
30
Coordinates from ORCA-job 30N1.struttura6 E -1159.259703033472

C	0.13741651243870	2.53008822221424	1.66794600963920
C	-0.09547216234773	1.74376414305602	-0.47010316386981
C	0.22992422805821	4.85511240088839	0.80416214477588
C	1.40258686332461	5.11000891702720	1.58435971401928
N	-0.64508720258482	3.05219778511455	-0.42075297251620
C	-0.21064704136775	3.98636200544026	-1.31665954685675
C	1.36791089091187	2.43490837485246	-2.27809524549248
C	2.16123743023990	1.12279211034733	-0.54051859654804
C	2.37150280959721	1.78754743781204	1.79054892629663
C	1.39763167487974	5.63828978094914	-1.24132981355626
C	0.66362220299155	3.68043544423587	-2.37098187814512
C	2.95318955637274	4.14326895967544	-2.13227505728762
C	2.57304932600772	5.80579547733637	-0.48900076929532
C	0.29449626924005	1.39257691978320	0.81091343463129
C	3.71758969693969	4.79262231468442	1.21837892338345
C	3.25596277025941	1.99218248461244	-0.89955387668108
C	1.71412872819223	1.04355451278600	0.79067368738381
C	0.98654873404108	1.47718225215589	-1.37629812764080
C	1.34954790276511	2.76304664287703	2.37230878006252
C	-0.39198673144895	3.56737201890672	0.89979898953342
C	1.91065319168833	4.01769432775350	2.41387246765617
C	3.33921383494225	3.84664423308102	2.09026082669244
C	2.80279058169664	2.74080876715716	-2.06175094035930
C	1.66244350209696	4.71783613478928	-2.33386331125728
C	0.23422885238208	5.17604441915215	-0.59531516949760
C	2.55786041624855	5.64235803331850	0.88872684967300
C	3.51752885248931	2.50659208432360	1.49086213313795
C	3.60498588449064	4.83949253587542	-1.07243195598685
C	3.96272902025310	2.68131710044047	0.12462529371674
C	4.15225344928274	4.13568415781339	-0.03308774674238

Structure: C34N_a.xyz
35
Coordinates from ORCA-job 35N1.globalminimum E -1349.796274575088

C	2.36625963906632	1.61338363308481	1.15666307652032
C	0.12620555959780	2.61863757527558	1.36727546600483
C	1.41985562528741	2.32237882371003	1.97850647677882
C	-0.27553134533872	3.97973787874343	1.84023355942547
C	0.80796822745152	4.55226318933147	2.44779103350809
C	2.84748901081523	3.35252990188127	-2.28771686650259
C	0.51062296369039	2.22263282966044	-1.19947390810080
C	-0.31900612636477	2.51719271541104	0.01379169607305
C	1.73450876445987	1.57018588167344	-0.97427726331099
N	1.71237069049238	0.91482629671664	0.21466787833794
C	3.63191623563910	2.25468772909548	0.89457498893780
C	3.93479443643651	2.44558584207932	-0.47266444221163
C	-0.93719849258879	4.65177613163068	0.77757515480589
C	3.97728073041050	3.48307224760094	1.55007007814280
C	-0.44149185930334	5.83177932047106	0.26248380344178
C	1.60872399494384	3.98834262915885	-2.55705788935694
C	0.42522420879546	3.39950548449104	-2.03460097658265
C	2.93242499622603	2.21571194180346	-1.46047259108378
C	-0.29955532832363	5.62586818598220	-1.18824065023756
C	0.93531484088968	6.15005990089410	-1.57112645462815
C	3.06417170390620	4.10528599412182	2.37658933775224
C	1.43980705300734	5.74794463440500	1.89312856077338
C	4.46671992493281	3.81385552102249	-0.66889705014143
C	4.40028643225369	4.46557092353874	0.53891708634580
C	-0.58981298516820	4.26077904696338	-1.47862391221037
C	1.82868011302746	5.40986843537318	-2.32757766496454
C	2.82099673564326	5.49215156696668	1.86148235282958
C	1.82157321949537	3.51969979940775	2.63745229842502
C	3.16157965589744	5.57504317302790	-1.72307571867346
C	3.80430348638437	4.36453647893643	-1.79586755944121
C	1.64296161972377	6.65472492018827	-0.36176298361680
C	3.61812808410834	5.68220286296296	0.70278183140756
C	0.82729212896129	6.36566267655614	0.75113372320108
C	3.00386979441020	6.25650555763960	-0.44837764199773
C	-1.09188375006400	3.67281030053731	-0.29677482373994

Structure: C34N_b.xyz
35
Coordinates from ORCA-job 35N1.struttura2 E -1349.796426475944

C	1.85311772138139	1.30358584064203	1.18404906230755
C	-0.08546766022712	2.57813354183464	0.69148890100671
C	0.95646464301112	2.35344527874522	1.70699462493381
C	-0.33988968083286	4.84760958419464	1.46261166190456
C	0.77960171778712	4.69290012954214	2.28145715999931
C	2.22749592508135	3.60773199624413	-2.52785878393665
C	1.84429917914916	2.36809766937842	-2.10491553517541
C	2.57242180171962	1.66241898831703	-1.07204725193494
C	0.34225682884081	1.84589611342241	-0.47423992813823
N	3.08243024197716	1.39365884777879	1.73408369212259
C	3.82802679887163	2.11720130563770	0.92989776807795
C	3.69716382014789	2.27440067996190	-0.50755790923505
C	-0.65800249110524	3.82889246259124	0.49696474881722
C	4.04599071257170	3.43668972048228	1.47511282831534
C	-0.26989588017780	6.11496376015511	0.79730174489844
C	1.05103671794132	4.49161491228681	-2.57437592593830
C	0.42625201619425	2.45954195521430	-1.69444195579972
C	1.58729256970519	1.14020371993093	-0.16019673280312
C	-0.38083980228903	5.87000944119535	-0.61859059119113
C	0.72167977051376	6.47778827954822	-1.23427247094872
C	2.99134919405355	3.95407142353048	2.20195103427024
C	1.65360753679258	5.86557772392229	2.04392445151369

C	4.26717620888838	4.36879998941210	0.35796770416241
C	4.02349876215708	3.66282860200127	-0.84569591120969
C	-0.02806325878093	3.81310468051103	-1.90204608395855
C	1.46271639003274	5.74615050837685	-2.16361283441078
C	2.92341369680570	5.40269156981891	1.87704734926407
C	1.54928391973054	3.49007014348948	2.32771141724040
C	2.86381749968453	5.63663886022059	-1.67791008764644
C	3.32647659063929	4.32828526587699	-1.89507948359839
C	1.66434721255743	6.88883882783882	-0.14716817369605
C	3.63116409074281	5.60573174232424	0.63057478727266
C	1.02731120197844	6.70558477018580	1.07325603672206
C	2.95136891776121	6.29273431356190	-0.41667875283904
C	-0.67205294037792	4.47090738216889	-0.83717655445791

Structure: C34N_c.xyz
35
Coordinates from ORCA-job 35N1.struttura9 E -1349.775646927284

C	2.19540089752288	1.75433740804801	1.23380893535770
C	-0.03074651462024	2.57036069453658	1.55196382485680
C	1.30774633662850	2.49311061376800	2.04612586244017
C	-0.54371117815761	3.83345720478415	1.88887757846971
C	0.50472235819757	4.62167023134944	2.56595485452519
C	3.01018118335850	3.40236292643614	-2.63519917209266
C	1.31302409889655	2.00435698811241	-1.70747800413290
C	-0.24604169106311	2.31312251202316	0.14437170922453
C	0.76003870581118	1.54369979325044	-0.47588422710774
N	1.75468901842748	0.95628496130239	0.24614667576149
C	3.43055576497411	2.38438522590590	0.81997367921298
C	3.66473659097783	2.48400433666254	-0.64451498426305
C	-0.90175129709953	4.49661151574476	0.67956912609967
C	3.79662934748368	3.58900794173012	1.41057796747511
C	-0.27374198732567	5.73646865740394	0.67913816091868
C	1.79693562401822	3.93247677777623	-2.88709645833309
C	0.73778457371749	3.12395824075312	-2.31472798734753
C	2.81666501243738	2.26085311701760	-1.73229568128922
C	0.51716160000595	6.16960078590019	-0.43834313042335
C	0.51698350877264	5.37956959765384	-1.63295900025382
C	2.92254062359193	4.35756617240689	2.27388417041976
C	0.56236432708252	5.84758937049997	1.90777609446289
C	4.34497333916471	4.48511737592586	0.39768628702735
C	4.33175186614895	3.82885384201194	-0.84342358649084
C	-0.12605935570909	4.01293080882034	-1.59934370511508
C	1.70527661478527	5.29004688409648	-2.35548532652800
C	2.97011961278241	5.71209226353421	1.78178610790529
C	1.66938043666032	3.80544504543611	2.62223691612178
C	2.99320993938305	5.62723343875197	-1.78088622874254
C	3.80725752618934	4.44530539860904	-1.95345939564600
C	1.74725128989757	6.66003379443058	0.05587832751566
C	3.78267338090037	5.73758601702646	0.55125759513821
C	1.79058543852005	6.38886801402847	1.51470781043266
C	3.00013470592316	6.28805539094048	-0.52523923533653
C	-0.71187171477399	3.56037668895694	-0.42685554906064

Structure: C4N_a.xyz
5
Coordinates from ORCA-job 5N.struttura2 E -206.883747823423

C	-3.83121165319031	2.60992304219313	1.17134744636481
C	-0.86245572842590	0.72417917564871	-0.02492342633906
N	-2.88094949195755	2.00642332599836	0.78821343562798
C	0.18662319565085	0.05720488050265	-0.44678502689949

```
  C          -1.86057632207709      1.35827957565715      0.37705757124576


Structure: C4N_b.xyz
5
Coordinates from ORCA-job 5N.struttura3 E -206.811609566204
  C          -2.82605118278733      1.97170113135796      0.76682693897599
  C           0.16241623960552      0.07149772525989     -0.43699570962204
  N          -1.85015179208482      1.35064692702203      0.37274760080116
  C          -0.87370647066002      0.73033874288025     -0.02108653618080
  C          -3.86107679915342      2.63182547981236      1.18341770898322


Structure: C5N_a.xyz
6
Coordinates from ORCA-job 6N.globalminimum E -245.007977997289
  C           0.73036923315508      0.45958249795715      1.46363525739336
  C           1.85780744320188      0.27421251493198      0.88335928103077
  C           3.01945028814026      0.08482736406666      0.27557214019154
  C           4.09376188999667     -0.08905468542529     -0.28717640626704
  C           5.29094627681934     -0.28243718431732     -0.90685447367491
  N           6.30767486880104     -0.44713050721318     -1.42853579867372


Structure: C5N_b.xyz
6
Coordinates from ORCA-job 6N.struttura2 E -245.007961012781
  C           0.72806516472267      0.45255100334866      1.46149194505160
  C           1.85787321794963      0.27615426694537      0.88314579216945
  C           3.02206531466206      0.09182591838385      0.27825849853473
  C           4.09863217182378     -0.07882336277650     -0.28089246332390
  C           5.29133497773260     -0.28413250587799     -0.90579176946851
  N           6.30203915322353     -0.45757532002339     -1.43621200825514


Structure: C5N_c.xyz
6
Coordinates from ORCA-job 6N.struttura3 E -245.007984779542
  C           0.73307784770086      0.45997105272172      1.46901201924280
  C           1.85745007819513      0.27687341955940      0.88222127556357
  C           3.02012767576306      0.08685794936930      0.27609039924432
  C           4.09510133713662     -0.08895199402834     -0.28436474278450
  C           5.29052548073486     -0.28449602307704     -0.90746979604471
  N           6.30372758046946     -0.45025440454503     -1.43548915522148


Structure: C6N_a.xyz
7
Coordinates from ORCA-job 7N1.globalminimum E -283.080244251260
  C           3.38982342365134     -1.68000773323998      1.81301708017322
  N           3.01003581281802     -2.41972654856571      2.61338076586182
  C           3.84026596117397     -0.80600964659032      0.86806174932386
  C           4.24474370354952     -0.02279874899552      0.02258105023523
  C           5.10666898473769      1.63291044718513     -1.76203590079147
  C           4.68506201297800      0.82516712219889     -0.89244164916297
  C           5.54340009922254      2.47046510800752     -2.66256309563969


Structure: C6N_b.xyz
7
Coordinates from ORCA-job 7N1.struttura2 E -283.042666500026
  C           3.01591892548220     -2.40162475199982      2.59195774790130
```

```
  N          3.40769832259128     -1.64506828334128      1.77509143216969
  C          3.83738046435586     -0.81541842281038      0.87942175104386
  C          4.24238759552083     -0.03354760921640      0.03545235823085
  C          4.68163066542332      0.81407891115731     -0.87899348245684
  C          5.10089228677763      1.62291841101808     -1.75120212190525
  C          5.53409173984886      2.45866174519250     -2.65172768498362
```

```
Structure: C6N_c.xyz
7
Coordinates from ORCA-job 7N1.struttura6 E -282.984821573262
  C          4.98700467760561     -0.64771160045314      0.96908963948056
  N          3.80404119575620     -1.06558671375273      1.17431052598644
  C          2.82412567744235     -0.59540762841555      0.35547682120984
  C          3.02620870874054      0.24987950461668     -0.61581128636824
  C          5.59621789495814      1.12919548286374     -1.02775438622931
  C          5.32698136783270      0.22763447375420     -0.00435914796116
  C          4.25542047579553      0.70199647609502     -0.85095216611814
```

```
Structure: C7N_a.xyz
8
Coordinates from ORCA-job 8N.globalminimum E -321.165877623372
  C          7.32004932434333      3.47230633353199      7.35973257531769
  C         11.53514262588080      0.55702635734402      6.67054763227507
  C          9.43716342748382      2.01185234476492      6.99737400914065
  C          8.36274385290594      2.75306431942660      7.17509092533470
  C         13.64649500726300     -0.89332852212759      6.34876288252481
  C         12.52702678681566     -0.12466793979046      6.51872075900178
  N         14.58944438579497     -1.54040822012348      6.20681785927969
  C         10.43925458407116      1.31029532791142      6.83745336025741
```

```
Structure: C7N_b.xyz
8
Coordinates from ORCA-job 8N.struttura2 E -321.165884659718
  C          7.31059600266855      3.46189033034542      7.34770359677392
  C         11.53771975939552      0.56086307353647      6.67073141515146
  C          9.43509749089847      2.00713181774329      7.00596211772691
  C          8.35891715245315      2.74680769748424      7.17986530726563
  C         13.65023077264983     -0.88737947061673      6.34687291657378
  C         12.53113054529493     -0.11857468618905      6.51822262506800
  N         14.59313882533193     -1.53427055558544      6.20317148515880
  C         10.44377866753614      1.31565597959797      6.84328524742051
```

```
Structure: C7N_c.xyz
8
Coordinates from ORCA-job 8N.struttura3 E -321.154663474115
  C          7.35321974864375      3.46128120640459      7.33831069322479
  C         11.54070746878653      0.55387201153352      6.67310346334551
  C          9.44585399592935      2.00277363991654      7.01065248022219
  C          8.33430861122579      2.77548101891282      7.19333469132758
  C         13.64385277481706     -0.89917998453543      6.33857449292941
  C         12.52257137057750     -0.12631582430923      6.51483439951964
  N         14.58556051133669     -1.54420409230969      6.18878563594221
  C         10.42923949214808      1.32068174620723      6.85189861322886
```

```
Structure: C8N_a.xyz
9
Coordinates from ORCA-job 9N1.globalminimum E -359.237768222574
```

```
  C          0.55004347086078      0.00008573170840      0.00009184696164
  C          1.84836477417791      0.00003618218752      0.00002704017769
  C          3.11440397344811     -0.00001545095706     -0.00003655566743
  C          4.42546806665756     -0.00005896875848     -0.00007146906076
  C          5.65747032303158     -0.00008420963911     -0.00006443099255
  C          6.99907436638217     -0.00007151376201     -0.00003823354993
  C          8.21175216683889     -0.00002033366222     -0.00000443360770
  C          9.58046526141607      0.00004054032315      0.00003413888011
  N         10.73295759718690      0.00008802255981      0.00006209685893


Structure: C8N_b.xyz
9
Coordinates from ORCA-job 9N1.struttura2 E -359.173062253930
  C          5.07934885518317     -1.46484768837627      1.10461597560021
  C          4.17652077864553     -0.69244971120868      0.52010645600191
  C          3.76427159237149      0.26265886810547     -0.19770480635745
  C          4.40856922998915      1.18693828657461     -0.89583318808420
  C          5.64920832149436      1.38477369616706     -1.04241080502906
  C          6.94336410307489      1.16307164454245     -0.87709784648970
  C          7.33693950424551      0.21260925447575     -0.15839707275971
  C          6.30387706013716     -1.24761975535491      0.93936741689150
  N          7.45790055165485     -0.80513459492547      0.60735387551829


Structure: C8N_c.xyz
9
Coordinates from ORCA-job 9N1.struttura3 E -359.199671583151
  C          0.56348069549361     -0.00138828244001      0.00203458007077
  C          3.12759173290990     -0.00020681916068     -0.00109722104178
  C          1.86018005310862     -0.00093625927507     -0.00065793615879
  C          4.43824330335897     -0.00002431373776     -0.00156008336034
  C          5.67044130387259      0.00389163756740      0.00103333210643
  C          7.01260720430658      0.00084199023269     -0.00062292328327
  C          8.22281153436786      0.00348772074422      0.00124614602066
  C         10.70104347104768     -0.00486152832097     -0.00083714685237
  N          9.52360070891388     -0.00080414560982      0.00046125249869


Structure: C9N_a.xyz
10
Coordinates from ORCA-job 10N.globalminimum E -397.277795835177
  N          2.40165599371325     -0.00000155787397     -0.00000752165301
  C          1.65167263345863      1.05322646980181     -0.00000310006062
  C          0.67062682569317      1.81271790095288      0.00000756619191
  C         -0.65312385602980      1.83704433673081      0.00000380322970
  C         -1.71121335875452      1.16399278121932     -0.00000223799021
  C         -2.31763128967549      0.00000264644681     -0.00000624277338
  C         -1.71120337144358     -1.16398427744124     -0.00000125381524
  C         -0.65311064304971     -1.83702725122261      0.00000226324186
  C          0.67064163783236     -1.81273500757754      0.00000671260852
  C          1.65168542825569     -1.05323604103628      0.00000001102047


Structure: C9N_b.xyz
10
Coordinates from ORCA-job 10N.struttura3 E -397.321940450025
  N          3.03110847419198     -4.82160843748979      0.00143652923497
  C         -1.03225864309503      1.64276137531053     -0.00318353903719
  C         -3.06434349264328      4.89014474944413     -0.00282257102303
  C         -2.38798015273742      3.80457206188389      0.00374065285571
  C         -1.68918003884321      2.69108840468879      0.00199517303569
```

```
  C          -0.32362514750465      0.51429926473847     -0.00229445071697
  C           0.32294059664778     -0.52069364523980     -0.00114146671466
  C           1.04261507493957     -1.66442699922918      0.00042751406450
  C           1.68447618203020     -2.68822234054646      0.00125676764225
  C           2.41624713643050     -3.84791443356058      0.00058538536693
```

```
Structure: C9N_c.xyz
10
-21.7469230051
N  3.91800713687103963068 -2.11952185442222917899 0.00047491675825019606
C  4.96127701727597703041 -2.66739094726926273538 0.00049899145712429985
C  -2.81566660249675049243 1.43811407211539110662 0.01977078815052127694
C  -4.84555112395748022180 2.93799640050182775397 -0.03703307855582319008
C  -4.00905614999146742150 2.02471247747517413273 0.02223075449084079697
C  -1.69972516887397073759 0.90373831067535348449 -0.00012776709693068319
C  1.70931999444948323763 -0.94197249821747863763 -0.00233890101853081230
C  -0.54525524551137749540 0.26844427489268651854 -0.00041409627636483908
C  0.53882419081164190100 -0.32152779183875812574 -0.00043963008954313646
C  2.78782595671467925413 -1.52259244920447645022 -0.00262197781954324419
```

```
Structure: C9N_d.xyz
10
Coordinates from ORCA-job 10N.struttura8 E -397.287862382753
  N           3.33229767230121      3.30372901402894      0.00094460540651
  C           2.51549276620265      2.49395593304337      0.00068317668211
  C           1.53766235502241      1.52465273945636     -0.00007660552468
  C           0.68611522813637      0.68028092559797     -0.00053114806116
  C          -2.04421160505195     -2.02676368330843     -0.00003455793736
  C          -0.31572809544537     -0.31299603721314     -0.00035805826461
  C          -1.69369821373465     -0.23122753790949      0.00101991776631
  C          -2.81432135699166     -0.93380407724461      0.00157871169845
  C          -0.24574048807942     -1.69163456074758     -0.00153425509695
  C          -0.95786825706782     -2.80619272628693     -0.00169179196039
```

S2 C_n family: Energies (Hartrees) and xyz coordinates (Angstroms)

```
C5_a -190,18186
C5_b -190,08478
C5_c -190,07402
C5_d -190,11332
C5_e -190,04178
C6_a -228,21281
C6_b -228,18675
C6_c -228,10882
C6_d -228,13924
C6_e -228,14820
C7_a -266,32526
C7_b -266,24633
C7_c -266,22395
C7_d -266,22394
C7_e -266,26805
C8_a -304,39179
C8_b -304,28140
C8_c -304,27225
C8_d -304,30717
C8_e -304,31918
C9_a -342,47196
C9_b -342,37185
C9_c -342,39008
C9_d -342,36431
C9_e -342,38263
C10_a -380,62516
C10_b -380,55683
C10_c -380,52683
C15_a -571,00649
C15_b -570,90359
C15_c -570,91495
C15_d -570,95030
C15_e -570,94504
C20_a -761,49286
C20_b -761,36108
C20_c -761,38639
C20_d -761,33255
C20_e -761,39835
C25_a -951,88052
C25_b -951,79391
C25_c -951,81421
C25_d -951,83759
C25_e -951,82689
C30_a -1142,59141
C30_b -1142,48448
C30_c -1142,48878
C30_d -1142,48549
C30_e -1142,48560
C35_a -1333,06460
C35_b -1332,96369
C35_c -1332,96742
C35_d -1332,96777
C35_e -1332,96638

C -37,84496

Structure: C10_a.xyz
10
-20.9955106858 converged=true
```

```
C  2.07971135611587154557 -0.00000004990271703244 -0.00000003818217772562
C  1.68249080167995823665 1.22238107181220523323 0.00000003373703295306
C  0.64264809684858703598 1.97783621803158315977 -0.00000003317876415949
C  -0.64264846446884893449 1.97783656048117029158 0.00000005781790449158
C  -1.68249056345450220817 1.22238078627873836268 -0.00000005718736470182
C  -2.07971119396035319937 -0.00000003956092726074 0.00000000715892663249
C  -1.68249031621569744566 -1.22238072330648361863 0.00000001920752266977
C  -0.64264843915939673380 -1.97783662401215298843 0.00000000654173182773
C  0.64264801839196095123 -1.97783608063597848847 -0.00000002829005860766
C  1.68249070422241064904 -1.22238111908544722084 0.00000003237524661994


Structure: C10_b.xyz
10
Coordinates from ORCA-job 10b E -380.556833972568
  C          1.90761063015953      0.37639603860339      0.00011416423053
  C          2.30407265480240      1.67394254278619     -0.00008024961787
  C          0.94748345322780      1.69804965345505     -0.00001282111174
  C         -0.33369956963772      1.53982938538376      0.00002800354360
  C         -1.36135991188738      0.79332573783748     -0.00001621492498
  C         -1.90780358185947     -0.37621160766161      0.00005250390649
  C         -2.30387055117092     -1.67379527775924     -0.00002607715545
  C         -0.94736784502718     -1.69829068593905     -0.00006478364259
  C          0.33378775814645     -1.54008229985378      0.00007604413940
  C          1.36114696324649     -0.79316348685217     -0.00007056936739


Structure: C10_c.xyz
10
Coordinates from ORCA-job 10c E -380.526833300541
  C          2.02989616837089     -0.09259006069394      0.00009590798738
  C          0.49909380679559      1.79062437619924     -0.00000436048186
  C          1.60668171365940      1.08529646831054      0.00004012642952
  C         -0.65880486693186      2.43280661316239      0.00005416697333
  C         -1.01916265738855      1.08275787802502      0.00002444386980
  C         -2.13329892098637     -1.34536262004168     -0.00012428021170
  C         -1.48319733523017     -0.10786033615716      0.00019991870608
  C          1.55703947408165     -1.30634693199720     -0.00001286508551
  C          0.44735828456903     -1.88504144700236     -0.00011418352301
  C         -0.84560567223140     -1.65428393980485     -0.00015886408048


Structure: C15_a.xyz
15
-31.5565921094 converged=true
C  3.10168619167565706718 -0.01442378311652410885 -0.06211917410494969399
C  2.77456330004534779121 1.23880868090586204744 0.07910277537049073548
C  2.09153297852092201481 2.28632644254395467343 -0.05464977502437215473
C  0.93549445157663957406 2.91012086797627800294 0.03633242034832904338
C  -0.29688334569463203927 3.07742937407915428949 -0.03936731321546100421
C  -1.55199164893287644418 2.65403202435757590294 -0.00317982959627483106
C  -2.47894509662923612225 1.82897495752793282087 -0.01838500515647325390
C  -3.02544950243210708507 0.62027590885045080782 -0.03409237822233032733
C  -2.99954128676878317705 -0.62163310338117694354 0.01712501451052208384
C  -2.49104316190413754839 -1.83853470444868549727 -0.05272549373884647533
C  -1.52921302146930226940 -2.63255219091819814281 0.06112243206186740441
C  -0.30787870680865875306 -3.08647253972116297760 -0.06258090562846156668
C  0.93342608223841883053 -2.88442236797859097308 0.09531882290379391220
C  2.07773721772021380261 -2.30521956816185058514 -0.06443360224865518626
C  2.76650855458907773965 -1.23270999851501894540 0.10253201703259341715
```

```
Structure: C15_b.xyz
15
Coordinates from ORCA-job 15b
  C   2.44115852736320      0.40462872266091      0.08672001332644
  C   1.39943463600529      1.31952632065589      0.25160176060118
  C   -0.15869896430658      2.54509374329388     -1.05552317705432
  C   0.97018951007878      2.41064467870014     -0.58300887091684
  C   0.37797748889652      0.97771276951826      1.14478812968388
  C   -0.73591563557736      0.48773075209403      0.96094945137231
  C   -2.04768920822680      1.30896602320385     -0.58479650122419
  C   -1.50906721676276      2.31602095436351     -1.04456464828225
  C   -1.90140672203222      0.13171265856133      0.25429857510376
  C   -2.20739446571898     -1.17313852721750     -0.02306026744221
  C   -1.41960680281324     -2.17942088904206      0.20558839811392
  C   -0.41907981363659     -2.96380860526830      0.04873428886913
  C   0.85392599006168     -2.62663989922550      0.17873139372262
  C   1.99889172448218     -2.12023582407502      0.03928014637885
  C   2.35728395262165     -0.83879288351520      0.12026131303950
```

```
Structure: C15_c.xyz
15
Coordinates from ORCA-job 15c
  C   2.42915242337905      0.30745995326481     -0.02410964218431
  C   1.42827723724145      1.24186463547368     -0.41276428272861
  C   -0.24609843866429      2.79161528069271      0.79751161267303
  C   0.75553551503899      2.19888484788548      0.26811506594482
  C   0.38101270161023      0.63665929328669     -1.14395201097577
  C   -0.66085325432771     -0.00132643666788     -1.19285406111993
  C   -2.42833351627441      1.45534226435365      0.69457481952334
  C   -1.40161395192840      2.21445382397511      0.92705883640480
  C   -2.31731665532347      0.31670926337563      0.12301614553531
  C   -1.72091432989979     -0.68852031812910     -0.55875006751043
  C   -1.36953041227563     -2.02085848855897     -0.20178950703974
  C   -0.39276770080948     -2.71160033285460      0.02204164279139
  C   0.95688915798849     -2.74697608468563      0.24655874871335
  C   1.99511555467261     -2.10917458150427      0.29469441096735
  C   2.59144867000714     -0.88453311990731      0.16064829429716
```

```
Structure: C15_ch.xyz
15
-31.5565921094 converged=true
C  3.10168619167565706718 -0.01442378311652410885 -0.06211917410494969399
C  2.77456330004534779121 1.23880868090586204744 0.07910277537049073548
C  2.09153297852092201481 2.28632644254395467343 -0.05464977502437215473
C  0.93549445157663957406 2.91012086797627800294 0.03633242034832904338
C  -0.29688334569463203927 3.07742937407915428949 -0.03936731321546100421
C  -1.55199164893287644418 2.65403202435757590294 -0.00317982959627483106
C  -2.47894509662923612225 1.82897495752793282087 -0.01838500515647325390
C  -3.02544950243210708507 0.62027590885045080782 -0.03409237822233032733
C  -2.99954128676878317705 -0.62163310338117694354 0.01712501451052208384
C  -2.49104316190413754839 -1.83853470444868549727 -0.05272549373884647533
C  -1.52921302146930226940 -2.63255219091819814281 0.06112243206186740441
C  -0.30787870680865875306 -3.08647253972116297760 -0.06258090562846156668
C  0.93342608223841883053 -2.88442236797859097308 0.09531882290379391220
C  2.07773721772021380261 -2.30521956816185058514 -0.06443360224865518626
C  2.76650855458907773965 -1.23270999851501894540 0.10253201703259341715
```

```
Structure: C15_d.xyz
15
```

Coordinates from ORCA-job 15d
C 2.86474848005715 -0.39431664260181 0.01910192172479
C 2.60204206931808 0.92924704450291 0.01305969863728
C 2.07968916953958 2.04902235540819 0.00537019194128
C 0.91911424826776 2.68631951949009 -0.00468026215892
C -0.28350333413060 3.01273799381605 -0.01319422231460
C -1.44315344057264 2.39062762512981 -0.01827380702163
C -2.39223741745048 1.57103594194443 -0.02097178438926
C -2.72083918756738 0.31619708438558 -0.01840151207644
C -2.76544228769818 -0.96141508074151 -0.01324124298401
C -3.07560692300862 -2.41098170303701 -0.00878121294035
C -1.79714351607547 -2.00973957721059 -0.00228395372779
C -0.41048516262088 -2.05745597318717 0.00712882389889
C 0.78888792727050 -1.91354677045307 0.00946960957028
C 2.12353357100198 -1.53462099844079 0.01796304030041
C 3.51039880939575 -1.67311081900509 0.02773471683186

Structure: C15_e.xyz
15
Coordinates from ORCA-job 15e
C 3.02352144476909 -0.07365181463096 0.01526678122081
C 2.48194222961780 1.17761616975700 0.00814252450797
C 1.61886223000689 2.02456257702584 -0.00078641246734
C 0.39988256115870 2.69388164304364 -0.00821717753556
C -0.50029996101412 3.69494050101811 -0.01426423956520
C -0.94240126002257 2.28284154525977 -0.01382291651457
C -1.75568872143508 1.27631545685958 -0.01638632168354
C -2.46593180835708 0.19430712696455 -0.01376723819954
C -3.67300628537875 -0.66137910051209 -0.01409771449009
C -2.44297083504322 -1.20922613908733 -0.00745589436968
C -1.43018017483495 -2.16230934035364 0.00075016299582
C -0.35745304988438 -2.72057271447195 0.00868413404174
C 0.99981950626034 -2.85040752941677 0.01611038354596
C 2.11990499641073 -2.38404313804211 0.01982760226649
C 2.92400213347317 -1.28287523812185 0.02001632624671

Structure: C20_a.xyz
20
-42.1975919882 converged=true
C -0.77853629480111841143 0.21900999456384848130 0.34318406458157307837
C 1.13339935920984657969 1.19137165649101373077 -0.34858033676219501995
C 0.56923257851340169911 -0.04478836296033739811 0.11636976861734786570
C 2.88765625812431014907 -0.63737959190295467859 0.32148246851323447615
C 1.65199709326558474842 -0.88332982141056826111 0.34772130084860242150
C 4.69147281133224858252 1.32998580629367557648 0.22685716647157325165
C -1.39966824622104790699 1.31619191136241764362 0.31318500783290093015
C 0.43372822242043651553 2.42703644409773078294 -0.38220298746869152406
C 2.52507218713704961743 1.47409103553361675587 -0.37828481411813980051
C 2.68547922301430119774 2.88440634641931570314 -0.43024915303068755890
C 3.54513456312646813018 0.55975391386945028138 0.05294780215495678566
C 4.83363277647549782756 2.58224891151613666906 0.18011589139309211460
C -0.92702949173631099544 2.59750463387382213298 0.04443117901908075207
C 1.12421648656378181386 4.83506219971095863031 -0.06339456480249093528
C 1.39295714766054556932 3.47340663791033588126 -0.43265987284380436062
C 2.37041777525533570170 5.42885900845190594310 0.11520798162038534129
C 3.88827711719781321520 3.57558674587710090265 -0.05821193066949237327
C 3.51800054979643839204 4.90586709481795590904 0.11683039543837403507
C -1.09811098219663638709 3.96822226085530083139 0.21547076107325882632
C -0.24645414100891410714 4.89730332920192701351 0.17075586914143131567

```
Structure: C20_b.xyz
20
Coordinates from ORCA-job 20b
  C   0.44378475193820     -1.50529199762590      0.15413841721733
  C   -0.61944926446287     -0.66153800278579      0.05278400170426
  C   1.60309414764826     -1.85421686812323      0.23587950464381
  C   4.04638122270683     -1.21486916641933      0.33743617205004
  C   2.95740337473413     -1.75014743312447      0.30552254701119
  C   5.38719483835623      0.92970191475524      0.30207904308466
  C   -1.25901093216667      0.36440944065639     -0.03417042944206
  C   0.64572139952267      6.21541613038427     -0.23866122155244
  C   -1.50969344086235      1.70407598700778     -0.11654588919797
  C   -0.68435574783023      4.13191101347951     -0.19813275806492
  C   4.95684532064682     -0.20439531069337      0.33615192993819
  C   5.36607276596612      2.28896922549985      0.23196858732580
  C   -1.38762426639017      2.90372905964921     -0.17246188632447
  C   1.61130284771443      5.26234553443109     -0.12983424449990
  C   0.67558893360377      4.26415855369566     -0.12705358560646
  C   3.01091986391846      5.07050954604349     -0.04156590910260
  C   4.94201258610283      3.42154139164161      0.15027907405779
  C   4.03417236498783      4.43634305816044      0.04814146373851
  C   -0.84668027779038      5.48052490897087     -0.28277807920660
  C   -0.57280550050665      6.81223000294704     -0.34220075134738
```

```
Structure: C20_c.xyz
20
Coordinates from ORCA-job 20c
  C   -0.89601902917083      0.26811283286445     -0.34536408653053
  C   0.85142779818259      0.98272409660569      0.85671680059855
  C   0.38803992180338     -0.11632223427120      0.11002125868552
  C   2.84696737750579      0.00783465292194      0.08914346053819
  C   1.62358646439789     -0.40908085221403     -0.54356114880412
  C   4.58796319631944      1.60616964641431     -0.31092335165004
  C   -1.51437009173743      1.33183360132725     -0.29299472080058
  C   0.34400874452591      2.29964155321016      0.72956818198519
  C   2.29915782611432      1.04932745256176      0.85466984594157
  C   2.68484548126016      2.40395019953855      0.71746686598217
  C   4.09810699457357      0.47557594027472     -0.36089340122920
  C   3.87267711791248      2.77617190701772      0.06902133660263
  C   -0.88799779253531      2.56175594193855      0.05582973654232
  C   1.40564212143579      4.46685941842224      0.08444785890897
  C   1.46230299985829      3.19458864850644      0.67885940707559
  C   2.70973088746443      4.91943907458809     -0.28781078897079
  C   3.82339773348768      4.15639188632188     -0.30274007937275
  C   3.92712265869380      5.51051894397030     -0.73217096195765
  C   -0.89441398750213      3.92019150553367     -0.30009744053752
  C   0.06869856524743      4.68972277301789     -0.29821278128899
```

```
Structure: C20_d.xyz
20
Coordinates from ORCA-job 20d
  C   -2.10508039228060      2.77737940206771      0.29547548510677
  C   -1.24436467837526      5.14855024634608      0.32320065649768
  C   -1.08631848885098      0.47863019239849      0.08627487050532
  C   4.47496544333538     -0.45612425352958      0.60109113921842
  C   2.19387101663020     -1.23125202916180      0.36926632800641
  C   -1.85407029173724      1.58999287173327      0.21861708071204
  C   -0.11039388954953     -0.23495163560000     -0.02698866680710
  C   3.44241378921714     -1.21141507277105      0.66513235040047
  C   -1.88393899407288      4.11377176933121      0.33854863730652
```

```
  C   -0.14999272584307      5.94425241789278      0.25209872670794
  C   4.85187455438616      0.70419545709738      0.20326323608842
  C   4.35211563363976      1.78920204099743     -0.39177002911524
  C   2.05361940355882      0.62953634251293     -0.82486861515630
  C   4.20743451318356      4.32845484903549     -0.25709448827201
  C   1.02730400670011      6.23295760497348      0.14886342572959
  C   3.40119032553722      5.41189790995578     -0.12280637505437
  C   4.57616992916731      3.17386193757776     -0.32980127741228
  C   2.35863875431454      6.02482564169123      0.00440858864266
  C   1.26565613286349     -0.45698506709195     -0.19580412580908
  C   3.02978094601308      1.33862635780197     -0.88613095557731
```

```
Structure: C20_e.xyz
20
Coordinates from ORCA-job 20e
  C   -0.66864418562556      0.19429314225467      0.15724877349705
  C   1.39873240351293      1.15062395936597      0.07262730373814
  C   0.69764735172581     -0.09250249467561      0.13367705421691
  C   2.63545142606916     -1.46860478471060      0.13267570877493
  C   1.42208173140555     -1.33197836604490      0.15904106668609
  C   4.05281495805044      3.77249526774791     -0.09140356498466
  C   -1.29845506433529      1.25118717714447      0.13606692563429
  C   0.70938912001203      2.39010906514502      0.04618675427148
  C   2.85482964468177      1.51082553417069      0.02257611428656
  C   2.97793078378942      2.86503413992002     -0.02930039953392
  C   3.74773891348987      0.42244374845995      0.03839642418713
  C   3.83289661310169     -0.79809217723432      0.07757697854906
  C   -0.73205771370554      2.52178941730404      0.07669994172706
  C   1.14330585713146      4.83328339254470     -0.05384353262702
  C   1.61040322875141      3.48364734122116     -0.01609491653846
  C   2.08014759855775      5.91866818936093     -0.11851509118035
  C   4.35124660671860      4.95798340095848     -0.14371656146027
  C   3.29813376779848      5.83998696151536     -0.14700146421508
  C   -1.06078035577840      3.87425182772229      0.03615233182020
  C   -0.25193769222265      4.79996225167184     -0.01807384454704
```

```
Structure: C25_a.xyz
25
-52.8482374542 converged=true
C  1.68431643119350638038 2.33123755303655011417 -2.31887985127795470675
C  0.44201781783194105113 2.81690183879016942115 -1.82642720296664551327
C  0.49490208262061013977 0.84611677230910398162 -0.26445074953827574227
C  2.38539155693664328695 1.30568965534236247628 -1.65835402707625911845
C  3.81823859584632296915 2.11094268973724918936 -0.01007767172260583786
C  2.72091110339934694196 5.03645440726563986544 -0.26793885492038482798
C  0.76818404073638402174 4.17070407103744145161 -1.34204977179924678232
C  0.86245061943814993466 1.90306414974686255448 1.89786495007508615451
C  2.28930350194856258028 1.80988223270316606062 1.70795474027441440690
C  3.52274558514073765636 2.22702617230794519543 -1.36138680156542668520
C  2.80512044615926159352 1.19106017568167699139 0.57461835853261788998
C  -0.01804028088556875950 1.28893611181537881549 0.99598560923351309171
C  -0.77813587825111607543 2.40076064314208625916 0.28063493601855887816
C  -0.31011753171183381594 3.61870912292346513794 0.79410470775108366048
C  3.85808622406681145378 4.48828036485247050535 0.22481801145921304075
C  2.21976282934476065734 4.44880752946962587657 -1.51392028762576691392
C  -0.25258791830678506418 1.93961492653057065993 -0.96818396025883779465
C  3.91484588651395348791 3.29027935336388566157 0.85582790000914976680
C  2.78752272138856804773 3.30286107288625840894 -2.05402632963306031044
C  0.56895577554001797171 3.32350108773907626514 1.90957476963614158905
C  2.89171838091042365804 3.14985861889403739866 1.85373794859215879072
```

```
C  1.63754534476552771061 4.98466594059034484587 0.67348853730585078736
C  0.42872968914528919449 4.53186264400941674069 -0.04287864426383356659
C  1.88341242440735889829 0.70833115902457743918 -0.47000768470010245714
C  1.78207255457408364485 4.07551671372339630040 1.78936236609679721887
```

```
Structure: C25_b.xyz
25
Coordinates from ORCA-job 25b
  C   1.15513084908997      2.82973866559902      1.89348178379641
  C   0.49039632316346      4.96486902016238      1.06057098857714
  C   2.69664054535253      0.82700530226759     -0.94230126960119
  C   -0.53171937804022      1.92920817445013     -0.69326628384420
  C   2.90701685706556      4.38634701169219      1.27702887210503
  C   1.02776130789688      0.82420703717686      0.79141594940784
  C   -0.38919781853600      3.42916782840093     -0.76250155076283
  C   4.01305435810634      3.68495426597915     -0.23367670142434
  C   -0.22020015502775      2.68546116116730      1.41285759345720
  C   1.43566709993665      4.89476359162221     -1.71341968364794
  C   3.25979703837972      1.91876573723239      1.18182048593039
  C   -0.29574274327131      1.54690823984897      0.69523392411534
  C   0.74517609942096      3.71452484449160     -1.68176033991770
  C   -0.33396892527795      3.91759052587770      0.52519763533503
  C   3.34548617426998      4.98805586466135     -0.05159869331358
  C   3.62064371349021      1.44741356070091     -0.11538695848463
  C   1.38982030506977      0.52808684401044     -0.50330614104355
  C   1.55114502797669      4.18064478967626      1.67204769361961
  C   3.74367509158662      3.28118189002655      1.16385335363925
  C   1.93347850130037      1.65478652865227      1.61548562859784
  C   2.69389739013679      2.04998635825863     -1.75251550908871
  C   1.35607834528968      2.42291630822938     -1.99469309770002
  C   2.38965562757550      5.21832688897027     -0.96649672580563
  C   0.50354356244108      1.39253194325637     -1.37368968940053
  C   3.92011681594095      2.58362263509536     -1.04499026161829
```

```
Structure: C25_c.xyz
25
Coordinates from ORCA-job 25c
  C   0.87369659254562      3.57320530944534     -2.07621666210779
  C   -0.15896940293696      2.99917946106346     -1.21998998789285
  C   1.79995461471238      2.50878848918388     -2.45410896945596
  C   2.47840088012808      0.76752854918146     -0.87471007440101
  C   0.20207880029701      1.71273820269370     -0.91904380582728
  C   3.02659686548566      3.06636127661797     -2.84219522277810
  C   -0.30931231796798      3.72039422848852      0.02359802018031
  C   1.11024178231326      1.58677702955372      2.48272162207915
  C   3.13493687529313      1.70555746183391      1.24837100341877
  C   4.16574758510533      3.85014210820663      0.63194004687918
  C   2.28375598335776      0.80318202647232      0.52004131923606
  C   0.97956262772645      0.81809187600557      1.27214800641350
  C   -0.37969212605432      2.73903342937797      1.08024796394553
  C   -0.00491309885590      1.44753716611877      0.48732420694287
  C   0.41288764065366      2.87039172791931      2.19412827971217
  C   1.83183135426001      4.52898628567245      0.97909847165432
  C   1.47053283549802      1.35517470481023     -1.60126886063340
  C   4.04411267207733      2.62286756707422      0.74633928505054
  C   2.28305835721168      2.31561207678986      2.24451248541154
  C   1.64635371860284      3.63763082784159      2.06320589441779
  C   2.82754092057288      4.82173914768172     -1.21116915738580
  C   3.08041726708577      4.59442642056030      0.17873116329644
  C   3.44222847614562      4.02430751059536     -2.17790905400345
```

```
  C   0.75800059414843      4.61163600091953      0.01645691338188
  C   1.40830250534716      4.61977613869023     -1.33286188989822


Structure: C25_d.xyz
25
Coordinates from ORCA-job 25d
  C   -1.13025874987470      1.94305689022569     -2.11537018499339
  C   0.26639664441644      0.91608323342910     -3.46678810364916
  C   1.35126726025515      0.59137272972404     -2.95958014938696
  C   2.96406605394610      1.03144288278875     -0.91468656263978
  C   1.67002971187053      0.82639738047324     -1.61340847822122
  C   4.93747578702471      2.19443344258701     -0.55114856979646
  C   -0.92152511715682      1.50445145775524     -3.25792067407815
  C   -0.58213237459484      2.10258657895009     -0.86145000624440
  C   0.64830262474767      1.26895529493604     -0.71150604424343
  C   2.63963108315431      1.55333108380730      0.39913463930966
  C   4.24660435433514      1.39378305596913     -1.24075380604360
  C   4.66442153285961      2.97930569180794      0.53116443311982
  C   -0.59038279381115      3.02480153965865      0.20766286770909
  C   0.61205006567583      2.77682327199569      0.99185566980419
  C   1.23834935476744      1.64995436618648      0.49782479943096
  C   2.76161061493450      3.61937998092084      1.65086958780720
  C   3.40275351342522      2.48453375296028      1.04077901547872
  C   4.96599861603767      4.35658430431426      1.01129423670915
  C   -1.03995793234814      4.31427186622394      0.52613981675584
  C   0.82752156372633      5.17576640844754      1.72733685942078
  C   1.37053963490118      3.80483428838961      1.63854998295564
  C   1.81516937281590      6.09544463124187      2.05017604290235
  C   3.74866391772167      4.68965469661104      1.67962587615412
  C   3.02610564652805      5.84150671020713      1.97286092152369
  C   -0.48534838789668      5.16230946202000      1.22672883314332


Structure: C25_e.xyz
25
Coordinates from ORCA-job 25e
  C   1.03987788697838      0.57805778919108     -2.57152930533769
  C   -0.21692668437747      1.15713233754988     -2.61866217829398
  C   1.82498693135624     -0.56060511654454     -2.83261621660625
  C   3.15322608156792      0.80873414489151     -1.47833342817701
  C   2.86275381234613     -0.39131343967498     -2.13433324871689
  C   4.78570876754693      1.63000026461717      0.55628654170892
  C   -0.84912718964517      2.09176560553550     -2.12991746555009
  C   -0.79475894046371      3.13616145963862     -1.21427484702739
  C   1.91634460560496      1.46371611145134     -1.77335294137579
  C   2.85694145703337      2.59989985199368     -0.07710648645933
  C   3.75371615439100      1.54439337539814     -0.37531193546112
  C   4.94657260235960      2.40116876990508      1.51032348538431
  C   -1.71213862261340      4.00887036746710     -0.60136232915099
  C   0.53113088854326      3.32295132810631     -0.64256912127757
  C   1.66845407249321      2.52542864343812     -0.91786128625425
  C   1.92241789964917      4.38250426117050      1.17412029786189
  C   2.99235889642339      3.49172209251794      0.98972474095098
  C   4.09316222776899      3.42456396120183      1.92856318346376
  C   -1.48809085310131      4.69681091388711      0.39754610458171
  C   0.28516236240370      5.18113001464363      2.38762234277900
  C   0.71079585989680      4.26477726115092      0.39768508063653
  C   1.70946271769349      5.18333859472691      2.32077387850661
  C   3.86849021749395      4.36574440911177      2.95744150113324
  C   2.84125444427975      5.01781211636289      3.14340685716002
```

```
  C   -0.29442359287625      4.97629988918517      1.06312776257402


Structure: C30_a.xyz
30
-63.7147481964 converged=true
C  -0.04352568544567140402 1.74780678938890710050 0.58449200764456599178
C  0.98869780494706960283 1.93455579650646503076 -1.67436664130934098615
C  -0.53342407815088022094 3.10284149480665538690 0.84885830495656988681
C  1.74568998114400986132 2.12453825368492266179 2.18495486154630347286
C  3.31601295832857134727 2.17941960853338567716 -1.41794695116171443061
C  2.15168929437513956060 1.30124190803914974168 -1.11167207217512209461
C  1.39692261259209660196 3.10640928956941886341 -2.40262595828774472295
C  1.14193112945905594913 1.29321953394152977523 1.14828715255153368524
C  -0.12761933561664609571 2.06178834989978909675 -0.84384982021075083392
C  3.17309162944964029407 2.20441180646274714761 1.90268385951915264265
C  4.02817454834761168314 2.41181996340271265211 -0.23525668560021009701
C  2.21431109345261667443 0.92244004350420405469 0.23022531586576874818
C  0.78272247480337919789 4.34612481015025586828 -2.21843609955231668707
C  1.86174512382654167020 5.29258709996004039766 -1.96646662622527546027
C  0.19001343196332237673 3.98341002182545400601 1.65715793647091325091
C  2.46693374965514866659 4.35759404653488235937 2.12374437132936977335
C  3.44955646558722284212 1.49522538052389886154 0.74946053123423239661
C  4.07727102720666767510 3.74910999929937593933 0.34161050204664544783
C  0.56295343787326779239 5.24567293070058227045 1.07589187988131862461
C  -0.20907340300897589436 4.52415106931636401555 -1.16252385962626791915
C  1.31776733454979466664 3.44054897944622561923 2.36687545213119765464
C  3.56470943935855677509 3.60055357335498804616 1.69767845720573440360
C  3.12411619835766352082 4.57371016613233916104 -1.78136327199382882291
C  2.86395626403514302893 3.26062297337323636626 -2.19118505508680661009
C  -0.72322432436470618367 3.37347932358940560960 -0.57819914366145686024
C  0.31407926620443349375 5.54826133452210790153 -0.26373978956297328136
C  2.01482925032314907909 5.43879475838520054509 1.35052538902440777413
C  1.58521709067211302724 6.00181135737305115896 -0.81327831397835392835
C  2.66075058433603883756 5.76263786666924548996 0.15144484635080202040
C  3.62156162646730894750 4.83875947550898111160 -0.43755958618783608038


Structure: C30_b.xyz
30
Coordinates from ORCA-job 30b
  C   0.62729857190828      1.21897332014492     -0.52996984052328
  C   1.01900548396018      3.84808935531000     -2.54532529004771
  C   -0.44882672821597      3.00149699785619      0.54033652015449
  C   2.95108452534735      2.63144159311036      2.60754186423952
  C   2.53562959316953      2.05640286284741     -1.63595237000320
  C   0.14253574100057      1.69780564767688      0.77325149093306
  C   1.29298198671790      2.50755933767265     -2.29500422843610
  C   1.18399853583641      1.52461789133094      1.65307903722685
  C   0.17017846097127      1.98701643564504     -1.53123085984719
  C   2.09876854647957      1.23108219419039     -0.50266095825287
  C   4.07128788019714      3.23382033727285      0.99555769584810
  C   2.45911130638491      1.21677681998606      0.84965332179741
  C   1.66599713375771      5.74520722669463     -1.39525690920612
  C   2.05103637546885      4.79804383636161     -2.41979976023297
  C   1.05341297783727      3.86019893498603      2.29487612120454
  C   2.23593157727442      4.73326356082467      1.99283158960799
  C   3.47586033519678      1.96787086447128      1.48756610524689
  C   4.12138597187453      3.65202352733999     -0.28545334832208
  C   -0.53155336077571      3.14445472098098     -0.92128619256098
  C   0.39998056257255      5.37268732126982     -0.86254011783284
  C   1.52282594272448      2.56217888316209      2.56692685040769
```

```
  C   1.87804150616772      5.55025804776503      0.89818950587011
  C   3.34181843065699      4.35428908296064     -2.10468990358771
  C   3.53205971531782      3.05333540289224     -1.53999548782838
  C   0.51989220443990      5.27993700826723      0.54500326535386
  C   0.07473318841303      4.09968183286572      1.28086650304462
  C   2.63109478827257      5.79695351029104     -0.33039393746780
  C   -0.10397648540823      4.25884799929128     -1.59582588928724
  C   3.37194218595496      3.91698411097464      2.10625563642455
  C   3.63430004251767      4.92224936642172     -0.78112943136822
```

```
Structure: C30_c.xyz
30
Coordinates from ORCA-job 30c
  C   0.00130720159300      0.80854018164864     -0.10237272855090
  C   1.14423103909120      1.97339507851160     -1.82744591007910
  C   -0.52716284147765      1.97002095111664      0.59646285049080
  C   2.81390949477967      2.19738855926223      1.72504235415863
  C   2.13236983538365      1.41286444452132     -0.90040326570233
  C   3.35027070676284      2.12331369799341     -0.67695516819793
  C   1.30649228069754      3.29996057003206     -2.30658316093075
  C   0.36513923232380      2.48961166355248      1.54430037207158
  C   -0.11184297350883      1.66983062321179     -1.24624382018466
  C   1.69810694399322      4.36248194622081      2.30866668397709
  C   3.58669218857166      3.34087294259196     -1.45477689779231
  C   1.38306270928438      0.84820635834504      0.21406624106170
  C   0.38310157034211      4.30850937538156     -1.87141383776608
  C   4.12345647646418      4.38773929732091     -0.50099372240453
  C   0.55593912072985      3.93163059531057      1.62088523124921
  C   3.79364423515931      4.38593419655784      1.92042469111485
  C   1.55932727495536      1.64612247776942      1.40771643550640
  C   2.79226852574550      3.43559172635631      2.38659140352771
  C   2.40095521868808      5.87038305214519      0.48662978486001
  C   -0.43991747351007      4.01878183407121     -0.72964524431323
  C   3.75051733227994      2.40128586412268      0.64488031373198
  C   1.06929754608899      5.66755821183542     -0.03720780375800
  C   3.29762470518504      5.49799105114514     -0.63266170468847
  C   2.64531534146298      3.89158013150828     -2.27284584615436
  C   -0.88075357843379      2.72350445582752     -0.58451027948310
  C   0.15096471316713      4.72847871617472      0.44651333542415
  C   4.27979075522036      3.77978609776879      0.76354894065946
  C   2.49754952779562      5.30425728590896     -1.81865488107518
  C   2.67199247833947      5.30118237048639      1.70996535178192
  C   1.18418740884593      5.44674424770661     -1.49755871481248
```

```
Structure: C30_d.xyz
30
Coordinates from ORCA-job 30d
  C   0.04251459363964      2.19469770856432     -0.71325197921119
  C   0.62423901234990      1.13289970195290      0.12674421191641
  C   -0.25971069940555      4.60981660351174     -0.44644379106193
  C   3.20049304024723      4.05386017533859      1.37834879426388
  C   3.43779853138379      2.19531578441935     -1.46407733380699
  C   3.66320582917548      3.49969964040951     -1.89252577449248
  C   0.98843113901999      2.49289298743389     -1.78553099779982
  C   2.88635984736970      1.26827286490444      0.63349162793490
  C   -0.64936872255385      3.26870671581919     -0.06388757402843
  C   0.95967930311366      3.53704861896906      2.46096514168897
  C   2.15319835719563      1.63735223434415     -1.52400387088684
  C   1.89502838403791      0.84330847513432     -0.36153101395908
  C   1.24439329578462      3.81851138548665     -2.22603846537016
```

```
  C   2.71461792367585      5.55541316084131     -1.61668421442726
  C   -0.70962952580671      3.28651732155244      1.35423679001050
  C   0.07337388315533      4.45185127109407      1.83512781471487
  C   3.82932563597872      2.09308954391784     -0.01188931016646
  C   3.67568195828524      5.40357876368764     -0.52529195100314
  C   0.34940642674928      5.28308741748050      0.77084983746638
  C   2.21302062310126      1.77664936170930      1.73889549599260
  C   2.30031003887176      3.19424891309471      2.12474024282933
  C   4.12011423737787      3.36580208513743      0.43879089357639
  C   2.60544742209438      4.28349383706109     -2.37888561498724
  C   0.79160498877445      1.44701931258347      1.53405668165164
  C   0.52675598602545      4.89424312461448     -1.52386992309740
  C   1.57934825392898      5.81360305621308      0.38338378693195
  C   0.03724649843395      2.39924231089951      2.15154303730314
  C   1.51771802988435      5.88735764198538     -1.10057712495599
  C   2.88781844936817      5.32174004514815      0.74393728651605
  C   4.27941424418044      4.21422794109697     -0.72520170511233
```

```
Structure: C30_e.xyz
30
Coordinates from ORCA-job 30e
  C   0.86080707458463      0.83774686817503     -0.04953614905326
  C   0.74322007650565      1.68676679340661     -2.12270770418401
  C   0.14987668161447      2.75028679135124      1.15627891480385
  C   1.21495174569397      6.21760793616116      1.80497974212254
  C   2.09950148563786      1.03692211383661     -2.00904958348086
  C   2.65552752699223      2.33209087451200     -2.33652357693367
  C   1.28583851989418      2.89343772373479     -2.54951597886526
  C   1.01044573027486      1.54785326143864      1.17660902883698
  C   0.04730618650569      1.61540218186337     -0.89277233893010
  C   2.36932535262822      1.74850240713200      1.38110267516633
  C   3.81503927692651      2.35248057737303     -0.24192224299987
  C   2.18479726195361      0.63045288567370     -0.69252868841376
  C   0.93792666344780      4.06760471595656     -1.87057230214709
  C   1.97332366418685      5.01408152350331     -1.46746504998002
  C   0.56877290419326      3.91121764652142      1.85477789744496
  C   1.95071189717300      3.98782122660346      2.29552829563992
  C   3.12712811078551      1.23496387724718      0.18806834709228
  C   3.74274152066717      4.70126945759049      0.23042903519154
  C   0.24458566166820      5.24375215100375      1.36472628268710
  C   -0.08645078249436      4.09821490448228     -0.85034412535586
  C   2.32307156251851      5.34105358257879      2.12410730992512
  C   2.87313028947352      3.00763683897301      1.82670196008720
  C   3.21663682139918      4.42333433073254     -1.11873324489530
  C   3.51386422926684      3.00907655787881     -1.49377417647810
  C   -0.32026144614308      2.84325643387166     -0.19092615158503
  C   0.14287917408131      5.25958583214923     -0.06123554965055
  C   3.19487342721592      5.71635908262285      1.02498343532970
  C   1.35110872136884      5.91426264745076     -0.54525460586428
  C   2.00803235966375      6.39823744481938      0.58007102693135
  C   3.77912529833634      3.40226934105358      0.79991851598845
```

```
Structure: C35_a.xyz
35
-74.3980549248 converged=true
C  2.56918307409910529060 1.49978688756896882950 -0.63580338603950758980
C  -0.22753992711563655882 3.22386143135367175816 -1.56455831380345578019
C  1.25859669438638621664 1.49160722046977323174 -1.11672109160965904451
C  3.12528198818842861684 3.36811996636790444981 2.22491599775205584066
C  2.50801015294248186294 1.46659009326072675705 0.83764422230562918248
```

```
C  3.46248190046013348820 2.37087515774209478536 1.30467366147168983836
C  -0.50620920761478027661 2.73287131451918385849 -0.27048389527051525105
C  0.36193346902704942591 1.63348056609294034480 0.01300449742360534205
C  1.80526843568356842873 3.34966561307057641983 -2.61794174858535289019
C  0.87518778154413412462 2.45317661373022266602 -2.10907221611479300805
C  1.76755775184617136553 3.58926622667301709058 2.58331458896326582320
C  4.23730797314289731048 4.40347975091792775970 0.40401582201977320752
C  1.15214373558022709076 1.64009376080818691790 1.21920489441337664971
C  0.78846046431188343551 2.63007096456749378532 2.18498127354277160705
C  0.00662117069709035160 4.61869214643410952448 -1.80633081207967838289
C  2.21951749710040280306 5.58705995360668516980 -2.24910929718245000686
C  1.20516040401619717315 4.69549595382946893807 -2.60257086982881924442
C  3.41625342645269070019 4.80980907739230012510 -1.86221484649642987641
C  -0.76193663839125813642 3.64853987265147328145 0.81622041871674511082
C  0.80033339935682146216 6.57707124453618519055 -0.57787682664327932880
C  1.50114563039081261309 5.00619874987935364885 2.35598361138766776435
C  2.78145611926941338510 5.62979104401832408655 1.97895253635021584060
C  3.10912045641647694083 6.48883664897861933696 -0.30121708131641788952
C  3.51438404834825890433 2.50604868186735130209 -1.04588091639685565326
C  -0.44853445984254530776 4.99624792953948571039 0.61594097230786437613
C  2.01386619379480658054 6.59519951013912830717 -1.29939599964151142331
C  -0.49076145069158538803 3.21149304292817028283 2.12911317696692581336
C  3.90167124339551163459 5.33690795142438467735 -0.63057588102285500042
C  3.13902618099329488999 3.43400908991762499056 -2.06296522984989305627
C  4.11381375374760072106 3.02134045751395996504 0.14065453062764857184
C  -0.14233271351485676792 5.51135093165964118356 -0.72002606805262792111
C  1.12430919970123444074 6.68980735249296731837 0.82255217951105852325
C  0.49708523941337112717 5.62621842043934528022 1.55416334323126759109
C  2.54370619379317242092 6.62867628205796499685 0.97630480711521439030
C  3.69526581628290573533 4.62506910732273279763 1.72966394021059666208


Structure: C35_b.xyz
35
Coordinates from ORCA-job 35b
  C   0.50869011158708      1.49628223481776      0.89744556532890
  C   3.36941073810575      2.59802806554068     -0.56238747281049
  C   2.79020663966748      1.38998463569360      1.35760556775050
  C   3.60519090387836      2.38641760692616      0.85297177563833
  C   1.75995168656114      1.17650482922991      0.33216387044111
  C   3.98503069755971      4.97874707268299     -0.32887501357039
  C   -0.27755326279194      2.45374226187403      0.23168802253775
  C   1.59035750867735      3.50674099455782     -2.56003082866488
  C   1.22030594176688      2.37377452115908     -1.72181183911341
  C   2.17867228909472      1.80211638329482     -0.89074429776855
  C   3.61167491649503      3.58047400525716      1.73175777509768
  C   0.35733972129020      3.41267817325566      2.24348445394104
  C   -0.10236352550198      2.62068642403769     -1.15959762873177
  C   -0.37580196514597      3.68214687611051      1.08415292217125
  C   0.48569112359846      4.39784500208192     -2.66915817135630
  C   2.84906002882387      5.42703334890593     -2.93727089024251
  C   2.87922337855550      4.11019103525521     -2.42699152438568
  C   3.58212287816277      3.83716554185389     -1.16361251217462
  C   -0.57427294607188      5.03593996176235     -0.93114523982272
  C   1.36772845588505      6.50218413949565     -1.42881595598710
  C   -0.29540920774464      4.99150441185497      0.46275364430497
  C   1.60665522987022      5.49080151723853      1.99375549825810
  C   1.46793221661007      4.27298138645983      2.64553328195029
  C   0.77370412748270      2.02323977668094      2.25741268386270
  C   0.67800798819275      5.90676491636576      0.96290436586559
  C   1.45692361648213      6.68836390609464      0.02390005933161
  C   2.61150719061632      3.39064191050505      2.68546107668225
```

```
  C   -0.47883206446952      3.85129080972090     -1.68860720479988
  C   0.39550858570255      5.63437678081623     -1.85442426591714
  C   2.73974761020968      6.27776687214971     -1.89398928650146
  C   3.77051527640475      4.84550538065374      1.12722201867592
  C   2.93023146475113      5.90032752763675      1.52372593957948
  C   2.11356602601349      2.00407467289020      2.47537604466233
  C   2.79347178361885      6.79683185583943      0.43163581133234
  C   3.54263982798195      6.25365416119706     -0.68492823530666
```

```
Structure: C35_c.xyz
35
Coordinates from ORCA-job 35c
  C   5.26569907764182      3.41114846526056      0.30640033305569
  C   1.00862704953344      1.08793055100543     -1.18952100822766
  C   2.25691299821077      1.36271500806703     -0.59214050295900
  C   2.68665153258855      3.25718882415365      2.37539256326801
  C   2.06902441489087      1.57457768809684      0.84916258745990
  C   3.06844057233805      2.42966508787599      1.36653886637938
  C   -0.76299291671602      3.68146455044651     -0.45075339345881
  C   -0.60626549149822      2.43090603920041     -0.95671134848926
  C   1.18773592810755      3.22282404319495     -2.40552048001492
  C   0.43868741736820      2.18102182272527     -1.99362165327321
  C   1.27933878892891      3.56107226878971      2.59070139559473
  C   4.21883081170938      4.46310970590237      0.22927053054989
  C   0.69539802793444      1.78970189066873      1.08316752560177
  C   0.29714329638053      3.01455163674229      1.75183337578674
  C   -0.30244014699046      4.83324772692170     -1.23084068177080
  C   1.82618937746569      5.45945664581653     -2.24624822500360
  C   0.69693871679783      4.60311843031675     -2.21260362478289
  C   3.01541913638066      4.60302843195018     -2.05933085187718
  C   -0.49960160049088      3.96677815994316      0.98279433057273
  C   0.99003073799741      6.73549192444796     -0.36885568782879
  C   1.57974549097844      4.97975491159625      2.64842598870679
  C   3.63586574581749      5.29542947413703      1.36208048300620
  C   3.21736885836674      6.38461439754350     -0.67443428386040
  C   3.09545533803887      2.39559166361150     -1.01722979518157
  C   -0.19350792219825      5.32712006187501      1.05326741780475
  C   1.95419218296578      6.55960923217884     -1.38239922134467
  C   0.08461173977843      1.45121729094945     -0.16630421961719
  C   3.81102632862601      5.19652809506156     -1.01438935208076
  C   2.63963182028982      3.27168865916243     -2.06135478193015
  C   3.85226971417991      2.94630694352828      0.18131845639540
  C   -0.11869045267636      5.89674346096696     -0.30333823907280
  C   1.65364674466307      6.71749364157524      0.96937945239171
  C   0.88980331159602      5.86243389919442      1.84032097612833
  C   2.99032114837331      6.43938791114682      0.75312871706462
  C   2.99532723041761      4.70389048759392      2.40097434539026
```

```
Structure: C35_d.xyz
35
Coordinates from ORCA-job 35d
  C   -0.42398121447803      2.52563413727372     -0.49735514305587
  C   0.52403312162353      3.64789575896039      2.41430814907266
  C   -0.67038710720312      3.97734975529536     -0.60440453012408
  C   1.58729523726792      2.75709422779045     -1.88516547625450
  C   1.27250658724718      4.16198885669651     -2.17557599291796
  C   3.91958395505406      3.37117122687714     -1.30478088756320
  C   1.48339946639258      1.28677571858150      0.16008778350350
  C   1.47986117129029      2.57801853492279      2.24407537832257
  C   3.39288330426850      1.13643648691972     -1.23882839400900
```

```
  C   0.73250206167617      1.98795722862705     -1.00128228151981
  C   3.55099726733018      2.28866892885595      0.91669752393669
  C   4.23390748627248      3.34342393177328      0.11622177526201
  C   -0.46902001015982      2.19446235503267      0.93107006599375
  C   0.84846245718815      1.65045052421463      1.31257717899646
  C   2.37580530986970      4.94900992191518     -2.47935484836659
  C   3.70059654069017      4.58629342229408     -1.90624086691451
  C   2.92473501993626      2.37450930849244     -1.74865107020046
  C   4.07141724569122      4.54683724696997      0.80642324311234
  C   -0.68725304517572      3.32394145399710      1.62381715436623
  C   0.19965462703997      6.47622363787888      0.30996797529531
  C   2.91634130205375      1.36411577415581      0.10840295028584
  C   2.80638591615157      2.94788521643737      1.96647100089465
  C   2.21336295722930      5.35032315695853      1.95702618830749
  C   3.74144520755391      5.83606229380050     -1.20557843053871
  C   0.10400178961126      4.79604392300621     -1.50136922676810
  C   2.52219470077357      6.27855059487037     -1.91467426345820
  C   3.73171738231783      5.78564355755338      0.18129591380237
  C   0.24361905638105      6.14139849883019     -1.10640426071024
  C   3.16673344304617      4.32729754785257      1.94118884330463
  C   0.84246101445606      4.98820034442981      2.17779198682281
  C   -0.92269525820569      4.43480098625138      0.71511496871869
  C   1.58143662343266      6.95275733111755      0.21450774880626
  C   2.58196216427763      6.34895947242092      0.92786202878157
  C   1.51283775488990      6.85181945295909     -1.25143908137700
  C   -0.17196854387055      5.52880819117587      1.21475689586745


Structure: C35_e.xyz
35
Coordinates from ORCA-job 35e
  C   2.74148637136524      2.05455581863978      1.63225614544097
  C   -0.15545158054769      2.90522504685707     -1.38653554854093
  C   1.88651615629549      1.85117769551527     -1.07499518801074
  C   2.62823375743152      3.19638572424908      2.52263301767715
  C   -0.24445704588969      5.19941423056998      1.87652051031261
  C   3.25796732084269      2.25251994534673     -0.81453028666054
  C   -0.20270189662728      2.25386421189546     -0.16070117873469
  C   1.02053638595384      1.47122922340054     -0.01056339565011
  C   2.87733759580498      4.04538782648267     -2.59008908044956
  C   1.08911257320742      2.59136153492072     -2.03069150525202
  C   1.22106922389223      3.48891928522843      2.76288836397588
  C   4.48417480830858      4.63181944894160      0.02652051859573
  C   1.44545003330530      1.67593127557322      1.30673856527923
  C   0.47817384823194      2.56527969255369      2.00215886707207
  C   -0.36383679892985      4.32210085050654     -1.50890845224194
  C   0.59068274200869      4.84755585187097     -2.50041695530909
  C   1.51920441029537      3.74569141536918     -2.72751620871031
  C   3.41368435082737      5.18088429806926     -1.96379373404424
  C   -0.81698618146088      4.37688724274932      0.92498723991447
  C   0.59833946769678      6.21019760723127      1.18560191773809
  C   0.40625593448176      6.13664547987766     -0.16680676973760
  C   3.66587417074601      2.40085528304510      0.56960908574953
  C   4.29143400403041      3.62377396836946      0.97627635851033
  C   3.78237447267454      3.22154441108260     -1.72885972871076
  C   1.69494628659713      5.81174158888506      2.02363619365589
  C   1.41111193737811      6.34445015040317     -1.20015389583326
  C   -0.49644533383284      2.97675307417036      1.03464993940793
  C   4.50837883315493      4.35173503188834     -1.31623692965045
  C   2.80455891762079      6.03493233346474     -0.93781062439878
  C   3.48164816564842      5.68242436629599      0.28057527367819
  C   -0.58975867331635      5.05710771878070     -0.37680757172470
```

```
  C   1.20277444500541      6.10715935382588     -2.60043283526227
  C   0.82766705180537      4.83222614600465      2.72321109364230
  C   2.97578247300120      5.47455231655139      1.61466739058111
  C   3.48169678608024      4.17451956715605      2.05147940736553


Structure: C5_a.xyz
5
Coordinates from ORCA-job 5a
  C   -2.78462212149513     -0.54159659041958      1.81207774428175
  C   -1.38187484042164      2.29843689123008     -0.34722791850130
  C   -2.31757029035652      0.40398529173602      1.09320216806436
  C   -0.91477755636265      3.24397321620409     -1.06613407908968
  C   -1.84971518136406      1.35121119124940      0.37299208524487


Structure: C5_b.xyz
5
Coordinates from ORCA-job 5b
  C   -2.25447441910404      0.15143918713875      0.84200562450468
  C   -2.32359560274191      2.26030171093329      0.00523935856734
  C   -2.31345641283562      1.48625250988659      1.34812117204650
  C   -0.97429234901708      2.02723515424502     -0.39998461771166
  C   -1.38274120405685      0.83078143354530      0.06952846555066


Structure: C5_c.xyz
5
Coordinates from ORCA-job 5c
  C   -2.08600777157874      0.26406844470651     -0.29980512177996
  C   -1.15655264781594      2.74555727676234     -0.71228987143780
  C   -2.27889598272838      1.17842552768647      2.21405830056885
  C   -1.62684407936820      1.61307580359242     -0.28439141433383
  C   -2.10025950626424      0.95488294300120      0.94733810464850


Structure: C5_d.xyz
5
Coordinates from ORCA-job 5d
  C   -1.87627878941867      0.78331426382218      0.81118355771052
  C   -1.78694655705708      2.71345449725923     -0.67842375196894
  C   -1.92280806016640     -0.25217301720059      1.60971460827160
  C   -1.10096513369631      1.74322363677139      0.08877234919356
  C   -2.56156145800059      1.76819061509674      0.03366323975080


Structure: C5_e.xyz
5
Coordinates from ORCA-job 5e
  C   -1.90177406141352      0.19192137597528      0.22018017460985
  C   -2.53012884242576      2.05680102848527     -0.26695598679252
  C   -2.51147057814980      1.29528624378129      1.01589861323084
  C   -1.18836545371518      1.40735951004637     -0.26930358676732
  C   -1.11682105734302      1.80464184804429      1.16509078338491


Structure: C6_a.xyz
6
Coordinates from ORCA-job 6a
  C   2.32655872998026     -1.29592919280703      0.80008990907503
  C   1.70070752982877     -1.67039122054726      1.80755118286299
  C   3.15358044363642     -0.44099942175761      0.21868692110084
```

```
  C   3.91926939446018      0.31286415683457     -0.40644049472179
  C   4.72169597662792      1.05708165847351     -1.15148437066731
  C   5.47818792942328      2.03737403038737     -1.26840314764977
```

```
Structure: C6_b.xyz
6
Coordinates from ORCA-job 6b
  C   2.92186784512979      0.30490490365626      0.76515814820756
  C   2.47591257299413     -0.97093734778964      1.02754143753316
  C   3.38368623691558     -1.02246792117182     -0.00552377125785
  C   4.17780713367307     -0.30461673537821     -0.76536359340235
  C   3.71546896514121      1.02277086439588      0.00509066234902
  C   4.62525725010297      0.97034623628753     -1.02690288342955
```

```
Structure: C6_c.xyz
6
Coordinates from ORCA-job 6c
  C   3.09420135711941     -0.58408863023723      2.00114200539116
  C   3.37113259963630     -0.22877479764265      0.78045006964249
  C   2.58190363638115     -0.97388392077535     -1.63844536803325
  C   3.17477818968311     -0.37780085416707     -0.64510955745219
  C   4.97156767037199      1.55681836772610     -0.35421427080305
  C   4.10641654018123      0.60772983509622     -0.14382288932870
```

```
Structure: C6_d.xyz
6
Coordinates from ORCA-job 6d
  C   3.12712487045843     -1.60455933294276     -0.06105254859903
  C   2.45880358840328     -0.79589785790440      0.77595260302234
  C   2.42521327833341      0.50228826811283      1.11357924933726
  C   3.51153421652752     -0.02983087982564      0.02966144526612
  C   5.33203174356248      1.28890452408013     -1.24639391574203
  C   4.44529229079640      0.63909527318807     -0.61174683328466
```

```
Structure: C6_e.xyz
6
Coordinates from ORCA-job 6e
  C   1.80248576430425     -1.21324621083240     -0.25300544098431
  C   2.74019592461775     -0.88689442179334      0.59249036619130
  C   3.73106776732181     -0.56416553322737      1.52114352919366
  C   3.87210967714702      0.10598871023962      0.29654056188415
  C   4.82009305276993      1.67086405924889     -1.51878483113257
  C   4.33404781250425      0.88745340165638     -0.63838418515222
```

```
Structure: C7_a.xyz
7
-14.5569391917 converged=true
C  0.42509985757982332588 0.00000021787509585836 -0.00000044101934401221
C  1.69889532732642778434 0.00000000277095130575 -0.00000006205473803567
C  2.98965524670565852006 -0.00000015276184853408 0.00000025145300760868
C  4.26000000639695652893 -0.00000018294809272848 0.00000043885247631947
C  5.53034473458950959213 -0.00000010282415604242 0.00000033474567604304
C  6.82110469154111775936 0.00000004463391344116 -0.00000003583621106264
C  8.09490013576050593258 0.00000017335413669972 -0.00000048614087686065
```

```
Structure: C7_b.xyz
7
Coordinates from ORCA-job 7b
  C   2.87387974391938      0.15248680793076     -0.37557327712907
  C   4.00257693976652      0.40277014197728     -0.98794098676794
  C   2.40136295608619     -0.39848913034322      0.97714078956740
  C   3.72122381009352     -0.31202696239639      0.76570527741804
  C   5.11653449964655     -0.18500068966967      0.45439629306536
  C   6.39544640983713     -0.03416233063050      0.08508110068009
  C   5.30897564407355      0.37442216842351     -0.91880919683388


Structure: C7_c.xyz
7
Coordinates from ORCA-job 7c
  C   0.97418461959374      0.21265822668345     -0.55953223680617
  C   2.20485583235858      0.08370177618091     -0.22197629917566
  C   3.43108928701949     -0.04001378787048      0.10598322581151
  C   4.67659522148498     -0.16742015695627      0.44333776406838
  C   5.73716380599612     -0.49552404874043      1.29877460717268
  C   6.04956259470196      0.01128992399406     -0.02231609474522
  C   6.74654863168442      0.39530806670875     -1.04427097161730


Structure: C7_d.xyz
7
Coordinates from ORCA-job 7d
  C   0.97783637932194      0.27269853642427     -0.54323584449406
  C   2.20957609682221      0.11443725979968     -0.22227109549328
  C   3.43309952826966     -0.04417020325886      0.10046922104929
  C   5.73228950098355     -0.60585504256568      1.26025758767246
  C   4.67646290815622     -0.20893318943873      0.42954669851868
  C   6.73767327129054      0.47218312778800     -1.00865724081101
  C   6.05306231857872     -0.00036048874868     -0.01610933173386


Structure: C7_e.xyz
7
Coordinates from ORCA-job 7e
  C   2.10076604178238      0.03939926875251     -0.12023074432712
  C   0.81780373044623      0.06264873461757     -0.19430982684752
  C   3.37682830568370      0.01611317823744     -0.04799414263092
  C   4.66713705498562     -0.00604914252660      0.02457349326009
  C   5.84171949417505     -0.25894431378032      0.79156713234672
  C   7.08912266759576     -0.05321488601983      0.15607505331270
  C   5.92662270875412      0.20004715542744     -0.60968096511394


Structure: C8_a.xyz
8
Coordinates from ORCA-job 8a
  C   9.71973884575974      1.14922813739304      8.08890647890107
  C   11.02994687931005      0.97322662887878      5.27283685936328
  C   9.68662003997017      1.22300723884204      5.48610083395428
  C   12.24472766089269      0.73803816109367      5.43973987636705
  C   12.27756712992458      0.66279549247398      8.04250651975584
  C   12.44795332745509      0.66269929724674      6.80561101455548
  C   10.93440187492758      0.91342013202990      8.25579333049622
  C   9.51635524185999      1.22372891204183      6.72301308660672
```

```
Structure: C8_b.xyz
8
Coordinates from ORCA-job 8b
  C   9.48633745524912      1.21359850885857      7.68177968011587
  C   12.80429928721212      0.69964888687485      3.88015067753073
  C   10.79064476795677      0.89776427011080      8.52699628757405
  C   11.42067873183767      0.88529699114000      6.06294671500569
  C   12.13101406609816      0.79040234206205      4.93475181463854
  C   11.54290559226454      0.79154742387681      7.46248467037681
  C   9.56006687417779      1.13183473668394      9.02466577785614
  C   10.12136422904187      1.13605083661832      6.54073238119339


Structure: C8_c.xyz
8
Coordinates from ORCA-job 8c
  C   9.58472934304321      1.16903912900765      8.29364782980668
  C   11.61983819862810      0.84604911697268      5.82710064531330
  C   9.99293323736116      1.16030791055922      5.68415126577489
  C   11.03987099691447      0.98635136922319      4.68159713095622
  C   10.79058552668671      0.95051666288461      7.88548629260970
  C   13.25517872867248      0.48626436604773      7.70366458083773
  C   12.04509904197977      0.72925456500003      7.20334066539241
  C   9.52907592526038      1.21836088182200      6.83551959360033


Structure: C8_d.xyz
8
Coordinates from ORCA-job 8d
  C   10.26055613773987      0.63562553577394      7.50210083166181
  C   12.68912092535047      1.55000081776230      8.25211304827364
  C   9.47189710128311      1.32675326687828      5.01309128405494
  C   10.64517612971886      0.69895288669466      5.41216726600004
  C   11.33672371247090      1.27577822827696      8.09290718694233
  C   11.47202224119297      0.08413689190171      6.33679682940838
  C   12.34408141336089      0.81698715156339      7.12375967074955
  C   9.63773334272098      1.15790922266587      6.38157188720057


Structure: C8_e.xyz
8
Coordinates from ORCA-job 8e
  C   10.49730049721835      1.00991053955580      7.83770104120451
  C   11.46586026410282      0.87228088192120      5.69069748386929
  C   10.30907590047619      1.14003987086403      6.44816304377970
  C   12.00481420112885      0.79612002296278      4.49551807713877
  C   9.95793856653638      1.08560309862950      9.03272444029398
  C   12.52487622319635      0.72765478469900      3.34065721608084
  C   9.44344417319585      1.17281872423654     10.18882607192672
  C   11.65400117269149      0.74171607864827      7.08022062999747


Structure: C9_a.xyz
9
-18.7876109972 converged=true
C  4.01824362466928253212 0.80981247098727549005 0.53849836319319588362
C  4.61600237379344058297 1.58864697170193402975 -0.25905249507461164393
C  5.09660005601708476775 -1.29932263512968870778 1.25325690717186843948
C  6.25995560819870711100 -1.52930997678652547123 0.69861976098120415202
C  7.21553207908813920568 -1.34969408223587916673 -0.15754420423991782885
C  4.34644272128284736567 -0.27449272182294737199 1.23375570573400139551
C  7.17796477834245383320 -0.29531088128733856690 -0.91109576625654431492
```

```
C  5.64325563295850418655 1.47328295355840088732 -1.09509898437285269068
C  6.74600312244569710884 0.87638790630654106462 -1.30133929242811552385
```

```
Structure: C9_b.xyz
9
Coordinates from ORCA-job 9b
  C   2.58494811133403      0.29266114601313      1.97262055547110
  C   3.53131919942021     -0.58660722383214      1.92019281407283
  C   4.35174096354697     -1.75919444064119      2.16328360302997
  C   4.79105664480694     -0.83870398005427      1.21318379578385
  C   5.59665786108243     -0.35642116291020      0.30891422613478
  C   6.37732073139676      0.10921416043685     -0.56656811382618
  C   7.16656752122898      0.57657821773453     -1.44933394428161
  C   7.96222487300390      1.04694672001660     -2.33886085680637
  C   8.75816409097589      1.51552656323670     -3.22343207428661
```

```
Structure: C9_c.xyz
9
Coordinates from ORCA-job 9c
  C   5.08056526912945     -0.34570806717516      0.67183219195240
  C   4.59092739104697      1.54419931125469     -0.30588575043234
  C   4.10802037481126      0.77367576813070      0.58723627673279
  C   7.23000811510611     -1.41150958964509     -0.12613014085989
  C   8.12337904138207     -2.29653480990247     -0.12391714544062
  C   3.99609269624076     -0.37714330154793      1.48034465465750
  C   6.28028831793061     -0.45181388693579     -0.11841699901822
  C   5.56400009048026      1.82979483832534     -1.20601531270009
  C   6.14671870596040      0.73503974278748     -0.85904777489152
```

```
Structure: C9_d.xyz
9
Coordinates from ORCA-job 9d
  C   5.90727743972529      1.34431083042869     -0.81816277306298
  C   3.83734134495068      1.36287115692237      0.45079113038062
  C   4.11277800641487      0.22031206907972      0.90259684187959
  C   6.05839931377925      0.01299803461108     -0.42548031896277
  C   6.27760053203884     -1.34408150349825      0.99548067243930
  C   7.00568851492766     -1.09948606226464     -0.33262997302788
  C   7.92542013394941     -1.68747624547942     -1.03280005919988
  C   5.14430195784140     -0.60895183815330      0.54733997737828
  C   4.85119275846048      1.79950356893731     -0.28713549253252
```

```
Structure: C9_e.xyz
9
Coordinates from ORCA-job 9e
  C   4.93456449518424      1.76712120622418      0.05519822660559
  C   5.59442954633097      0.56612574715274     -0.50533032434601
  C   6.20551318222374     -0.52209152119154     -1.07090630820440
  C   5.99629153828100     -1.45848224792585      0.98867662593477
  C   6.43890002049050     -1.67223633546918     -0.24131756880588
  C   5.42472334659554     -0.54220255228311      1.65909364757179
  C   5.23318746026524      0.53863445594815      0.78397738833932
  C   6.82841401729709     -1.65280508838490     -1.61447350153847
  C   4.46397640071133      2.97593634122129     -0.05491818026495
```

S3 C_nNH_2 family: Energies (Hartrees) and xyz coordinates (Angstroms)

```
C5NH2_a -246,19337
C5NH2_b -246,20872
C5NH2_c -246,20223
C6NH2_a -284,24773
C6NH2_b -284,22616
C6NH2_c -284,24260
C7NH2_a -322,36333
C8NH2_a -360,44250
C8NH2_b -360,41487
C8NH2_c -360,40236
C8NH2_d -360,37976
C8NH2_e -360,37095
C9NH2_a -398,49880
C9NH2_b -398,45633
C9NH2_c -398,46222
C9NH2_d -398,45072
C10NH2_a -436,61614
C10NH2_b -436,59595
C10NH2_c -436,56548
C10NH2_d -436,55553
C10NH2_e -436,51194
C15NH2_a -627,06147
C15NH2_b -627,05129
C20NH2_a -817,50441
C20NH2_b -817,47527
C20NH2_c -817,46837
C20NH2_d -817,43618
C20NH2_e -817,42326
C25NH2_a -1007,94890
C25NH2_b -1007,95249
C25NH2_c -1007,92812
C25NH2_d -1007,91412
C25NH2_e -1007,91873
C30NH2_a -1198,59076
C30NH2_b -1198,58628
C30NH2_c -1198,56904
C30NH2_d -1198,56247
C30NH2_e -1198,55162
C35NH2_a -1389,13316
C35NH2_b -1389,11626
C35NH2_c -1389,11395
C35NH2_d -1389,11545
C35NH2_e -1389,07628

C -37,84496
N -54,58957
H -0,50211

Structure: C10NH2_a.xyz
13
Coordinates from ORCA-job 10_1 E -436.616144846712
  C          2.12435291440562      0.95000345701131     -0.06273610052391
  C          1.09623804411419      1.70743561700640     -0.58013434508543
  C         -0.10923883121846      1.88427367804271     -0.82406545035455
  C         -1.39872789580839      1.61664214830772     -0.86645053360298
  C         -2.21599597854390      0.71101072106534     -0.53145017181868
  C         -2.41211672712830     -0.45411855042195      0.00677826804647
  C         -1.57809084866102     -1.29992249444792      0.53080027471991
  C         -0.38206514731664     -1.58735448389548      0.82624750661707
```

```
  C          0.83148447558849     -1.08085597396218      0.74211510062405
  C          1.71949388811425     -0.25939034525355      0.45863681767148
  N          3.53423306766845      1.36075521067672     -0.12922636428416
  H          4.08523728154713      0.93692981195219      0.58963828468242
  H          3.63746576557800      2.35493120767220     -0.16016328284915


Structure: C10NH2_b.xyz
13
Coordinates from ORCA-job 10_2 E -436.595948024285
  C          4.62628012724735      0.22213811505940      0.71009612468502
  C         -6.65998780757580      1.01892963529741     -1.35229761132728
  C         -5.40868695317665      0.91630519288770     -1.12928798458436
  C         -4.11917110009752      0.81550133166824     -0.89858075735600
  C         -2.90248837891456      0.72187185118843     -0.68015291572117
  C         -1.60082663666426      0.62244132717895     -0.44615816444927
  C         -0.39758081337485      0.53397156058956     -0.22779714294090
  C          0.91963750875229      0.44087343695355      0.01296643039903
  C          2.11316307716421      0.36104027155006      0.23361195720870
  C          3.44383568801908      0.27680951590457      0.48195996575372
  N          6.06836738919123      0.06206787996290      0.94602380261135
  H          6.26690204556964      0.10565900237373      1.92514779349813
  H          6.58282585161568      0.74273087784723      0.42445849548202


Structure: C10NH2_c.xyz
13
Coordinates from ORCA-job 10_3 E -436.565475815802
  C          1.80000733686450      0.95061281635240      0.09492989936461
  C          0.80732525160297      1.70646326974896     -0.48998240518824
  C          0.09837019707377      2.66243606985878     -1.10418197399471
  C         -0.72780087035679      1.62312938255994     -0.77367454431327
  C         -1.60171529395061      0.69486402515864     -0.51472264339181
  C         -2.01293416472494     -0.42580323269565     -0.07380248392406
  C         -1.49622739855077     -1.45032216370895      0.52523651197103
  C         -0.40751089491140     -1.92866474345593      0.98421744250612
  C          0.72965870663953     -1.23752623355773      0.90416212372327
  C          1.43595261175902     -0.26667214707214      0.60319670954313
  N          3.15743743027107      1.50305557483681      0.20934381893265
  H          3.83776277152598      0.77906667210980      0.09533721733430
  H          3.31194432509708      2.22970072420212     -0.46006967930402


Structure: C10NH2_d.xyz
13
Coordinates from ORCA-job 10_4 E -436.555526054983
  C          1.91907215470212      0.52644915858756      0.24091202400792
  C          1.27940659234649      1.49009860934713     -0.41225056236917
  C          0.26931492317410      2.15483199197400     -0.97402879234038
  C         -0.90347666349515      1.62740637888401     -0.91462482088148
  C         -2.00047580556088      0.95141471399560     -0.75917515081608
  C         -1.93653384915743     -0.19152892672047     -0.12117347796185
  C         -1.45164279383435     -1.17376286576487      0.51128756272867
  C         -0.43119946549292     -1.79312351740797      1.05056961836070
  C          0.78022712859647     -1.31294863262401      1.02446194424895
  C          1.86640118195311     -0.66183796189178      0.88104693873414
  N          2.85541749081944      1.58793186644094     -0.15583982453874
  H          3.48637234328080      1.41907139340873     -0.91305981980630
  H          3.19938676571584      2.21633778494110      0.54186436447613
```

Structure: C10NH2_e.xyz
13
Coordinates from ORCA-job 10_5 E -436.511935074622

C	2.74456416765476	0.29587983448990	0.60799707189581
C	1.49739284380381	1.99959796201421	-0.70444756715415
C	0.30582697794813	1.83071241473602	-0.86259816693310
C	-1.02680663414739	1.58590690963997	-1.01351001398540
C	-1.79222410083535	0.69271569114939	-0.64976800156840
C	-2.39420738119745	-0.41144257003726	-0.12244241157526
C	-1.57792793741450	-1.13453787763555	0.49602542215875
C	-0.49102568262437	-1.66953269639263	1.06288946966230
C	0.66382835781596	-1.16036491844148	1.01643161117203
C	1.74207166349975	-0.43855299149720	0.82311409014501
N	2.73925668415906	1.54684137792966	-0.16399202196840
H	3.39830714409676	1.44005246387565	-0.90847077582310
H	3.12321389499670	2.26306440921460	0.41876129252467

Structure: C15NH2_a.xyz
18
Coordinates from ORCA-job 15_1 E -627.061472663596

C	-2.32707250388482	-0.29926139576019	2.55635293316304
C	0.70509626895227	1.21834324828049	2.50117516619738
C	1.49859999923464	1.47728876207094	1.45194610261228
C	1.92841543654623	1.42161061320411	0.31242176037889
C	1.87631184572633	0.50181837337902	-3.16616658240538
C	3.32879711690843	1.29209974233467	-1.79266507499955
C	2.17427887665793	1.21937741020066	-1.03862963316549
C	1.17788869349513	0.68406666510403	-1.94853769980264
C	-0.06731791820503	0.32936230193202	-1.55129768677601
C	-1.10664997964064	0.06031856937229	-0.93571420185591
C	-1.84723734040845	-0.21908985639518	0.10829338062317
C	-2.34422265714158	-0.35164010341597	1.24162801725805
C	-1.35634955233176	0.26281589786838	3.53268600951980
C	-2.49981031632583	-0.31170109220911	3.92261365885274
C	-0.17760893322708	0.87316307681436	3.27742227281616
N	3.13938262070743	0.86554318400383	-3.06160307739319
H	1.46570039593414	0.11312653820148	-4.08854473964402
H	4.30244795558071	1.62753806400194	-1.48040060547656

Structure: C15NH2_b.xyz
18
Coordinates from ORCA-job 15_3 E -627.051293773789

C	-2.50831352752988	-0.23325440102295	2.31460067521129
C	0.71367804658248	1.12040368409274	2.82707957654812
C	1.17061305769497	1.11487367018029	1.67478838156809
C	1.01718207577029	0.84279226148482	0.35415421489716
C	1.90725055342780	0.54957632047417	-3.61861422996618
C	2.88203334563169	1.25362273527656	-1.64518873254662
C	1.75658700116583	0.92252888913879	-0.95857914817064
C	0.72230206517301	0.19129185505446	-2.95562113967096
C	0.63831105672059	0.37312522839240	-1.63080714317652
C	-0.11118139025523	0.28755702155057	-0.33192575141498
C	-1.28945461073693	-0.09023117459837	0.20649913607982
C	-2.18109214142181	-0.30862716134373	1.04254127972557
C	-1.20968208057850	0.55368543059831	3.98634801610389
C	-2.33360294429760	0.02876319039437	3.52891027358063
C	-0.04264095301415	1.00047331004925	3.91735373033600
N	2.94171852023098	1.05981611802073	-2.98291477052334
H	2.02740022256462	0.42338971443295	-4.68468917426655

```
  H          3.76954170615811      1.67499330152063     -1.20295520499560


Structure: C20NH2_a.xyz
23
Coordinates from ORCA-job 20_1 E -817.504408349606
  C          1.11947898109253     -0.28306140607834      0.64456443624706
  C          2.60578614427616      1.60828196122309      0.68925747461931
  C          2.45615472017670      0.23834316900559      0.50296367364399
  C          4.64524873664241      0.41252829285050     -0.45670613697207
  C          3.68116183335781     -0.23567361532083     -0.05250158785286
  C          5.44145178665200      2.88682297631620     -1.17198827998540
  C          0.10202787860806      0.61165811822842      0.79987712429415
  C          1.50919397225790      2.48082023992675      0.86095704538180
  C          3.71237369338036      2.37370082868073      0.21353411195872
  C          3.28378805025251      3.72535304400848      0.08426504412756
  C          4.79910316946114      1.82186246257915     -0.49720664390110
  C          5.06965786551236      4.05545848349606     -1.28170396508080
  C          0.18872436600951      2.01353435378225      0.82788407907960
  C          1.02251337374118      4.79644821989287      0.05228358963893
  C          1.91581913142610      3.79186963701009      0.47811353060825
  C          1.78926859120346      5.76131373985627     -0.64286370138832
  C          3.89981220636944      4.66692188103651     -0.77085872454054
  C          2.97277442100366      5.71514878495963     -0.98022770173834
  C         -0.61663018500200      3.15843452902851      0.57069121435069
  C         -0.29444679033167      4.30026830774328      0.24724359932030
  N          0.90692649526419     -1.73745791864443      0.62329873893153
  H         -0.01903351458673     -1.97992497182446      0.33380299780739
  H          1.60392508141295     -2.20728111963047      0.08158006049060


Structure: C20NH2_b.xyz
23
Coordinates from ORCA-job 20_2 E -817.475266673134
  C          1.27262254941213     -0.06835811439098      0.32859791164522
  C          2.54556702200988      1.34977806905492     -0.86085622687059
  C          2.57077585060715      0.08197785292317     -0.27601639405903
  C          4.93630796873028      0.45511437072905     -0.36100064947624
  C          3.94484721646522     -0.25345530760476     -0.16780327064347
  C          5.67301952345041      3.03630512971187     -0.45938918561266
  C          0.52866406057583      1.18714672280185      0.21806576897221
  C          1.36106563475905      2.05832331239451     -0.52483638626520
  C          3.62351226462083      2.22888342387312     -0.99284491305955
  C          3.09476157701962      3.54619739506712     -0.81681224495676
  C          4.95164729095843      1.84309321302312     -0.66741714219780
  C          5.24227951667555      4.18859675466453     -0.37724243029172
  C         -0.61169727458586      1.60062364985092      0.95926542525177
  C          0.96459333469317      4.48122067139935      0.16249236045114
  C          1.68151362296877      3.45020582737100     -0.50304253760051
  C          1.85768676319792      5.58380268564533      0.25311462340058
  C          3.90703047384582      4.64034928694035     -0.42493955199413
  C          3.06502178127114      5.67665294931784      0.04501197899046
  C         -0.61684049954998      2.92632212387007      0.84485670866908
  C         -0.25677565553629      4.14085356870722      0.78966487523601
  N          0.72647996656709     -1.24727543512958      1.01618953095454
  H          1.39384331540494     -1.89867640904193      1.37716449915024
  H         -0.06084629538111     -1.03231174305224      1.59403723993039


Structure: C20NH2_c.xyz
23
Coordinates from ORCA-job 20_3 E -817.468372185251
```

```
  C          1.24708234484745     -0.14440474663291      0.41832253499140
  C          2.28597602793506      1.29076235634264     -1.02328891225783
  C          2.47851047845705      0.09404343067215     -0.31805452898492
  C          4.74621846912269      0.75402439845262     -0.42629863627454
  C          3.87067614556809     -0.08797037160484     -0.19476214018233
  C          5.22677959888414      3.36089988767438     -0.48877946410134
  C          0.38327217373529      1.00915937462447      0.25247133079677
  C          1.02066420912966      1.84907700073480     -0.66917145718016
  C          3.23414709015941      2.31965515136185     -1.12118592772453
  C          2.51389640849070      3.57026247034932     -0.85737280947223
  C          4.58680152072511      2.11645553519277     -0.76833043340958
  C          5.99424709662426      4.51777239131354     -0.16787143288629
  C         -0.46848944198773      1.84418773378812      1.01121492282818
  C          1.04484028777934      5.18253159823078      0.67470686773355
  C          1.14004235594781      3.24695489225885     -0.55501761348962
  C          2.19993130404488      5.46912676904843      0.35729489596881
  C          4.57997364270087      4.53176109303489     -0.30600769357604
  C          3.15990385763086      4.70102327715141     -0.33490184767893
  C         -0.53021801500041      3.07666792450557      1.03647565146867
  C          0.37749073294275      3.97161814144627      0.41940180424337
  N          1.03409934836318     -1.23706973639408      1.37833372440284
  H          1.59010236261663     -2.05274143073317      1.21852029866831
  H          0.07913199358761     -1.40842713740025      1.62056086632398


Structure: C20NH2_d.xyz
23
Coordinates from ORCA-job 20_4 E -817.436177036348
  C          1.05366304459784     -0.32975134055593      0.50231368659354
  C          2.18980339195135      1.63253471851362     -0.60861897042031
  C          2.15686790509529      0.26010339581441     -0.10424124079616
  C          4.50024091483763      0.34584197320228     -0.35817296081461
  C          3.48304296766416     -0.30913447638345     -0.13991391416844
  C          5.46176722705963      2.85006118838864     -0.43482693960317
  C         -0.03675373255370      0.52203798947606      0.70314782241418
  C          1.31834413575821      2.84002499353871     -0.39993668428066
  C          3.39704322941172      2.13593917222246     -1.05742628832564
  C          3.39216340715988      3.53452718766046     -1.04666114304990
  C          4.74690589443698      1.70110842772507     -0.67075619433146
  C          4.63618010232447      4.02652518898933     -0.60929882104892
  C         -0.37103182032948      1.68985782447946      0.67700234350223
  C          0.69616776747690      5.22474546351859      0.60849985816684
  C          2.13521896054424      3.99203076154733     -0.62254521878020
  C          1.98995602195239      5.24272090202581      0.04509194873790
  C          4.32263916429801      5.30034790130734     -0.10017744773292
  C          3.25270597358745      5.87272896145648      0.14739583040822
  C          0.11491418342558      2.95944023713695      0.32365878587382
  C         -0.10536554955451      4.29791985214790      0.71179796892706
  N          1.05263745800068     -1.73869830360704      0.92155992493053
  H          1.96912557233906     -2.13387672505325      0.98387803906829
  H          0.43884377282092     -1.94166530600893      1.68448960964555


Structure: C20NH2_e.xyz
23
Coordinates from ORCA-job 20_5 E -817.423259364709
  C          1.33646234050648      0.10355342139357      0.55146582215876
  C          3.09941669708518      1.53070357956465      0.87922370536658
  C          2.81953391200553      0.24614280619438      0.49259475609289
  C          4.91006033718163      0.40569962047242     -0.63689679831675
  C          3.93631337444351     -0.23243527759711     -0.22758716299584
  C          5.55501275777654      2.89575007441221     -1.34428419284520
```

```
  C          0.76399216147082      1.31715825960541      0.81073217586046
  C          1.85723268725201      2.31306606187627      0.93489170840521
  C          4.17347297765162      2.31061764536540      0.39982545584702
  C          3.62306652276100      3.61119814127323      0.30214154292793
  C          5.10648745856414      1.80846137880118     -0.54565958940778
  C          5.01035962588061      4.00021111464046     -1.41077819794134
  C         -0.55403352504042      1.84673029335523      0.67652244425051
  C          0.19438447758909      4.87221318204660     -0.13171730800604
  C          2.18223808754681      3.59497445131944      0.59390195946807
  C          1.61507522637741      4.75793148054172     -0.16436134322412
  C          3.89842094300742      4.50844778562316     -0.70851138023591
  C          2.64319995783013      5.25515847932006     -0.88791340155816
  C         -1.11031693487875      2.90870504022839      0.47912810519225
  C         -0.78750406909463      4.19123670970790      0.07926610034101
  N          0.65711875160284     -1.14026327488456      0.16119371304280
  H         -0.30321060612859     -1.16830771499104      0.43864826771471
  H          1.16829683620609     -1.96158325485136      0.41443362336227


Structure: C25NH2_a.xyz
28
Coordinates from ORCA-job 25_1 E -1007.948902297502
  C          2.71790694572696      3.71422358410877     -2.50497365092656
  C          1.62548410796676      2.89661551058038     -2.19534221764590
  C          1.08406820157721      1.29931384679975     -0.43256658918114
  C          2.08687095792008      1.67240655942711     -1.44022601077185
  C          3.96216085572393      3.18385686565276      0.80225736747044
  C          2.50610221716402      4.80220048378457      1.52539738421258
  C          1.11902973767224      4.53511241001357     -0.34063672274884
  C          1.04786234997643      1.24178484299413      2.03424736750777
  C          2.44859131416460      1.52773488663905      2.46086009378781
  C          3.63310851115426      4.03386408615808     -0.37101445062649
  C          3.72225769340750      1.83901350484854      0.37001510650268
  C          1.54145805344023      0.74736469434065      0.76273927434959
  C          0.18521724385854      2.41210206250408     -0.34652743245829
  C         -0.14404641106759      3.00294059419313      0.89164575054626
  C          2.48150449154009      4.81365092247567      0.07373992404974
  C          2.93125128661375      1.03184355733380      1.18544017952826
  C          0.75924315809915      3.44662108504801     -1.24527894290723
  C          3.33643401980909      3.71428674233778      1.95039695357655
  C          3.68015828550841      3.31624976433176     -1.57061964022473
  C          0.31274046333046      2.39932684827078      2.14513353015964
  C          2.53270443655528      2.85298893641327      2.82064669645323
  C          1.18302118072242      4.49630037404543      2.01534021695871
  C          0.32598788158520      4.35655531772712      0.86202609910712
  C          3.36336281723593      1.93345073896419     -1.05222285997977
  C          1.15462990580753      3.38945237921493      2.85151914584459
  N          2.70486455961158      4.95432929188137     -3.29419335075215
  H          3.38929264737845      5.62976569695640     -3.01970500632743
  H          1.79211306522526      5.30394441921758     -3.50550820608219


Structure: C25NH2_b.xyz
28
Coordinates from ORCA-job 25_2 E -1007.952485260308
  C          0.96901303882345      4.48765563216774     -1.67475013995952
  C          2.68613719215405      5.00333999819650      1.38847596591630
  C          3.17382695784509      1.11279633383429     -0.41443853129842
  C          3.51448748365418      3.46926637664169     -0.93707881760179
  C          4.16017507882283      2.48052583770523      1.25481179229099
  C          3.69514588128613      4.83388832872786      0.47236001815974
  C          3.16354118962349      4.91293878276623     -0.90696028296765
```

C	2.25993863357616	1.55797375242228	2.32576336705264
C	0.92771227619076	1.15770642224289	-0.12081584428811
C	4.28352056371859	3.53041516864848	0.28634409229830
C	2.70645992027346	0.62526455980931	1.40691622279041
C	2.03181438383824	0.36222924868425	0.17668074182535
C	1.29636205036306	2.18428650109143	-1.13418860071292
C	0.03424312491589	3.31343307658128	0.25941656623795
C	3.20286096266157	2.68652643943368	2.28794057612864
C	1.74427462362225	5.23831484008831	-0.77800799235390
C	0.38851529813781	3.32659462231042	-1.10709489999990
C	0.30602965308710	1.95955208641195	0.87121745778976
C	2.72287109984546	2.27192167010420	-1.17620654858663
C	0.95408635358507	2.16019150342015	2.09850389596819
C	2.43544534429170	3.91851210364894	2.33572415487514
C	0.48053635548282	4.31793242743896	1.13225262996364
C	1.46528212871953	5.20391425778170	0.61543364265069
C	3.94858637422571	1.16284417241425	0.73611774305266
C	1.08816790062750	3.58645154758941	2.25779833434463
N	1.37437095943913	4.44192803710821	-3.08701567735776
H	0.75272938518774	3.93395535730168	-3.68327527315672
H	1.71724578487604	5.30694092169131	-3.45333459422285

Structure: C25NH2_c.xyz
28
Coordinates from ORCA-job 25_3 E -1007.928120202352

C	1.77677118192878	2.88779005298857	-2.47227845078965
C	1.77595182440170	4.62917447051151	-0.95730468979612
C	1.93731910965872	1.34682339095708	-0.55444773869685
C	2.56250920913812	2.00020259191879	-1.70589511406537
C	4.14094896829323	3.42193275094925	1.17007778041925
C	2.56016139383156	4.58983994851090	2.38175447049625
C	1.45115625547922	5.06487724438044	0.34212396850210
C	-0.16980264197937	3.27295839449260	0.57753558813478
C	2.36180011740995	1.33761513678481	1.92582764511538
C	3.73195710881508	4.64219849842043	0.40052372161560
C	3.84998425735394	2.35395052233373	0.29674301666022
C	0.59711129464498	1.58907543181983	-0.31656340491266
C	0.11632572038986	2.93228418645155	-0.77323121050007
C	0.13276910101527	1.87416352128440	1.03474772571416
C	2.70727624189339	5.22198636629996	1.12231141616858
C	2.86958623060525	1.37081421491872	0.61065864218577
C	0.90735680954183	3.68872382275724	-1.64225489237149
C	3.51485825118200	3.44366855575788	2.44293539558884
C	3.22870079621628	4.25608673978509	-0.89929825756871
C	0.91131817103196	1.64709974637319	2.11137757076605
C	2.75919998800100	2.34870918873552	2.85421979416561
C	1.21730974829938	4.07989373185077	2.48332871440077
C	0.53201505968054	4.33632227396010	1.16956702410222
C	3.50442209179531	2.87671048691350	-1.05692466498201
C	1.32135997707670	2.77463183154674	2.93611373161795
N	2.21353113539640	3.34771385834303	-3.79840470465608
H	1.90547482098155	4.25017183450896	-4.09952081986265
H	3.06600777679221	2.96188121799978	-4.15113226919654

Structure: C25NH2_d.xyz
28
Coordinates from ORCA-job 25_4 E -1007.914123054973

C	2.53520551946018	3.18152183845970	-2.18373635047492
C	1.32863848220022	2.58850055890964	-1.70142134356861
C	1.70434571715329	1.38960462333672	-0.87397479938895

```
  C          3.55105023025953      2.76242203470724     -1.27484170456447
  C          4.20943777488098      2.67587861965039      0.92769997346712
  C          2.94155824179414      4.42079298460275      1.54328952231341
  C          0.48178156113269      4.51688509690659     -0.39594151674774
  C          1.19401910571491      1.08296314291651      1.52100947594211
  C          2.60244873327499      1.22011674026279      1.81009943303492
  C          3.45574982435397      4.80383163054993      0.23805867488079
  C          3.51999274042268      1.40830570190414      0.72855836617744
  C          0.77302855179720      1.17610637693625      0.17960715818977
  C         -0.14866566872837      2.30655217334642      0.04938801700168
  C         -0.24262727052184      2.93000804692806      1.26306772129091
  C          2.53358237594031      5.50154167406671     -0.46649834154471
  C          1.32383178823450      5.55024984017376     -0.75844822022882
  C          0.27423153385516      3.22535059629761     -1.04402177399095
  C          3.64112845343863      3.25626943697911      2.05649593312035
  C          4.08728606160911      3.54052649464954     -0.24821817056719
  C          0.46978171901152      2.15498336951363      2.25417703459612
  C          2.77113983496631      2.34268945704497      2.71985925950438
  C          1.54799705157416      4.25702515321746      1.89448235602731
  C          0.41746823459829      4.22182736672361      1.03018807778468
  C          3.05859939464268      1.49353215848596     -0.60318023707340
  C          1.49150212550507      2.97216147653112      2.70401543585329
  N          2.66319583294449      4.16241459228993     -3.27110375593560
  H          1.82155130675854      4.66511178128250     -3.46841068325980
  H          3.47612074260146      4.74012705546483     -3.19760955887556
```

```
Structure: C25NH2_e.xyz
28
Coordinates from ORCA-job 25_5 E -1007.918732839070
  C          0.98809718038893      3.34735690047777     -1.91301691938129
  C          1.48392075365086      4.54990645107908     -1.39174382312544
  C          0.80055332078492      2.30202124803128     -0.90639477616192
  C          3.62143797671909      3.41345042016572     -1.72641927380871
  C          4.33792530981544      2.59983019962925     -0.87554136387475
  C          1.99635324266820      4.74388496617222      2.03471467926449
  C          3.31286677611822      5.00479180292218      0.02217442699524
  C          1.41296354835883      1.21605963563594      1.65727752356233
  C          2.43865661588423      1.24853443652296      0.65412849822399
  C          3.96503126019369      4.11605048831996      0.93335663853936
  C          3.51439557928782      1.94415469190874      1.26893861557460
  C          2.06705051063095      1.63837692651038     -0.71369893933398
  C          2.97109742966043      4.57573698923912     -1.32261559942749
  C          0.23334740299170      1.90301135622994      1.48919102789286
  C          3.16009000520324      3.98547121992754      2.19575445883173
  C          2.09569875387530      5.33580097525631      0.71199458433801
  C          1.03717185031317      4.89571615985305     -0.07719067640460
  C          3.04920672320563      2.58761269393417      2.51771235884395
  C          4.35611564809629      2.83040145289061      0.49805961467047
  C          0.20886991187185      4.03628978752069      0.72608279042610
  C          1.81536911972092      2.04638397505122      2.84909226968589
  C          0.69890223305529      4.09120642018223      2.12895156856162
  C          0.09171848595466      2.69473325531167      0.30933584864310
  C          3.20556598987806      2.01065803238649     -1.52866779199992
  C          0.63710411285750      2.81172858986784      2.63661916308122
  N          1.11249314856134      2.98745564654057     -3.33283970247622
  H          1.65304408395841      3.61904747597657     -3.88862423184076
  H          1.21833302516988      2.01162779284384     -3.52404097575280
```

```
Structure: C30NH2_a.xyz
33
```

Coordinates from ORCA-job 30_1 E -1198.590760597089

C	1.23204811386830	1.55906270434579	1.87531919971279
C	1.90370810475058	1.21755264798105	-0.79358884431912
C	0.57282420684593	2.80678483464487	1.98339702278618
C	3.41997808118236	2.66488033224536	1.90234408487088
C	2.38581288085210	2.18767434921533	-1.74535474708302
C	0.60674241806109	1.27181060657719	-0.33142221814117
C	0.29101138833821	3.44044091542860	-1.33729449794254
C	2.58238753725994	1.53029453641684	1.60044106351532
C	-0.09634938188026	2.49888667285312	-0.37417075150258
C	2.77661766480152	3.87969593767941	2.29727736234636
C	3.76909407578273	2.41861267989445	-1.40753907366015
C	4.02186412112269	1.71253515353516	-0.14534231213413
C	-0.09578897324339	4.59142268152129	0.64572944959876
C	1.29416931453953	5.51059690513062	-1.15394435553338
C	1.33681909121578	3.92253500829195	2.35048385569540
C	4.41040759637314	2.71455378818816	0.85469959334027
C	2.95570315693650	0.96611851898252	0.25468445603495
C	4.27035865739444	3.69842041387762	-1.27324294635545
C	3.17789067835897	5.11143403186177	1.66812251221784
C	0.85790047171555	5.08451712247876	1.59284684923366
C	0.19541834978539	4.77872782775357	-0.74332771625438
C	4.68276996455039	3.88851364254153	0.18029148709055
C	2.13461343965856	4.63072538365206	-2.01025758876230
C	1.55066154970741	3.31413459863856	-2.02649903736884
C	-0.31972089482765	3.19750102501117	0.89162447618471
C	1.95879063103368	5.81694602354309	1.18871981973741
C	4.11595822570399	5.10638938730237	0.61262981854758
C	2.15858069768242	6.12678896270571	-0.19407066102119
C	3.48617905094747	5.74622161496915	-0.52069831868520
C	3.48087531175291	4.81377645797315	-1.62563356232298
N	0.48608152315966	0.64075308704689	1.00288505041337
H	-0.47163172788112	0.57210174915265	1.28229929206147
H	0.89991467445829	-0.26959959500003	1.00322124489074

Structure: C30NH2_b.xyz
33
Coordinates from ORCA-job 30_2 E -1198.586278494393

C	0.93418836668465	2.39023379176707	2.01520980331217
C	0.73927718072162	2.07263595894149	-1.64475893085115
C	2.00039541102300	1.38015206141241	1.47911188247723
C	4.23195462533614	2.05076152420191	0.64781337744354
C	2.86820234357057	2.69189805345291	-2.42490144827647
C	2.05962425054982	1.59224796959275	-1.96273002124426
C	0.64729731728467	3.41488008769043	-2.06044237044932
C	3.30506609086674	1.87703751597961	1.71751936095985
C	0.52415755039510	1.76203516653085	-0.28246727745777
C	3.90038008111582	1.59436038274506	-0.61603064821290
C	3.97461524809730	2.75641817557173	-1.56248459323047
C	2.65826200265374	0.94955615250101	-0.83478554206154
C	0.20268573582479	4.38670631949390	-1.17620200031494
C	1.16644285479402	5.49200209133029	-1.27151629732130
C	1.85497096065558	3.53702927729509	2.43094149482862
C	3.96638706477850	4.15864638617540	1.64017439927843
C	1.68066022220242	0.95353907870011	0.21360693094165
C	4.27832255767497	3.94704132340281	-0.80179778026102
C	1.74170639524561	4.85484100530647	1.93557892917922
C	0.05648787653805	4.08795959461818	0.23100902833414
C	3.17657923444618	3.15077147748643	2.35701599125202
C	4.53312882601097	3.50540264610841	0.55078583424131
C	2.35902518187122	5.02295711264793	-1.96390455836368

```
  C          2.02251433947047      3.82417633939546     -2.55988483331181
  C          0.22826029906837      2.76561505380720      0.66477545298545
  C          0.81548476626236      5.17465847282349      0.96235800014635
  C          3.11531585743996      5.26284068578997      1.42041654228391
  C          1.49840931862554      5.94995922543256     -0.03844189112485
  C          2.95967129034228      5.80456215681372      0.15958916219769
  C          3.47583308733018      5.11359863848212     -1.00336356201885
  N          0.07595172103314      1.88856531653683      3.09810342962066
  H         -0.48152734383126      1.12605864327623      2.76977331933393
  H         -0.53804069820017      2.61166232642165      3.41455882816773
```

```
Structure: C30NH2_c.xyz
33
Coordinates from ORCA-job 30_3 E -1198.569042623011
  C          1.20647513205280      1.54569215349284      2.03300247952767
  C          1.87000054609496      1.43007326370739     -0.99030621964806
  C          0.57274661334746      2.81082430357479      2.31623041923572
  C          3.36715445242271      2.67795839612211      1.87303753755572
  C          2.59030631826811      2.23603592333560     -1.97175241829603
  C          0.52733928688260      1.98424472181238     -0.85726821728633
  C          0.53841804561919      3.23811966806873     -1.51549089040816
  C          2.47217705902980      1.57515312741908      1.52870384351889
  C         -0.19869978558368      1.90578655600944      0.33214647142562
  C          2.78949459852503      3.89088472958874      2.33831117748626
  C          3.90264039640258      2.45898402591996     -1.46476837410587
  C          3.99094138714617      1.76050163965226     -0.20066273487726
  C         -0.04584891137366      4.34300427640154      0.60575519776837
  C          1.33820893890566      5.35493335204924     -1.19639318058579
  C          1.33470687357146      3.92197331320642      2.47067422472705
  C          4.35073239749679      2.72515214561560      0.82421317117618
  C          2.77750410717063      1.14904434289903      0.12348123719469
  C          4.36890333208548      3.76803305454988     -1.26527797897386
  C          3.16376658539227      5.11753551111918      1.70316445988461
  C          0.83845253492833      4.98923885865878      1.56748284111796
  C          0.30007378625353      4.45087951895208     -0.78935899312332
  C          4.68692423474138      3.92269326855800      0.19680832902267
  C          2.25618699262174      4.66192594793073     -2.13589775595560
  C          1.78920408749237      3.36080775623867     -2.28117620012670
  C         -0.30797794893510      3.05800809426190      1.11905117982756
  C          1.91172553072653      5.72947272677958      1.16808342009172
  C          4.08147664734670      5.12363914696482      0.64620850101553
  C          2.12566474870063      6.03324468916019     -0.22795715712947
  C          3.48241826487028      5.77178486660840     -0.51297132264168
  C          3.57931643515204      4.87284099505094     -1.64078708297211
  N          0.23174468421075      0.79753372163361      1.22611969879260
  H         -0.52168466935435      0.42929370963491      1.77086137940514
  H          0.66119732954652      0.05551220146290      0.71136296454807
```

```
Structure: C30NH2_d.xyz
33
Coordinates from ORCA-job 30_4 E -1198.562473344608
  C          0.63757902376870      1.44129709197842      1.44752945234606
  C          1.96270197983389      1.55350537086969     -1.15297916383755
  C          0.37636510624889      2.83301033086644      2.07579260670622
  C          2.65516567267227      2.48578335803021      2.22870940032138
  C          2.73159755747889      2.44379935029886     -2.06573325939681
  C          0.63934462441693      2.11535702783575     -1.02454867283128
  C          0.62630831388959      3.33325460950301     -1.76141425268378
  C          2.16427617195705      1.50122848410380      1.32616649026270
  C          0.00497020264194      2.09745652656917      0.21829794336267
```

```
  C          3.77752670231480      3.23696215226816      1.92891495084960
  C          3.98090347900934      2.70765095304814     -1.50220852484711
  C          4.01335214022204      2.04245791927417     -0.16878897701406
  C          0.07670893380650      4.56304535536173      0.33546925458192
  C          1.26968776569624      5.49807999947752     -1.43646342488779
  C          1.52475420638677      3.43057749494478      2.47832130314395
  C          4.54361276845378      2.93029679757535      0.77837138765439
  C          2.77772642718252      1.36755002002500      0.06820855822701
  C          4.40546161535898      4.05149176637418     -1.31954401584982
  C          3.36686881237361      4.66719249369109      1.81756031646126
  C          1.94191914304038      4.72966956273758      1.97648822422580
  C          0.28362204415977      4.56587150542303     -1.10794708256110
  C          4.75979778078497      4.18023825598765      0.07399988610244
  C          2.31130785049997      4.85949613792487     -2.25637460293982
  C          1.90210949167643      3.51279656395298     -2.46314007883384
  C         -0.26908794030084      3.33274986823837      0.90288742755191
  C          1.16950427592653      5.32417792133059      0.92866813927686
  C          4.01800503240078      5.22383201728589      0.70391640906527
  C          1.87748302790360      5.96137646739823     -0.19074903125620
  C          3.23857074054587      5.84082865830239     -0.32488770351258
  C          3.53229256658716      5.11404431809692     -1.63814483638714
  N          0.11135944722153      0.14118592052505      1.88764750923447
  H         -0.88460672629205      0.17804687098974      1.96945611145630
  H          0.50450177272377     -0.11350115326553      2.77114826849224


Structure: C30NH2_e.xyz
33
Coordinates from ORCA-job 30_5 E -1198.551619116160
  C          0.29975011601487      2.02548231022076      1.05008728310488
  C          3.43607022699604      1.21252370016221     -0.54703570498627
  C          1.66301261127635      1.27409104533960      1.01272307487729
  C          3.48103237728833      2.09390310342839     -1.64841614874477
  C          2.19504977289594      5.87680501895877     -0.25357100276153
  C          1.66922891363470      3.67265025829983      2.50627916804314
  C          2.09613598439232      2.40780305289016     -2.07702261886003
  C          3.97151462773095      1.63580684873375      0.68733885901273
  C          0.50699081283263      3.29432129819344      1.87004587825760
  C          2.89581114292146      2.95979961115205      2.31883929385589
  C          3.25738410041227      4.39952501953438     -1.93530353713265
  C          3.99129503705759      3.67837900497766      1.80623021086803
  C         -0.07210083324151      3.72696608793114     -0.85559466052230
  C          0.20068218514299      4.65535637590459      1.40031381051884
  C          0.20559600912217      2.44847441092986     -0.46874680766034
  C          4.66960908948264      2.84601852942488      0.77808947478865
  C          2.84631802349385      1.68461207762985      1.66112752591930
  C          4.18918293386954      3.30650248564566     -1.56886833770729
  C          1.97902828645133      3.83304055611798     -2.25539917071338
  C          1.50117456626679      5.05964023607448      1.95123146816100
  C          3.35029414043739      5.41565479264833     -0.96439420108493
  C          4.83439886181696      3.65641501708701     -0.36297720642097
  C         -0.04321314360050      4.86009026118042      0.09644870174378
  C          1.22194077315970      1.75279082818323     -1.19731392409451
  C          0.84323493132313      4.45560897333149     -1.74165492439822
  C          2.47837655453896      5.62971170525348      1.20089582842037
  C          0.95105089064749      5.52051846509285     -0.75635958926117
  C          2.01212889844044      0.95505184156519     -0.29872646026088
  C          3.80386350648410      5.00217949609866      1.25881189294888
  C          4.35728997723407      4.99813825335030     -0.01278288630678
  N         -0.90037671676021      1.31000445894221      1.50691004355103
  H         -0.77552190892597      0.96962978439589      2.43887375193139
```

```
  H          -1.08454274353797      0.53331509246946      0.90455093268859


Structure: C35NH2_a.xyz
38
Coordinates from ORCA-job 35_1 E -1389.133164648125
  C           0.07570938222126      2.77135115542996      2.40704673091197
  C          -0.91040618578588      3.92317266014046     -0.96117901151079
  C           0.21452927493348      1.99286767897212     -1.57903749950500
  C           3.45071931161704      2.47712413530941      1.17163161763668
  C           0.90931402588818      1.42279214945548      0.57289542720092
  C           3.19691678895030      1.82015556477136     -0.03597367583712
  C          -0.56370403319313      3.63452978163686      1.47016926854717
  C           1.56263327007497      1.70691747698779     -1.65210015198928
  C           0.61939559689145      4.16718144775358     -2.64347380421423
  C          -0.24659744026785      3.26179007582668     -2.05818068481801
  C           2.38791696988020      4.17462518278901      2.32222985662863
  C           4.07516537075877      3.92569053486585     -0.62113699838305
  C           1.99030800081479      1.21953947745911     -0.32740720924887
  C           1.14269583532469      2.01519078027133      1.86011144202745
  C          -0.47097656547195      5.28527062780074     -0.90109043554908
  C           0.36816417821500      5.50290451593281     -2.06156156814184
  C           1.59541112220441      6.11051618680473     -1.89285474245536
  C           2.65901891819556      5.12874013641324     -2.21302699081985
  C          -0.84071098002868      3.07744601831142      0.16581654251027
  C           1.10664932773879      6.65393304337820      0.37657794703698
  C           2.38819056350833      2.70584393332507      2.06162576803055
  C           3.57005724673880      4.74053232855363      1.64953674718502
  C           3.27572902360646      6.25656105130113     -0.28148897818265
  C           2.51597863735754      2.68648147452705     -2.12509532937613
  C           0.01560505089135      4.91640435783754      1.50563197473897
  C           1.95290667575969      6.79299179282691     -0.72057487268722
  C          -0.22509701729485      1.84228988849229     -0.19234345579488
  C           3.62213328242617      5.17850309919486     -1.17394676606417
  C           2.04025940855984      3.92094093916576     -2.65689175941575
  C           3.56003334028245      2.73657146324560     -1.15423306105254
  C          -0.04726700731631      5.80308507193183      0.34574695194813
  C           1.90810860883273      6.29858180945446      1.52753152939972
  C           1.34708590840937      5.12130692249012      2.14287066911363
  C           3.22518768649161      6.03540636842312      1.10527583282603
  C           4.06465393084585      3.77120508587239      0.82831954962353
  N          -0.03565203352155      3.00340990806013      3.85433639621399
  H          -0.50844094190429      3.83766151421025      4.13805263049770
  H           0.72168547569661      2.67158435648810      4.41677012235875


Structure: C35NH2_b.xyz
38
Coordinates from ORCA-job 35_2 E -1389.116264119857
  C           0.38408127942652      1.89170718585114      2.22013574609139
  C          -0.86144054554416      4.12640445930004     -1.04729436454191
  C           0.05323027664273      2.02078528630010     -1.35930382873674
  C           2.85498956444134      3.00014090308744      1.68909654185791
  C           0.28424164488187      1.40635469386296      0.90065157213902
  C           2.68555450985136      2.05290891400526      0.64209737445961
  C          -0.31336156619817      4.12351729166591      1.36561357653288
  C           1.35474768507404      1.63237983429049     -1.20312892142009
  C           0.73567827159216      3.95083028775385     -2.67396631714296
  C          -0.24637137110160      3.23902414621367     -2.03794607474155
  C           2.14950192096509      5.04449625043415      2.47837493364920
  C           3.90672358076588      3.88185394793727     -0.25642789907760
  C           1.55208802572505      1.32371227891280      0.23587211621915
```

C	0.45272453470013	3.28807257107855	2.29740932122487
C	-0.33779648361619	5.44520872078780	-1.21667387617709
C	0.57761215754335	5.38501008192660	-2.33126413125733
C	1.83696865032369	5.93746328570684	-2.21107221997487
C	2.81328602678989	4.82885941028570	-2.22917120653350
C	-0.89569728054220	3.45092743633674	0.20799194360142
C	1.22568891392785	6.97942098530896	-0.11079868479617
C	1.83426820564916	3.67980562556360	2.48902440390610
C	3.38857111762988	5.26364808037609	1.71136553844748
C	3.37092040657049	6.29246902929787	-0.51810447215800
C	2.43435704064854	2.48149623703773	-1.67769985883697
C	0.05966539786488	5.46962221981330	1.22754111343936
C	2.16347383371947	6.80784625085077	-1.15883879708351
C	-0.54054447886390	2.08467660719511	-0.01941820661287
C	3.67828933295831	5.01635093706533	-1.11338246891004
C	2.13747712848873	3.60717594668703	-2.49442996721663
C	3.30711379517288	2.66001660443156	-0.58230043498418
C	0.04520770397902	6.15730504511030	-0.06505517813484
C	1.89762061853421	6.94566627045540	1.15669306584013
C	1.23186191626901	5.96521401143272	1.94541154313987
C	3.19620409740567	6.41003181400618	0.88066211913360
C	3.74866589631473	4.05318957014221	1.17392828229100
N	0.91694692304250	1.03854055281969	3.29207833178060
H	1.37141036281097	1.50778761359550	4.04922440336586
H	1.25935090390452	0.14117961407544	3.01368498531590

Structure: C35NH2_c.xyz
38
Coordinates from ORCA-job 35_3 E -1389.113954362601

C	1.15997875132079	0.67913633700938	0.54336474761758
C	-1.01052414946159	4.42616016225360	-0.37639075156168
C	-0.21358754039257	2.17675673656049	-0.38818409142590
C	3.26429447586407	3.33697594466941	1.91359433766632
C	0.63010091588007	2.46747641950440	1.90825695112930
C	2.87764030651393	2.24893253642858	1.17038825764359
C	-0.01997264331041	4.74992906409132	1.82741431004643
C	1.00784369307889	1.41175531298882	-0.65717285629580
C	0.35010333132637	3.78578318416697	-2.09904697257374
C	-0.56112619620373	3.29371998656969	-1.22011376619490
C	2.64574035565629	5.51099009531297	2.35113646290338
C	3.92739399449407	3.77836164935864	-0.28497719817186
C	1.68291832454105	1.54723857847789	1.52849871826216
C	0.92158949987041	3.78377296441510	2.42032699384860
C	-0.40171260088865	5.64356330232145	-0.85685130672982
C	0.34569062283900	5.25968102027228	-2.03681613153501
C	1.64823002308777	5.67895397818620	-2.20873794229063
C	2.52711278097243	4.48056915927332	-2.13706365984245
C	-0.84833812427098	4.00716677431436	0.91954996636836
C	1.45012081494195	7.16873124629865	-0.31415623033191
C	2.27211351271176	4.14902773480968	2.58160356492037
C	3.81844552109438	5.53081091299761	1.43535748748273
C	3.50242472317292	6.14992114442830	-0.86593697726734
C	2.08860555069600	2.28915924847901	-1.12140415109202
C	0.39892827869172	6.02634864323852	1.45452554578199
C	2.19635722542997	6.67007394768799	-1.39467697741671
C	-0.43201097160614	2.59265122242599	0.94558367427153
C	3.61742006015755	4.76046570511125	-1.26040703357348
C	1.74969524416501	3.33188785594855	-2.05316471974669
C	3.17354263715715	2.53694136461152	-0.24234075182310
C	0.21170512293489	6.49875852076924	0.06917709411865
C	2.32895480842842	7.19303442915577	0.80730613382553

C	1.72903274376033	6.43964022283537	1.86442088972256
C	3.60144205244128	6.53456325174374	0.45505320301525
C	4.06831768024951	4.22607958101610	1.07358525422829
N	0.30445219042423	-0.46908072482919	0.87588826164607
H	0.08910910068003	-0.59047452309735	1.84485208723058
H	-0.38872211340876	-0.71436299938709	0.19813758024229

Structure: C35NH2_d.xyz
38
Coordinates from ORCA-job 35_4 E -1389.115447116528

C	0.34181217071544	1.90651994409715	2.21716070544452
C	-0.89847679442548	4.20584480061609	-1.01455266954506
C	-0.02044848982611	2.08211681515435	-1.32187468550375
C	2.84642056350844	2.94131565279878	1.68285338506046
C	0.22558163202179	1.46360749069308	0.89050394696848
C	2.63799562734640	2.01023537402224	0.62604265302694
C	-0.29934518128231	4.16644360253089	1.37796391297130
C	1.28633706535590	1.64909382458984	-1.19153094800919
C	0.70212470666156	4.00395150366990	-2.64246097950665
C	-0.29793191067263	3.31303312337663	-1.98747469286405
C	2.21921937641532	5.01009075627971	2.45252525693683
C	3.89947279541877	3.82443930207584	-0.27022938609153
C	1.50367292258313	1.28745713767089	0.22198053169444
C	0.45809424438136	3.30141474752461	2.29577783308869
C	-0.32363675151441	5.50812253825957	-1.18927345831018
C	0.57633498369947	5.43313927871958	-2.32159240940662
C	1.85468891588062	5.94646677094677	-2.20277242134498
C	2.81037161665511	4.81137875062821	-2.22683549840101
C	-0.92612601070977	3.52247304036773	0.22959249818241
C	1.30061458239278	6.99445747992861	-0.12698468873227
C	1.85863346135745	3.63758774618909	2.45595737037322
C	3.48439991183106	5.20738871439757	1.72201117584762
C	3.45078010303564	6.25295176895411	-0.52076584381631
C	2.36596262909843	2.47326826723369	-1.68017703568974
C	0.12297525259895	5.50064333010349	1.24243336439348
C	2.21459591395910	6.80969500994692	-1.16562868946907
C	-0.61301916521971	2.13175343760107	-0.00810874096288
C	3.69359399283198	4.96742627938703	-1.12504138620110
C	2.08823490757731	3.61205089697372	-2.48725125391235
C	3.26134920378556	2.62694955531601	-0.59233982267758
C	0.09670970999049	6.20712020884265	-0.04764847386395
C	2.02023815985189	6.87160369847575	1.12109575159862
C	1.31491643504756	5.93626122287011	1.95470691009037
C	3.32786526671452	6.37531706724536	0.87155726031589
C	3.77223743670155	3.99797016615898	1.16756670504881
N	0.84833918017438	1.01868108214900	3.27360405403532
H	1.16669039251477	0.12142978998566	2.96767496163155
H	1.34203114129134	1.46139979347074	4.02211483286489

Structure: C35NH2_e.xyz
38
Coordinates from ORCA-job 35_5 E -1389.076283500400

C	-1.06159176577987	2.06805735834251	1.78750069959517
C	-0.75918774077183	4.02038236752010	0.28116507826779
C	0.10569133185808	1.90389068569431	-0.50305244556050
C	4.02774711380652	2.84867389333405	0.99764398893096
C	1.48236509276985	1.78814983896692	1.41551147772201
C	3.48223260944563	1.98492878487791	0.07426664585347
C	0.79485783774744	3.89926175647686	2.20422728155998
C	1.39465640282352	1.65740750549621	-0.90185580355522

```
  C          1.47756498510102      3.67897628033254     -2.30090063694609
  C         -0.47754494087092      3.16431196083864     -0.87232550645431
  C          3.49857262909270      4.75178466434577      2.14849533550908
  C          4.09365304997682      3.93018980396740     -1.11889617092625
  C          2.23496423039373      1.41006043046617      0.30755473913037
  C          1.88780871197391      2.90057655440153      2.20990000791397
  C         -0.30971720258190      5.34728619789218     -0.02365735189307
  C          0.07890215113110      5.35508567323938     -1.40627244720178
  C          1.26527378851649      5.97024417123577     -1.79669369048305
  C          2.17542583886839      4.90836333578374     -2.28702898102554
  C         -0.41275141607770      3.37449398110545      1.51995645055976
  C          1.53516558023309      6.87722333090748      0.41826772351265
  C          3.23265740570685      3.34402224453077      2.08650259046864
  C          4.37728421273946      5.13453957832523      1.01156822083446
  C          3.37972069640994      6.29795644436874     -0.89159127877025
  C          2.14070288885052      2.52479667123824     -1.78619743052598
  C          1.08028232310372      5.23661700839061      2.04195067006145
  C          1.96578678671809      6.78787052569293     -0.92422794319456
  C          0.05778403332755      1.78521113926362      0.98809027027963
  C          3.44806737731979      5.08126535484440     -1.67478140099885
  C          0.12849609004956      3.95964521552105     -1.83449144707862
  C          3.43829392681522      2.68476201619367     -1.23578606895644
  C          0.49586076734571      6.02847304324238      0.91118119974715
  C          2.70604490719038      6.71622769982459      1.24110649442764
  C          2.45655421382038      5.66936406882357      2.17854633010677
  C          3.82830276038836      6.32712838513975      0.41819932280272
  C          4.57271584093973      4.01309570711068      0.25992425479071
  N         -2.44113080188935      1.66551149240422      1.47809988675386
  H         -3.11953543586114      2.39149273304155      1.59087571172632
  H         -2.54866628288314      1.10377211369657      0.65780421653092
```

```
Structure: C5NH2_a.xyz
8
 Energy:      77.8312100
C         -2.05158        2.33875        0.44305
C          0.24301        1.70418       -0.59902
C         -2.75108        2.93270        1.20192
C         -0.50608        2.48317       -1.44728
C         -1.22934        1.69942       -0.51092
N         -0.28149        3.33186       -2.33782
H          0.67036        3.54202       -2.64196
H         -1.03873        3.83927       -2.79648
```

```
Structure: C5NH2_b.xyz
8
Coordinates from ORCA-job 5_3 E -246.208724205615
  C         -2.15416143267672      1.09728777791488      2.16502824036121
  C         -1.29678587022084      2.82051401314188     -0.79270162852279
  C         -2.28113115896359      2.79811812705506      0.13861835006658
  C         -0.32804309113336      2.75341404435637     -1.59614342711239
  C         -2.22967799984857      1.92969111613484      1.20237025131814
  N          0.65638772991607      2.61825001623641     -2.32621441783403
  H         -3.12456980874564      3.47771061566280      0.04202902086447
  H          0.67819714963523      3.22359014935383     -3.12892662316575
```

```
Structure: C5NH2_c.xyz
8
Coordinates from ORCA-job 5_5 E -246.202228125545
  C         -2.75329000583697      2.55738217498226      1.01149596757158
```

```
  C          -0.89756096390984      2.60673229345560     -0.80542060195499
  C          -3.08265199654816      3.15354217642616      2.13279302000378
  C          -0.04257555379189      2.67599994179891     -1.80249540106100
  C          -1.79794923776529      2.63811219894668      0.02659457736350
  N           0.95448049311663      2.41963269166275     -2.45059106690808
  H          -3.54414600200809      1.78920675084137      0.93091819956650
  H           1.08390877941443      2.87796764232584     -3.33923492860583


Structure: C6NH2_a.xyz
9
Coordinates from ORCA-job 6_2 E -284.247734705896
  C           5.13093621205245      1.87171065524869      0.32096346021672
  C           6.35326879906828      1.40269112684177     -0.07688492154546
  C           5.10958563934697      0.99823883815071     -0.71445937012742
  C           4.45427526663396      0.29172014524140     -1.63344740068435
  C           3.13367931319850     -1.05559189964628     -3.36804721280833
  C           3.81309272927837     -0.37354461998526     -2.49569458617792
  N           4.30029107224283      2.77016699650387      1.13564429308782
  H           4.75094330017095      3.01268412367961      1.99477059831876
  H           3.35672766610605      2.45350463926984      1.23266514631256


Structure: C6NH2_b.xyz
9
Coordinates from ORCA-job 6_3 E -284.226162757891
  C           4.47251876634574      2.07742573110452      0.42029376214105
  C           4.30729875791390      1.42821431065226     -0.57805214374495
  C           5.57692224492394     -1.36266779258338     -2.29090493406377
  C           3.47172061007529      0.32221463338402     -2.86388679169373
  C           4.09196738874829      0.67331687871543     -1.71605852585740
  C           4.63008319479422     -0.45290867017556     -2.37725322010944
  N           4.74483855429123      2.92842369212799      1.58757453740713
  H           5.20659067837954      2.40215271162056      2.30159402371378
  H           3.90085980791795      3.35540851045849      1.91220328821616


Structure: C6NH2_c.xyz
9
Coordinates from ORCA-job 6_4 E -284.242595291655
  C           4.47372898305083      2.10680410424372      0.50882509048370
  C           4.52288649368301      1.34889437787809     -0.43068645662944
  C           4.62152725873558     -1.13885635085518     -3.27472312899049
  C           5.31250052434994     -0.44333912096893     -2.23098616372954
  C           4.55929067044636      0.44448055336657     -1.45106518240736
  C           3.86652555109241     -0.22506008674154     -2.47026559488535
  N           4.45181289907448      3.13739331074182      1.55682583572843
  H           5.07694088832815      2.88364708180449      2.29495038091147
  H           3.51758673462937      3.25761613054353      1.89263522081920


Structure: C7NH2_a.xyz
10
Coordinates from ORCA-job 7_2 E -322.363330960903
  C           5.65213621616763     -0.99485104369561     -0.58435618770248
  C           4.44071825041152     -0.70989765889727     -0.44887626089462
  C           3.42987237464015      0.04980255169099     -0.02670330838726
  C           3.81550939281174      1.17049189394453      0.64849487244435
  C           5.13652969162305      1.30156243857419      0.76287455309493
  C           6.32271693981990      0.93943259520303      0.58039185954211
  C           6.76694391043944     -0.21923491436465     -0.09443849735999
  N           8.10124626239113     -0.47501111433839     -0.20952937132953
```

```
  H           8.42833718471508     -1.29091678448754     -0.68629918089723
  H           8.74949977883294      0.17378203170562      0.18901151550679


Structure: C8NH2_a.xyz
11
Coordinates from ORCA-job 8_1 E -360.442497800699
  C          13.96926467651099      0.35116154444583      9.05878391344689
  C           8.84367922627350      1.10572018212374      5.38958780458343
  C          10.91421675805215      0.78878780929590      6.87480492876502
  C           9.84283687382389      0.95008849133043      6.10432334795854
  C          12.98724687803110      0.48320594369684      8.36776902806277
  C          11.89959748729275      0.64038319105551      7.58387054302637
  C           7.78366786405487      1.27246738258561      4.62904744931552
  C           6.75501325650254      1.43589508832390      3.89380123348331
  N          15.09728018068220      0.16538478278140      9.98288781564263
  H          15.54397681825306      1.04004391550987     10.17112572725319
  H          15.73056998521263     -0.52514832744675      9.63344820282466


Structure: C8NH2_b.xyz
11
Coordinates from ORCA-job 8_2 E -360.414871698294
  C          12.60046859752320      0.74696956518322      8.05888302486766
  C           9.95712971936264      1.31513798087495      6.11084639387876
  C          10.96605140349600      1.99451559889582      6.71069166733202
  C           8.92380431947797      0.57218724379969      5.50668567308103
  C          10.84227294233454     -0.65872182015366      7.07069596933780
  C           9.89219404365793     -0.07366996976044      6.30005806966206
  C          11.85567230092264     -0.40226687011688      7.74450728251302
  C          11.96011932894507      1.83424735841993      7.44057219559910
  N          13.76274212374419      0.81494505241292      8.95632360131842
  H          14.34085163142310      1.61232470234563      8.78319048412009
  H          14.26604358851074     -0.04767883819888      9.00699563265248


Structure: C8NH2_c.xyz
11
Coordinates from ORCA-job 8_3 E -360.402361659708
  C          13.82926712298038      0.00994685070780      7.53450604253573
  C           6.58920010779076      2.38048691616255      6.73590896387498
  C           7.80492319889097      1.93829495315465      6.74895194621207
  C           8.99778679621742      1.50436428487213      6.77217558054269
  C          11.38521140142583      0.63895203328793      6.84059234396129
  C          10.22471146520278      1.05720302532268      6.80165413080871
  C          12.66024591611471      0.17353018186332      6.89695467304816
  C          13.74053000749002     -0.52883923108831      6.26461022683673
  N          14.63612988977594      0.33613211537662      8.71918982040664
  H          14.09148814794009      0.48785610852608      9.54402022649186
  H          15.40785594556909     -0.28993724506670      8.83088604493535


Structure: C8NH2_d.xyz
11
Coordinates from ORCA-job 8_4 E -360.379757125804
  C          12.30478432889752      0.58144961611198      7.62122719817143
  C          10.46643767391050      0.33989016541790      5.98913441101423
  C          11.46931003927539     -0.07820009083811      5.04714855778248
  C           9.60138918325888      0.87764547121620      6.86414720387735
  C          10.70854678989633      2.54290967423620      8.78902979499272
  C          10.08779783517879      1.64480986629718      7.85323130468010
  C          11.42263128592344      1.47295941334559      8.25429841662518
```

```
  C          11.80364892584602      0.15915529240156      6.37840751812566
  N          13.48117210603537     -0.01131593857923      8.27363872462605
  H          13.87412026905557      0.56569687446651      8.98963310352160
  H          14.14751156212020     -0.38701035095702      7.62955376623732


Structure: C8NH2_e.xyz
11
Coordinates from ORCA-job 8_5 E -360.370953308376
  C          12.86232165414329     -0.05937456233319      7.79354606054788
  C           7.49608890324240      3.04564900021001      5.84686075302928
  C          13.22231565573397     -1.21709256434045      7.19972884206624
  C          13.55528878154678     -2.27779839741784      6.60150548062001
  C          10.69579564839688      1.19648139765433      7.01398479036832
  C           8.56760486635576      2.42158014021787      6.23317609713000
  C           9.61045639119316      1.81715591771171      6.61349471039617
  C          11.71030601626290      0.61673187905057      7.39196494473707
  N          13.60721550636911      0.51932239775376      8.92099582979744
  H          13.51269969186062      1.50562743721900      9.05615915982154
  H          14.52725688958492      0.13970736326830      9.01803332055669


Structure: C9NH2_a.xyz
12
Coordinates from ORCA-job 9_1 E -398.498797588594
  C           4.10808559294433      1.42103214480057     -0.18518068201834
  C           5.25460039988386      1.68663598276966     -0.95075023031998
  C           4.81954302517121     -0.76010942433152      0.87805919922021
  C           5.96672520135904     -1.51422478446225      0.80147605525863
  C           7.03832916468819     -1.43435486536944      0.21936576640446
  C           4.17561796990440      0.22389295234750      0.54519540389920
  C           7.66783821657245     -0.58155460095856     -0.65595130412529
  C           6.38140231066827      1.35648973492830     -1.29007889491223
  C           7.43155386854735      0.47102775844131     -1.23026050591285
  N           2.91380488448157      2.27780038882110     -0.20845664193864
  H           3.09986749727946      3.22408014482352     -0.47290951741697
  H           2.32681185868785      2.17601455730527      0.59471135295539


Structure: C9NH2_b.xyz
12
Coordinates from ORCA-job 9_3 E -398.456332297699
  C           4.11731993703841      1.78209018679212     -0.41664795960343
  C           5.25745623656496      2.05751755640404     -1.13437378812265
  C           7.03126421602539     -1.93058576462267      0.51407235262884
  C           7.63772138298858     -2.95532195383267      0.92240931020581
  C           6.38837487135658     -0.81870908545610      0.11062228733697
  C           4.24216516896006      0.55499524755153      0.26808297793613
  C           5.08265843696983     -0.32476284372515      0.41461454905927
  C           6.33728593901109      1.31447681007831     -1.18883971902058
  C           6.81012894535520      0.23898267728700     -0.75402388860494
  N           2.89738144568975      2.60224134886022     -0.41811546185519
  H           3.05371139123519      3.55835735358551     -0.66591203178176
  H           2.32871202957648      2.46744848265218      0.39333137820689


Structure: C9NH2_c.xyz
12
Coordinates from ORCA-job 9_4 E -398.462220442017
  C           4.32413056354933      1.45499880645647     -0.14562719786305
  C           5.40837961508382      1.86732304514025     -0.95488959513318
  C           4.36999698725015     -1.02348311424831      1.24510170625480
```

```
  C          5.51415257539920     -0.72558652183657      0.49418115456582
  C          6.80254176316061     -0.76106415193065     -0.16080642084246
  C          4.54085869250314      0.20061209923197      0.47621547225525
  C          8.07854439069142     -0.96140957224738     -0.68954907772919
  C          6.47484152461831      1.28994417420237     -1.20055194149923
  C          7.28229304000496      0.21022441326522     -0.98556112689503
  N          3.12177300109593      2.26428890197867      0.09990454790384
  H          2.93486576552677      2.96911716988632     -0.58440736482319
  H          2.33180208188780      1.76176474980062      0.45120985548275
```

```
Structure: C9NH2_d.xyz
12
Coordinates from ORCA-job 9_5 E -398.450718596329
  C          4.07414448661632      1.25231295520456     -0.08611428962081
  C          3.23190914523443      0.49089389209599      0.69729869666429
  C          4.67657260254809     -1.34430150508670      1.10094709763257
  C          5.60064623041334     -0.65476625173582      0.31993020318857
  C          7.80570119992140      0.07708607216936     -0.98464720310506
  C          3.57440071276086     -0.64419313032561      1.18607469943339
  C          8.95179432213564      0.16444009894094     -1.49882095002684
  C          5.30363288551508      0.56486417000735     -0.23613336511227
  C          6.58794844117076      0.00776779676918     -0.45484389878794
  N          3.75813345256600      2.52422378813074     -0.75190694827817
  H          4.57314651296254      3.06297476829222     -0.96521679007650
  H          3.04615000892705      3.04542734523679     -0.28134725610941
```

S4 C_nCONH_2 family: Energies (Hartrees) and xyz coordinates (Angstroms)

```
C6ONH2_a -359,56024
C6ONH2_b -359,55452
C6ONH2_c -359,52447
C6ONH2_d -359,47101
C6ONH2_e -359,44834
C8ONH2_a -435,72288
C8ONH2_b -435,70537
C8ONH2_c -435,67035
C8ONH2_d -435,62091
C8ONH2_e -435,62259
C9ONH2_a -473,75867
C9ONH2_b -473,68784
C9ONH2_c -473,74702
C9ONH2_d -473,74071
C10ONH2_a -511,87238
C10ONH2_b -511,83060
C11ONH2_a -549,97353
C11ONH2_b -549,94828
C11ONH2_c -549,93805
C11ONH2_d -549,89832
C11ONH2_e -549,88915
C16ONH2_a -740,39228
C16ONH2_b -740,36165
C16ONH2_c -740,39227
C16ONH2_d -740,34769
C16ONH2_e -740,32866
C21ONH2_a -930,82709
C21ONH2_b -930,86375
C21ONH2_c -930,83213
C21ONH2_d -930,78628
C21ONH2_e -930,75716
C26ONH2_a -1121,29170
C26ONH2_b -1121,28821
C26ONH2_c -1121,26817
C26ONH2_d -1121,25737
C26ONH2_e -1121,23008
C31ONH2_a -1311,96015
C31ONH2_b -1311,94263
C31ONH2_c -1311,93344
C31ONH2_d -1311,91623
C31ONH2_e -1311,90981
C36ONH2_a -1502,51488
C36ONH2_b -1502,49471
C36ONH2_c -1502,48475
C36ONH2_d -1502,47030
C36ONH2_e -1502,46371

C  -37,84496
N  -54,58957
O  -75,07371
H  -0,50211

Structure: C10ONH2_a.xyz
14
Coordinates from ORCA-job 9_1 E -511.872375430126
  C          -0.05651235936141      0.05672942417351      1.92554820491411
  C           1.11410283282237      0.05479008748808      1.63128370518916
```

C	2.42780621022194	0.05800680209207	1.29982408813745
C	3.61089696910726	0.06440406522569	1.00354500984119
C	4.90372749263270	0.07215618685707	0.67932777816749
C	6.10495616302430	0.08071484645136	0.38047625865295
C	7.37010710165510	0.09037868630969	0.06481874595468
C	8.60364697999587	0.10033356696733	-0.24169128712762
C	9.85852921524255	0.11371070392823	-0.55356567377675
O	-2.13012569499580	-0.99466663146513	2.28165503636469
C	-1.47899629044246	0.01324457374815	2.29371737591871
N	-2.02131505178185	1.34148153980050	2.61399370659252
H	-2.89942263620144	1.31258907710467	3.09158379404627
H	-1.36469093191766	2.03273707131844	2.91567325712315

Structure: C10ONH2_b.xyz
14
Coordinates from ORCA-job 9_3 E -511.830598472622

C	0.72983620130970	0.33319811736942	2.33192105434298
C	1.29056439634200	0.06689896696932	3.56927571824145
C	2.05501225622623	0.17038782336921	2.35112067165946
C	3.26592496358063	0.13124222278536	1.72803783218614
C	4.35927732361038	0.09858883384235	1.17876365221793
C	5.54371479136755	0.05920120556152	0.58554916404258
C	6.65336123902618	0.02065911373009	0.03285768111192
C	7.82415373489150	-0.02158570614893	-0.55402275432574
C	8.97005205893463	-0.06563605244705	-1.11894943572093
O	-0.45836530962909	0.52595719761038	0.30412484190055
C	-0.46006604589111	0.57437321192068	1.50314652483980
N	-1.62589556448581	0.92591771162855	2.32665001795744
H	-1.59703981857533	0.66727182480614	3.29219113412378
H	-2.50782021956482	0.91013553553326	1.85552389733645

Structure: C11ONH2_a.xyz
15
Coordinates from ORCA-job 10_1 E -549.973526335966

C	-1.07449515095523	0.10367539987687	2.25902729020029
C	-1.13757604235956	1.21923683177393	1.44661646932559
C	-1.24422710746387	1.77175225199433	0.33937530943205
C	-1.37861715123559	1.84956000912819	-0.97001806999375
C	-1.50183491436367	1.10122743866290	-1.98011891587042
C	-1.57586310017900	-0.10345283633695	-2.46203932168893
C	-1.53834168304776	-1.26121794789394	-1.87883964967493
C	-1.43543327515886	-1.89790305556005	-0.79004966839164
C	-1.29611026211652	-1.73946153774593	0.50770808881676
C	-1.17298720506253	-1.07603928984119	1.55152571222785
O	-0.87430971853112	-0.86938152123123	4.39255362871989
C	-0.91153376610191	0.12362291474910	3.71983038732723
N	-0.85963483718739	1.47255321347208	4.30171831059356
H	-0.51895742094698	1.48482711480286	5.24181843571844
H	-0.55736836023525	2.21758100574808	3.70710199607031

Structure: C11ONH2_b.xyz
15
Coordinates from ORCA-job 10_2 E -549.948279250826

C	-0.99614273344165	-0.03664243529512	2.29504702454860
C	-1.04005263740681	0.94200554323718	1.33423575940451
C	-1.14713454604898	1.43980246731179	0.19981649439953
C	-1.31549873311797	1.48483113017942	-1.11221501592717
C	-1.48647353393597	1.57490228229909	-2.46566112412667
C	-1.54007621584717	0.29750930181619	-2.04786138767437

```
  C          -1.52654875779310     -1.69959029370340     -0.66706902808730
  C          -1.60146924802902     -0.98825093086751     -1.68019766558458
  C          -1.37608738192163     -1.93826151064080      0.63670895287224
  C          -1.18369852195017     -1.24545144328173      1.63850636311574
  O          -0.73187773750426     -0.83154182118065      4.49169910613997
  C          -0.79318293266975      0.10508419684983      3.74405388646818
  N          -0.60530912313766      1.49177848027003      4.19425649123894
  H          -0.70353284128532      1.59245600258675      5.18431514280628
  H          -1.03020506143932      2.20794901143443      3.64057498734308


Structure: C11ONH2_c.xyz
15
Coordinates from ORCA-job 10_3 E -549.938048526047
  C          -1.08170351466458      0.30272185141569      2.00565645349985
  C          -1.17486929937156     -1.03705037037642      1.65230386469584
  C          -1.55791238946430     -0.92693580854217     -1.67598848538978
  C          -1.16858681661669      1.18073278916375      0.95359887410367
  C          -1.20535208197031      2.33007459422423      0.21225594854299
  C          -1.33663549645498      1.21832287777526     -0.53411530996841
  C          -1.48764362423232      0.29979411864334     -1.51129311618805
  C          -1.57900344206481     -2.25146820255963     -1.42883863268373
  C          -1.44506156722925     -2.45163480359335     -0.19730449649870
  C          -1.27868619698626     -2.12465613578929      1.07939618590830
  O          -0.84634893042451     -0.10180585057585      4.30428603095334
  C          -0.90952472014805      0.69722487974457      3.41122460490224
  N          -0.88761700898935      2.14723583922309      3.65182442714348
  H          -0.58192082642480      2.73639355668761      2.90386518069290
  H          -0.53642407461172      2.37763066144998      4.55933846780657


Structure: C11ONH2_d.xyz
15
Coordinates from ORCA-job 10_4 E -549.898324580474
  C          -1.04321175425479      0.95002587458503      3.00051676076804
  C          -1.81640292990308      0.46830174715209      0.54799954157289
  C          -1.63416656032278      1.25707794709788     -0.57114787485110
  C          -1.46677870029024      1.52456007501562     -1.80229588525722
  C          -0.90974546996825      0.63647982080743     -2.61482012195950
  C          -1.14427808492783     -0.55017180543317     -3.11586277409155
  C          -1.27127449031799     -1.38905230471953     -2.11915771554147
  C          -2.02504042757459     -1.57817693186919     -1.04298688237074
  C          -1.99203306629433     -0.74984339326228     -0.07910796609630
  C          -1.37983488920897      0.71585644638998      1.87148592681523
  O          -0.27041857821430      2.35032940054115      4.73777323491011
  C          -0.61024470729323      1.25953117738362      4.37078900532809
  N          -0.69357359884171      0.09598850441615      5.26528593905807
  H          -0.17299727857909      0.20676804410526      6.11188393907404
  H          -0.64728946424585     -0.80109460531922      4.82585486487016


Structure: C11ONH2_e.xyz
15
Coordinates from ORCA-job 10_5 E -549.889148470102
  C          -1.66475845611632      0.78002042243601      2.82696667386797
  C          -2.10482877376782      1.38079783737191      0.49511167417121
  C          -2.39188478732598      1.80804806938851     -0.61036669766355
  C          -2.14795398749347      1.36415159951630     -1.87397516947128
  C          -1.71304183430150      0.62521998042973     -2.76770553370218
  C          -1.03307193077067     -0.46582868863193     -2.30219253709716
  C          -0.51572157954242     -1.27949758774340     -1.52455707172439
  C          -0.63509291112590     -1.04132231234921     -0.16783842085592
```

```
  C          -0.88528876774003     -0.60250208346699      0.94499680381432
  C          -1.56398036532468      0.53631164020802      1.53645365817078
  O          -2.12156054907986     -0.36772181096002      4.82641707691064
  C          -1.33456972815550      0.23817582371401      4.15296811582954
  N           0.01722212883667      0.60678827496589      4.59758440099043
  H           0.69015861167466      0.74536157527554      3.87098002008094
  H           0.32708295116288      0.06857725144467      5.38136699890741
```

```
Structure: C16ONH2_a.xyz
20
Coordinates from ORCA-job 15_1 E -740.392276926999
  C          -0.60299807617412      0.26345934324922      3.16981424179675
  C          -1.02767765534345      1.33948475144663      2.39733653821968
  C          -1.44008564463032      2.07339355162326      1.51073819819935
  C          -1.91979712749885      2.59149879752343      0.36041590244591
  C          -2.33833402887594      2.69661768888177     -0.77949934625662
  C          -2.72817725235982      2.33286128848071     -2.02416180394797
  C          -2.94394874558786      1.55577839945091     -2.93603454994433
  C          -2.97552851089121      0.32625987367189     -3.50936334327748
  C          -2.79135838816881     -0.87591635518069     -3.46004267005000
  C          -2.40893614095404     -2.01555364589974     -2.83115621842818
  C          -1.98251691799365     -2.62222421626750     -1.86533498564513
  C          -1.51120091678891     -2.79954192509917     -0.60914268710951
  C          -1.15394976689637     -2.49746059804519      0.51684328696568
  C          -0.85376602724067     -1.80956277882926      1.63733513379761
  C          -0.67809048711200     -0.93744204305822      2.47516380981527
  O           0.23512433671051     -0.62104326131303      5.18068161467911
  C          -0.11588521382523      0.34063706816153      4.55461193929273
  N          -0.15583422164036      1.69198749384312      5.13180870701004
  H           0.38858366951806      1.77551500429162      5.96645372774330
  H          -0.10894288424699      2.46212156306871      4.49565250469380
```

```
Structure: C16ONH2_b.xyz
20
Coordinates from ORCA-job 15_2 E -740.361647442558
  C          -0.19963888045721     -0.17771478837640      4.10007773464163
  C          -1.48740415706588      1.24504056730692      1.07460147583497
  C          -1.88935190713120      2.07489022087989      0.27976821726439
  C          -2.37398302584961      2.64556533673472     -0.85787256579106
  C          -2.78411844989321      2.68563128333095     -2.00117686543723
  C          -3.13223565244423      2.13822928127639     -3.20307541891424
  C          -3.24569825615729      1.16100819709153     -3.91563153909345
  C          -3.11234014309479     -0.19024956181367     -4.07983592792545
  C          -2.79031844867265     -1.25808294391277     -3.59829650699858
  C          -2.31520405437761     -1.99527580846832     -2.55204267467772
  C          -1.89408124089067     -2.13159868263147     -1.41994652163079
  C          -1.50359345356205     -1.75815439850158     -0.17074265557721
  C          -1.25388934649929     -1.06866400104431      0.80114961471776
  C          -1.10365151227052      0.02899981640690      1.66059789404286
  C          -0.61475814184921     -0.07777489164388      2.97279558960523
  O           0.69743196947543     -1.37138814801878      5.92611191370569
  C           0.33640765757406     -0.32584415065483      5.46081717389258
  N           0.32658881169732      0.93916685946031      6.20951652869141
  H           0.89924391657644      0.92848109720754      7.02924327657316
  H           0.32727431032527      1.77860471140124      5.66606125982893
```

```
Structure: C16ONH2_c.xyz
20
Coordinates from ORCA-job 15_3 E -740.392266722921
```

C	-0.72998587423268	0.88505936720659	3.12127328142254
C	-1.25948113473611	1.83894156473905	2.26114408027013
C	-1.74109967345550	2.43729140808384	1.31060711457666
C	-2.26040780584609	2.77334222521381	0.11237648980463
C	-2.67628338733095	2.74407598501431	-1.03353822292276
C	-2.99936883473805	2.20704612667682	-2.23283258539280
C	-3.12272325038304	1.35059601351477	-3.08961754517592
C	-2.99487191908065	0.07304279624767	-3.52775801380531
C	-2.68445070569346	-1.09717202944558	-3.40190852441638
C	-2.17789946275460	-2.10881450170817	-2.65233644217359
C	-1.69725242459898	-2.59760759617857	-1.64648106434781
C	-1.23184704967115	-2.58505303128460	-0.37476339609104
C	-0.92334273748197	-2.15855810629364	0.72450794916395
C	-0.72878016399807	-1.32904742901263	1.77140532640127
C	-0.66187349824042	-0.37164081700798	2.52878695375474
O	-0.38422037014006	2.31580654125264	4.95516636710105
C	-0.29733217192092	1.21430557363861	4.48703596736202
N	0.19280216167391	0.06144687569864	5.25618171301239
H	0.56405851547319	-0.70851727379055	4.73721743282060
H	0.70103979846409	0.32632630346531	6.07565312138846

Structure: C16ONH2_d.xyz
20
Coordinates from ORCA-job 15_4 E -740.347689200731

C	-0.80784608093287	0.93771110435320	2.79164787100627
C	-1.16608268034161	2.02090259858767	2.08274812871533
C	-1.57132452833207	2.77420218359813	1.14966611856924
C	-2.00833338778456	3.30491544626584	0.07033099117065
C	-2.42531645491664	3.14869252671957	-1.15440635062306
C	-2.75115387581285	2.61644923519069	-2.23745534600738
C	-2.85977794652935	1.45696240652607	-2.89076507393799
C	-2.77861135980787	0.24012571272282	-3.02836541329098
C	-2.49321032471436	-1.01079235155289	-2.60889944457478
C	-2.12952169327711	-1.89074862671757	-1.85147778476062
C	-1.65757399442008	-2.56808760012862	-0.74366807063345
C	-1.23608786618738	-3.65015227650632	0.05894227933338
C	-1.24645923500305	-2.30638187015388	0.51352731148123
C	-1.05180238442761	-1.28648146288383	1.42506888804064
C	-0.91576203644651	-0.29901694456601	2.11473313832293
O	-0.20429795638145	2.11432952761091	4.73586199894033
C	-0.32578835651209	1.06048601629886	4.17492176313456
N	-0.06487790784326	-0.22577940016308	4.83699955728730
H	0.46371307591491	-0.14382591169915	5.68191090971907
H	0.11679499977242	-1.02264031747204	4.26079852556841

Structure: C16ONH2_e.xyz
20
Coordinates from ORCA-job 15_5 E -740.328663399206

C	-0.44436374633694	0.39061396390750	3.43952357824847
C	-0.90618090495704	1.15713837743105	2.35250775156931
C	-1.28858055183724	2.13392485917520	1.56248157701914
C	-1.74766778364320	2.27589940454866	0.33981532596371
C	-2.17526145191563	2.24725847247845	-0.82927982466610
C	-2.54863967639537	1.70250696187214	-1.98658667202139
C	-2.81105441350760	0.93103011322596	-2.90447892503441
C	-2.92125015318105	-0.28530883367875	-3.52212184505231
C	-3.15344124636983	-1.35421266443901	-4.43478100013904
C	-2.65703220305030	-1.55909956319736	-3.13739433994673
C	-2.17231488171848	-2.09943817348870	-1.95902772221805
C	-1.75120091275622	-2.18575316516634	-0.82542353641292

```
  C          -1.32620266673111     -1.85624200869678      0.42312597647729
  C          -0.99771251371198     -1.31930420524695      1.45964600672699
  C          -0.77013971101902     -0.29633025611143      2.35334777423510
  O           0.30676564982738     -0.70042608407919      5.38248139290285
  C           0.03582942699635      0.32844595433920      4.82748958798760
  N           0.19660974870156      1.66331641749980      5.42177112027037
  H          -0.30350065959789      2.42250376988457      5.00520883280367
  H           0.32201864663672      1.67434666635588      6.41381493874755


Structure: C21ONH2_a.xyz
25
Coordinates from ORCA-job 20_1 E -930.827090280814
  C           0.15007861985200      0.15950301734722      1.57380831306746
  C           1.64080033023977      1.96160908922502      1.70937156565861
  C           0.59075734795662      1.27344910971728      2.33706893030582
  C          -0.42752661830078      3.09184098735566      3.39013049233404
  C          -0.41565173812518      1.72146734744662      3.17100991468951
  C           1.17206691524698      4.45624156721302      2.05625452505586
  C           0.93846202274614      0.17730792610000      0.33665642425595
  C           1.85927326804838      1.24481164482592      0.50981113774485
  C           1.82328419346621      3.36480812349229      1.42722530787486
  C           2.29024084766968      3.44993439662215      0.06272346504840
  C           0.29411180450390      4.04632175479701      3.10608017791517
  C           1.32665319498386      5.59620202203351      1.25724530342058
  C           0.89221111639576     -0.05792137547582     -1.04302304240541
  C           2.06551685539910      3.11642097829597     -2.56124358806548
  C           2.26099099157849      2.13125381703196     -0.51326927322435
  C           2.16632467238222      4.24932015770925     -2.09748765861693
  C           1.73905018011325      5.67175992211831      0.09854492897782
  C           2.16171013976463      4.62589969743839     -0.73464777978471
  C           1.34073071853740      0.61602641505139     -1.98166487514278
  C           1.96280444554846      1.87872621812738     -1.88115637652480
  O          -1.49763372858471      0.82531191266244      2.95365394400981
  C          -1.21230138165883      0.02248077112825      2.10864060677218
  N          -2.21613477728871     -1.00007251709041      1.78061120035350
  H          -2.02519115239721     -1.45419257732656      0.91037244066623
  H          -3.15705826148398     -0.67651042381286      1.88046391456688


Structure: C21ONH2_b.xyz
25
Coordinates from ORCA-job 20_2 E -930.863749868305
  C          -0.77689752451882      0.38448394124933      1.41658810805244
  C           0.01555674864492      2.60371604132195      0.92721668662141
  C          -0.52525673891056      1.73326411365836      1.86696480281692
  C           0.13621136096885      3.44761883773120      3.43474968891090
  C          -0.43748158897060      2.40918996079862      3.10852014610992
  C           1.94322323447435      5.21926085179603      2.50698626252235
  C          -0.32657622260769      0.04285158753845      0.17799452938704
  C           0.45773328152664      2.16956428995017     -0.34374867077528
  C           0.69410422150529      3.81722545426621      1.24027056531114
  C           1.55950895811295      4.12661661860439      0.15098971198468
  C           0.88303023173947      4.28080149152348      2.55790957279479
  C           2.69750163444321      5.49501580972131      1.57367415527820
  C           0.37650864968199      0.83389371731991     -0.75128405841357
  C           2.39613117310280      2.79118087763214     -1.79359267629878
  C           1.41364495564672      3.10795190046157     -0.83299006609332
  C           3.40692372526655      3.77667791352043     -1.69740335122685
  C           2.70653126479411      4.94169696301823      0.27099061606594
  C           3.53006233121513      4.65667480926006     -0.84504205361964
  C           1.27840379699366      0.68868400385120     -1.84247195237702
```

C	2.10700071068022	1.48689680033742	-2.27570536930376
O	-1.71774725454331	-0.28666848266661	3.46180092667908
C	-1.40847768022168	-0.57122377422745	2.33779887350140
N	-1.68965750044322	-1.89463229580309	1.76297037855728
H	-1.87372639566681	-2.59250902462280	2.45513127678325
H	-1.12268536102895	-2.17623241891532	0.98886188510102

Structure: C21ONH2_c.xyz
25
Coordinates from ORCA-job 20_3 E -930.832125944315

C	-0.35405818040852	0.01988535957967	0.78993454352249
C	0.26516548871662	1.29387526296526	0.76078622310405
C	0.33260412310964	-1.67772298441524	-1.38672517163925
C	0.34038211165729	3.31991689655569	2.11602798676838
C	-0.10974725500852	2.00708223771183	1.84250505351000
C	0.54357758992999	5.30387187750819	3.24827884844354
C	-0.00506858495114	-0.80015955639528	-0.30154856284332
C	1.15636301541155	1.78602038096958	-0.22391978076706
C	1.35560251520848	5.91734222798021	2.46536372745743
C	1.25867516723117	3.87707444682911	1.13159391835124
C	0.01743369975618	4.13331354240946	3.19542245093994
C	1.77541479429021	5.23311473170057	1.33362695504821
C	0.83661204883734	-0.36556970437091	-1.26144805478736
C	2.35168129219819	1.58455369510586	-2.15636124175165
C	1.66264773421875	3.10466357999557	-0.03621957602580
C	2.58372378100212	3.63488286737240	-1.03798508482092
C	2.63944629184369	5.57524919133414	0.27693030212407
C	2.98510566439579	4.93921488619091	-0.70240882462407
C	1.47669033945683	0.92194096514183	-1.31610827609011
C	2.81797393881906	2.72107741074464	-2.04435289393830
O	-0.97667247779633	1.18845247372899	2.57488577056132
C	-1.12409811292009	0.12561241213323	2.03763656247382
N	-2.07108237027468	-0.83299444563670	2.62516843214607
H	-1.89934186941354	-1.76505698153501	2.30616274976944
H	-2.13546072813309	-0.75364078627878	3.61993394202091

Structure: C21ONH2_d.xyz
25
Coordinates from ORCA-job 20_4 E -930.786280773804

C	-0.56411245583685	-0.88291507956808	0.49396099544408
C	0.45043980868853	2.08872506380979	1.22758515334505
C	-0.37090373119802	1.54877226371567	2.25598752208884
C	-0.05720814310792	3.55826531885092	3.47900037582440
C	-0.52348620366907	2.43441727177322	3.32635076905937
C	1.45359701802662	5.48373041455972	2.41170549514665
C	0.12899678458268	-0.42325673283489	-0.39592994061776
C	1.00399640323654	1.73110404350058	-0.10972963234650
C	0.96556912923086	3.42938419313781	1.47684811872083
C	1.76002967807587	3.85673651910697	0.39284830171493
C	0.76272431843448	4.27290193710309	2.60398585221990
C	2.13957967971114	5.82435308160388	1.44971504525266
C	0.90434599201674	0.58248882003425	-0.94096606166360
C	2.50061165414828	2.94365723781586	-1.78919561249700
C	1.78422332373705	2.83279404335523	-0.56722990489465
C	3.12176859048542	4.20675454215749	-1.80776034056975
C	2.42547582826958	5.09946763210950	0.27476782644698
C	3.09779572914258	5.10312634504742	-0.96333228166982
C	1.64034836942952	0.78440572742823	-2.12197091434482
C	2.30653661130903	1.74433262332602	-2.49793413461293
O	-1.05224429324742	0.35790673849631	2.42246804805489

C	-1.19823263560933	-0.65818087717640	1.80097520547642
N	-1.96798907876430	-1.71040592777212	2.48009041367020
H	-2.64241316460097	-1.34212372381324	3.12002819700556
H	-2.34587922177292	-2.37444147256668	1.83491150799091

Structure: C21ONH2_e.xyz
25
Coordinates from ORCA-job 20_5 E -930.757158523465

C	0.13780407913871	0.23530256317503	1.46738859862593
C	1.62144959885981	2.03083045848944	1.66017295042575
C	0.57323055641450	1.32940154435550	2.25243797423075
C	-0.47697720950463	3.11239821818194	3.36056335524545
C	-0.44395498349122	1.75803855484232	3.09595525500146
C	1.18297473908302	4.52269396200124	2.11655609695161
C	0.89583390814974	0.30340267377266	0.20489486300366
C	1.82798718339877	1.41066137792044	0.39662869851419
C	1.89732338466361	3.44231487068084	1.53462710327107
C	2.49315637254990	3.57371738412661	0.24790028581946
C	0.24459290310255	4.06977337874933	3.07241215870228
C	1.32627879742341	5.68428715817519	1.31166050665675
C	0.70710506312979	-0.09687102953240	-1.08898195412079
C	2.18110808496095	2.70342258889061	-1.87145261533310
C	2.40875147782905	2.36563251497270	-0.45768672399483
C	2.16463923886333	4.09902031473140	-1.94092112379811
C	1.77656966325762	5.70757545058542	0.16485157423037
C	2.25529524113641	4.66848981177547	-0.65795405071442
C	1.19612360571858	0.53313326306204	-2.13121206769650
C	1.66455867077221	1.61613302210153	-2.58479751628330
O	-1.51565217027720	0.85121590060552	2.86552087467274
C	-1.22642737715758	0.08035836184107	1.99252166833281
N	-2.26987277784576	-0.84252111101574	1.52303773496509
H	-1.89378481750689	-1.68443729639883	1.13608169258939
H	-3.00454321549168	-0.98197395934767	2.18697466494715

Structure: C26ONH2_a.xyz
30
Coordinates from ORCA-job 25_1 E -1121.291702606328

C	-0.53707065459634	-0.11794175971395	1.84087012013407
C	-0.10090099532213	1.20533472557710	2.04225483386321
C	0.54083773927580	-0.99525689537062	1.63482106802323
C	2.29102982343853	0.69377387099711	1.58046786023816
C	1.70918352030552	-0.57055016283981	1.58498293321537
C	4.40488308983357	1.53985106447830	0.15250382547809
C	-1.13920390234370	2.10447049784029	1.76984550236846
C	-0.93019535036383	3.39537446934595	1.33419870855708
C	1.22222762113288	1.58325598702141	1.99909359180659
C	2.80142597924899	2.88854914046560	1.05299489706212
C	3.31932978756679	1.54282740003092	1.03054247171472
C	4.86381791292717	2.46157392101062	-0.53191874951907
C	-1.72091036218940	4.39041926609904	0.69704656970940
C	0.50408039917586	3.79847708986272	1.28520584568560
C	1.51262389574811	2.89332873310213	1.63889503086442
C	2.23282116670520	4.93743600662311	-0.02738811852775
C	3.18352510778734	3.91627879427305	0.17754692809061
C	4.28858446021824	3.71461754898784	-0.77398069003150
C	-1.24978743174014	5.26620620710998	-0.02688776286987
C	0.87330132187804	6.25606344186786	-1.29794943478347
C	0.91724328677986	4.86944027696884	0.49647420773258
C	2.21903647342221	5.82126268999442	-1.16310874844122
C	4.30821987348041	4.76678516655151	-1.69301929822424

C 3.39303755735524 5.58147897067210 -1.86769353152999
C 0.05418480474246 5.69929463102806 -0.33030754156898
O -2.27924837316946 1.26538811717076 1.52216747464612
C -1.95728156102935 0.10948603597592 1.53726235994763
N -2.99079868813844 -0.91210819533320 1.31570519019271
H -2.61971007529119 -1.80802193882164 1.07150895454050
H -3.78421642316294 -0.59943510027981 0.79347551925353

Structure: C26ONH2_b.xyz
30
Coordinates from ORCA-job 25_2 E -1121.288208208219
C -0.54999165469881 -0.14397424700085 1.86924274334844
C -1.07915744893804 2.09312558415453 1.73784855833809
C 0.56001565323302 -0.99868486187715 1.65224345788350
C 2.34941172630082 0.63161393822612 1.57721911066553
C 1.74346865092175 -0.64432705039681 1.60975288995086
C 4.50446627014586 1.47381985392273 0.18358764583588
C -0.07226135173277 1.18604842584885 2.02686334957844
C -0.83091351929349 3.39053329308391 1.29654919910370
C 1.24692791418673 1.55362980460966 1.96729132716408
C 2.87227427797650 2.80391106841715 1.09450415680731
C 3.38176474376304 1.43304435880116 1.06044342146523
C 4.91376141286612 2.40319967326356 -0.51300602172753
C -1.62000193786911 4.37555025825967 0.64290883982885
C 0.57022983369196 3.78005203208833 1.20506267182280
C 1.54330016728868 2.84912732884613 1.60578709942828
C 2.36893247951499 4.84674935574996 -0.05613720072501
C 3.26825165313192 3.80025528444989 0.28265877330758
C 4.33849585406897 3.69009873700046 -0.71577697423142
C -1.23504850792109 5.31738033504158 -0.03132909209086
C 0.73262081124427 6.42631757279510 -1.37158803023479
C 1.01634190026937 4.86154931515240 0.34869077926853
C 1.91882634424366 6.29998065412390 -1.71733845424675
C 4.12246774196200 4.74748983037794 -1.56434141489772
C 2.90023858433136 5.47310378033250 -1.22233725613476
C 0.08205417963714 5.69990176948176 -0.39348996370232
O -2.23241010058100 1.29713490860833 1.51866702532955
C -1.95779283508807 0.12930632024836 1.54626665738022
N -3.04816088640524 -0.83654034773566 1.34843411220084
H -2.70686194004513 -1.74564885955640 1.10961043377315
H -3.77118002311365 -0.49608811562150 0.74732214138779

Structure: C26ONH2_c.xyz
30
Coordinates from ORCA-job 25_3 E -1121.268172417888
C -0.64690271864547 0.76564143804653 1.87488624717857
C -0.97371542585220 2.13513789592914 1.81391747250436
C 0.80568914372615 0.67826760388680 1.82578842727961
C 2.94737855775346 0.30278624853277 0.96015823536177
C 1.84879870743244 -0.11031710618302 1.41597249624471
C 4.24203708269528 2.43354114849866 -0.11288832816379
C -2.26919813052674 2.28866116193061 1.43455224897627
C -2.63655124682754 3.48274841492691 0.83092995627270
C 0.20222354106729 2.98370834993486 1.75367870773420
C 2.56357965654212 2.41339273977083 1.54603536516106
C 3.49555427783766 1.54571009835101 0.83747930845275
C 4.84256332689509 2.34755239058167 -1.36245720887757
C -1.98071083742113 4.49605379925684 0.62442202092144
C 0.37476398464594 4.26006182052160 1.13448900814376
C 1.26993301731462 2.05816100671728 1.91424923529607

C	3.73009618703279	3.74136904959215	0.12423622696650
C	2.74805159323195	3.71543837124215	1.13464575989906
C	4.77037231532268	3.39750922075909	-2.12862239294903
C	-0.69191431264458	5.02333588090981	0.42399208659175
C	1.04364732428572	5.99063896496314	-0.78802754423091
C	1.72685052348679	4.58693762700982	0.81126091715336
C	2.16281531980855	5.34654685424608	-0.36943037850977
C	4.10046276224097	4.49932781298693	-2.04106748001544
C	3.43243868895461	4.73959351926887	-0.83372725319707
C	-0.15431224691153	5.95047745017198	-0.42756721363914
O	-2.79419042778065	0.98713040119011	1.30157649029548
C	-1.93982118745246	0.17014493690627	1.50794509777557
N	-2.31876790594390	-1.24952848255163	1.46520435005441
H	-3.04909365348902	-1.43679225640364	0.80827469070377
H	-1.52200791839534	-1.84557636558975	1.36570345236836

Structure: C26ONH2_d.xyz
30
Coordinates from ORCA-job 25_4 E -1121.257367424263

C	-0.88466923741146	0.47923931776678	0.94647071680318
C	-1.34876659332850	1.71562314114590	0.44617138795148
C	0.50802845878768	0.34261080690853	0.50785964725803
C	2.85738609961478	0.25010654142574	0.62768309101212
C	1.71784230160741	-0.23461297240611	0.82057480498545
C	4.20982944433381	2.54008374292014	0.10414873545783
C	-2.40291655441225	2.16909102301794	1.16885441607260
C	-2.62581797214250	3.55070956996954	1.21128975329399
C	-0.33960532604287	2.40411691287778	-0.31856350264077
C	2.05044322479522	1.94745907047724	-0.58230980578538
C	3.24366212234142	1.40611270705725	-0.00207737784883
C	5.38518962428817	2.83327260150135	0.74326792249732
C	-2.09449570672374	4.50963901573673	0.66703013520872
C	-0.14167945285743	3.75279587735979	-0.48246142617744
C	0.76850483330874	1.48389035427392	-0.34359921705290
C	3.49764543379706	3.71692629221474	-0.35803891326382
C	2.22484214861152	3.33599324508118	-0.79514047534325
C	5.66826187004867	4.10917188118769	0.88560707370080
C	-0.87675208871445	4.93633362351805	0.07480844083282
C	1.34566448289542	5.56616091273385	-0.30680946259773
C	1.17653725974251	4.20244510641418	-0.74140885329612
C	2.62987065208996	5.93848091010879	-0.04812905724802
C	5.02998286911933	5.17817138203158	0.62804900970347
C	3.75597504056783	5.06705294801876	0.00372134977313
C	-0.00344214446039	5.97234846814891	0.07743063815074
O	-2.65245623438307	1.16517980304706	2.12949288740349
C	-1.84815926831526	0.27927197924435	2.03853298192657
N	-1.99926732116335	-0.87798078194137	2.93230723081574
H	-1.15875771162430	-1.41748465348937	2.98209833580979
H	-2.36281025069469	-0.62454882036353	3.82874951847447

Structure: C26ONH2_e.xyz
30
Coordinates from ORCA-job 25_5 E -1121.230083514493

C	-0.58492805814189	-0.25054910596700	1.82750399529824
C	-1.06722647965923	1.97934370236485	1.58323662683377
C	0.44456578961679	-1.21750065798789	1.75381819335244
C	2.32279332183161	0.30254073260429	1.45192851528402
C	1.63323292653030	-0.92576436195763	1.62959405409431
C	3.87364036835796	2.00979055525479	0.59157089651531
C	-0.03878678491288	1.04915726459698	1.80585734208809

C	-0.82413253223533	3.29286444403791	1.21392344563530
C	1.32503268331024	1.33225521970332	1.67440983333766
C	2.87857933557888	2.99501864682469	0.92634256528396
C	3.54696090866906	0.71817560798952	0.91856615544505
C	4.74398608900009	2.70368797349192	-0.35028225394764
C	-1.42403791951196	4.38958403363871	0.48865953539567
C	0.58710046159769	3.59504953336936	1.22048711345509
C	1.62274865619479	2.67058532228636	1.41084629193754
C	2.05864563606238	5.05437535682759	-0.08253320703974
C	3.09585818849086	4.15422448097427	0.19237319190982
C	4.31549843571687	3.97550686855029	-0.61385890400211
C	-0.48761060114928	5.26982424688185	0.02008213604425
C	0.91138106077099	6.60637960629612	-1.40474846329719
C	0.81112762251452	4.75214124464805	0.48554679646882
C	2.17963375651269	6.03574992756128	-1.17391156787603
C	4.32859395012273	5.06891574753677	-1.51718471491446
C	3.45896060233246	5.91365930542582	-1.74566441225732
C	-0.17397648617613	6.28970030873828	-0.91583128862422
O	-2.24645013722833	1.21045926207016	1.52678224912181
C	-2.01429041767226	0.03887741244799	1.64296245371514
N	-3.14617184773775	-0.89862173208424	1.67194153505632
H	-2.86660007823655	-1.83699041480726	1.46870808103003
H	-3.93405844687404	-0.58078052003796	1.14448379582531

Structure: C31ONH2_a.xyz
35
Coordinates from ORCA-job 30_1 E -1311.960145674239

C	-0.12480509721332	2.08920705183149	2.07945217329586
C	-1.07540622750456	3.86516639497849	0.75813999567389
C	0.54092256176095	3.44009617441001	2.39775889755871
C	1.06451799228758	1.38412231966263	1.41581375454815
C	0.80042708735334	1.16410061050971	0.08055977407791
C	-0.04076755401628	4.44575246666953	1.65827635081033
C	-0.53168900661383	1.73669512997572	-0.26371804551437
C	2.74542459164569	4.29974045589889	1.84731854152479
C	-0.95028204784916	2.47083113005823	0.85656776570292
C	-0.72244609246054	3.72805384662973	-1.64236574171940
C	2.28406543518987	1.99040761473791	1.76946351056677
C	0.13488833453879	5.49469340632823	-0.38312419545857
C	1.74949069706789	1.49387567226905	-0.96154484244500
C	3.28431958751526	2.19782330666764	0.83812787614821
C	0.74759936163208	5.41213822108256	0.92149768833800
C	-0.96384107743637	4.49783771916969	-0.47997905285422
C	2.16690505854649	5.33871952107972	1.01507804484979
C	0.39581923777267	4.34897149576792	-2.34003929011857
C	1.94123670941720	3.32817727718880	2.41266959037287
C	3.01481745316172	2.02351205169935	-0.57741105650687
C	3.73754292735066	3.58956316885031	1.01863582824730
C	2.95513501380316	5.34587027755929	-0.22615424272256
C	2.31868419514957	5.28103389092868	-1.44005279432527
C	0.98371885917822	2.17461890170362	-1.97932671158729
C	1.43728214290097	3.38011568495295	-2.54396966711419
C	3.49291780504868	3.24972524084117	-1.23381942533596
C	3.88162448152095	4.19737798262668	-0.17026085829379
C	2.66273924615078	3.93317185455366	-2.08655813025574
C	-0.41534970336769	2.35412350398128	-1.51036751791030
C	0.91172550472768	5.43514030195856	-1.55495893778091
O	-1.97572972388898	1.69581601339358	3.48124891762240
C	-0.83355246122100	1.45685672212378	3.20137473720537
N	0.00908669782570	0.58994878726692	4.03763419754160
H	-0.50722711334945	0.05661993145256	4.70769417679381

```
  H          0.78070514409790      0.13027584827330      3.59796869016200


Structure: C31ONH2_b.xyz
35
Coordinates from ORCA-job 30_2 E -1311.942633492586
  C         -0.04337624056866      1.88980010327914      2.00008327672543
  C         -0.86781017615099      2.30220780985658      0.84640405100017
  C          0.56109642741348      3.25267858522552      2.36728875184855
  C          1.13553852495804      1.25884952668025      1.22711922277980
  C          0.83147829813793      1.17992902206126     -0.17674494031652
  C         -0.06106117129574      4.29952976672916      1.59900704328101
  C         -0.52037410902548      1.70497159598141     -0.38568675535377
  C          2.76913091444244      4.14199300594847      1.89145363850320
  C         -1.13498265814531      3.96314464702288     -0.63433793199669
  C         -0.36682066586407      4.98274685382679     -1.19465126704439
  C          2.29965740370940      1.83755811002532      1.67514054974444
  C          0.57826889871878      5.66233454992337     -0.36223972248444
  C          1.84764405456090      1.55085275899119     -1.11931772127492
  C          3.33436717986400      2.16512210745862      0.76600454352729
  C          0.76753560999010      5.31933159535941      1.02490142390922
  C         -1.09038190878410      3.69407342212933      0.74668620622759
  C          2.19141550354099      5.23060681001550      1.22063764098861
  C          1.69938697196538      4.75957785323011     -2.17812114888861
  C          1.93191572988428      3.12264439972718      2.40560412021987
  C          3.11420714381522      2.01128281639021     -0.61096846834506
  C          3.71312379819560      3.54321329349677      0.97665745178833
  C          2.85623852998741      5.36938588723622     -0.10713776559067
  C          0.34188660851494      4.46714458550515     -2.34858084488739
  C          0.19478157158274      3.02547110659073     -2.30734975427565
  C          1.49912758655563      2.45016533509843     -2.18934376787802
  C          3.44955609764847      3.29939870563547     -1.28451108522882
  C          3.73651620998840      4.25512625951428     -0.26118353704141
  C          2.47608360514991      3.53772659241366     -2.23644816790049
  C         -0.77103677192266      2.68949554163313     -1.35905772262695
  C          1.87203063797795      5.64087525454290     -1.04029258739601
  O         -1.42519519010085      0.13337863566181      2.72690043067782
  C         -0.67152732258580      1.02379353106046      3.00822952379037
  N         -0.31967830461639      1.34387669330902      4.39914614338964
  H         -0.52518305989828      0.61493830186895      5.05215197252995
  H          0.49694027720361      1.89695493482007      4.56418718811262


Structure: C31ONH2_c.xyz
35
Coordinates from ORCA-job 30_3 E -1311.933444854900
  C          0.39822856186326      2.13765335418777      2.21317396511612
  C         -0.96835082146613      3.67010779339613      0.93236327754145
  C          0.65567115487294      3.60608953264918      2.58164006620087
  C          3.29444870419293      1.59005675538716      0.06859968848885
  C          1.10096528574834      0.99116192894931     -0.41266073188488
  C         -0.16566174572364      4.42650906621687      1.90836705072437
  C         -0.18035691149585      1.69391951736208     -0.27988909606481
  C          2.02491154152460      3.99195963825886      2.24786287104732
  C         -0.50560084058243      2.33329054895873      0.92965266171578
  C         -0.73679242040454      3.78952426141120     -1.47805397896315
  C          1.79530144549853      1.75032362828865      1.77944261641037
  C          0.02924217412882      5.52750648077874     -0.08860112150744
  C          1.76210044108032      1.45820170269822     -1.62314345402276
  C          2.08457531119520      1.03130869880986      0.64566548732421
  C          0.60067154045788      5.44157951287295      1.16734322271737
  C         -0.98318396723610      4.42036054699569     -0.25109432117439
```

C	2.00842185861390	5.12321828057308	1.32793217791652
C	0.31566000969199	4.52046187856088	-2.16082334383237
C	2.73857226589029	2.84744915613737	1.94583467213860
C	3.10745825874901	1.89207712525096	-1.26560590903684
C	3.63939305093347	2.79500502404756	0.83218377911258
C	2.82037971717394	5.02927107439744	0.15668421386439
C	2.21247385046497	5.27457045485684	-1.11478395979358
C	0.90753417212626	2.32387577730037	-2.28624516664301
C	1.32039034341544	3.64994242740865	-2.61911519884163
C	3.43704736705196	3.30640528723313	-1.41915261128001
C	3.66640197265966	3.85693193160816	-0.04275659610233
C	2.56403898932726	4.13376969154557	-2.04531175619618
C	-0.35462903734638	2.43010130000247	-1.50030572254970
C	0.84684610432832	5.52108741523232	-1.24868485344787
O	-0.15055882188108	0.00674189411146	3.04747914446495
C	-0.22003540995498	1.19934555535827	3.16092295123314
N	-1.03196609665549	1.84232092387635	4.20411576195973
H	-1.32563931356465	1.22419303431562	4.93327551788936
H	-0.80745870866364	2.78385876216846	4.45531872303149

Structure: C31ONH2_d.xyz
35
Coordinates from ORCA-job 30_4 E -1311.916231033684

C	-0.08182733533815	2.22777195951133	2.20585706948092
C	-1.24308891945224	2.96844619766209	0.02774049899237
C	0.28402804178116	3.71457465158780	2.31341481356708
C	2.10558557200631	1.44629916549596	1.06922330024965
C	1.33396840212030	1.03885881059217	-0.14056588357966
C	-0.14927834402968	4.69904346291219	1.45454260190006
C	-0.04392473303191	1.24128396192318	-0.17746282376314
C	2.67051735319228	4.18142835888505	1.81364174629994
C	-0.65708682238609	1.98809127325882	0.86013560873952
C	-0.37122441837772	5.03966548843516	-0.86830471923573
C	1.43952916073590	2.04790648278785	2.11495679585164
C	0.63764766903266	5.91608907442107	-0.38990623460667
C	2.09170714042566	1.48798472258906	-1.28781129544574
C	3.28137403305395	2.13686657756406	0.57260431539967
C	0.85768261148741	5.63214873900369	0.98435100709706
C	-0.93166083221677	4.30966750892305	0.24779732288592
C	2.23898945082584	5.36866237603003	1.15139598834135
C	1.58176248830420	4.75320844208956	-2.14893944818302
C	1.67457459387932	3.41496447306301	2.46951791296842
C	3.31717138149872	2.05291704286830	-0.84443460563736
C	3.54361616302329	3.48266771299254	0.92875001265725
C	2.87928657925023	5.41619677709814	-0.19137186931107
C	0.19942226764678	4.33139653707664	-1.96392412913175
C	0.15003639713038	2.88448954329971	-2.04366213626894
C	1.51893254931652	2.42878066256774	-2.19014258195938
C	3.48181849900868	3.37926749940335	-1.40381731737112
C	3.67192552679203	4.27198742059334	-0.32557072108982
C	2.38268920620957	3.59792471313795	-2.28489064146531
C	-0.61658598534208	2.24366838581871	-1.11221378058727
C	1.86614959196235	5.70933282281486	-1.13081720520538
O	-1.91097583430760	2.06915726149062	3.67243626862752
C	-0.87111095873987	1.61384799022691	3.28336749942880
N	-0.32841795382823	0.33496936181195	3.76383993472765
H	-0.75979455092906	-0.00206291517471	4.60069324386210
H	0.65706200885138	0.19267747136290	3.67119946344565

Structure: C31ONH2_e.xyz
35
Coordinates from ORCA-job 30_5 E -1311.909806138167

C	0.12885127969023	2.50915522272300	2.36209983372346
C	-1.10363371623610	3.83740165055975	0.73521057481947
C	0.34629781688180	4.01801240103897	2.48632042613058
C	3.01833833842071	1.95997352541176	0.07460811077503
C	0.74980228081113	0.89910364754936	-0.03928530484232
C	-0.44598583443349	4.75191858240917	1.64948047506132
C	-0.55262591573850	1.61163938644206	0.10668054602484
C	1.71028167866543	4.40572183824370	2.28335584514829
C	-0.80055020329487	2.49747879757276	1.10879135410008
C	-0.42217587760599	3.40385657849099	-1.58876634864047
C	1.54339437032613	2.11456647530793	1.89526883285948
C	0.07791920355396	5.41389933952374	-0.47616615671052
C	0.74406541780608	1.22430920543890	-1.46228650687021
C	1.83580678876842	1.35490690373008	0.74357433878627
C	0.36020249002414	5.67722393635583	0.87195186735829
C	-0.87505542847342	4.29529576843066	-0.57686755008882
C	1.72656845784690	5.52342637509948	1.32456023880929
C	0.79051930488954	3.97594743072649	-2.20496559732795
C	2.42302033057612	3.23981998608245	1.94731361741596
C	3.05157776412192	2.02257243297682	-1.30186931703016
C	3.36646506648216	3.16805876620762	0.85322710992346
C	2.72019346485252	5.52812740958010	0.38093659925456
C	2.40801144732380	5.45853185090896	-1.04183856926531
C	1.83110264521704	1.73477482190726	-2.07885051500450
C	1.82440859016367	3.12374342976914	-2.54633583147269
C	3.16230776022394	3.32589161744141	-1.96544901273883
C	3.60183685441102	4.35277737184788	0.18834579610297
C	3.43590057128858	4.43903237006370	-1.25452268238851
C	-0.45191002462350	2.03511097940657	-1.28222959408490
C	1.09991524621419	5.22656488129885	-1.45962769186158
O	-1.19293329483733	2.31522572960979	4.30193789849111
C	-0.44352240472945	1.80277296478509	3.51722426874123
N	-0.12106105415806	0.36937022597817	3.56492406587953
H	0.74912190649596	0.07859884165578	3.16713817762234
H	-0.39595529491036	-0.07463077807623	4.41774072885572

Structure: C36ONH2_a.xyz
40
Coordinates from ORCA-job 35_1 E -1502.514884877974

C	4.02136561497897	2.92456147896347	1.91206739726190
C	1.31433867786479	1.20603895359724	0.75708970181529
C	2.63636265435944	0.86298805420268	1.01714018481144
C	0.94135806990994	1.54494396228589	-0.54475198256348
C	3.61608441854709	1.28253421393446	0.12489001590793
C	4.39889917552410	2.44729645796831	0.67677553140571
C	0.12526702600112	2.77203983385082	-0.51110459387677
C	3.25819942444654	1.62782315072070	-1.17682155650243
C	1.91143690804678	1.72237242204316	-1.53217224327124
C	4.52496722878640	5.23775748686965	1.14798161162028
C	4.62047237436113	3.37587340285676	-0.43489883604512
C	2.93254724244165	4.98030682526132	2.76385836519698
C	0.81576869708425	2.31815908007616	1.64449821978364
C	0.73029580578981	5.10815133929785	2.16388462014060
C	1.73684279402457	5.83349965049268	-1.60543411219239
C	2.95528457515247	5.16156523294160	-1.90691465775859
C	3.88889228380750	2.91314044437718	-1.52577149780487
C	4.67279322510246	4.78072438636767	-0.18627037031128
C	-0.00127285616981	3.20380299689395	0.80635348024066

```
  C          0.56419952383398      3.66935538530040     -1.53970655375921
  C          1.70429165898475      2.86040459178351      2.53815812475293
  C          2.71555926154360      6.30201429698594      2.12693393588955
  C          1.36370882231748      6.39067484249037      1.77249883535731
  C          3.34004718422872      6.73157538223275     -0.17814370596658
  C          0.58115450039256      5.07258076672720     -1.27001997428218
  C          2.93290506465718      3.75872734343902     -2.17850648408768
  C          1.69288415950289      4.27715505788446      2.77161917173425
  C          1.98685101318104      6.82626602677499     -0.53248220322166
  C          3.94685771191462      5.67358177330914     -1.02318484438979
  C          3.70120047673977      6.47163000728391      1.14834649885917
  C          1.69942223616170      3.06367534725444     -2.13101136524501
  C          0.12775236380832      5.53243903211874     -0.00164958267704
  C         -0.02388778453488      4.60886252816651      1.07110246246478
  C          0.99945175325757      6.64702270277322      0.44669186608583
  C          4.04816032495920      4.35208166758788      2.13490546640646
  O          3.01280101056923      0.97912193559770      2.35113746222409
  C          3.10063744468752      2.11623074054031      2.72432406894517
  N          3.47685871916768      2.22379414765316      4.14128831371716
  H          4.38848222286793      1.83416593944586      4.27217927114567
  H          2.82659899779525      1.70454111473579      4.69584992295743


Structure: C36ONH2_b.xyz
40
Coordinates from ORCA-job 35_2 E -1502.494709637582
  C          4.18397923005745      2.95901043752782      2.15335013034728
  C          1.15477604695527      1.52458669419455      0.74149133004900
  C          2.50942732458666      1.22265879616715      1.09259378731850
  C          0.87099970731093      1.82520974029475     -0.60208334995736
  C          3.52423639853666      1.58510746831697      0.26016059820236
  C          4.52376818404554      2.49015193274103      0.90350428865007
  C         -0.14929788018195      2.88992128591344     -0.64821885074945
  C          3.26092294014758      2.09512612930742     -1.07912981736804
  C          1.90656572569139      2.19192878946751     -1.52362482976444
  C          4.37433259650793      5.30326104621526      1.29329814912816
  C          4.90347126385800      3.41778096334332     -0.11451421657203
  C          2.16067361186132      3.25214841890450      3.16843825145228
  C          0.36849453190588      2.42044577564983      1.56795482951170
  C          0.58577806077877      4.67110396986586      2.18983358892727
  C          1.59463510587600      5.64530772918563     -1.91851083813390
  C          3.71344124875465      4.38304259176745     -1.92103309673217
  C          4.13726842971890      3.13989890348527     -1.35213768809523
  C          4.94992646323475      4.76853381694603      0.08065422626788
  C         -0.38302669118996      3.30230936309189      0.63678214779916
  C          0.22416650283929      3.81913921255865     -1.65573271066775
  C          2.72126580480119      4.54358267407939      2.91880952906962
  C          2.12477746977644      6.35078953301181      1.34517648224169
  C          1.73773205071134      5.44477950200937      2.37459511382043
  C          3.46719942758985      6.40313776107290     -0.57141857734414
  C          0.29967209895532      5.16627485689652     -1.39436095791851
  C          2.36951050027151      4.50265500951986     -2.35893914506420
  C          0.90858082876606      3.25432748101016      2.58463204025979
  C          2.12070166581043      6.51821852417428     -0.99308257797106
  C          4.27330575042664      5.40862545158204     -1.06950241917820
  C          3.48738385745633      6.33941012059384      0.90919504014214
  C          1.51092137377461      3.36705217443816     -2.21460749886529
  C          0.17529782788445      5.63102795184881     -0.01990202841932
  C         -0.16461925984196      4.68781915300490      0.99296782080682
  C          1.27459852921841      6.49958215648882      0.21579433643077
  C          4.00298848320067      4.38715469883468      2.32076779623552
  O          2.83888947624941      1.17449435541871      2.42862524447777
```

```
  C          3.24793227612677      2.16726518719707      2.96443310481677
  N          3.81079675094121      1.78101546115740      4.26631433479709
  H          3.99922685676875      2.58582496360019      4.82914268860350
  H          4.66513946237163      1.27976994866237      4.12901370292265


Structure: C36ONH2_c.xyz
40
Coordinates from ORCA-job 35_3 E -1502.484754487877
  C          4.08106847251927      2.87090860687262      1.98781238904548
  C          1.04855732283330      1.33546097407613      1.05080772566844
  C          2.36802621996624      0.98410287940358      1.36432986660841
  C          1.02786214142612      1.65433668023356     -0.40014828364418
  C          3.19403465149088      1.40433096166980      0.30458432116151
  C          4.18682018535756      2.40729541679486      0.66209733998148
  C          0.18295142236493      2.77700530788809     -0.63854310597924
  C          2.34863018715763      1.66503722066208     -0.85725703900716
  C          2.73575118115316      2.61177402608629     -1.83771353174207
  C          4.33525284883780      5.21524036911722      1.18620936442130
  C          4.57002904980477      3.34671609425849     -0.37315540029226
  C          2.18219954527728      3.20676015724295      3.19542090134852
  C          0.33948281689986      2.35576099584988      1.69652125936348
  C          0.63284633219764      4.63258395153102      2.23938901693424
  C          1.61780036619613      5.81835025648441     -1.93551893385214
  C          3.76725168737579      4.71386535813741     -2.12723089542085
  C          3.95884418148392      3.32272923696519     -1.66102931925549
  C          4.79984015336398      4.70852733895049     -0.05141128797741
  C         -0.22426493269223      3.23131847484099      0.64723637301106
  C          0.55843199697201      3.72474388427793     -1.63453868423920
  C          2.77226887554333      4.50568444680474      2.93136681349253
  C          2.15862699888096      6.34153785238358      1.39212757096766
  C          1.79173649830025      5.41682805065169      2.41087150335000
  C          3.48039049821508      6.54851485950273     -0.56186454615390
  C          0.45142642461629      5.12579465663994     -1.35682310316438
  C          2.43621173801455      4.84720236376470     -2.53306484822398
  C          0.91032202671640      3.23573135100352      2.69853922977455
  C          2.14096174609720      6.67138381366597     -0.95432985845596
  C          4.29163507384131      5.56677452505564     -1.14507621263681
  C          3.50020900642847      6.33379862591588      0.90335153046890
  C          1.77809041660385      3.53087531749956     -2.33742246986295
  C          0.28554526763088      5.56199864398244     -0.00371743776235
  C         -0.05184054591823      4.60278415819245      1.00305888439912
  C          1.30829099780806      6.51025395141405      0.26487084632751
  C          3.96857292226170      4.28481002196741      2.20230453056420
  O          2.87505296172279      1.05388996898159      2.63089309624913
  C          3.29733493189065      2.12142326206752      2.98025050212718
  N          4.12926323761075      1.90919499074213      4.17346232735776
  H          4.47328972663980      2.77642800703630      4.53339659459083
  H          3.57703539437227      1.46772296034730      4.88067294051773


Structure: C36ONH2_d.xyz
40
Coordinates from ORCA-job 35_4 E -1502.470300719534
  C          4.50410693664141      2.79589184466249      2.05572129646919
  C          1.44411803150216      1.62460026554944      1.00384817666830
  C          2.75870459311030      1.09889339578115      1.06695061407786
  C          0.96310175091064      2.06050637162773     -0.24732919750472
  C          3.69380136974945      1.53451620692688      0.14343827168649
  C          4.73144078998103      2.33941365476753      0.74630645173509
  C         -0.03863620371317      3.12286183591573     -0.36797201627694
  C          3.27963203221881      2.14829233443209     -1.08519168613671
```

C	1.90382614898137	2.21355042196940	-1.35440913712580
C	3.39326777220774	4.99040996209255	2.36627116882093
C	4.94905677894907	3.46852472492794	-0.16288746628785
C	2.28850232543970	2.90202220948802	2.91734396629841
C	0.26703642335118	3.52864074572345	2.01716698060259
C	0.73080580561313	5.75741452662109	1.72932468464166
C	1.49318723861696	5.52296706146371	-2.25501501383132
C	3.55362703403120	4.46743578570559	-1.88222804860496
C	4.11931625727913	3.32758121122666	-1.32272666483483
C	4.84901010085944	4.61003227747602	0.59729933034339
C	-0.35374150492421	3.88955946383789	0.78637459834384
C	0.16618478544670	3.79670606928252	-1.60537824499275
C	2.27937759308583	4.31322535964542	2.96367297710744
C	1.82916937498281	6.44392877411619	1.07373684762858
C	0.95983589949520	4.71695391312026	2.56452593423500
C	4.02696104000441	5.70110471781932	0.19677375042500
C	0.19536627667820	5.19657538175672	-1.69879791396808
C	2.24848096455812	4.33952485653516	-2.50996868218660
C	1.13117434245299	2.44990048357052	2.16936752102070
C	2.23751646851303	6.32853042513331	-1.34384124358432
C	3.53364627265387	5.71172932647082	-1.10854462893149
C	3.13790992511287	5.99933915268808	1.33214278354640
C	1.42885227601100	3.26483427834065	-2.17946677393545
C	0.09504300163512	5.94209989595431	-0.49319252838970
C	-0.23768460231606	5.33119300179727	0.68293888688841
C	1.41276044387004	6.65443363333401	-0.27490635320396
C	4.51831916814688	4.20328612132401	1.97591860522716
O	3.28545416621897	0.96956896697247	2.34495284646344
C	3.55870535458425	2.01513594920075	2.86660727057040
N	3.97455798397422	1.75971170197089	4.25323088548192
H	4.82594357492935	1.23529078456809	4.24203835418174
H	4.14810205229454	2.62329293574951	4.72663336210047

Structure: C36ONH2_e.xyz
40
Coordinates from ORCA-job 35_5 E -1502.463709193911

C	3.80552269763772	2.13941792433904	1.55144341662878
C	1.93648257380949	1.28130335360362	0.24012137511386
C	1.27444091552124	1.45058624838874	1.40456527528496
C	0.38581977216489	2.80670254586282	-0.87835521486892
C	3.42687925155974	1.47345005738267	0.35902147736414
C	4.62534726503983	3.29526240681097	1.43855937165673
C	1.48792332601622	1.93974184609521	-0.95683492457194
C	3.83306177261550	1.98272975879638	-0.88562325544768
C	2.63597179989845	2.33753560105897	-1.67340433751926
C	4.35021026784946	5.63782164148183	1.07873617751076
C	5.04525502614733	5.09229001014185	0.00913753900612
C	2.24293075607956	3.33436860442729	2.91640406310205
C	0.37659354944626	2.55777575757395	1.56047778715702
C	0.70447921812188	4.92596477478794	2.39751554427545
C	1.70683323153523	4.56023756164643	-2.05442899555264
C	2.79477804792290	3.65651726733616	-2.17305920104609
C	4.74045076954861	3.02778794870735	-0.97766880915287
C	5.18577158985239	3.64144814649759	0.20281717134187
C	-0.21097240457133	3.10523749352933	0.42034365179803
C	-0.56293563940263	4.54008427174753	0.38581202408601
C	2.94265140627247	4.61193751957356	2.74996199080471
C	2.07341571346804	6.49833709028758	1.34056366850514
C	1.97386621055706	5.61437978307813	2.48493769635328
C	3.14967896879888	6.17873547188273	-0.85250733484232
C	-0.20685405231930	5.05805436232706	-0.84511805468184

```
  C          0.46566821633996      4.05290875917102     -1.60048133639411
  C          0.92088592781753      3.49211401705350      2.58113475030497
  C          1.91394463014599      5.89716222002538     -1.43249824799604
  C          4.28762071493713      5.32011274479358     -1.17916547332475
  C          3.23840718744853      6.45706368793037      0.57941203459445
  C          4.10663536029036      4.09625984839536     -1.80082256546003
  C          0.79805493931353      6.46742578880592      0.68079983038602
  C         -0.04395659894163      5.43478560352500      1.37258015841318
  C          0.73245869341147      6.17357769439269     -0.66111838088313
  C          4.13423291497007      4.59743004339261      2.04663671206431
  O          2.05393946264488      1.14755314130749      2.51295538197545
  C          2.91533613851526      1.96006534406949      2.70742822883896
  N          3.63207709436608      1.59566247909126      3.93803497312234
  H          4.05864595510075      0.70043544950564      3.80916461815223
  H          4.35428737849966      2.25921574484631      4.13325117337797
```

```
Structure: C6ONH2_a.xyz
10
Coordinates from ORCA-job 5_1 E -359.560238164954
  C         -3.72509754167157     -2.75261059469405     -0.72164537352884
  C         -2.69465205646391      0.49445816100720      1.15465197075580
  C         -3.38019079975171     -1.66092154570531     -0.09881883839879
  C         -3.04973817278166     -0.60642644587029      0.50645917689804
  C         -2.37843750564743      1.50642836824397      1.75718366549930
  C         -1.94376713210559      2.72683402849749      2.45185747072354
  O         -1.29941611936472      3.58331596177463      1.91218120716022
  N         -2.43658810666653      2.80112817686973      3.83479198523571
  H         -1.95733401855230      3.48290722837541      4.38750605218509
  H         -2.64356854291511      1.93074666402053      4.28155267839323
```

```
Structure: C6ONH2_b.xyz
10
Coordinates from ORCA-job 5_2 E -359.554518083490
  C         -3.89296275317833     -1.54873205144678     -0.30355445918243
  C         -3.04931041913466     -0.78872579784731      0.45749219763474
  C         -2.70735458034419      0.38663669500273      1.10530786486864
  C         -2.74153586498467     -2.10582521921982      0.12900401122389
  C         -2.41710211678707      1.40098609742836      1.68384602808201
  C         -2.03531925362402      2.65221577186889      2.35434297219824
  O         -1.45789780462545      3.54317479762354      1.79507873789979
  N         -2.49431714036555      2.70723090165114      3.74976200615836
  H         -2.05735470870291      3.42710510876654      4.28906243736001
  H         -2.65563535417368      1.83179368810847      4.20537819868002
```

```
Structure: C6ONH2_c.xyz
10
Coordinates from ORCA-job 5_3 E -359.524473797421
  C         -2.93058496634496     -0.16317494230743      1.02248378465918
  C         -2.32217572641250     -0.84720141860978      0.07552095820009
  C         -4.04096223228858      0.14613274368274      1.91457219253053
  C         -1.72439210716009     -1.50230877843754     -0.84545846983047
  C         -2.91577289350973      0.87616113062660      1.93821960320848
  C         -2.05951898938293      1.97855819691289      2.39914255207254
  O         -0.99421495517251      2.24208719450573      1.91367394492429
  N         -2.65259711099094      2.67117662377852      3.55215515448183
  H         -3.63219318755654      2.55314989716429      3.71482418789614
  H         -2.23637783768523      3.55127934991151      3.78058609207244
```

```
Structure: C6ONH2_d.xyz
10
Coordinates from ORCA-job 5_4 E -359.471013576125
  C          -3.38720266008625     -1.43647826052488      2.23619822754352
  C          -2.48014951167687      1.34167172940022     -1.29469243596077
  C          -3.02526327814294     -0.31901666686289      1.74176788803410
  C          -2.54649182926818      1.12716157465150     -0.11001936479540
  C          -2.63260895778838      0.87416464112060      1.27825220529294
  C          -2.23704219155685      1.97355259356161      2.17031331401017
  O          -1.71278631552658      2.97177933194533      1.75958336522841
  N          -2.58463108625059      1.76095565261055      3.58271711811946
  H          -2.13249362112417      2.38667801212671      4.21836107292476
  H          -2.77012054979147      0.82539138919879      3.88323860452608


Structure: C6ONH2_e.xyz
10
Coordinates from ORCA-job 5_5 E -359.448338389773
  C          -2.83103836505252     -0.60116777504274     -0.70942059825930
  C          -3.16516519383142     -0.29202038608663      1.83399110255014
  C          -2.70147201367230      0.07650548653104      0.40171757124866
  C          -3.78486833920885     -1.31404455584389      2.35306487005188
  C          -2.50235424966292      0.88316118732863      1.49714766975483
  C          -1.98856148453006      2.18312883670053      1.95212141620860
  O          -1.43845042704150      2.95477309644594      1.21595366363126
  N          -2.16211812644267      2.38767092140722      3.39743820934096
  H          -2.02600527171503      3.33628968483605      3.68308786941176
  H          -2.90875653005502      1.89156350624314      3.84061822098448


Structure: C8ONH2_a.xyz
12
Coordinates from ORCA-job 5_1 E -435.722881954123
  C           0.00330092980509      0.03983927425311      1.95865701490039
  C           1.14698730511537      0.03876399638456      1.56185708839111
  C           2.41856696651329      0.04109426865578      1.11974489746296
  C           3.58120811471567      0.04975654437445      0.71411930116917
  C           4.81934390849384      0.06194697444937      0.28306689242552
  C           6.01621562840576      0.07585169427780     -0.13419052078641
  C           7.24022981703530      0.09599189991985     -0.56031583943773
  O          -1.92178828864870     -1.13144287669947      2.74609678179092
  C          -1.42272767276302     -0.04709448560802      2.46332690137585
  N          -2.04817494841604      1.16426376146849      2.56138499444215
  H          -3.00757870169184      1.18179655552634      2.88384073762321
  H          -1.58377305856474      2.02277239299774      2.30085175064282


Structure: C8ONH2_b.xyz
12
Coordinates from ORCA-job 5_2 E -435.705368948905
  C           1.16772837678128      0.02362740739361      1.56033654918944
  C           1.75033742715185      1.25233156042882      1.21018932902712
  C           2.03986642514766     -1.03659804805141      1.37822331340448
  C           2.94367961659241      1.37243686764398      0.77647601631305
  C           3.22516305108391     -1.09248340979559      0.96834265725965
  C           4.27691640943924     -0.41778802936805      0.51879631738718
  C           4.12616279966819      0.95970780708320      0.40972136722209
  O          -0.68647966630724     -1.25183966066888      2.36876646826925
  C          -0.24576312876393     -0.14412872619700      2.07966932880220
  N          -0.94324778494334      1.02657221503337      2.18532397468764
  H          -0.51676561442721      1.90612042288533      1.92217273438297
```

```
  H          -1.89578790891575      0.99558160351281      2.52042194700695


Structure: C8ONH2_c.xyz
12
Coordinates from ORCA-job 5_3 E -435.670346936093
  C           1.40874294005162      0.73138217991238      1.43286340144449
  C           3.46622518471449      1.05106287297297      0.70855317185341
  C           2.52107564550202     -1.58015225694758      1.15490350387131
  C           1.56786609274023     -0.70719633821212      1.44425383405357
  C           3.56576509235279     -0.86610594102879      0.76267285500120
  C           4.38561664585637      0.08808934395978      0.43574189019655
  C           2.39566834746660      1.64287684504732      1.04975346367842
  O           0.13439148969093     -0.82845387137667      1.94282205185930
  C           0.01677393042155      0.39249725137688      1.92620516098047
  N          -1.11786352899306      1.05863760409899      2.29639883080063
  H          -1.15047317847454      2.06588296056498      2.20082639204535
  H          -1.95197865353017      0.54501935424025      2.54344544716728


Structure: C8ONH2_d.xyz
12
Coordinates from ORCA-job 5_4 E -435.620907856210
  C           1.13851097317817      1.37907890556810      1.33058542458816
  C           3.50953424985309      0.82796324076457      0.62493383867047
  C           2.54755903021826     -1.26403732349999      1.27600355706890
  C           1.40461686127633     -1.22491263513049      1.66076159819043
  C           3.63632021508031     -0.55181370206758      0.80024603769434
  C           4.73638382518956      0.14079750843606      0.32212461678136
  C           2.31554658792822      1.36491149505899      0.93868109363863
  O           0.22012337529075     -0.74079834871194      1.98357427709340
  C           0.04724432062566      0.46656722515106      1.85237666803711
  N          -1.20811338382635      0.92103841450371      2.14515896495423
  H          -1.31159458032857      1.92473984495744      2.22649627598817
  H          -1.79432146668659      0.35000537428670      2.73749765024682


Structure: C8ONH2_e.xyz
12
Coordinates from ORCA-job 5_5 E -435.622594458248
  C           0.34432669541047     -0.82104935068141      1.51426601344175
  C           1.24114531104215     -1.56891997196939      1.07187307490553
  C           2.38457849201768     -2.11353441232167      0.60375663478211
  C           3.14371036677395     -1.07794707910274      0.62334021684819
  C           3.39854022786499      0.19817506805570      0.84675764163341
  C           3.78502856476395      1.60446987172969      1.06062291735334
  C           2.53966390745962      1.16270178187992      1.32926493986108
  O           1.29888171682819      1.42637574434923      1.75699938486389
  C           0.35124344033594      0.65420475222813      1.85978624580583
  N          -0.78434328645848      1.22881448124930      2.35823875438804
  H          -0.83582969824098      2.23097600361480      2.49151336149122
  H          -1.62513574058216      0.66927311557685      2.38202082286940


Structure: C9ONH2_a.xyz
13
Coordinates from ORCA-job 8_2 E -473.758668689315
  C          12.80749200345967     -0.71811057938069      8.20335989794196
  C          10.55761727771021      1.38289348002199      7.07796982527224
  C          11.68142784343047      1.58291468238714      7.75900766688149
  C          10.15012219135077      0.88494357486441      5.93862356362547
  C          11.60348900996183     -1.13156735874020      5.93739444956689
```

C	10.51991944231853	-0.36890137134467	5.88945195436299
C	12.30069310669319	-1.16281723342826	6.99927224637061
C	12.33701767230832	0.57479443473161	8.18016406369517
O	13.25505368764892	-2.76506222264712	9.27109624096948
C	13.12128542088209	-1.57552722510386	9.35543211429723
N	13.33785365334786	-0.83953311991605	10.60935032642033
H	12.87179864140241	0.04045752570875	10.70105193656593
H	13.30496005621694	-1.43282458969192	11.41366571170703

Structure: C9ONH2_b.xyz
13
Coordinates from ORCA-job 8_3 E -473.687843796442

C	12.04989712467540	0.11277231170332	8.90441772518933
C	10.24394589014399	1.89372370872335	6.00670735259533
C	10.84523559266094	1.40530321260967	7.13471731397247
C	10.84254588328236	0.57893641530158	5.93401297686718
C	12.04748618332707	-1.45385217628519	6.62979978029415
C	11.39973164961098	-0.59139054814866	5.93024246483621
C	12.68434283843506	-2.23534878962772	7.41776208988397
C	11.43394621929405	0.95733327230520	8.26360327377074
O	13.03332070930508	-2.02166238200676	8.72110662945586
C	12.79086933848298	-1.05843182479881	9.39445958311286
N	13.20992757833623	-1.12720106797620	10.80178363523541
H	13.96654949695040	-1.75999816102102	10.96637739926251
H	13.30093150751837	-0.22852396802589	11.23084977320065

Structure: C9ONH2_c.xyz
13
Coordinates from ORCA-job 8_4 E -473.747022856072

C	12.90361448942720	-2.15858074651148	8.00324315355918
C	10.39523031022862	1.45081243699157	5.99403550317702
C	11.42303194548768	1.12349979465205	8.37531324830146
C	10.86006747876751	1.40914693983355	7.21543015078425
C	11.60421804100114	-0.85958212140193	6.19341638769473
C	10.99094626056304	0.19082473703642	5.92820007726684
C	12.19467292415405	-1.13286946393601	7.44391162814865
C	12.03422422453507	-0.06268763342734	8.45137993184121
O	13.87842549068703	-2.46384044824496	10.27535304024004
C	13.28351042122774	-1.89920491065175	9.39941767223829
N	12.72714743210813	-0.56609680291872	9.67178322139829
H	13.46143971262521	0.05678243750016	9.94166522242926
H	12.09220127062709	-0.61654422146040	10.44269075530584

Structure: C9ONH2_d.xyz
13
Coordinates from ORCA-job 8_5 E -473.740711069129

C	12.23624138731853	-0.67423666837049	8.15263304470726
C	10.99260956477191	1.27022504641427	7.15481013391841
C	11.23648331196025	1.90607164204003	8.44631472397737
C	10.93875457241802	0.42680664346284	6.16215060021078
C	11.83430628460565	-2.13737237927166	5.82036783295799
C	11.28413547515042	-0.77832017235984	5.82641944130335
C	11.98672303016913	-1.34136785093026	6.93103418545439
C	11.70976752541957	0.63096212638975	8.19813256599559
O	13.39090742055966	-2.44054118191320	9.18954530554517
C	12.96827292429963	-1.31917920744612	9.25221430820456
N	13.19896625340159	-0.44596055356282	10.41202884499001
H	13.50024039183649	-0.94291768586409	11.22582703742787
H	12.57132185423691	0.31749024416449	10.56436197827581

S5 C_nCOOH family: Energies (Hartrees) and xyz coordinates (Angstroms)

```
C6O2H_a -379,43519
C6O2H_b -379,43510
C6O2H_c -379,43524
C7O2H_a -417,52343
C7O2H_b -417,51667
C8O2H_a -455,59194
C8O2H_b -455,58607
C8O2H_c -455,54676
C8O2H_d -455,54525
C9O2H_a -493,59956
C9O2H_b -493,58366
C9O2H_c -493,57046
C9O2H_d -493,57947
C9O2H_e -493,56912
C10O2H_a -531,74713
C10O2H_b -531,74149
C10O2H_c -531,69247
C11O2H_a -569,85054
C11O2H_b -569,82617
C11O2H_c -569,77359
C11O2H_d -569,80585
C11O2H_e -569,80092
C16O2H_a -760,26921
C16O2H_b -760,26921
C16O2H_c -760,22490
C16O2H_d -760,20233
C16O2H_e -760,20010
C21O2H_a -950,69399
C21O2H_b -950,74095
C21O2H_c -950,70814
C21O2H_d -950,66207
C21O2H_e -950,63864
C26O2H_a -1141,21153
C26O2H_b -1141,19445
C26O2H_c -1141,20316
C26O2H_d -1141,18296
C26O2H_e -1141,16402
C31O2H_a -1331,76518
C31O2H_b -1331,74614
C31O2H_c -1331,73863
C31O2H_d -1331,73092
C31O2H_e -1331,71666
C36O2H_a -1522,38959
C36O2H_b -1522,37237
C36O2H_c -1522,36321
C36O2H_d -1522,34479
C36O2H_e -1522,33878

C -37,84496
O -75,07371
H -0,50211

Structure: C10O2H_a.xyz
13
Coordinates from ORCA-job 9_1 E -531.747132443580
  C           0.46029125667692     -0.61299541561068      0.80170532196469
  C           1.64857462263720     -0.47171620954069      0.65026429198015
  C           2.97951996830306     -0.29455938307072      0.47524591347004
  C           4.17892977140087     -0.13725593512390      0.31921175893168
```

C	5.48905831041751	0.03639657264345	0.14802558370848
C	6.70639024305809	0.19447290002463	-0.01056300087718
C	7.98820389397941	0.36364661119846	-0.18065752508688
C	9.23721166680719	0.52852683577557	-0.34629822086860
C	10.50853739493860	0.69646516398683	-0.51538524675835
O	-1.44748507285442	-1.91844088977503	1.32550271702396
C	-0.97395598958099	-0.86368367591655	1.00416578137584
O	-1.71149051044183	0.30279802781784	0.76887177569165
H	-2.62999555763100	0.06806538892473	0.92003085740655

Structure: C10O2H_b.xyz
13
Coordinates from ORCA-job 9_2 E -531.741485499283

C	0.37346970433835	-0.77756933778996	0.85492852665530
C	1.55511772633808	-0.59056009721120	0.69340133642257
C	2.88161609840532	-0.38472482599334	0.51029698676150
C	4.07526379508043	-0.19719127306767	0.34651027458350
C	5.37981346376295	0.01155054971590	0.16622948654996
C	6.58969986232079	0.20671755936600	-0.00302115507420
C	7.86529675277090	0.41480896266960	-0.18235110345639
C	9.10631602246060	0.61882081488027	-0.35833448680493
C	10.37077626859765	0.82789089897163	-0.53876702201618
O	-1.50677684191607	-2.10341297067293	1.38226867771373
C	-1.05883506997955	-1.03827964410861	1.05847993176947
O	-1.87405775876194	0.07862303566539	0.83952699129028
H	-1.32391003099874	0.82504632420063	0.59095156356738

Structure: C10O2H_c.xyz
13
Coordinates from ORCA-job 9_5 E -531.692468980929

C	1.15400908087106	-0.67126259770989	0.76853149529102
C	2.22622013648610	-0.36899989645336	1.21748138255439
C	3.46982796054011	-0.01576193515346	1.73803453062757
C	4.28682518432868	0.36334127014765	2.79187879514794
C	4.77602559286540	0.18466641116675	1.45366625386584
C	5.77566823081919	0.20010547271960	0.54762260234042
C	6.68580279446719	0.21234943036291	-0.29013952979493
C	7.65344643688363	0.22605537552474	-1.17622591978128
C	8.59105656088761	0.24015400054724	-2.04752321710028
O	-0.46489428167455	-2.21702120231048	0.02202642257127
C	-0.14771617899169	-1.07655738655407	0.21885206936145
O	-1.00147877776556	-0.00383185195230	-0.06460755046086
H	-0.57100273671482	0.81848290099859	0.18052267333946

Structure: C11O2H_a.xyz
14
Coordinates from ORCA-job 10_1 E -569.850544798566

C	1.05692426489131	-1.79797974791185	1.04186669018187
C	1.21035597266589	-0.43127451591448	0.92025354624017
C	0.84183401031241	0.68720911671993	0.52787969837220
C	0.04644277307086	1.54502365528594	-0.07776499196536
C	-1.03401604920514	1.60842792193548	-0.72979789085201
C	-2.04788535390722	0.97273135783777	-1.23478806776240
C	-2.36320177511773	-0.28585122192436	-1.22732569268727
C	-2.05808250758824	-1.45835293053356	-0.86400994253089
C	-1.13639700646305	-2.13356354886749	-0.21107521984239
C	-0.08571843877160	-2.27659671430109	0.43638664554694
O	3.03492943456163	-2.23305679465353	2.28869382499355
C	2.03549362873324	-2.63028998193028	1.75640267904229

```
  O          1.65812341810693     -3.97842556536905      1.74534933034213
  H          2.34445762871069     -4.44476103037338      2.22812939092117


Structure: C11O2H_b.xyz
14
Coordinates from ORCA-job 10_2 E -569.826172710955
  C          1.16800675030744     -1.69089862974851      1.09496696588477
  C          1.38243431873163     -0.32192790090488      1.00832419713785
  C          1.01719283472888      0.78846063093973      0.61768834605299
  C          0.02421268629030      1.35772438010817     -0.06352703203532
  C         -1.04613723523973      1.27929723477751     -0.68823314195728
  C         -1.98112247866161      0.42755119260911     -1.11254028526433
  C         -2.93932355336329     -0.41551085092621     -1.55218039162695
  C         -1.96376289638218     -1.07548213815580     -0.86628654507311
  C         -1.04590270780697     -1.77443615070855     -0.20911052217673
  C         -0.00726914303815     -1.96912927138009      0.43909617191214
  O          3.08070112361819     -2.36623737112720      2.33707105044095
  C          2.05899129591751     -2.64394286116716      1.77228021492262
  O          1.56158349497698     -3.94928557003077      1.67918934256393
  H          2.19365551207953     -4.50294270209683      2.14346161910744


Structure: C11O2H_c.xyz
14
Coordinates from ORCA-job 10_3 E -569.773589096875
  C          1.14844780819070     -1.54083810557928      1.05803464402930
  C          1.25989316963624     -0.18165888544993      0.91343724512093
  C          0.94692500768170      0.94686174807210      0.54753430886519
  C          0.02312169803427      1.65984502147287     -0.10701073922814
  C         -1.02591880215455      1.80233293484381     -0.75839263894920
  C         -2.28066322978841     -0.35964862236646     -1.16845133608011
  C         -1.85804355072499      0.81693306002999     -1.09345187877679
  C         -1.74824860864061     -1.46707288042856     -0.67782682770447
  C         -1.04720109627471     -2.35759479338413     -0.12527324455074
  C          0.08957155076589     -2.39979717420182      0.55801885308761
  O          0.81307409140944     -3.56953029785716      1.17291169630229
  C          1.66349048920658     -2.82016237353060      1.56691642511884
  O          2.78689360881585     -3.21715377717880      2.30201047357138
  H          2.73191786070935     -4.16927584637819      2.41174303024999


Structure: C11O2H_d.xyz
14
Coordinates from ORCA-job 10_4 E -569.805845012777
  C          0.99296332500719     -1.58925389402270      0.97473600746033
  C          1.12256963641154     -0.22377069870647      0.83983066025210
  C          1.54218080355704      1.05712331162760      0.89121974740761
  C          0.37670567367236      0.93929544537362      0.21403516960924
  C         -0.75622565090762      1.02273830785444     -0.47476019765495
  C         -1.78330432757606      0.55930613679523     -1.01522381669496
  C         -2.38545108133461     -0.59873644839874     -1.19435102209998
  C         -2.22497300238516     -1.80895020404801     -0.91017097605783
  C         -1.18493109007527     -2.29927736432424     -0.21336942057108
  C         -0.12611683517068     -2.17155917652405      0.39825927812806
  O          3.00861715434264     -1.79655221535523      2.19815503905044
  C          2.04189246056753     -2.31195601673009      1.70845892197603
  O          1.84806877702773     -3.69461128366996      1.81188423909865
  H          1.03126415902190     -3.94055589709935      1.37149637056887
```

```
Structure: C11O2H_e.xyz
14
Coordinates from ORCA-job 10_5 E -569.800916045574
  C           0.47206553796986     -1.92150759125489      0.71383153485007
  C           0.24392244500826     -0.56451276608063      0.36659318031076
  C           0.57920269751702      1.83177182178782      0.19254485848274
  C          -0.57464689958477      1.73047285933287     -0.47732259924362
  C           0.85400816432843      0.65953245233543      0.53848540544288
  C          -1.43180898586158      0.88630297702397     -0.85516840531269
  C          -1.01171724406895     -0.38914329097290     -0.40680594402668
  C          -1.53942962134516     -1.68152471367083     -0.51894548721337
  C          -1.71444077329795     -2.99183295930187     -0.41833297519827
  C          -0.56361893130718     -2.65835892601353      0.21340984489418
  O           2.51281601748476     -1.66057085503344      1.89039161421345
  C           1.64024268163308     -2.37588976881846      1.48181526033778
  O           1.62229727690507     -3.76053191716222      1.68783488852540
  H           2.41436762619409     -3.96096732998280      2.19186882440990


Structure: C16O2H_a.xyz
19
Coordinates from ORCA-job 15_1 E -760.269214414326
  C          -0.69187528077911     -0.51356158365927      2.74767264782140
  C          -0.97831737338825      0.73438753819541      2.21140574338685
  C          -1.24239427522635      1.66904298952450      1.46923865465884
  C          -1.52836232102263      2.45157662946879      0.40908978119592
  C          -1.75916790680449      2.84497504967773     -0.72177985069308
  C          -1.94480243087096      2.78932650480419     -2.06107022540752
  C          -2.01790687800620      2.26782949756288     -3.15890062639659
  C          -1.96234297760550      1.20412816167116     -3.99891357083886
  C          -1.79768387025627      0.01893011394218     -4.22198751269289
  C          -1.52682792497968     -1.24893292292364     -3.82257995168049
  C          -1.26509971923480     -2.08876458850510     -2.98092787436972
  C          -1.00404850501193     -2.56725125075272     -1.74197334359938
  C          -0.83129258076108     -2.55732683352399     -0.53535106277048
  C          -0.71041768563200     -2.15769980374523      0.74754142897863
  C          -0.66560974915500     -1.51792327408163      1.78707163520490
  O          -0.18922531548176     -1.86342600124679      4.63692863193662
  C          -0.43556714678850     -0.78611191437453      4.16926417233039
  O          -0.51460235441700      0.38576137816992      4.93114764497930
  H          -0.33564570986276      0.12075030111273      5.83632367750860


Structure: C16O2H_b.xyz
19
Coordinates from ORCA-job 15_2 E -760.269210143600
  C          -0.71508354899079     -0.24282885421771      2.78867598768623
  C          -0.94980369161162      0.98298657998326      2.17684813775750
  C          -1.17959601649859      1.87754510745421      1.37749770651689
  C          -1.43952390757303      2.60500911872380      0.27145521467971
  C          -1.65985038837476      2.93789588718631     -0.88028377653111
  C          -1.85133777505282      2.80675717848093     -2.21385559284345
  C          -1.94723966215517      2.22214107992631     -3.27744248275630
  C          -1.93588855414704      1.10694116725281     -4.04997307608869
  C          -1.81850084999694     -0.09524302517529     -4.19988654856724
  C          -1.59546697907645     -1.34578317326403     -3.72369259482537
  C          -1.36279633731088     -2.14146622098268     -2.83185290467401
  C          -1.11513928447187     -2.55363549161852     -1.56688024565308
  C          -0.93749701635303     -2.47445397107063     -0.36309478438861
  C          -0.79635334413659     -2.00289292880554      0.89221189060966
  C          -0.72788297486765     -1.30167945878298      1.89104584288119
  O          -0.47592867418414      0.54883096496469      5.01586053748515
```

```
  C          -0.48543734546286     -0.36286624833930      4.23565686181563
  O          -0.27369959022566     -1.69654096121258      4.60508735185252
  H          -0.13416407537788     -1.68500674331113      5.55482247459579
```

```
Structure: C16O2H_c.xyz
19
Coordinates from ORCA-job 15_3 E -760.224897250316
  C          -0.69082717908333     -0.41461600945707      2.85723776753541
  C          -0.92155638880905      0.88781763139838      2.61728262155878
  C          -1.18467029998747      1.79574099588383      1.73641460907603
  C          -1.44259755919731      2.51811470600048      0.74094093204028
  C          -1.66166722077243      2.70194794448992     -0.55059583835045
  C          -1.80818112580418      2.48396561885785     -1.75409199042641
  C          -1.86765398450864      1.75878512369069     -2.88391871104724
  C          -1.82440090492675      0.77260221239835     -3.59957701919318
  C          -1.67673259925481     -0.55473891056942     -3.94245792183165
  C          -1.59892972730020     -1.77888667239218     -4.64376626213305
  C          -1.40251543214919     -1.67863023192406     -3.24357460492588
  C          -1.15490646652211     -2.00626555965328     -1.92641901730429
  C          -0.97839827897472     -1.98629615992472     -0.72640159630229
  C          -0.82650590047463     -1.69932723244628      0.59317573420224
  C          -0.73045520174324     -1.24689980647406      1.71258296203684
  O          -0.21389099427667     -2.14581390978596      4.38523553471350
  C          -0.41816797667622     -0.98016323849921      4.18641544997739
  O          -0.40846609269934     -0.06037216283020      5.24182544343319
  H          -0.59066668270750      0.81874565255352      4.90189190120141
```

```
Structure: C16O2H_d.xyz
19
Coordinates from ORCA-job 15_4 E -760.202334784357
  C          -0.68810984176104     -0.16927937519632      3.15400092414105
  C          -0.99497040949943      0.79400306889488      2.17917105973077
  C          -1.27958220895066      1.89674687126720      1.53050966038790
  C          -1.52483404035880      2.27101864619190      0.29534091578245
  C          -1.73504641419183      2.47123530209857     -0.91534958697070
  C          -1.86940528554098      2.15654464838432     -2.20370613800804
  C          -1.92961779623983      1.58447255021292     -3.28770291699711
  C          -1.86946260488249      0.51316404321016     -4.13832975137117
  C          -1.88151788793154     -0.35515165851533     -5.26739349807126
  C          -1.61756656231863     -0.80827447469162     -3.96440374679203
  C          -1.32480709990635     -1.56377183571889     -2.84263377170126
  C          -1.10415857181567     -1.86948545374403     -1.69039037649940
  C          -0.92239820945352     -1.78556876788334     -0.34599057617787
  C          -0.80835233890767     -1.46128109214245      0.81719087912133
  C          -0.78944026578443     -0.63301379956314      1.91632858901101
  O          -0.43836483959312      0.36242882798474      5.45161105633015
  C          -0.43102140151536     -0.45477762732653      4.57290764651613
  O          -0.17575960782751     -1.81971242868583      4.75121747132670
  H          -0.01677461351363     -1.94358745346062      5.68982215450202
```

```
Structure: C16O2H_e.xyz
19
Coordinates from ORCA-job 15_5 E -760.200097307148
  C          -0.96358661300018     -0.11091951095241      1.33212444864125
  C          -1.19781282779756      1.24675564507595      1.28086915172910
  C          -1.43108898584980      2.41572883223867      1.01731784520639
  C          -1.71929190552219      3.59389573913919      0.39390259715500
  C          -1.92533221437976      3.76471552451968     -0.80995239561471
  C          -2.06285823812097      3.43213929777373     -2.11486669530296
```

```
  C          -1.92379903557786      2.26393814704670     -2.49351013121569
  C          -1.70021141090054      0.93977930582525     -2.47400603067412
  C          -1.43766241526823     -0.08186338559126     -1.85992877805757
  C          -0.63904566133961     -2.35812078601541      0.98512932537167
  C          -0.50218769951357     -3.61812542392818      0.47775276229399
  C          -0.73969809435950     -3.30378569548767     -0.77904947386712
  C          -1.01443623651872     -2.49632257219720     -1.73630392573915
  C          -1.13422240653505     -1.22461991668150     -1.09920175982426
  C          -0.93888401541446     -1.14029973776985      0.32965083501814
  O          -0.48194348644692     -2.03971685108392      2.41387447877042
  C          -0.65741439085484     -0.86145143969337      2.55844426035225
  O          -0.56906213336571     -0.27102678640130      3.82477463238934
  H          -0.36265221864347     -0.96499037862518      4.45517885292045


Structure: C21O2H_a.xyz
24
Coordinates from ORCA-job 20_1 E -950.693987560160
  C          -1.32184683497132      1.03533019175699      0.11706846765807
  C           0.39358007736170      1.81563919914135     -1.17602163615541
  C          -0.36600551295138      0.64300803385552     -0.92751595182012
  C           1.58786635171734     -0.43639615464955     -1.79629193525283
  C           0.40654247561814     -0.41198074727023     -1.42225255086674
  C           4.37485882612328      1.88435976212209     -0.92931003833130
  C          -0.99085593158434      2.37096369297790      0.47621026706313
  C           0.06115764509956      2.87869458262377     -0.30232503197665
  C           1.77909578442880      1.83385743445264     -1.44609429134417
  C           2.33506407884188      2.93983213714477     -0.71092798054907
  C           2.49797713209180      0.64050099760357     -1.73334722737218
  C           3.86511153337431      0.91654697476835     -1.48804033149922
  C          -1.37052784504385      2.64774417908163      1.77458761847394
  C           1.55726548020516      4.21112433849565      1.29338348273100
  C           1.28298440646641      3.54763825887858      0.07039383671613
  C           2.94186613216139      4.38099218388334      1.41649583361979
  C           3.71001339377500      2.98132949163178     -0.33498790163172
  C           3.83794439198304      3.87344291556294      0.73994872856504
  C          -0.53545086107768      3.52111366575728      2.45949024309698
  C           0.43529434529875      4.22047698659058      2.17796301894769
  O          -2.00445836788027      1.45244459984576      2.22936054169009
  C          -1.96093943419530      0.61395117985806      1.37201880267739
  O          -2.50344626455336     -0.64828515875896      1.64117631833091
  H          -2.77531100587959     -0.66370873571914      2.56174770457747


Structure: C21O2H_b.xyz
24
Coordinates from ORCA-job 20_2 E -950.740953338204
  C          -1.37211271329142      0.76672391489449      0.64599220102806
  C           0.82075387004335      1.62123153617514     -0.84626171453328
  C          -0.71526846477640     -0.02505313773316     -0.24767910566029
  C           2.66025555966834      0.04305017335598     -1.87470689056510
  C           0.44376989588450      0.28135244752017     -0.98672503349453
  C           4.38442940512756      2.04032356817477     -1.32265906283346
  C          -0.85413919654555      2.09207970364271      0.89060344868590
  C           0.18639609536875      2.49913068210952      0.06337250368185
  C           2.12263498540958      2.12992037117669     -1.12900450644544
  C           2.28842934862351      3.32934103695036     -0.38018919327512
  C           1.53238753830229     -0.37684304164847     -1.62427009913692
  C           3.18981630910827      1.32861816273803     -1.58463872344478
  C          -0.92814283490158      3.06165344649416      1.92254199356039
  C           2.29076309770945      4.94652777884206      1.69061751625680
  C           1.09074019764443      3.56037037473503      0.35691256449310
```

```
  C          3.33012172039368      4.75377702911320      1.05941061689697
  C          4.52646558911057      3.07724383552000     -0.67414044512229
  C          3.54620233845144      3.85691680968992     -0.01382803989862
  C         -0.17183667930955      3.98563590708806      2.21764296249468
  C          1.02311627774560      4.33481075175239      1.53310310684314
  O         -3.09378180933643      0.90780955460899      2.27533216297743
  C         -2.52400641748387      0.28540670258526      1.42212017030723
  O         -2.89585237360885     -1.01345347147275      1.05511494298898
  H         -3.64336174292819     -1.23795413196963      1.61406860625174
```

```
Structure: C21O2H_c.xyz
24
Coordinates from ORCA-job 20_3 E -950.708136679547
  C         -1.04953004678511      0.84251710291915      0.96944814693149
  C          1.19129380768286      0.93149637781570      0.52685931476529
  C         -0.00969904568490      0.25149749727480      0.20758783008119
  C          1.24551796602559     -0.32718711458651     -1.77480003436234
  C          0.30829736108592     -0.45389634275035     -0.98783209976860
  C          3.23174875542736      1.38365793080189     -2.38372528799130
  C         -0.49573688763754      1.99311345751512      1.61909980517514
  C          0.93381458164356      2.03764443398658      1.31097422856985
  C          2.26088786209396      1.18631874099033     -0.36148295744587
  C          2.74799884244513      2.48281031357412     -0.04559537193309
  C          2.33968825195539      0.59072973973383     -1.63767868611130
  C          3.74964800034462      2.47061464327506     -2.11087673962307
  C         -0.99267215913277      3.14136841025308      2.20082718207330
  C          1.84799091713698      4.45910140542037      1.21108797856051
  C          1.89477528515368      3.05112985150704      0.98253443493654
  C          2.82774660153910      5.08473529918019      0.39261730422864
  C          3.50939448739650      3.24760684189396     -0.96529474006878
  C          3.49542558977413      4.59171047860776     -0.50780627831185
  C         -0.27839937324427      4.23121120106134      2.30440699076358
  C          0.73701558833206      4.88408338659815      1.94121177750765
  O         -3.32898619420038      1.08163771672263      1.62165615751555
  C         -2.47697254471138      0.49422631542994      1.01418551145946
  O         -2.75216389708054     -0.63394195419061      0.23219697016105
  H         -3.69930375844225     -0.77356572339890      0.30312853965164
```

```
Structure: C21O2H_d.xyz
24
Coordinates from ORCA-job 20_4 E -950.662066743268
  C         -1.32864232756883      0.91117717813845      0.02528122923624
  C          0.38649197802668      1.78870997658938     -1.19881269598397
  C         -0.34644583062596      0.58820574979659     -1.00040847455736
  C          1.64445374782044     -0.40931387972225     -1.89237525572470
  C          0.45893399355324     -0.42524241791373     -1.53613512975349
  C          4.36772067627331      1.95286985574111     -0.91987089349129
  C         -1.03826749860928      2.23323083020791      0.44541118445629
  C          0.01571744507037      2.79670772804157     -0.28652213798687
  C          1.77425439509808      1.85204878154244     -1.45437193009943
  C          2.29397199104107      2.94109401635134     -0.67347864312455
  C          2.52503083293725      0.69085419237175     -1.78403801298181
  C          3.88077431448498      0.99491256910783     -1.51605454041091
  C         -1.44607819805338      2.54301065368871      1.73875182792842
  C          1.47699230747760      4.10235825192092      1.37417782080423
  C          1.22046816900304      3.48250596328044      0.12553220182852
  C          2.85462319821955      4.31383342620736      1.51890272980996
  C          3.66749523028655      3.00422847265441     -0.28858176594591
  C          3.76070582008924      3.85444821394842      0.82256412018159
  C         -0.60077552334964      3.31194052971775      2.49829682769349
```

C 0.34552126952018 4.06620327853998 2.24143170023050
O -2.32751197583680 -0.84492407084022 1.51007679840668
C -1.96736011942547 0.24854162082843 1.17151591559786
O -2.12810780339441 1.26867963326425 2.11675589331542
H -2.25218610621190 0.98253945617179 3.02468121262754

Structure: C21O2H_e.xyz
24
Coordinates from ORCA-job 20_5 E -950.638643148890
C -1.33400115811862 0.79137424293041 0.42206952821270
C 0.55493961979362 1.73032878845428 -1.25556256329925
C -0.78839719673856 -0.10601274828739 -0.51483875605648
C 1.54231094996765 -0.27289683398096 -1.68440374758836
C 0.28109741032376 0.28255595938187 -1.27225754700637
C 4.46829603712761 1.95038673343936 -0.98137657378384
C -0.82407277463388 2.12308742458874 0.59722872146617
C 0.18321567339241 2.59846017886286 -0.26174531212214
C 1.91124295969872 1.94696459229685 -1.55740159917145
C 2.41747495313466 3.01243046021345 -0.78247094321817
C 2.56728295322324 0.71270262200120 -1.77035370434221
C 3.93487749295052 0.97753975570551 -1.52159432444240
C -1.25569667967224 2.61188803139441 1.80854783864737
C 1.59571293847388 4.21789789394046 1.21472551297329
C 1.35011646260614 3.48587392326631 0.03512178042402
C 2.97664698887389 4.46655097790183 1.31998454078358
C 3.80481006912289 3.06382651007951 -0.40979530213245
C 3.89057038300209 3.95703519667570 0.66793117402235
C -0.50348647811322 3.61509571564380 2.40700749589658
C 0.46941027029744 4.28240273982933 2.07834714001042
O -2.06202225267980 1.61158511742671 2.38335960592207
C -2.13262170939762 0.66462306890876 1.64968487134929
O -2.91540796479607 -0.40773651626480 2.09385892206361
H -2.89451895142904 -1.06734383006533 1.39666322344813

Structure: C26O2H_a.xyz
29
Coordinates from ORCA-job 25_1 E -1141.211530742094
C 0.32286898980373 1.90728652060559 2.08226645649220
C 0.61901219395019 1.92315908415880 -2.27251664292411
C 0.12001508915938 1.02414926602414 -0.05878502473279
C 1.54328583933048 0.96173099402144 0.23195487220486
C -0.56170971573172 1.67759170379884 1.08309990346655
C 1.67049614406020 1.65648524066850 1.50353395113016
C -1.32251192340535 2.85128087884386 0.52255035837981
C -0.31811008329382 1.59276833635999 -1.24188974883790
C 1.96804917201627 1.62667075362165 -2.07400753117834
C 3.21027569620967 2.57430687299769 -0.70454159028570
C 2.44485438725895 1.26787349418991 -0.78361547455539
C 2.37462174813130 2.84372837394939 1.57634173110278
C 0.52888257637594 3.36407201461302 -2.43878782189298
C 2.72863621653200 2.93061609386855 -1.99396625205987
C 1.81565553149971 3.97399240132757 -2.14417154569565
C 2.23518497428435 4.64452692808750 0.16610932421982
C 2.98017234760230 3.41222276282723 0.38097716376017
C 1.46966755476626 3.83446616338637 2.20213639932521
C -0.99674303342349 4.02012969915430 1.22853783073198
C -0.47811875413410 3.89498346211697 -1.52538312596329
C 1.58421118140773 4.85214574043053 -1.03726940653191
C 0.11964422792552 4.89706481262871 -0.72528885573317
C 1.26943158314170 4.80607797413481 1.28072034916345

```
  C          -0.09728036008382      4.93625234060345      0.66100958143695
  C          -1.10062755747797      2.81159619445941     -0.85996962838405
  O          -0.77449388810001      3.79012373005169      2.58385599741451
  C           0.21637706254276      3.16311461180826      2.83887438396223
  O           0.33624168238706      2.92622332025814      4.21347068567568
  H           0.40323112175800      3.77518024352886      4.65662366029072
```

Structure: C26O2H_b.xyz

```
29
Coordinates from ORCA-job 25_2 E -1141.194446921409
  C           0.46256603407739      1.95183105390810      1.94055125290548
  C           0.59206980278634      1.01974759655159     -1.43849072543807
  C          -0.11227683340879      1.34167011080366      0.83382750960520
  C           2.15826977828466      1.22453079381469      0.42484331720713
  C           0.95017529964620      0.77779051290602     -0.07391551172647
  C           1.87786975050916      1.85838875256315      1.73820200505042
  C          -0.99244034170166      2.07297448573974     -0.03072436078465
  C          -0.63235719170973      1.82651885835735     -1.40178142067098
  C           1.48796005416822      1.69389820043256     -2.24707115265800
  C           2.95578371329504      2.15881745400958     -0.35902877968252
  C           2.62174284098631      2.37478418802875     -1.68425218996053
  C           2.63176354151943      3.03206352307272      1.88112537411110
  C           0.88085493753838      3.00994337395928     -2.64282936756157
  C          -0.37241817266743      3.10869700070103     -2.06712602787405
  C           1.79499819434607      4.66280412776933     -1.10164874682742
  C           2.54752879843453      4.53341486190702      0.14678743403925
  C           3.11080486558308      3.33294477700345      0.50054116597315
  C           1.91569142917177      4.11573639304412      2.38852250845155
  C          -0.53984304081699      4.06666884927928      1.29932534089170
  C          -0.59683362958086      4.12075785964451     -1.08118119720054
  C           2.00521380709390      3.70437522265088     -2.07878922292495
  C           0.43079435418364      4.95772358447886     -0.68870550112296
  C           1.71452216745514      4.97339362343940      1.30664933209783
  C           0.40654263678611      5.01270983902834      0.79952326912615
  C          -1.11710098135525      3.48361923311520      0.18257560804514
  O           0.80135425683460      3.79832069592202      3.15225662456454
  C          -0.04247450041373      3.18707424573847      2.55699966957180
  O          -1.11428970873983      2.86034923571559      3.39632255209128
  H          -1.51525184722894      3.67827155364871      3.69936124068292
```

Structure: C26O2H_c.xyz

```
29
Coordinates from ORCA-job 25_3 E -1141.203156454786
  C           0.17178171564434      1.94316249329321      2.17464864864832
  C           0.77708306211354      1.89769233103192     -2.08996345744981
  C           0.02960116481405      0.96063740781048      0.04069229909152
  C           1.37792186583983      0.82133603378211      0.44200249450210
  C          -0.67864819827778      1.72182951679685      1.11508370900778
  C           1.45795218823221      1.46715247713007      1.76950783394427
  C          -1.31227738132537      2.89468893812878      0.47513278027212
  C          -0.24949231800348      1.56612516381530     -1.19378291372649
  C           2.13273583234927      1.79419292258826     -1.65718017690189
  C           3.03758275214691      3.59648778620197     -0.47112887129345
  C           2.41189421650050      1.31767731143819     -0.37099830094086
  C           2.32344230820313      2.59554161177475      1.62982113585875
  C           0.56661293396555      3.32523592538075     -2.45815657376936
  C           2.72362836576785      3.15796879001240     -1.79726846155117
  C           1.71989341488050      4.04973998196660     -2.30809100150050
  C           2.20603148323370      4.77213346419651     -0.15199660527360
  C           3.00323147279616      2.49280832150696      0.40041958069052
```

C 1.52600122311445 3.78259041632995 1.97881502923839
C -0.96529942087461 4.09936922534812 1.13353358251322
C -0.51824184592870 3.87065123979523 -1.62457802524896
C 1.43432909737079 5.06728164798586 -1.27950918151813
C 0.02448485754644 4.96766266491073 -0.88262223886840
C 1.36690717945837 4.76218769249019 1.04040202957895
C -0.07940542921636 4.97789872158230 0.51514505051020
C -1.07061597982984 2.79297054709234 -0.91105128335993
O -0.73318183336451 3.96236233377527 2.49214314052017
C 0.22105465997297 3.28816051567661 2.76579346309470
O 0.38646492987031 3.33276575748480 4.15527171190729
H 1.01974766103416 2.66150878907394 4.41978459671439

Structure: C26O2H_d.xyz
29
Coordinates from ORCA-job 25_4 E -1141.182955519556
C 0.47361610401842 1.95098242259725 1.82425544509182
C 0.38103861373174 0.88603638682773 -1.45263271667132
C -0.26012438558426 1.26512683984815 0.79838239300808
C 1.94177845412923 0.99420268517229 0.29437028108851
C 0.65456794758818 0.51613143140255 -0.03012057918183
C 1.83143489353543 1.83508208663416 1.51410703921321
C -1.06159570116277 2.07869112431809 -0.08457666784595
C -0.66099654646527 1.82280287186728 -1.45980723582819
C 1.51879722387659 1.50454923468264 -1.97481654094045
C 2.98647619261082 2.92131350740307 -0.85500159657471
C 2.49624555062632 1.59430858519248 -0.90059609665945
C 2.60892330100307 3.04891540791487 1.65549567267547
C -0.18227214974269 3.10761499241920 -2.04047415389508
C 1.18718131089888 2.91245148777748 -2.34578629755515
C 2.16326549239685 3.76209896721113 -1.79496707097128
C 2.57257465692145 4.90188144073458 0.21977838666775
C 3.13072018371153 3.62132545644501 0.36565566049988
C 1.70785951322551 5.09202841580397 1.33796624480502
C -0.49735595204195 4.06662426204808 1.25925934387800
C -0.50021670280561 4.11972052624468 -1.11498180254554
C 1.78975910537040 4.89397081719764 -1.04463741407693
C 0.43840046657570 5.06914196933119 -0.68537474353100
C 1.94950904353987 4.06618008412648 2.31015741713383
C 0.39097027067273 5.07927483926531 0.81519147635731
C -1.07414972666972 3.48873076949563 0.11603212149359
O 0.86000100438286 3.73164164564643 3.10207594070709
C -0.00855730911248 3.16518001556982 2.49814910023596
O -1.07536867904887 2.81727202829527 3.33533100227185
H -1.45126217698050 3.62653970046920 3.68943538583966

Structure: C26O2H_e.xyz
29
Coordinates from ORCA-job 25_5 E -1141.164015236187
C 0.30290468417855 1.98574127301234 2.26750059833542
C 1.39558900882556 2.34956614244662 -2.67915804577288
C 0.10666341694457 0.88941716296834 0.25081106862776
C 1.48012700648617 0.92398087075013 0.47654204241488
C -0.57443873388709 1.65775039518447 1.25645498190159
C 1.64570421456302 1.70162635916983 1.70185116517901
C -1.18257484216478 2.78533882350161 0.49379385753400
C -0.14738970669877 1.38177041916693 -1.09158762715896
C 1.07771866612555 1.31529645775232 -1.81858061608379
C 2.89326418203917 2.50699591298375 -0.84697919865565
C 2.11134763214709 1.29533405943509 -0.75506277293976

C	2.30130103254285	2.90239137745544	1.61686914085185
C	0.68687544706257	3.55192374807348	-2.66397749784886
C	2.60330495759532	3.07509655210060	-2.11093787798480
C	1.92769077479993	4.30805728056426	-2.23148596912155
C	2.22724577318771	4.64622315980655	0.15225588178889
C	2.82389264391231	3.39470699009095	0.32219215802630
C	1.42939119839354	3.93160234283328	2.23872468604390
C	-0.98363020314940	3.98329923284180	1.14262696933477
C	-0.31159956644293	3.77211721378077	-1.65910880846686
C	1.62411315899625	5.01681733806435	-1.09240212341382
C	0.19122344618956	4.83759345571413	-0.81881620179132
C	1.24303979529470	4.84305242263944	1.25010432913325
C	-0.06752060784144	4.90149045050542	0.56241366269984
C	-0.81609210776017	2.63045098181159	-0.92805294675536
O	-0.83813748159688	3.87229149202332	2.51322304683335
C	0.15116229963613	3.30811415395309	2.89136304515270
O	0.12961648029424	3.33556389477358	4.29092808755507
H	0.88042743481990	2.83621005970526	4.62036496985474

Structure: C31O2H_a.xyz
34
Coordinates from ORCA-job 30_1 E -1331.765176437095

C	0.58797508443311	3.43433856764903	-2.13982413087744
C	1.66865252777095	1.34999253541441	-1.42695363376128
C	0.72397613974885	0.60390635562245	0.55806242558399
C	-0.82455568856635	1.53810620820382	-0.95153659728105
C	2.46167306517242	1.41324828390748	2.02415461562846
C	2.13447868025085	0.77261257342476	0.84960087091869
C	-1.10500646927924	1.96517889975026	0.38827753794407
C	0.12047019010733	2.10545142089090	2.41599746106547
C	-0.24320683023928	1.23728520989824	1.31422953138029
C	3.25238848019679	2.63999162096822	2.03322691875534
C	1.70490930106695	4.52365937830056	2.50824884662717
C	3.45272126844950	2.42994469739425	-0.37148762560718
C	-1.08833763626893	3.30803644236300	0.76165628139992
C	-0.45647864247563	3.39031961560232	2.11251678728688
C	1.43516965070271	2.08792669361801	2.82988109180694
C	2.34004023803273	5.22269044385955	1.39005483880918
C	2.23978467087516	3.30763759239366	2.86444451514569
C	3.24945117872944	4.55612382730571	0.56308918121114
C	-0.60052056529667	4.45565550944895	-0.00162590263258
C	1.28533282922450	5.72052351932400	0.54046361477929
C	0.26423016873910	4.56013053055218	2.22990647098673
C	2.86926982103897	3.31895216980140	-1.37571650827633
C	1.77411213718156	2.69100059843046	-2.02042869423469
C	3.67411270626185	3.20191016883486	0.83821420177953
C	2.74530543538993	4.58820421906496	-0.83247301193114
C	1.46865651449547	5.19666219161512	-0.79944571749906
C	0.06966246291247	5.29555764440109	0.99642997085096
C	0.30876380671244	4.46804081895905	-1.20617011333796
C	0.48259659332427	0.93152745609034	-0.85111468209040
C	2.72137123334178	1.26227950185249	-0.38197445765263
O	-0.52300534363759	2.77416040827576	-2.84038624934286
C	-1.25721026473051	2.28493278032333	-2.02699861114093
O	-2.62780395565087	2.53471633671286	-2.16513295990196
H	-2.73233876124995	3.23856578249946	-2.80954620744647

Structure: C31O2H_b.xyz
34
Coordinates from ORCA-job 30_2 E -1331.746138215821

C	0.47772735218531	3.66609453208078	-2.20100760356231
C	0.85017605238557	1.45087455291704	-1.55630299901361
C	1.01084383998203	0.49720966383347	0.71542846263495
C	-0.59942484229777	1.67956258097843	-1.23910423858081
C	2.41066859270190	1.81206461919438	2.26298876000496
C	2.30441477826660	0.82287955766370	1.27727205608183
C	-0.85684707824742	1.87304319182004	0.12598209571135
C	-0.03904713058334	2.00763211528917	2.29628195795589
C	-0.13333051375093	1.14509021660377	1.13987723126344
C	3.24716744751989	2.94610109824616	1.98354992195037
C	2.52703363477934	4.18782035958348	2.40872104577153
C	3.37557980142486	2.28289109542398	-0.40407736423264
C	-1.11217233452801	3.22991971228137	0.63672085118812
C	-0.74784687805869	3.22278942808754	2.00987820989216
C	1.19899762291614	2.33320427452517	2.85212569581065
C	2.62568378417444	5.12835143212519	1.34052442704137
C	1.29364110491067	3.81399422242612	2.90906323805900
C	3.43951260973876	4.49964327310292	0.31850403105117
C	-0.53772819343301	4.43514446145319	0.06157082389054
C	1.43532694536337	5.53966951296291	0.70761664603351
C	0.08884365525554	4.33682919091540	2.32702098650312
C	2.68729872523427	3.10788120539090	-1.40486415862678
C	1.50681057449766	2.68234350416879	-2.03286681669174
C	3.75582683642931	3.16342597852888	0.69177066890114
C	2.73819390152594	4.46969839479189	-0.92250682498702
C	1.51721277052353	5.14968371841639	-0.70578011371647
C	0.17716262564188	5.13552315855112	1.16581836926171
C	0.29746385492385	4.58233101253180	-1.14753297718862
C	1.52273129053188	0.69048097114579	-0.65687471225276
C	2.80232524577035	1.08377157732615	-0.07582720224801
O	-0.71595666620474	3.19393147097965	-2.91730391474379
C	-1.29528365395196	2.49128510805144	-2.13585127590037
O	-2.69516590973436	2.50855794398910	-2.13025459423987
H	-2.98119982442053	3.23954687265857	-2.68292066641040

Structure: C31O2H_c.xyz
34
Coordinates from ORCA-job 30_3 E -1331.738627460667

C	0.54434009979572	3.72918309843284	-1.97757211683702
C	1.86911106465373	1.09712325087429	-1.28375778079966
C	0.76020109564263	0.61061423624217	0.64018716725652
C	-0.64172874658764	1.62177542966471	-0.96264433993833
C	2.14474489698008	0.82214929185099	1.05659574197830
C	2.38094951783165	1.80717055526175	2.08719507032564
C	-1.19895470051095	3.29632051454245	0.84213659564768
C	-0.04600123359811	2.04322286198857	2.43963196219393
C	-0.28639434997047	1.20702490763988	1.30143490260144
C	3.22831486868427	2.92134464165922	1.77114618576556
C	2.58714928007617	4.15748983181870	2.27820108328288
C	3.67357938482981	3.17243000222302	0.42610636869124
C	-1.07693680012917	1.95288933078431	0.33170173186817
C	-0.73096762262252	3.26106616168238	2.21984413076671
C	1.27829700591363	2.33587070062395	2.83412860534080
C	2.59662048856120	5.10954487608160	1.23625820923262
C	1.40834251514595	3.81130643374709	2.93583730482185
C	3.23377624202687	4.48115634688236	0.08343566602583
C	-0.64834373436572	4.47532653653075	0.24977169586949
C	1.35014056007360	5.60844287091093	0.77561814413860
C	0.17338652918089	4.34596939823594	2.46128588045051
C	2.54900849407123	2.32387887632755	-1.67165371713312
C	1.96084142725099	3.56061424409334	-1.90544097036848

```
  C          3.47872082275829      2.15497327752063     -0.52799409745892
  C          2.41642362691044      4.66976435995101     -1.07342488806637
  C          1.28059412088677      5.37207508182540     -0.64788075544290
  C          0.14688856537048      5.17835831774777      1.32672025811663
  C          0.08734196249929      4.64770530345952     -0.99458425906436
  C          0.60509525718311      0.88785709226524     -0.80230995491868
  C          2.83265942231690      0.96987329723623     -0.14287527819592
  O         -0.32347731325069      2.92132764983603     -2.84657298207899
  C         -1.05491549265323      2.33389312227746     -2.09829571167499
  O         -2.43093183046341      2.42009951207639     -2.34149830244897
  H         -2.57123537656061      3.10142859575023     -3.00309152804431
```

```
Structure: C31O2H_d.xyz
34
Coordinates from ORCA-job 30_4 E -1331.730923415766
  C         -1.07520073195944      3.24333108736614     -1.39424095574913
  C          1.19194887892287      1.25525178629722     -1.22838553750457
  C          0.22796284340479      0.83991985515810      0.88521085836053
  C         -1.08980085511122      2.05558182934726     -0.60492869154827
  C          2.26563106662201      1.48654772774549      2.04354538458709
  C          1.61246215193950      0.76819005493458      1.02650748411230
  C         -1.34002302268146      2.51146810499162      0.76069195123217
  C          0.13805342516807      2.48681557292782      2.73679810500255
  C         -0.51274526902997      1.78049858022656      1.69700412645787
  C          3.28152639874039      2.37312614736537      1.38467547834839
  C          3.19468221439130      3.63566478670336      1.94973379630211
  C          3.02825405989374      3.34208460565062     -0.80915998797585
  C         -1.30298024235243      3.90445020849711      0.74252412942244
  C         -0.02276865308920      3.94411294464399      2.79280610373361
  C          1.55188113642075      2.23197581540891      2.97195449929505
  C          2.82168788656014      4.81399522835434      1.15267633742275
  C          2.22938652645103      3.53424447904546      3.03942787500785
  C          2.77361962488542      4.63505223475438     -0.20185548670171
  C         -0.59526274048544      4.60124313166878      1.72337668319256
  C          0.43935001498133      5.42250991155598      1.01525134937411
  C          1.31237683752633      4.52932544154420      2.92750288932271
  C          1.91668108001725      3.49691380880508     -1.69518232241057
  C          1.22626500179150      2.38151469375395     -2.14905126299485
  C          3.15409563588851      2.17594620306042     -0.03177952663699
  C          1.56822220867705      4.80696148278632     -1.04862330187464
  C          0.34159858310859      5.12052430910056     -0.40672754636872
  C          1.60626156709800      5.34852222366314      1.75437572365974
  C         -0.88264750472273      4.34700291316086     -0.58949875899243
  C         -0.07744819744339      1.07769557958611     -0.54211354847261
  C          2.20282389469126      1.13018145353565     -0.26660848215668
  O         -0.83540391119921      3.22288486113401     -2.84440632365769
  C          0.12929651341619      2.54488083239054     -3.06725992539589
  O          0.12158526045000      1.89511656954861     -4.30731917519643
  H          0.97526834379255      1.46573555391569     -4.39928192987698
```

```
Structure: C31O2H_e.xyz
34
Coordinates from ORCA-job 30_5 E -1331.716658656395
  C          0.67473319308001      3.53646606138067     -2.08742333429005
  C          1.80557157562003      1.52185171345474     -1.17844235970809
  C          0.58241499953630      0.61333344704814      0.59999671930791
  C         -0.74062435922920      1.67207402834728     -1.01875409212113
  C          2.14338594499110      1.57117438530715      2.19239091921664
  C          1.90751524535398      0.75776520765526      1.07028636071721
  C         -1.20348915192587      2.01391436488773      0.27479707871321
```

C	-0.21803027909092	2.03710371592843	2.39715123868978
C	-0.48159694613589	1.21510220413097	1.27015765504991
C	3.13801562116966	2.58544454634317	1.81001786463690
C	2.62166330172653	3.88400717489851	2.17042088927649
C	3.81321006302889	3.40892129936502	-0.36972035102448
C	-1.19265410614291	3.36941349948587	0.71898145171730
C	-0.78812531638166	3.33805016951533	2.11225679342508
C	1.08980219052264	2.20767708415820	2.87854749990716
C	2.75234672750143	4.96522310198900	1.21283100670482
C	1.37650724313993	3.66340246765536	2.82345941157374
C	3.54428834527746	4.72762634933255	0.07892695589102
C	-0.50317097049934	4.47030744655647	0.07792626636970
C	1.51102916777264	5.53352395671393	0.69749423333538
C	0.20090772372455	4.30993750497415	2.33493529070709
C	2.99190853192973	3.61043168475905	-1.56832784488151
C	1.91893516626912	2.80030887418078	-1.89703224683589
C	3.54587936399450	2.31952311809390	0.45668640401285
C	2.73755320432736	4.96776434231339	-1.14846494319046
C	1.48671268455536	5.33272875111707	-0.75878484732874
C	0.28960735239528	5.12525050123486	1.16178164103222
C	0.33079026482764	4.53228518130345	-1.11236100984625
C	0.53740154642290	1.02796436380304	-0.80191467776768
C	2.68849000043694	1.29577483877327	-0.04009023187203
O	-0.35888587968488	2.89556994574812	-2.91312528653621
C	-1.13440141855590	2.39809207312119	-2.14430064398058
O	-2.48463990259200	2.62440654566198	-2.43694003692623
H	-3.00641110589306	2.07685004293134	-1.84572375736349

Structure: C36O2H_a.xyz
39
Coordinates from ORCA-job 35_1 E -1522.389585096390

C	-1.25013105153561	2.08560633273156	-0.44853339360889
C	-1.56836410971922	3.43034982938703	-0.56820212433151
C	1.05385302607384	1.37966341314392	-0.17804276228193
C	1.97291947589095	2.35430192423614	1.84478900807309
C	0.89644422798419	1.70757957601839	1.14420720442376
C	2.84076173988828	4.45292042586154	2.48989399231576
C	-0.58280981599499	3.59182666832690	1.68186865360027
C	-0.12498583464321	1.59512077178499	-1.09410560352092
C	0.35934613169497	2.28674319996598	-2.20472350432468
C	1.82164943080606	2.42536306588488	-2.08902529664331
C	3.22432030348489	2.55122359218947	1.22563242763798
C	3.78319710350767	3.85952804968360	1.64221075844715
C	-0.64671011664353	5.51396814004068	0.15576315303064
C	0.39184514911256	6.16538813315031	0.89072751303927
C	0.99976913056496	4.47968234801678	-2.62843507384319
C	2.20483485080734	3.76649333079234	-2.41887870163435
C	2.24477206630012	1.83436349758718	-0.90172197774521
C	3.32784856753681	2.42999667445619	-0.18383105665956
C	-1.19838861382133	4.24678328852423	0.64475372003159
C	-0.14891324016921	3.54736044311289	-2.52409574604167
C	1.53516005495025	6.66504277842541	0.20559452346415
C	0.85168265275605	5.56994364301893	2.09375182634383
C	2.74295034861034	6.38064839739241	1.01956351379214
C	4.20346018562382	4.52203302688255	0.48426396833002
C	-1.09078643536088	4.13191640957601	-1.67537747895932
C	0.72588966561018	5.64955866283199	-1.87759124377401
C	2.32428149077052	5.71554922645906	2.17692801326193
C	1.78078503666641	6.26987639570615	-1.13974439871618
C	3.26304025074493	4.38299289014482	-1.68291917980512
C	3.91916915696558	3.63122697418532	-0.66799845263142

C	-0.53066079956170	5.41981437347358	-1.22849130032481
C	3.68972967453609	5.78862584167956	0.17200089266341
C	1.70001879976943	3.51479864918441	2.63052479457499
C	0.49369302823357	4.23707902676117	2.38364776687607
C	3.08633934190387	5.70317875034135	-1.17972912743678
O	-1.40251533360474	1.56883585773172	0.91917534297702
C	-0.55722767808520	2.01186731057662	1.64664480526349
O	-0.65824128843392	1.48118705547276	2.93822318889575
H	-1.51894656047723	1.71957202805875	3.29030136344371

Structure: C36O2H_b.xyz
39
Coordinates from ORCA-job 35_2 E -1522.372373951435

C	0.05251855397971	1.50083847646676	-0.15819771717686
C	-1.19521831101139	3.58234148359536	-0.77193441381279
C	1.38741585160529	1.26934498761417	-0.03532124060133
C	1.07695970091459	2.22246155293917	2.21874045749021
C	1.97707796500423	1.63900365328678	1.28866290293981
C	2.50093154645531	4.22140336011163	2.34211337064573
C	-0.77427827257240	3.62137313218137	1.76785962203246
C	-0.46200030921582	2.37890601429065	-1.14235723088802
C	0.36625298676380	2.64875625340310	-2.24244153560249
C	1.77387731654984	2.37563929528262	-2.21065103459020
C	3.18138665278182	2.26575966447604	1.02072746724422
C	3.45225080154442	3.60377097536145	1.47820562113135
C	-0.89950892025681	5.59434386203775	0.36156400006567
C	0.03502195735701	6.25398450144029	1.14332067504115
C	1.38850078753580	4.64775441804422	-2.79313126855953
C	3.44739277237195	4.12398280456123	-1.91160374950935
C	2.29884783526678	1.69518695022285	-1.08292905910733
C	3.44785654336110	2.21912155840046	-0.43388604732655
C	-1.23357556378049	4.21567475036054	0.58104107275781
C	0.17139661917622	4.06149610620513	-2.61504016594352
C	1.14521731540585	6.64739549584185	0.25100390507050
C	0.58955606666180	5.59053922514490	2.24473714740568
C	2.36206158252303	6.17121568603004	0.81851106132311
C	3.91772676829191	4.33057545340534	0.35354430059628
C	-0.79498807025937	4.60252355646021	-1.71516285851206
C	0.79180626740497	6.36451360762106	-1.09574627307337
C	2.05462623661256	5.53517246920994	2.05532085213483
C	1.77988337666427	5.78412501079611	-1.96695773018659
C	2.42239420785698	3.58732414186302	-2.65307923409366
C	3.99113186811314	3.43279608191288	-0.81279891423766
C	-0.51823926776385	5.78264241692729	-1.04823761854084
C	3.32772765089925	5.56142909935346	-0.05011105794640
C	1.35685622214751	3.45545695380766	2.74012668572818
C	0.14242938961728	4.32926137301782	2.57597192716331
C	3.04625265497900	5.48475747911774	-1.44556762399200
O	-0.76719568221635	1.36204973178937	1.05411672558829
C	-0.47661324696693	2.08392746086882	1.96760146572006
O	-1.14836250147645	1.59680130739526	3.09519228395450
H	-1.05629733958258	2.22435963607794	3.81581323257879

Structure: C36O2H_c.xyz
39
Coordinates from ORCA-job 35_3 E -1522.363205568107

C	-1.64136713554945	3.07138201459583	0.21078409474936
C	-1.13933330306641	2.99845315257606	-1.02631783656721
C	0.94876136549369	1.63237714814552	-0.25186567348490
C	1.92890668414260	2.42646782980498	1.82979198031213

C	0.80239998131204	1.86771977676314	1.10371345053550
C	2.76382465859554	4.52327830069772	2.40891831196573
C	-0.65610213918787	3.59479047235214	2.12093108107996
C	-0.08162624949648	1.99089902694223	-1.30285031607425
C	0.58695727628415	2.40689755013132	-2.40991477631174
C	2.03319284599641	2.30850862139108	-2.22440720467061
C	3.18161078985727	2.57634410509106	1.21027851710566
C	3.71166695004201	3.91314192482319	1.53950219854753
C	-0.90268104492896	5.38017964511972	0.45621581559836
C	0.08645445275318	6.10947302433122	1.14832318632840
C	1.47535527441695	4.48393415949062	-2.78219987125040
C	2.59949654454409	3.51545188617873	-2.63337609052013
C	2.26939999015770	1.81859671275271	-0.91452056832415
C	3.35578622418340	2.34561074915766	-0.18792097081758
C	-1.36655669784153	4.19261538428480	1.05191890493688
C	0.28381634915613	3.81390349385705	-2.66377553390623
C	1.12094799498057	6.54162060118077	0.19385235041885
C	0.72212444715688	5.57054474541007	2.24780371642539
C	2.41425208673523	6.28471076228015	0.75090135486524
C	4.09985570339146	4.51591686578618	0.37761826260605
C	-0.71579655507805	4.22309841504249	-1.69837009221698
C	0.74765798876641	6.11991220079406	-1.08498513225163
C	2.18578172000962	5.72374810180116	2.05816933698235
C	1.76855601010517	5.62849822606377	-1.95575458066404
C	3.57382567802140	4.13051215344768	-1.84369018961542
C	4.04557243441781	3.50488760525946	-0.70607796819057
C	-0.53206108558846	5.37932303195476	-0.95789272080094
C	3.41111088014180	5.71265522752691	-0.09024411115464
C	1.71274885200556	3.54668462409078	2.70795389356886
C	0.43604579920304	4.24263411600252	2.66270206220233
C	3.07963275538539	5.47627974933153	-1.44373559389870
O	-1.51956234374430	1.84981002791723	1.01937889231939
C	-0.61306142869044	2.05814897922995	1.77756819879045
O	-0.61110226958857	1.20950908428841	2.89103418126751
H	-1.40741149291910	1.37949049631931	3.39955944302558

Structure: C36O2H_d.xyz

39

Coordinates from ORCA-job 35_4 E -1522.344793956545

C	-1.09313440053071	1.72705172299430	-0.57221506242267
C	-1.37844705774352	3.10689038199706	-0.41073903887772
C	1.12110317332382	1.37563682795672	0.14408930920410
C	1.55317912665725	2.46116189151453	2.08673982957568
C	0.58193524936421	1.71404589027867	1.43009532291800
C	2.75466134018936	4.44574842075746	2.36499323212169
C	-0.64993723682071	4.51145169457685	1.40438691113792
C	0.23600370202593	1.49562484621933	-0.98455353592488
C	0.72242150240796	2.33295245028410	-2.05534048895091
C	2.09047152497260	2.62346323699222	-2.21895964705015
C	2.79964239364773	2.44100687250795	1.27640994046697
C	3.49903581180480	3.66610186205431	1.41253544376297
C	-0.42950597026436	5.59485685673596	0.42439191294867
C	0.60562732700031	6.45992248252551	0.95828998985261
C	0.96546507849378	4.61185908618579	-2.63325855895610
C	2.22773833879540	4.02552070006290	-2.66231350335661
C	2.50819872686332	1.83295380246419	0.06045108560538
C	3.00518315055466	2.44609828251437	-1.12759647616344
C	-1.19720813286759	3.31675439012391	0.98738063301512
C	0.00675705281754	3.55044625022418	-2.33154570974311
C	1.71229458711636	6.80023121259420	0.18390049903410
C	1.02738428625396	5.93160221255987	2.20518207572007

C	2.93312828169375	6.38117222612680	0.91128884330552
C	4.01586442849041	4.28083267269199	0.23566544429110
C	-0.87994725736675	4.02372273699508	-1.35498826973941
C	0.71731613132449	5.72277166965377	-1.77689939967997
C	2.48581550359104	5.76594221499115	2.12588369244684
C	1.81715215939397	6.39137129923053	-1.19261062690309
C	3.30377751263562	4.61008262295171	-1.98612738015975
C	3.80112056238589	3.61828737277642	-1.00497333809813
C	-0.43990835898306	5.33555376565917	-0.93237512529887
C	3.72082350392257	5.69006655398796	0.02386612163760
C	1.45378411632398	3.72971373656319	2.65125548120510
C	0.40351801069570	4.67472981355280	2.41016508952302
C	3.12640863218711	5.81192602041699	-1.32711766314203
O	-1.43876081061941	1.10973344941072	0.71633458636642
C	-0.95405984034938	1.84922290353266	1.52764223921155
O	-1.45780813001094	1.71052527608665	2.82648812403678
H	-1.11801400663418	0.89097429933724	3.19319802528276

Structure: C36O2H_e.xyz
39
Coordinates from ORCA-job 35_5 E -1522.338776725590

C	0.05927074838888	1.08531924100291	-0.22818391514394
C	-1.23365859718898	3.09808111307337	-0.54556297903984
C	1.41111718902443	1.13643217473336	-0.05525237493330
C	0.89303095594415	2.27426034244184	2.05418014368122
C	1.86812730957323	1.59358513156000	1.29590798224540
C	2.53442253158958	4.01886830621893	2.54143176818824
C	-0.61696361551577	4.59838507068699	1.26092784873521
C	-0.55088250296560	1.90736430322530	-1.16647906164133
C	0.21792026629517	2.42606586377515	-2.21713846338543
C	1.62851272798779	2.51589089171197	-2.09095862512964
C	3.12410111246307	2.19343876361315	1.10186477251255
C	3.47717035842626	3.37769087509718	1.75583817966795
C	1.61268091992081	6.56691616200152	-1.46068966474332
C	1.68499058482731	6.96632503785314	-0.05845119605571
C	0.86157258350849	4.65270654757304	-2.66514188763365
C	3.08828905276288	4.56112920881583	-1.59573996424740
C	2.23774842905556	1.82283051171756	-1.02690428551469
C	3.30580538278975	2.42854240535953	-0.32185023459410
C	-1.02039230037063	3.29410533396328	0.79261926050469
C	-0.23859316644656	3.76566017843947	-2.50198185345314
C	0.73735860291608	6.51413951417864	0.82451104025693
C	1.20151477873241	5.82764183112487	2.00612409782521
C	2.91689184311302	5.92902715103406	-1.32379531956588
C	3.94738193435543	4.34738008201523	0.74149104780382
C	-1.03278327600280	4.20595808543534	-1.46474463992160
C	-0.39367874804590	5.72467100699751	0.32056607576385
C	2.51447712061965	5.44819239743398	2.15151064237212
C	0.55755710760233	5.86242142700184	-1.94275690527341
C	2.03622412868139	3.92526233090244	-2.35948697372463
C	3.72283465856315	3.77771691744304	-0.53827861556248
C	-0.55669581686310	5.51056323847861	-1.03310143392091
C	3.44432975835534	5.64149052466929	1.06898420558497
C	1.20840401480209	3.52091829514028	2.61046669579880
C	0.35224096854113	4.66470729047202	2.24633338945134
C	3.05554075556271	6.46578549338176	0.02438714232800
O	-0.71880814270664	1.01093457568791	1.01679003848020
C	-0.60201273454863	2.02833387489300	1.64229584171636
O	-1.43379348073351	2.03800503007873	2.76837188832775
H	-1.14217344085460	1.34126345239823	3.36091634044294

```
Structure: C6O2H_a.xyz
9
Coordinates from ORCA-job 5_1 E -379.435192175518
  C          -3.38299153261314      0.51761596638735      0.90075145033815
  C          -0.30772241511791      2.35181455171970     -0.38165720320294
  C          -2.39399011447182      1.10414855477471      0.49654687535054
  C           0.74458805852520      2.98401833894729     -0.81994957242926
  C          -1.32452283595323      1.74288986674204      0.04509305234637
  C          -4.52991524888220     -0.20225057812353      1.47281626487588
  O          -4.45263925241847     -1.01237870270368      2.35470108798986
  O          -5.71448499097244      0.19480837972734      0.84104200627233
  H          -6.41795166809598     -0.30733637747118      1.25887603845902


Structure: C6O2H_b.xyz
9
Coordinates from ORCA-job 5_2 E -379.435096607091
  C          -3.35764152714261      0.48812771247237      0.94586981510899
  C          -0.29429998563908      2.30736166860610     -0.38312321686366
  C          -2.35600000641319      1.04959451171190      0.53767569670861
  C           0.73036152641047      2.95263320968483     -0.86621311813527
  C          -1.29280102788256      1.69061701805207      0.07409344297554
  C          -4.54766695069072     -0.25548050592416      1.38380395824105
  O          -4.53162959027939     -1.35890934530032      1.85517175371293
  O          -5.69442632626730      0.51649543707313      1.16245337278868
  H          -6.43552610592388     -0.01710969845181      1.45848829875683


Structure: C6O2H_c.xyz
9
Coordinates from ORCA-job 5_3 E -379.435243187392
  C          -3.51910337194288      0.64691445783002      0.78071921906105
  C          -0.59862988484774      2.88282623116745     -0.18731778869555
  C          -2.58949768504553      1.37104915005294      0.46942058924083
  C           0.41837250307840      3.63135859826744     -0.51075700410818
  C          -1.56901949131059      2.14586319686474      0.13227330912642
  C          -4.69182866059935     -0.15854051854574      1.15066202985169
  O          -5.81788649951709      0.25516652920099      1.17964859847550
  O          -4.30292151855089     -1.46710304860455      1.46117023185157
  H          -5.10911539038552     -1.93420459888720      1.69240081849279


Structure: C7O2H_a.xyz
10
Coordinates from ORCA-job 6_3 E -417.523425662937
  C           0.26528618997257      0.09881447052033      0.52276548183355
  C           1.46701108531549      0.07702795603846      0.37361639094248
  C           2.79879204102632      0.05786066974854      0.20669559095895
  C           4.02220583149701      0.04229263438683      0.05253332233564
  C           5.32969698581379      0.02708570962372     -0.11213741029582
  C           6.59806886554796      0.02004069476625     -0.27408739871109
  O          -1.70754866132693     -0.94263090475695      1.15007470650065
  C          -1.15011372263948      0.03822696803840      0.72985709933307
  O          -1.75617180341740      1.18477409316073      0.40371216553352
  H          -2.70389681201672      1.06579770419084      0.57284005834196


Structure: C7O2H_b.xyz
10
-23.3071070359 converged=true
C  0.15059448674041453486 -0.03706633113371810007 0.62710647492584115081
C  1.36530691513999191677 -0.05402678227061538663 0.51480313025256330128
```

```
C  2.68655678798356323256 -0.00172521498954680830 0.32588580472229011775
C  3.88797628981291332195 0.14530963903705479412 0.11820543395727986513
C  5.18678254112178560575 0.32857980511484635722 -0.15691297643816926044
C  6.34777883911104012782 0.03754513953365962164 -0.43349356640835035037
O  -1.77737481870842151110 -1.17855798844073778042 1.18575272466162462415
C  -1.26581426692697229797 -0.16103540431212234885 0.79542936155526033914
O  -1.98047774929015307777 0.92432596919454557138 0.48000263051008096316
H  -1.43799902521151379275 1.66594116927556035179 0.16909098903449215623

Structure: C8O2H_a.xyz
11
Coordinates from ORCA-job 7_1 E -455.591940123942
  C          0.37834901639758     -0.09321602958923     -0.58121784430953
  C          1.64913470488526     -0.11659474941184     -0.32563295576392
  C          2.89118003442704     -0.14036043253009     -0.07549592932650
  C          4.17598602688209     -0.16162225056213      0.17925987909879
  C          5.38325077520114     -0.18797016912540      0.42263052529779
  C          6.70217109476836     -0.20865711527745      0.67950317340396
  C          7.88589951199980     -0.21285552534269      0.93118978574805
  O         10.06763551412279     -1.10259977641897      0.69432779657546
  C          9.33473013109710     -0.28030569223161      1.17043927275139
  O          9.73465850209211      0.74925398886847      2.03070909370630
  H         10.68258469536421      0.64627775075933      2.14220720109894


Structure: C8O2H_b.xyz
11
Coordinates from ORCA-job 7_2 E -455.586066992063
  C          0.48938815046777      0.03950733175449     -0.44532888047108
  C          1.76708505017861     -0.02701802679338     -0.22836692035731
  C          3.01351679539351     -0.08925544222999     -0.01417217529653
  C          4.30393929981287     -0.15089100176158      0.21043771853687
  C          5.51556256325847     -0.21354598175433      0.41984589039427
  C          6.83923651013703     -0.27640335240919      0.64810492621691
  C          8.03151707197880     -0.33498980397735      0.85255963354705
  O         10.17644677448359     -1.27810357162122      0.58074188171184
  C          9.48038641076122     -0.43709774093859      1.07894782498879
  O          9.99117746976571      0.54273071661761      1.93861560472903
  H          9.27732390041627      1.11641687225193      2.22653447840557


Structure: C8O2H_c.xyz
11
Coordinates from ORCA-job 7_4 E -455.546757950987
  C          2.01331843520922     -0.72762848577559      1.39680262272297
  C          2.85469949395018     -0.34290694365592      0.52215775155108
  C          3.76582395916907      0.04374722066007     -0.34728101334937
  C          4.31695450094776      0.54822746950976     -1.55914433680265
  C          5.15567933847176      0.19766846654424     -0.57122317551436
  C          6.37396857546614      0.00525388366730      0.06608921492375
  C          7.41423674485593     -0.15166677053028      0.64288630747942
  O          9.31253515247413     -1.43677583921588      1.23135744549839
  C          8.69298063743714     -0.41285040151632      1.31927168059875
  O          9.07601108479700      0.70107194360787      2.07588564369206
  H          9.90937207387558      0.46720945584314      2.49111786806423


Structure: C8O2H_d.xyz
11
Coordinates from ORCA-job 7_5
  C          2.26486078286474     -2.10535333199538      0.30513307047314
  C          3.30834386224764     -1.64775330298398      0.02857206481762
```

```
  C          4.43880042136149     -0.95844482122539     -0.40398373543917
  C          5.43367395235779     -0.30217636171180     -0.55399899526535
  C          6.62101931910211      0.41405212439533     -0.52021694406285
  C          7.92240185551270      1.00540414698617     -0.80960339982848
  C          7.42756727936771      0.82812432627259      0.46504023762137
  O          6.39530575186273      0.77209642518985      2.60940120061908
  C          7.38512909589042      0.73964078317278      1.93176094616159
  O          8.51540893136314      0.10150715792334      2.45640651565315
  H          9.17306875530693      0.04425285311492      1.75940903223890
```

```
Structure: C9O2H_a.xyz
12
Coordinates from ORCA-job 8_1 E -493.599559424114
  C          9.01021831949961     -1.21597788937478      7.43432965997028
  C         11.73407481229428      0.76708778179527      5.88624303133808
  C         10.61832153167782      0.19405045320451      6.00938760676898
  C         12.99982454789208      1.25678017633330      6.10534907855324
  C         12.41806799880440     -0.03163114959576      8.30230459132580
  C         12.84075945207954      0.63228720201503      7.29674969843053
  C         11.16114738390493     -0.60102311166346      8.26827088702277
  C         10.22945652354135     -0.51393114199734      7.14554832516968
  O          9.31929894065853     -1.69922148059560      8.78778625500358
  C         10.40439645617881     -1.38000806341125      9.18861807363277
  O         10.84156551773983     -1.76737384676776     10.46095095665955
  H         10.13709851027044     -2.28048891413907     10.86349184215199
```

```
Structure: C9O2H_b.xyz
12
Coordinates from ORCA-job 8_2 E -493.583657031171
  C          9.16806990128078     -1.14526450336059      7.43760769219389
  C         11.56380133139653      0.93653923966087      5.34471419000322
  C         10.58345332141668      0.25397713507152      5.83462524821015
  C         12.48598569902293      0.91975238082703      6.31462086421373
  C         12.48067573498104      0.01430019672743      8.27803924549058
  C         13.16411304169111      0.75046368821832      7.36870333882505
  C         11.74076159652163     -0.66000142846172      8.99125467912661
  C          9.80884600644038     -0.45447906185296      6.58762917854270
  O          9.60598967162185     -1.56130623079016      8.77777020530264
  C         10.62743177642883     -1.36035749863053      9.37465850293743
  O         10.65917398988969     -1.93883292740456     10.64916178418066
  H          9.82592791855833     -2.39424097420157     10.79024508229233
```

```
Structure: C9O2H_c.xyz
12
Coordinates from ORCA-job 8_3 E -493.570459194639
  C         10.64883676915276     -0.54926733273589      7.64143759624877
  C         12.41211142343148      0.94085129567253      6.19766683761481
  C         10.04489580712001      0.31385878278843      5.16174859805017
  C         11.22700196330421      0.67194176091890      5.58169852331600
  C         11.95599655442480     -0.24842077902387      8.30992736166000
  C         12.65033464083762      0.52496144876850      7.33783210561545
  C         12.04914957470133     -0.78044923331642      9.55401758939519
  C         10.20380354887786     -0.20592174647847      6.44843397204544
  O          9.98583429284712     -1.34182583428183      8.68698946977018
  C         10.76862734162114     -1.39687099494410      9.59484688272552
  O         10.32559040716379     -2.12019866253079     10.70863113640531
  H          9.44204768164310     -2.44810871549278     10.52579992788870
```

Structure: C9O2H_d.xyz
12
Coordinates from ORCA-job 8_4 E -493.579473939305

C	10.23117204504315	-1.12655970916157	7.56914591985807
C	11.48086243840172	1.28987313861925	5.52091766784501
C	10.49527104918731	0.47370903773016	5.94536968029428
C	12.40782605463647	1.07139761921606	6.36975272337697
C	12.48963701918244	-0.30138680123530	8.10987254136982
C	13.12373821270822	0.38725517285258	7.27809665023771
C	11.44484652720362	-1.13283294870240	8.43591614146005
C	9.75838721987146	-0.34468663738121	6.58267173105955
O	9.50249999131268	-1.61898381637794	8.74704889153033
C	10.51009361122288	-1.50576536673966	9.38887164939463
O	10.57288519913294	-1.77778169522349	10.76075514927339
H	9.69701062134694	-2.05368799366872	11.04061125503568

Structure: C9O2H_e.xyz
12
Coordinates from ORCA-job 8_5 E -493.569120354345

C	9.74954918182526	-0.78502992224160	7.23904501562341
C	11.37308913118911	0.62286289130991	5.83546482245681
C	10.26865807482031	-0.07385347865029	6.22734333278819
C	12.46495141434496	0.88405116333805	6.37782012441588
C	12.75113369744811	0.07363610546365	8.41878930177766
C	13.26621099230811	0.77233761195502	7.42796122875038
C	11.93424821449905	-0.61035409706825	9.06914847767121
C	8.93161564351279	-0.66868122503591	6.16315168571249
O	9.83065184994611	-1.37174630789287	8.58443947852350
C	10.77913556538038	-1.26193915922169	9.31129644968220
O	10.61618396000014	-1.90468888738560	10.54430884417357
H	9.74880227985075	-2.31604468405912	10.55026123386831

S6 C_n-OH family: Energies (Hartrees) and xyz coordinates (Angstroms)

```
C5OH_a -266,06660
C5OH_b -266,06185
C5OH_c -266,00447
C5OH_d -265,99174
C6OH_a -304,12044
C6OH_b -304,10902
C7OH_a -342,22269
C7OH_b -342,22719
C7OH_c -342,18076
C8OH_a -380,30682
C8OH_b -380,28507
C8OH_c -380,27072
C8OH_d -380,22829
C8OH_e -380,20266
C9OH_a -418,37739
C9OH_b -418,37788
C9OH_c -418,34434
C9OH_d -418,33515
C10OH_a -456,48617
C10OH_b -456,43359
C10OH_c -456,41726
C15OH_a -646,90151
C15OH_b -646,87104
C15OH_c -646,85696
C15OH_d -646,84134
C15OH_e -646,83335
C20OH_a -837,37482
C20OH_b -837,34635
C20OH_c -837,34025
C20OH_d -837,31848
C20OH_e -837,30084
C25OH_a -1027,81687
C25OH_b -1027,81800
C25OH_c -1027,79643
C25OH_d -1027,78302
C25OH_e -1027,77965
C30OH_a -1218,46753
C30OH_b -1218,45617
C30OH_c -1218,44155
C30OH_d -1218,43450
C30OH_e -1218,41922
C35OH_a -1409,00641
C35OH_b -1408,99308
C35OH_c -1408,98269
C35OH_d -1408,97473
C35OH_e -1408,95300

C -37,84496
O -75,07371
H -0,50211

Structure: C10OH_a.xyz
12
Coordinates from ORCA-job 10_1 E -456.486170240191
  C          -1.18158428456660      1.33188490288166     -1.10402593241488
  C          -1.85573548108890      0.14378908115673     -0.91951791752305
  C           0.37407958761707     -2.04102621124264      1.85032991515518
```

```
  C          -0.73183979083209     -2.32673147303675      1.23304251567441
  C          -1.63210664384917     -1.98513489468390      0.41302064934863
  C          -1.93664322569097     -0.96555283858907     -0.36299571632219
  C           1.32525854160323      0.15965110148115      1.30190885726735
  C          -0.02597916527763      1.46148317420407     -0.36583946379891
  C           0.79278758580509      1.01525504202140      0.45518334914357
  C           1.12981482007225     -0.98771047737275      1.79890539890301
  O          -1.58694068407851      2.32659689058176     -1.93819273098124
  H          -2.40573126503199      2.05878569087293     -2.36181891916012


Structure: C10OH_b.xyz
12
Coordinates from ORCA-job 10_3 E -456.433586787531
  C          -1.09212677856566      1.08196449094545     -0.90444818399699
  C          -1.77511861774417     -0.10476417058595     -0.72750597177325
  C           1.22029661035800     -0.90899694018680      1.81970280908831
  C          -0.78260048546172     -1.84542715655696      0.93186834583250
  C          -1.67067604860480     -1.42702866253111      0.07717912857167
  C          -2.66939022837575     -1.10409469344790     -0.80182823610906
  C           1.64472002681920      0.24424241616185      1.47951430771697
  C           0.06148141221022      1.25570711030380     -0.19084047109776
  C           0.97562733702778      0.98584749061209      0.59984868601542
  C           0.27589941536895     -1.77352905906367      1.63439600956257
  O          -1.55076014021376      2.03775268910267     -1.75634275304689
  H          -2.37197250284476      1.74961646822935     -2.16154366017994


Structure: C10OH_c.xyz
12
Coordinates from ORCA-job 10_4 E -456.417255680810
  C          -1.57015705063301      2.18815381464812     -1.85089188551960
  C          -0.72135638357757      0.34067367982621     -0.23345698920666
  C           0.73617847775798     -2.89054997224737      2.57489768765861
  C          -0.20908178099263     -2.40894740797727      1.64509936710788
  C          -1.10817748363483     -1.91958960291418      0.74373597008007
  C          -1.31794906716323     -0.92066914245059      0.04416405527796
  C           1.01798785827239     -0.39589678560760      1.39351343004758
  C          -1.17073645559017      1.33694988718045     -1.10049406461786
  C           0.44302551775152      0.34088184352752      0.58277825101567
  C           0.89031128180545     -1.62064939755759      1.98088769069470
  O          -1.95153500999235      3.19858393238987     -2.67744002459275
  H          -2.77312990403000      2.94234916062412     -3.10279349323736


Structure: C15OH_a.xyz
17
Coordinates from ORCA-job 15_1 E -646.901514803504
  C           2.23571926182517     -0.89861031493764     -1.92387384584243
  C           2.00670865871453      0.26285460667915     -1.20509577530829
  C          -0.82754256681796     -2.92880700792863     -0.73485858829333
  C          -1.83149442168252     -2.97288911739705     -0.04532794288594
  C          -2.78576244232395     -2.56277320297693      0.82566079396556
  C          -3.34903338216075     -1.80090578852277      1.58914945633627
  C          -2.21587754068911      1.53441761926192      2.38692027357615
  C          -1.21840627916022      2.06967729250014      1.93955143216560
  C          -0.09604960952652      2.14958071354279      1.18514751303573
  C           0.78704844453901      1.82722286537038      0.40791894358205
  C           1.52941741388801      1.10963342910880     -0.45919269294226
  C           1.28325428042745     -1.88786450455389     -1.72719438410321
  C           0.30737208030577     -2.51120286146778     -1.33550969723329
  C          -3.09736449552198      0.50885448093343      2.51764319690142
```

```
  C          -3.47083771437197     -0.61582194281358      2.24267516912911
  O           3.28613826962315     -1.10085385793201     -2.76370564704060
  H           3.83543005047073     -0.31356241880405     -2.77082820470731


Structure: C15OH_b.xyz
17
Coordinates from ORCA-job 15_2 E -646.871043576711
  C           2.40085483716847     -0.86719602368801     -2.02597014787640
  C           2.20917111704560      0.27037119825314     -1.34403053270729
  C          -0.70362283403572     -2.72776391167094     -0.72709704502305
  C          -1.66117582446681     -2.60639322747495      0.00977305006554
  C          -2.56614554432687     -2.11087164782192      0.88827395260510
  C          -3.08719133387265     -1.31256429366478      1.64076564914912
  C          -2.59125579110454      1.01928262283306      2.40522731872938
  C          -1.44586006377882      1.49138138071987      1.82026410099255
  C          -0.43236711511896      1.53536748517893      1.13018267265831
  C           0.60001156030321      1.35265493461969      0.31146701693285
  C           1.41600180372518      0.86869263717574     -0.49860121492510
  C           1.43021294994698     -1.87571357895630     -1.82630472687884
  C           0.43920475830548     -2.41905730099928     -1.38840871283775
  C          -3.77509718637221      0.88158780450047      3.17532670335951
  C          -3.28865309686473     -0.14374069276997      2.34211069672806
  O           3.43040004315248     -1.12800059158349     -2.87545095585865
  H           4.00423172254102     -0.35908680458899     -2.90844782477813


Structure: C15OH_c.xyz
17
Coordinates from ORCA-job 15_3 E -646.856957801235
  C           2.12195925241563     -0.86142502698420     -1.80805876940440
  C           1.85391208837462      0.23687931735914     -1.09472877259083
  C          -0.83186635673585     -3.17576281194500     -0.85910498319536
  C          -1.82520493241770     -3.16333858965610     -0.16050909082059
  C          -2.73270378678106     -2.66155211994615      0.71464999579577
  C          -3.09300099024186     -1.73272562828730      1.41215890592879
  C          -1.70212474427618      1.67413389314187      2.08473213883012
  C          -1.14644907949921      2.94013484079604      2.31250735889438
  C          -0.55080912935521      1.98956063961556      1.43544506713826
  C           0.37250111202761      1.50275090766876      0.55595278940095
  C           1.07355986887547      0.86878597964616     -0.24731131743541
  C           1.23216524672017     -1.95619349100574     -1.71340427529320
  C           0.30161881924298     -2.66765159775321     -1.40695141005263
  C          -2.55393907493182      0.59135331605345      2.15484227457179
  C          -3.05348989851860     -0.50177443932365      1.97836046640363
  O           3.19682943477546     -1.01073014421481     -2.62779169540682
  H           3.71576218315617     -0.20349506039433     -2.60170868242925


Structure: C15OH_d.xyz
17
Coordinates from ORCA-job 15_4 E -646.841342213808
  C           2.42998207190163     -0.89773282792011     -2.05886745196763
  C           1.84550234204680     -0.02954056318274     -1.23775772329646
  C          -0.83318036419479     -2.24929170131393     -0.41438652596775
  C          -1.86754846790990     -2.36922177264777      0.27112326739407
  C          -2.89215851906635     -2.10862834054603      1.12056162278169
  C          -4.08656225655200     -2.28756130809928      1.87907152537125
  C          -2.20144435536830      1.16625019550918      2.20184426609347
  C          -1.11574768423914      1.83796158662623      1.75436107851858
  C          -0.08986569788070      2.11270133853533      1.15850951560678
  C           0.87636661915870      1.72727714754050      0.28911101932168
```

C 1.55728596252164 1.09667033796127 -0.49519447670800
C 1.26128351602217 -1.35312137679053 -1.45885565021268
C 0.25832166071921 -2.14603983756875 -1.13153851060472
C -2.89000981257465 0.16187937796883 2.20996801248691
C -3.34888787631749 -1.10297897636153 1.92747097642536
O 3.43826503426012 -1.21879599623056 -2.91319987691029
H 4.03711783501191 -0.47087729870961 -2.97314106799704

Structure: C15OH_e.xyz
17
Coordinates from ORCA-job 15_5 E -646.833348975112
C 2.14002649620876 -0.73975185115290 -1.77401454863640
C 1.42744604661180 0.00089438894239 -0.92301478797763
C -0.93846045528220 -2.84170328304338 -0.57697713016804
C -2.02032229113658 -3.16481173225000 0.03422058794934
C -2.92360495246104 -2.74222523494820 0.86549124695376
C -3.52637936238726 -1.96576056704126 1.65123732250580
C -1.82112568740221 1.21062506427457 1.92562475251170
C -0.80188222291493 1.64298456994522 1.40873681608888
C 0.34482065542048 1.79952436499527 0.67485143467705
C 1.49667231743324 2.48491178232930 0.18373780123230
C 1.16485208063034 1.17208187997242 -0.19313470099125
C 1.00301983263512 -1.35433157218886 -1.25401976222357
C 0.10084725662467 -2.29760848428157 -1.05474934854832
C -2.81818735901216 0.36556996522354 2.23501240478951
C -3.37473230500462 -0.72832871050495 2.11816669722770
O 3.20653456025377 -0.89230814676249 -2.60400679132716
H 3.71919539732166 -0.08081244873861 -2.58808197785316

Structure: C20OH_a.xyz
22
Coordinates from ORCA-job 20_1 E -837.374821481011
C 0.45155237862347 -0.50112066290483 0.52264875330409
C 1.84740923129504 1.14752720655277 -0.51956552584193
C 1.72849388791213 -0.15631127683707 -0.04982509365720
C 4.12096759746126 -0.15710933602383 -0.23905287367444
C 3.03888219495982 -0.70702450127396 -0.04261202812103
C 5.29136017455956 2.26444345919136 -0.41742022409627
C -0.46765860397661 0.49298289272045 0.69345759399707
C 0.85166956910241 2.13079206235603 -0.32387388663333
C 3.08969002381635 1.81243440327796 -0.74478028563265
C 2.85196000702861 3.21445542342870 -0.67532307829376
C 4.34799533003259 1.21435315766987 -0.52334644934336
C 5.08769488999476 3.47725544723654 -0.35788235744061
C -0.32482192178388 1.85721451842122 0.38435227669514
C 0.93925190316294 4.57709056093685 0.18924597908867
C 1.46672436856603 3.41344431974382 -0.40749993865356
C 1.99677257745467 5.51319341002058 0.27525120518411
C 3.85207096384359 4.16742238608842 -0.37906462922429
C 3.19396763424545 5.34864667705222 0.03843815873982
C -0.84828145244209 3.12157204765931 0.77259927400608
C -0.34119075038273 4.23972664400957 0.70312227460545
O 0.26963987732034 -1.79184208322882 0.91065140009461
H -0.59002988079374 -1.87753675609720 1.32924945489737

Structure: C20OH_b.xyz
22
Coordinates from ORCA-job 20_2 E -837.346352222841
C 0.63489213484229 -0.25159826491741 0.46607374087247

```
  C          2.43173872246884      1.04435645369726      0.79706454552282
  C          2.07210469958900     -0.24863108995770      0.40402957741605
  C          4.23097325657865     -0.18011026083944     -0.63432986127971
  C          3.20360963956222     -0.76077460758751     -0.27616376361589
  C          5.14952039342336      2.28478442592822     -1.16370054826717
  C          0.14099549728567      1.09059543009349      0.76658506683434
  C          1.29512373403052      1.88559775601225      0.95717300469533
  C          3.52199773440900      1.78350957825177      0.33135279202258
  C          3.09001461419019      3.14707871322131      0.26544066633506
  C          4.50645804512783      1.20910273996847     -0.51877896629563
  C          4.83292985077153      3.47627830288380     -1.17543732906540
  C         -1.17878548853691      1.57106470115209      0.56397278600820
  C          0.83585855111097      4.28468796156881      0.24737977389667
  C          1.69019761465261      3.21763083519800      0.63717275744118
  C          1.67220914413276      5.25609784936788     -0.36567195186988
  C          3.71770608152240      4.09874892367049     -0.57826181078963
  C          2.84270236472991      5.19990058696331     -0.73517596179957
  C         -0.55723226733674      4.04835298827300      0.28186556035887
  C         -1.02141694605175      2.89827515981265      0.52417670253843
  O         -0.17977032439443     -1.29577921749656      0.15686311247936
  H         -1.07770705013737     -0.95755896045146      0.18714009622905
```

```
Structure: C20OH_c.xyz
22
Coordinates from ORCA-job 20_3 E -837.340248591987
  C          0.62738330628388     -0.12780385244626      0.35280480057368
  C          2.09879449849264      0.94168806424929     -1.01463729649466
  C          1.93163304208893     -0.24471927069595     -0.29020859632318
  C          4.31615389603110     -0.15870645545786     -0.18912857777701
  C          3.22971289888331     -0.72445503280089     -0.03209707626992
  C          5.32109662993921      2.29846518712089     -0.22665814769207
  C          0.08417966386261      1.18469605890587      0.09219266559399
  C          0.99104034684758      1.79829060421527     -0.76251154400417
  C          3.27428102118012      1.71659064328924     -1.05505748638270
  C          2.87549179182826      3.10166948146592     -0.87901361969006
  C          4.50514682141065      1.19372014970817     -0.55224681021675
  C          5.01436898041990      3.48475925885449     -0.13107869513993
  C         -0.68567403906644      2.17939417750616      0.77613583519312
  C          0.79585954719206      4.07337339481994      0.16336948910663
  C          1.45151932349342      3.13975483001094     -0.66237226311584
  C          2.95250683233013      5.09613546745432      0.27303502435881
  C          3.73726870836238      4.06835679231425     -0.28336834751708
  C          1.73708869212763      5.07511198613883      0.47297121231432
  C         -1.45944289792202      3.07034559855660      1.57957216382174
  C         -0.37183943827182      3.49821275492708      0.77079883694132
  O          0.12902797575258     -1.08394775792741      1.18167800768818
  H         -0.70147759929555     -0.77932205952087      1.55459043057444
```

```
Structure: C20OH_d.xyz
22
Coordinates from ORCA-job 20_4 E -837.318484643392
  C         -0.14884167630492      0.27241781246109      0.57254242241604
  C          1.55853458253453      1.04340947192300     -0.71175892548666
  C          2.22394095544687     -0.96868610298979      0.10701314638630
  C          3.96743645875755      0.62913100602791     -0.51543649445278
  C          3.37168184602217     -0.62438179645333     -0.18577507866817
  C          5.34606021846633      2.37682720365390     -0.44803464616359
  C          1.11170178170046     -0.09721532571940     -0.04598853440226
  C          0.64444178815873      2.10033977625476     -0.50168399311818
  C          2.87513613907826      1.53001828889180     -0.79053803643628
```

```
  C          2.75035833453369      3.00280046852749     -0.66537710063166
  C          5.30820784382379      1.08002592966491     -0.42658184754680
  C          5.12913725014610      3.63585503181918     -0.31804646166616
  C         -0.39667610426763      1.68988139131074      0.32992226919026
  C          0.83979279340066      4.39499647995057      0.31443292854992
  C          1.34079110715706      3.30106628692689     -0.45527575855947
  C          1.89245233907067      5.32456927805502      0.45820543180232
  C          3.74814259327302      3.96834113234422     -0.33373476541513
  C          3.06137939531462      5.11739203314440      0.14162890661969
  C         -0.87069727104933      2.87610694252604      0.92790275826754
  C         -0.40323896255747      4.02023793332456      0.87404006812872
  O         -0.94133528797432     -0.49842593534685      1.36459910527315
  H         -0.55428613334384     -1.37309730148438      1.44671460087252


Structure: C20OH_e.xyz
22
Coordinates from ORCA-job 20_5 E -837.300844066473
  C          0.26616107688920     -0.42342139038550      0.61193070177320
  C          1.68886422721162      1.37944954788369     -0.47323508804977
  C          1.47015572524578      0.05466447834476      0.00260110898490
  C          3.80444183248286     -0.17550634320905     -0.10973862031425
  C          2.69420492520483     -0.67794009276501      0.05731313080609
  C          5.29518490843692      2.03369892623023     -0.27593736010825
  C         -0.69429990964036      0.51444528290110      0.83920271494407
  C          0.90535685639123      2.64045770054539     -0.37123903562773
  C          3.02491235673180      1.80086240340441     -0.80037863151997
  C          3.02300763589983      3.19906456407320     -0.96783536722829
  C          4.22989887483200      1.12169958246528     -0.43383841047520
  C          5.10126302007861      3.25650755947010     -0.22989925614014
  C         -0.83387989176236      1.73802948417463      0.79194254808097
  C          0.58066555449834      5.08384976571051      0.56098650556305
  C          1.74191378948618      3.68801699136825     -0.73517462927331
  C          1.86283992859323      4.95348271447735     -0.02016999291766
  C          3.98497684435556      4.08798455163322     -0.42062766931965
  C          3.22354772501764      5.17224795304353      0.12667390619302
  C         -0.27097481827449      2.92747102537329      0.41736665255485
  C         -0.36498965882702      4.31305836730409      0.73399715332580
  O          0.15466035202452     -1.71280715735123      1.02984446660659
  H          0.96620864180287     -2.17370590987947      0.80498516710101


Structure: C25OH_a.xyz
27
Coordinates from ORCA-job 25_1 E -1027.816869736505
  C          1.51052930358351     -0.18755629495178     -0.12065297560669
  C          0.33410442415650      2.84296976272543     -1.58237863309287
  C         -0.09077558122086      2.01563939279596      0.54278100243624
  C          2.23077197912311      1.50691823823037     -1.62423364496615
  C          3.16339605567597      1.44041636734647     -0.63546633160836
  C          2.92538024365630      5.04088003873581     -0.07621377837707
  C          0.65238563479868      5.10961496375984     -0.57848170346553
  C          0.90611929216354      1.94589298561940      1.59464175287462
  C          2.25803626641831      1.42518169587472      1.39414324525525
  C          1.69743939916913      2.81532763924817     -2.03119374544019
  C          2.60513922773164      0.50138034440210      0.40504709272704
  C          0.15292225798413      1.57984197720653     -0.82867480688783
  C         -0.54508108029778      3.39370276158391      0.46487096517294
  C          0.17533513194756      4.17977792588530      1.43949408747793
  C          3.49725365196858      3.86528880026661     -0.70679894283533
  C          1.90007388894714      5.11350774486567     -1.15517683635096
  C         -0.16536295478283      3.91702551916921     -0.81108901681482
```

```
  C          3.62986894859234      2.67694474236892      0.00702742349647
  C          2.48144759917047      3.93786153789626     -1.78039135815502
  C          1.04910515970495      3.27618615407276      2.15095393375493
  C          3.10221249760203      2.64533014368014      1.34028916940764
  C          2.27784260374141      4.99064348387417      1.13671882741267
  C          0.84422530942248      5.25803556869150      0.88145587132947
  C          1.10013797334027      0.60974473754035     -1.19196279657934
  C          2.38173812977722      3.73295186167238      1.87633475295077
  O          0.77527848521029     -1.09818002968824      0.57200181513668
  H         -0.04566387767523     -1.25480802767983      0.09964463193346
```

```
Structure: C25OH_b.xyz
27
Coordinates from ORCA-job 25_2 E -1027.817995154125
  C          1.11612917465967      1.78175242869605     -2.16188525644493
  C         -0.00736260787862      1.98127691875429     -1.32116949338693
  C          0.20654216993131      1.37798533442947      1.85784714820669
  C         -0.16778026409560      1.07105924945717     -0.20026328780369
  C          3.37390894024336      1.58827915155722     -0.14552158447175
  C          2.96543684541819      4.49748136853855     -0.64277108759796
  C          0.62997740879278      4.32045999693637     -0.98643737155816
  C          1.12531793942001      2.24284703905243      2.44402796307821
  C          2.42285876571424      1.87769511362500      1.89005299251272
  C          3.37389776782494      2.29472318403385     -1.36309365903538
  C          2.32851110142783      1.06091961579967      0.75007620854916
  C         -0.73992659110925      1.88587973723007      0.93489253569037
  C         -0.75437476858791      3.21564677098291      0.58201931187344
  C          0.10135943175490      4.14936097494631      1.30002495618274
  C          3.89427055263650      3.48477646362794     -0.59146357336185
  C          1.91254729147806      4.02668483252498     -1.56037786662326
  C         -0.32422496799845      3.26133525082498     -0.82571189425746
  C          4.07358957351766      2.71305596057732      0.58058091138849
  C          2.16693414867444      2.72547223393864     -2.03551494934261
  C          1.06362969038475      3.64556695521191      2.19191234283614
  C          3.33141728942300      3.00112912927218      1.71958058241659
  C          2.28735115565269      4.90193968738582      0.58160157383495
  C          0.86716080686593      4.85920745180405      0.30027817860981
  C          0.89269035097372      0.74251539838341      0.67401406871372
  C          2.44469856218495      4.12332209335090      1.73721998808001
  O          1.47832936944039      0.52450466503501     -2.53301743100504
  H          0.74097087020174     -0.07435700782679     -2.39421130060627
```

```
Structure: C25OH_c.xyz
27
Coordinates from ORCA-job 25_3 E -1027.796431039287
  C          1.59560844605747     -0.15144728893269     -0.17311643838405
  C          0.14433730461242      2.84770536105900     -1.47260559080033
  C         -0.04895325405454      1.98112422388227      0.31879160382404
  C          2.59877674771768      1.45046049237574     -1.55468984381861
  C          3.40241925271663      1.24970686895014     -0.42013225953599
  C          2.74701477577032      4.71661863952930     -0.36622016095274
  C          1.12975205839096      5.52561710043698      1.06968984108854
  C          0.84137872025133      2.06631993727729      1.44304786142750
  C          2.19385263806606      1.47202375845028      1.40270025914552
  C          2.53042079740566      2.86151762301567     -1.76640452945422
  C          2.64439097513321      0.44263253740597      0.54475205736012
  C          0.29784206635136      1.48194198028118     -1.03257570863349
  C         -0.48698054062493      4.43998420633084      0.31198058067666
  C          0.24803248977579      4.52911497193076      1.59252227450991
  C          3.40357930398092      3.55617001523904     -0.80151611137185
```

C	1.48106959669760	4.86058980921538	-1.11568044027917
C	-0.60018796087473	3.22637938466959	-0.26287387430219
C	3.74707395092022	2.54871362167559	0.13794708115442
C	1.29124104263932	3.59329935821282	-1.81968703333773
C	1.01495214113837	3.42058343904156	1.98542591497497
C	3.12258029343141	2.62039146154110	1.41081551750401
C	2.46376003838687	4.97771368490174	1.01771042003258
C	0.52326713909865	5.35718046860525	-0.23344198927584
C	1.37581651563135	0.64088444490940	-1.32721698758569
C	2.43332855267811	3.77078955262392	1.78536464163626
O	0.71870435872874	-1.04312049391596	0.36125992680943
H	-0.00921744836680	-1.16237513939535	-0.25315701122609

Structure: C25OH_d.xyz
27
Coordinates from ORCA-job 25_4 E -1027.783020332251

C	0.64621472055197	1.20725440485324	-1.70387288249460
C	-0.56724997123316	2.94976496132163	-0.33332085451332
C	0.39082500008279	1.05829588920179	0.63637215637682
C	1.91395835486366	0.89657801454646	-1.11784936634891
C	3.65533650794724	1.66135843333964	0.16712922074021
C	2.02217452518491	3.71459351671185	-2.29347779113159
C	0.17613277755685	4.09835937694191	-0.88874004521531
C	1.24715710221628	2.36145523439479	2.54780341472365
C	2.57914433268175	1.90482336980513	2.23389689312135
C	3.05540390521797	1.68454646339102	-1.18242191504456
C	2.76358048937267	1.02086518500712	1.13848263596958
C	0.16870908977045	1.92067004174740	1.73600208963412
C	-0.46746855380724	3.08826592029668	1.12883570435138
C	0.26846842213035	4.19647502482066	1.50686804499317
C	3.00054488719792	3.11979646107590	-1.56415474695891
C	0.87708234992330	4.13731931984462	-2.05902567694710
C	-0.18801239098182	1.63268365441573	-0.63432666356901
C	3.93324861066226	2.92340902194077	0.63823076462939
C	3.26625595640780	3.80903568334039	-0.31193364277827
C	1.24782262621626	3.83112504073520	2.48007164963171
C	3.42319895807701	3.08368690443021	1.97396626654426
C	2.30238918766311	4.56145811166086	0.44568093420410
C	0.88635431692911	4.64401499755719	0.25710628214904
C	1.66940325425421	0.60575367628667	0.34447103339739
C	2.49270497364193	4.12460775528691	1.88881247648026
O	0.45258818364103	1.33027306589253	-3.04438591285408
H	-0.41210765684333	1.71405046401163	-3.20753006261301

Structure: C25OH_e.xyz
27
Coordinates from ORCA-job 25_5 E -1027.779649429613

C	1.49187938104419	-0.09545681236563	-0.20172323702855
C	0.21020761742392	3.07723262545670	-1.49237437965109
C	0.04148445579971	2.08054065947254	0.58317943094684
C	2.62063950339354	1.55927202966787	-1.37816807090700
C	3.50984156307531	1.08035222141470	-0.43211999593653
C	2.96168806626964	4.48119586814978	0.47530262337395
C	-0.03374950017089	5.25707271123441	-0.67159480154898
C	1.08869477310613	1.98748846060286	1.58233009928124
C	2.34682212498455	1.27111074521197	1.49461670261688
C	2.66273529436565	3.03154680353883	-1.51614302012414
C	2.64539331251359	0.31607081131581	0.50353153816185
C	0.29114278359855	1.79386879887800	-0.84759458858890
C	-0.51026592899687	3.41306946414465	0.63531210153393

C	0.31051665587464	4.25441374707829	1.44187553051005
C	3.47892995725795	3.50776327872200	-0.47210499023257
C	1.30586854046361	5.08994700625881	-1.27480419158656
C	-0.58788675614037	3.99836175197333	-0.68671853932392
C	3.93228223087398	2.29867128621317	0.24260443234665
C	1.44469925024613	3.74355585386929	-1.78301744679833
C	1.34900591967223	3.40627808809482	1.95703852832918
C	3.34829589885150	2.36013270496174	1.46573422768318
C	1.93365474276764	5.31474353945023	0.01210715318074
C	0.63471441825061	5.38536562930314	0.64402428582947
C	1.30753008969361	0.86761097983952	-1.20000255814511
C	2.68234049967447	3.65751218569388	1.63422451920928
O	0.56512341723998	-0.95328960426580	0.30309976881652
H	-0.22772833064062	-0.90391081988984	-0.23592513134564

Structure: C30OH_a.xyz

32

Coordinates from ORCA-job 30_1 E -1218.467526226644

C	0.19547365996748	1.63678104819832	0.25477189196977
C	1.97717806188055	3.03765819142476	-2.59358408951937
C	0.35461251997059	5.26139358416618	0.43506259760288
C	1.05207584582825	2.09024695906636	1.44226721314925
C	3.57381797791679	2.44276845114192	-0.80950750930315
C	-0.11072490768285	3.27890181295500	-1.52132714203630
C	0.99346400251904	2.31206648225302	-1.94822188245624
C	2.53494004605758	1.58955572155306	-0.27051364000507
C	-0.41142851899545	2.98718406877481	-0.18023113473738
C	2.40908686742535	1.83651200280232	1.19670170711146
C	4.14272124382026	3.12715893257033	0.32658709442722
C	1.28129016902354	1.50453522729669	-0.83468185647616
C	1.44165508000899	4.39884476674628	-2.77900532974110
C	2.36852060586544	5.32018196382918	-2.46704108284895
C	-0.16145251720564	3.95071462613391	0.77175200497422
C	1.61740762961318	4.31605256004400	2.23507928718357
C	3.38153523287545	2.77156199312013	1.55308297065122
C	4.35717177673667	4.51719884551326	0.30495196971467
C	1.41373245294103	5.51609245555052	1.38174915668400
C	0.24917566257038	4.55079552855600	-1.92548595300018
C	0.66149796567973	3.36660870273475	1.87214284142795
C	2.99147221321771	3.98754107938029	2.16215324546421
C	3.58698605157989	4.62397936910158	-2.00884578563590
C	3.29036430451230	3.18325498360067	-1.99280839872843
C	0.56681704937271	5.56807997645564	-0.93969961829184
C	1.86367467649371	6.13282143164295	-1.34288196953260
C	2.88265735854253	6.21963600397534	-0.42760225958604
C	2.66605416141893	5.99762246250560	0.95857749039199
C	3.99156208812140	5.24942365632167	-0.85611992875806
C	3.64828826509393	5.06139800023253	1.42960188506272
O	-0.62221914050191	0.58375017854489	0.52329464435295
H	-1.12626788431796	0.35102894001772	-0.25988840606020

Structure: C30OH_b.xyz

32

Coordinates from ORCA-job 30_2 E -1218.456171252097

C	0.14714888434714	2.09172526854736	0.79898235916387
C	1.36414255443911	3.57492926890583	-2.90338561314997
C	-0.26345985103037	3.51297987955469	0.23283743303024
C	1.09397742366481	2.54984356114123	1.94046021151171
C	3.46757286052403	1.94248826551894	-0.62014346090926
C	2.68880217432547	2.04158549804306	-1.82827658894098

C	2.74518847581622	3.14979067874223	-2.65347396523104
C	1.20390702432898	1.50410084904876	-0.13238960074441
C	0.47194559413954	2.83093737261968	-2.04441347489178
C	2.47735145402357	2.25993082408189	1.65521667345448
C	4.33373650278297	2.98216272094270	-0.34884998346507
C	1.26659102461645	1.77781149239866	-1.46764845357174
C	1.25237146690851	4.91162315300781	-2.63273167244102
C	2.52553832817544	5.42925757685056	-2.19132968375456
C	0.13008224402297	4.49630868664507	1.16294681744183
C	2.28926105101647	4.52772575871339	2.14039241194153
C	2.53436960636281	1.59283858548479	0.43934867228959
C	4.22508385415410	3.76777745787264	0.89456054337131
C	0.78632718253180	5.67558300645100	0.73927020536787
C	0.26648295485526	5.09893646606227	-1.55572341783230
C	0.98617337833011	3.87721638268654	2.18321215994774
C	3.26724566273879	3.49081033199268	1.84134284446366
C	4.34770901929045	4.18887280505580	-1.16181564947328
C	3.49877906132543	4.32845791066950	-2.24029237396340
C	-0.19335873250893	3.79914457836814	-1.14095319739306
C	0.86370781887420	5.96293694503366	-0.61713718267253
C	2.16581566128710	5.61515883180838	1.28757860394199
C	2.29574288582749	6.09908088256297	-0.99017196762599
C	3.08440039478301	5.85283712479692	0.19801966362467
C	4.14531977359173	4.96511510005367	0.07196293486197
O	-0.87044312424833	1.22344408227447	1.04432759049537
H	-1.53637264069736	1.64993866027437	1.58860717460365

Structure: C30OH_c.xyz
32
Coordinates from ORCA-job 30_3 E -1218.441549929083

C	0.44834264019677	1.60109808538573	0.56547271980781
C	1.95687820891650	3.35451638307668	-2.84267183626057
C	0.94917452224187	5.98000520540633	-0.09878217425631
C	0.83816323614876	2.50775839347075	1.74090010998076
C	3.45389933292483	2.41306799909244	-1.37176319754752
C	0.06310622243195	2.90067561027025	-1.62537973760262
C	1.24519669192888	2.31355173256578	-2.20295418812075
C	2.17708584958852	1.62785081327491	-1.34421244455141
C	-0.25063376458775	2.66857320670994	-0.31236453488434
C	2.23264005139144	2.62235557444133	1.91221108121547
C	3.83448517320056	2.70768941075777	-0.01438905702472
C	1.86281549216453	1.39583507375088	-0.03142257110128
C	1.31358539420718	4.63632928501465	-2.59327282057641
C	2.28553449347668	5.54070624679633	-2.12384200863256
C	-0.33582818421660	3.92071929989048	0.38345097459171
C	1.26684903235647	4.76628048656633	2.04109979091539
C	2.85938960467761	1.99652832629612	0.80841414425073
C	4.17262625838366	4.03924466749156	0.32948466654727
C	1.52671505336456	5.86764806580737	1.27788036761425
C	0.18068191310121	4.38408090438906	-1.80712731561769
C	0.27877034703632	3.79855664713331	1.65245465304940
C	2.55847938201295	3.98866957953034	2.21280361503200
C	3.57832834474128	4.76194227593439	-1.95200066232339
C	3.31604002387847	3.43002363330507	-2.32693075547669
C	0.00574091841119	5.01326141761475	-0.52361488400250
C	2.05645947425845	6.27795184520804	-0.96964934634097
C	3.26967415289288	6.12989981828845	-0.14393362663333
C	2.99305351575732	5.95721103129924	1.15925542003031
C	4.10234047172224	5.04582464970219	-0.69801693470721
C	3.55050280921552	4.64916394019739	1.54679810062709
O	-0.26341903754378	0.49553283769411	0.91292170041720

```
  H          -0.46553763451441     -0.02120244015233      0.12951074386490


Structure: C30OH_d.xyz
32
Coordinates from ORCA-job 30_4 E -1218.434503351247
  C          -0.16176837974010      2.11944801643775      0.00248662202558
  C           0.47747369226182      3.39821356364893     -2.02213502256660
  C          -0.39545536128536      3.62786098435042      0.14703312120876
  C           1.46806827906974      2.51247136564950      1.99492057460832
  C           2.90460851715506      3.25182553060496     -2.67531016193695
  C           1.91618594927018      1.63577241357849     -1.19812836677715
  C           1.54431069780232      3.84623397796843     -2.83412918082965
  C           0.99265014857466      1.71524738087933      0.95496025319616
  C           0.63101283661152      2.15272399518449     -1.35815274154722
  C           2.95960185363168      2.66182339039905      1.83074261319906
  C           3.32075506069583      1.99344105754161      0.65422044890248
  C           2.10419738686223      1.35251949347894      0.18459885423586
  C           2.06405581029102      5.14066617185049     -2.67218848660258
  C           3.44514696577476      4.58505613762126     -2.54759486608390
  C           0.01409469254822      4.37265290852550      1.20812680321743
  C           2.02363092248918      4.72784563578758      2.28242985728834
  C           3.88952175623594      2.69760891955464     -0.46192858639187
  C           3.90663280368555      4.70528929442517      0.93653521654033
  C           0.63777190357410      5.62522235694286      0.65970554481950
  C           0.59566762168233      5.57475194211549     -0.76761144381158
  C           0.94448324977735      3.81941401604857      2.18089659890078
  C           3.27532950884802      4.02880850962698      1.97637023576370
  C           3.95313143349284      4.99768442021267     -1.36545769869487
  C           3.09799681069949      2.32885926506638     -1.68366033001033
  C          -0.07571890438590      4.32694432227518     -1.08118529786011
  C           1.76384228067778      5.91506824380892     -1.52035350992252
  C           1.86108051317021      5.79389197558266      1.32028789626687
  C           3.02456384499109      6.01730836452202     -0.79520930865890
  C           3.06804163573558      5.89668632506531      0.57161306315811
  C           4.27470078089417      4.05696348958528     -0.28796645058372
  O          -1.32492744673159      1.41669976180789      0.05545710833779
  H          -1.13954685871840      0.47634677606287      0.00095663818467


Structure: C30OH_e.xyz
32
Coordinates from ORCA-job 30_5 E -1218.419220052506
  C           0.63983702787316      1.58635829356791      0.24522257103776
  C           2.87254052775607      3.32221868574801     -2.58771175611827
  C           0.31128089805988      5.69409234662811      0.05944808102841
  C           0.68432882157764      2.57068457474608      1.45774539899252
  C           3.72382492590175      2.72916086437654     -1.56863977375615
  C          -0.51750011359915      3.61578626627067     -0.87231909227878
  C           1.89232244719307      2.24009561532169     -2.43069165945086
  C           2.70711347491643      1.70100380191103     -1.31675715445742
  C          -0.01829482509864      2.33361038867635     -0.91841704869125
  C           1.97038661585795      2.64493190270594      1.98446873094497
  C           4.23197779060020      3.49380022191724     -0.52101493276423
  C           2.19967473717213      1.51174967393423     -0.06447958864898
  C           0.17145071497551      3.92653972582859     -2.17269608284143
  C           3.21500377594352      5.47525240156650     -1.56107823485429
  C          -0.34396110930061      4.46531098694242      0.26517261224395
  C           1.18975194523343      4.80541094394724      2.02499168406972
  C           2.90569968768575      2.12376976297297      1.02021867351392
  C           3.90247049497631      3.08756732916069      0.82256139503352
  C           1.27853385575604      5.90952242186376      1.16836713113065
```

```
  C          1.03794906170980      4.96635157181403     -2.26577189854608
  C          0.16087111035483      3.88167072301444      1.47447563824494
  C          2.33353641096920      4.03126374358824      2.31005262872855
  C          2.49209693250187      4.65304320772577     -2.48458708702534
  C          0.59343277241947      2.52255999298234     -2.24681307763765
  C          0.99031060528696      5.96157425740825     -1.18743552964486
  C          2.28492846752120      6.34402811791344     -0.87046477281236
  C          2.51103780419180      6.24978641251078      0.57689251860828
  C          3.62802195796967      5.40499450055348      0.75068263487272
  C          4.05769871490557      4.91582304830489     -0.58184049646355
  C          3.52653464187514      4.29166266650994      1.60417692565372
  O          0.00133280639377      0.40055652979711      0.43439582159771
  H          0.42694704193698     -0.08883098458256      1.14217572728243


Structure: C35OH_a.xyz
37
Coordinates from ORCA-job 35_1 E -1409.006407477184
  C          1.87108305689446      2.07339385371409     -2.26546216431068
  C         -0.97562857080960      3.68358592250251     -0.51551533821245
  C         -0.15834423618705      2.65261278860345     -1.09030789306220
  C          2.07153737393609      3.87010468278041      2.82742703005913
  C          2.41536450328630      1.65827332534056     -1.05734282949635
  C          1.61903815480792      2.62056708666903      2.35305294216062
  C         -1.36330583281686      3.36305971796027      0.77363082877569
  C         -0.50564802060798      2.26893679681748      1.17303555495401
  C          0.78914667783403      2.97835689485907     -2.08807955068598
  C          0.22177579508481      1.78596174930168     -0.00135026240942
  C          0.90278624970051      4.76419879006849      3.00512414944382
  C          3.84936131105073      3.97244860508177      1.18020224173532
  C          1.59026926065912      1.51138935159778      0.11257997594427
  C          0.17697302320646      2.66388961732856      2.30088567755291
  C         -0.55462671061677      5.07130310454219     -0.59149525929173
  C          2.66941754875805      4.63355509174664     -1.90727343079900
  C          1.25576204237576      4.33016001467451     -2.11750178871344
  C          3.64879247130537      3.83302733614271     -1.17214049927851
  C         -1.05377484683432      4.51138617328829      1.61393895216939
  C          1.44621993142195      6.39155820966821     -1.00304561037067
  C          1.20210372228292      5.97784492010454      2.41350323261158
  C          2.63941068549643      5.94650354748149      2.03487380345810
  C          3.56940139918444      5.96480419691979     -0.19720985666876
  C          3.48664332105631      2.64372458634349      0.78889314032916
  C         -0.60546978863052      5.60140143031515      0.79269224639051
  C          2.75752223350493      5.95905243999339     -1.38654184264189
  C         -0.24732630364665      4.05386014764455      2.65772026022739
  C          4.09603851174356      4.69773207996771     -0.03026861072475
  C          3.49276882370203      2.54371551289931     -0.67032808591569
  C          2.32491473014955      2.01270471979007      1.27179514704185
  C          0.49892688157359      5.38583115206607     -1.43535392639415
  C          1.45752784530726      6.85196914487766      0.30026534720959
  C          0.47050273234428      6.39844927536863      1.25485919396325
  C          2.81535423346427      6.60072508068845      0.84593579849749
  C          3.14091353699116      4.59725670834715      2.24030892551794
  O          2.58386278551169      2.12329242223597     -3.42263768611444
  H          3.41267544352582      1.65613350911150     -3.29444981905009


Structure: C35OH_b.xyz
37
Coordinates from ORCA-job 35_2 E -1408.993079513691
  C          0.62980321771849      1.44052547024383     -1.76539290807838
  C         -0.28965464849482      3.51073291592463     -1.18236138611524
```

C	0.48743654576978	2.78078864885310	-2.12435140384076
C	1.95683908063813	3.85662254695821	2.96434721396882
C	2.71561205556649	2.50389750752641	-1.50501582262542
C	1.74192050866391	2.59620283527617	2.41303803825947
C	-0.56902507377217	2.95266060472524	0.09507786949993
C	0.22734272607205	1.95535701830174	0.81293597922535
C	1.85162299431491	3.35686961741804	-2.25960529771282
C	1.56288657456585	1.52939640468421	0.34718985683693
C	0.65732159250087	4.57064694085143	2.91282230906589
C	3.83542221787580	4.23318389394438	1.42326786832011
C	1.83029449317805	1.43362703217029	-1.01413033422851
C	0.34303094643890	2.39877052267143	2.13670970888664
C	-0.10712013638098	4.95806063787326	-0.99446992003516
C	3.26744056111535	5.22904899047413	-1.74320624549415
C	2.02350125905441	4.72521385537522	-2.22032217287895
C	4.04440999277094	4.41889459183101	-0.90616035592020
C	-0.80493680751279	4.05446232625008	1.03074268877673
C	1.64677984530984	6.65233224791020	-0.91955149477565
C	0.89572085841507	5.81063959568044	2.37766306046594
C	2.36620667631847	6.01996868804280	2.28216245230168
C	3.64196111280302	6.40099434300120	0.25985919469277
C	3.54220727697526	2.93090595434373	0.79887753184595
C	-0.45378298744362	5.28050683514546	0.39707345843105
C	3.05962535268874	6.47990935762646	-1.06479625897959
C	-0.26660994284819	3.69735381256683	2.27180067464601
C	4.21125321144598	5.13044929766940	0.38499499747371
C	3.66560902527707	3.09073007424796	-0.66050481716194
C	2.51189978822313	2.13855096186455	1.28205274953993
C	0.96178150571599	5.57717036583107	-1.61746882631368
C	1.35929916202586	6.92021308061118	0.41833119009798
C	0.32220875392363	6.22492909471271	1.11777098665309
C	2.63512992516340	6.79280956761064	1.18808646257210
C	2.97585118142151	4.71233780728572	2.45773010784679
O	-0.32214408235432	0.47381267032608	-1.67125100832463
H	-1.14517477655043	0.81419390276396	-2.02952617418703

Structure: C35OH_c.xyz
37
Coordinates from ORCA-job 35_3 E -1408.982694504257

C	0.26619487054181	1.47070575107442	-1.60653621945789
C	-0.57072319693099	3.64408277945022	-0.97422377129735
C	0.17445719683380	2.84550129695845	-1.89381967213964
C	2.07487799579682	3.76366638094058	2.80307152978975
C	2.52900820723946	2.51042559383286	-1.30051291371441
C	1.79268348323630	2.52726430191185	2.14613558726208
C	-0.97563998969148	3.10872461991939	0.25712148608826
C	-0.16167267567403	2.02686913833668	0.74011923101709
C	1.56481287038868	3.30492332807771	-2.04840506828090
C	0.62821799293707	1.31521140536560	-0.23529866167771
C	0.80131478755228	4.50787711627095	2.92552945399679
C	3.94406744980759	4.24993909537013	1.30153629583224
C	1.99728392972346	1.68717166194402	-0.18168530397704
C	0.37148908224664	2.42162257984940	1.97276664537879
C	-0.28795472826366	5.06134860606350	-0.79714406929005
C	3.05527468422857	5.24813315769979	-1.79862359118856
C	1.77999441246832	4.70719941194616	-2.10920911860007
C	3.98498585024417	4.46268065814878	-1.06544400020113
C	-0.53848502349797	5.40311281020638	0.61885091993611
C	1.53690641402691	6.67741451434164	-0.84206603970348
C	1.01218416629659	5.79514859862269	2.47998414917985
C	2.47208201282956	5.97602952965656	2.28517129104069

C	3.60782593814382	6.39464453448986	0.16811419629804
C	3.68091388945065	2.99620421517266	0.68794670097325
C	-0.92964441385957	4.18903268000389	1.25082047787714
C	2.91566252825243	6.49576802861180	-1.12245202030014
C	-0.20489670444564	3.72217971230753	2.34457519645784
C	4.24079714037407	5.17615878229519	0.23383935200966
C	3.67896900131981	3.15726313974563	-0.78231710622522
C	2.62596181053615	2.15011817014745	1.04643108389679
C	0.78074391573937	5.60342324306863	-1.48256080818333
C	1.36069861149086	6.93653132322903	0.52102209705739
C	0.35030634799273	6.27344098575887	1.29519624485338
C	2.67197270606610	6.77307792356674	1.19067014209820
C	3.08753959199908	4.65534881241133	2.37541223436252
O	0.68635950271636	0.60598296755392	-2.56846978601025
H	1.00740031260216	-0.19145684104884	-2.14112618713217

Structure: C35OH_d.xyz
37
Coordinates from ORCA-job 35_4 E -1408.974731677948

C	0.62739973460968	1.29694091605178	-1.86799126243621
C	-0.20100683090632	3.51940804548939	-1.42581978161475
C	0.45959869165505	2.58948005377785	-2.30535937349827
C	2.06653825729352	3.74891865211123	2.90162342672490
C	2.70405564439852	2.48069817335293	-1.67400322161197
C	1.83067599715424	2.54072660975738	2.28488307497159
C	-0.43674942220040	3.17524677926915	-0.08304483077459
C	0.26820181873922	2.06002814870144	0.63855479683165
C	1.79280123232855	3.23092322221538	-2.41840573357016
C	1.86270766691896	1.35969740186245	-1.15251450895791
C	0.77249184828322	4.48334637422787	2.94440537783710
C	3.85597629569771	4.24741163617529	1.28793117595007
C	1.59488013669149	1.56466711169991	0.18816511527708
C	0.40260489799684	2.44903914573376	1.95349043643728
C	0.20706738346914	5.87746602452581	-0.74978326996332
C	2.80329327024468	5.31628809501713	-1.74865878315345
C	1.68760629921470	4.62806702197526	-2.25582193804075
C	3.77699915731398	4.53925622634437	-1.04270218706207
C	-0.62072154839784	4.23753643436867	0.91681653621970
C	1.30088115718861	6.81800506722690	-0.55191705805659
C	1.01816893114597	5.79717341854591	2.63629599112734
C	2.49663719470412	5.97443436454959	2.41731771665569
C	3.45300475821933	6.44645098438564	0.24394544110812
C	3.57698860690557	2.96880808257557	0.63210724093322
C	-0.35750654928125	5.57964355482759	0.58430015310269
C	2.58287015689302	6.49065625236239	-0.96437230345317
C	-0.16225788237709	3.73568659000130	2.18217488289627
C	4.06088501364281	5.20877826262688	0.23766833016932
C	3.59973046919307	3.17807043813227	-0.83284842502580
C	2.56990932454143	2.14721736118579	1.12282478038523
C	0.36383239642245	4.86944830146559	-1.66613124265538
C	1.33745148738504	7.16971051073010	0.83734100310744
C	0.35988438543971	6.39636509132416	1.53003235869682
C	2.66893768630849	6.84827939166437	1.36191931501588
C	3.07568703029218	4.64940363835022	2.40784360634566
O	-0.36209093498351	0.37891203092170	-1.70146445442975
H	-0.02546379142661	-0.34941941081510	-1.17438241275416

Structure: C35OH_e.xyz
37
Coordinates from ORCA-job 35_5 E -1408.953001898439

```
  C          1.88247973360661      2.02321582652353     -2.27638923067651
  C         -1.09323395854695      3.58729631626075     -0.69796535907325
  C         -0.24823791492232      2.55979339743884     -1.20393167725721
  C          2.15583287846379      3.86381571273125      2.91869705726658
  C          2.39867292952947      1.69329588473155     -1.04727038367541
  C          1.62812527495038      2.67260526367134      2.34388114920196
  C         -1.35366201575094      3.41760861643131      0.69321461172185
  C         -0.50619471877215      2.38658734439727      1.11117960732700
  C          0.75133687991078      2.89037665650394     -2.16294022847692
  C          0.18160067782717      1.79918028569320     -0.02420478345713
  C          1.04410845590482      4.83637451787853      2.99088942968458
  C          3.90473262501884      3.94255581897113      1.19597466354345
  C          1.55114158275612      1.56492511699771      0.11339436537264
  C          0.21498463203758      2.82356267867922      2.25215980908070
  C         -0.75225546836312      4.93007398464765     -0.90851107078689
  C          2.57450178808430      4.63729298295297     -1.82462325996492
  C          1.19170448742758      4.25291534102257     -2.23972289494661
  C          3.55147766083659      3.89809583513868     -1.12149110212648
  C         -0.96344652138894      4.62730132355947      1.40929990378237
  C          1.13693841631896      6.23347476359386     -0.95736483694548
  C          1.57316090051084      6.06289284773953      2.63226040663405
  C          3.04365398671528      5.89177416393987      2.37016027378609
  C          3.37673689330180      6.00796764107021     -0.02796607691524
  C          3.49939874226366      2.62648718705730      0.76151027540887
  C         -0.60716861301527      5.58780129160616      0.42786243038894
  C          2.46362699801170      5.93785134466490     -1.13614432668193
  C         -0.11960719181490      4.20404777521382      2.49829700944981
  C          3.99131135397989      4.73037779908449      0.03483125902966
  C          3.52041357347173      2.53171243842318     -0.66459459749944
  C          2.30748127425523      2.03634933763822      1.26688304432161
  C          0.35108962585182      5.24732093739637     -1.67926058040864
  C          0.53794931002577      6.45671830708222      0.35430926089104
  C          1.44083856651454      6.73516266770500      1.38664240836514
  C          2.91797568912018      6.58748118004076      1.14997935555228
  C          3.31670099374754      4.52065521386058      2.37405499647801
  O          2.64636672426431      2.12369682364817     -3.39709446919855
  H          3.50143373975266      1.72412537872234     -3.22158646116950
```

```
Structure: C5OH_a.xyz
7
Coordinates from ORCA-job 5_1 E -266.066600786839
  C         -3.08360030905859      0.00893464734134      2.06310012121177
  C         -1.31875842996892      2.27863144131121     -0.42801921629547
  C         -2.40987023235199      0.70484675637826      1.30213675852530
  C         -0.77548992809625      3.06774022096388     -1.29670550487448
  C         -1.86585000078647      1.50716366644087      0.41887951231541
  O         -3.29971336877923     -0.89399975117481      3.05688310271449
  H         -4.24710773095854     -1.00372698126077      3.16644522640296
```

```
Structure: C5OH_b.xyz
7
Coordinates from ORCA-job 5_2 E -266.061850141361
  C         -2.76955777220156      0.04446263432694      2.03061507899797
  C         -1.99984957754737      2.57007756385766     -0.75616350884408
  C         -2.29272738964132      0.78536505676274      1.21447807439135
  C         -0.74153589644537      2.24810315421051     -0.39330477080440
  C         -1.77842794909797      1.64616779938299      0.26611733280176
  O         -3.22950455074902     -0.81515955015215      2.97882320899111
  H         -4.18878685910311     -0.80942664866169      2.94215458237486
```

```
Structure: C5OH_c.xyz
7
Coordinates from ORCA-job 5_3 E -266.004465152762
  C         -2.55110959128027      0.82310366080919      1.15700505031072
  C         -2.52613510477409      1.74675888389660      0.07481886189001
  C         -1.45116063003483      0.38148253454706      1.78240537456673
  C         -2.50950835276425      2.52677107202919     -0.83957924265090
  C         -0.42718138982692     -0.04786235410276      2.38387682948053
  O         -3.80747342920784      0.42862717267832      1.49691416167701
  H         -3.72782150218930     -0.18929097071412      2.22727896792623


Structure: C5OH_d.xyz
7
Coordinates from ORCA-job 5_4 E -265.991736496206
  C         -2.53389771768603      0.33381085394878      1.47518123176213
  C         -1.44240183719500      1.96183067636879     -0.52977152696198
  C         -2.39880866372799      1.64037752049790      1.82051780884277
  C         -2.16287390233899      0.93448151721637      0.25934272425627
  C         -1.53086477711211      2.17597019452085      0.78829636731981
  O         -3.22733478031298     -0.78149308664978      1.82851130803215
  H         -3.70420832699616     -0.59538767675944      2.64064208465741


Structure: C6OH_a.xyz
8
-17.1035692573
C  2.01069154562693119814 -1.44088453156492768414 0.76450904287359378042
C  1.59870738000925993738 -2.53679518482560251158 1.16644889931093187485
C  2.90248915873105950425 -0.50688311881061498543 0.41768888361996581171
C  3.73704669069117967339 0.34609172596511750264 0.11783165091939699909
C  4.57669321584909383205 1.31998930386847801444 -0.23712660647164432448
C  5.34455379037247713114 2.21801734674329598107 -0.52757313866598731789
O  6.16177928884576875390 3.12983850506436800387 -0.92984374853131024352
H  6.43577892588595901202 3.72140595176475397210 -0.22516498741754756296


Structure: C6OH_b.xyz
8
Coordinates from ORCA-job 6_3 E -304.109015155648
  C          2.56271151483408     -0.88690063403340      0.91127617055958
  C          2.01482112375545     -1.11409729192079      2.04192588176054
  C          3.14977835337407     -0.58675227439405     -0.19996519957936
  C          3.80738465796177     -0.14267606638672     -1.18903456810579
  C          4.57091158743475      0.95055840039260     -1.01486595891524
  C          5.14814812742392      1.91137504748801     -0.54316102311188
  O          5.44997585518570      2.71793130749921      0.40950410136467
  H          6.06400877604190      3.40134150426824      0.13109060224844


Structure: C7OH_a.xyz
9
Coordinates from ORCA-job 7_1 E -342.222687106406
  C          0.79605066132566     -0.28189555577134      0.68133749507053
  C          1.98430058055895     -0.19894372826518      0.48269706063148
  C          3.31240253786152     -0.11984104347761      0.29132856996599
  C          4.52716723914849     -0.03830929374581      0.09466459749046
  C          5.81813971641273      0.04263963559785     -0.10240943072579
  C          7.07031206159061      0.12517207466074     -0.30237633765367
  C          8.34516531841234      0.20793939257574     -0.50180182626760
  O         -0.55972348704475     -0.32014670692780      0.78140399783582
```

```
  H          -0.79284250041654     -0.70084188376967      1.63130205607134
```

```
Structure: C7OH_b.xyz
9
Coordinates from ORCA-job 7_2 E -342.227191968188
  C           2.46829383882559     -0.29003900436005      0.67616046057409
  C           2.61467844710925      0.27958054864988     -0.58525971268483
  C           3.69369169324465      0.53778540488846     -1.16899733434484
  C           5.02441615458881      0.55102587212129     -1.21411797026756
  C           5.63801356277450      0.04249602864471     -0.09779007807931
  C           4.80109134056677     -0.37331092652917      0.83053111359277
  C           3.66435602882803     -0.59667950206866      1.33908421421100
  O           1.23666699772561     -0.52097678345540      1.20470853161264
  H           1.35976405889402     -0.91410874701412      2.07182696309635
```

```
Structure: C7OH_c.xyz
9
Coordinates from ORCA-job 7_4 E -342.180757879609
  C           1.55444341957720     -0.50766033763749      0.80161360414970
  C           2.54149588056753      0.03769646791407      0.39316487482924
  C           3.68415641099840      0.66328973856805     -0.06693967337034
  C           4.43580036808125      1.63029140707011     -0.61580522664733
  C           5.08752307921720      0.44866098425278     -0.19913588921339
  C           6.95456759650215     -1.28068724630415      0.27552316189295
  C           6.05842952844064     -0.41677207445239      0.02682257218321
  O           0.48443346506678     -1.20088089863522      1.27503092783681
  H          -0.29987762589393     -0.65816513931530      1.16587183075770
```

```
Structure: C8OH_a.xyz
10
Coordinates from ORCA-job 8_1 E -380.306822831653
  C          10.42831831664642      0.50556870663208     10.43652074903766
  C          11.85265306944734      1.12211382791900      5.56781131641721
  C          11.47767573537139      0.97360134797684      6.83735455393148
  C          12.20650424989292      1.25814416824832      4.39004175007066
  C          12.58497225286782      1.40086485469468      3.13762352239943
  C          12.95314085023209      1.53893143821284      1.92417749478182
  C          10.75015206514639      0.66907081972918      9.28777696103551
  C          11.13164319134213      0.82991490365402      7.99943783249332
  O           9.98045280029943      0.38964561649768     11.71541805272194
  H          10.57578746718678     -0.19342569084862     12.19207777035098
```

```
Structure: C8OH_b.xyz
10
Coordinates from ORCA-job 8_2 E -380.285072669918
  C          11.02571178961983      0.67301867427564      8.65317446531153
  C          11.21493613166636      1.64717356611176      5.48480269627363
  C          10.44147451302708      1.85726258646669      6.58258353197658
  C          12.02565373295920      1.41572307672684      4.36465176430552
  C          12.51892305229993      0.18042708992738      6.78519512906578
  C          12.30594475930943      0.76654897940819      5.59036516549886
  C          12.09603855076014      0.09218722775851      7.95719845551197
  C          10.33701124844558      1.51654146709130      7.77170232333317
  O          10.67685608314784      0.48172350101791      9.95367661820487
  H          11.29875013719738     -0.13617616019286     10.34488985375813
```

```
Structure: C8OH_c.xyz
10
Coordinates from ORCA-job 8_3 E -380.270715244451
  C         10.68195901340190      0.60419326573020      9.68475490429565
  C         11.67145303273084      1.11985176089923      6.02037499682810
  C         11.22129701305287      1.09882111018683      7.16749966773337
  C         12.14979454711335      1.13950793634327      4.80337338708836
  C         12.61335067008509      1.15701792647664      3.62319486508233
  C         13.08915404147183      1.17104710549030      2.41880724162406
  C          9.80053874844895      1.62352587924287      9.39553560600698
  C         10.73007922770097      1.07400733908482      8.43607084458099
  O         11.21592742042881     -0.30069069688798     10.54827255728828
  H         10.76774627870634     -0.19285162855838     11.39035593271196
```

```
Structure: C8OH_d.xyz
10
Coordinates from ORCA-job 8_4 E -380.228287964109
  C         10.86394344418931      0.78197526714244      8.64250934166683
  C         11.02070806618750      1.90224284312147      5.62799639962299
  C         10.68702518319456      1.66157431693846      6.88852710734039
  C         11.91376228218881      1.44365400297624      4.81383158647773
  C         12.62592977907803     -0.00878128271655      6.68737881946416
  C         12.55577957887446      0.55722147876476      5.52050768765502
  C         11.62000083998120      0.60901776997332      7.40161609326358
  C          9.92991076581939      1.74127524170720      8.18766807144005
  O         10.99740336770665      0.21494184591946      9.87143632508255
  H         11.72683669121281     -0.40869147523547      9.84676856064318
```

```
Structure: C8OH_e.xyz
10
Coordinates from ORCA-job 8_5 E -380.202661916584
  C         10.76841809453082      0.65476003942717      9.36191262515792
  C         11.18642769445427      1.56004577878175      4.60871376777933
  C         10.32957353878768      1.68504427171797      5.57100157300043
  C         12.36896568741457      1.06826838975429      4.79483557604240
  C         11.99125602939819      0.73791067397392      6.99655965068568
  C         12.79601274062728      0.65155508403578      5.94215142434479
  C         11.06516366956808      0.86254953684291      8.10458683369073
  C         10.65593911451935      1.29032097063810      6.79876477314584
  O         11.58660490151848      0.35153551045194     10.40509297640246
  H         11.19293852232228     -0.36756025761599     10.90462080299047
```

```
Structure: C9OH_a.xyz
11
Coordinates from ORCA-job 9_1 E -418.377391070829
  C          1.47633999687702     -0.06903321893759      0.84932130709626
  C         11.35526335638235      1.20434294444690     -1.42011349579612
  C          3.94846689468706      0.23449430658262      0.26605534387095
  C          6.41588923076107      0.55488513090659     -0.30300395819159
  C          7.61492501605073      0.71206557329895     -0.57703778166254
  C          2.63947831817549      0.06199520140602      0.56794961507664
  C          5.12847289828982      0.38663311268840     -0.00720053324774
  C         10.10652848650071      1.03912271222386     -1.14065037502337
  C          8.87389328823942      0.87674056800801     -0.86280575096402
  O          0.16324165545348     -0.31087712212241      1.10794123899057
  H         -0.16655914163074      0.39095079212481      1.67386439144554
```

```
Structure: C9OH_b.xyz
11
Coordinates from ORCA-job 9_2 E -418.377881151360
  C          3.88064160906777      0.74057123530598      0.62905821238261
  C          4.93945584055460      1.61171679625287      0.87230898745730
  C          4.84923287245792     -1.11301146289109     -0.91294905444569
  C          6.13357018950361     -1.10417154385224     -1.31481411518512
  C          7.31783246259004     -0.71977574866994     -1.43754109862030
  C          4.20085774960656     -0.34568820953595     -0.19464117720956
  C          7.52477937281092      0.42550225456216     -0.73986855253233
  C          6.12909923604869      1.62580242625106      0.50160030689910
  C          7.36868574722330      1.43576771450366     -0.01886757695458
  O          2.61656183850309      0.86565352757408      1.11491493070611
  H          2.59522308671155      1.65895300583379      1.65511913909701
```

```
Structure: C9OH_c.xyz
11
Coordinates from ORCA-job 9_3 E -418.344335238458
  C          2.87583992596304      0.34545117661139      1.12072947877770
  C          6.43122108575560      0.84120919882091      0.77235712678461
  C          5.26695420269887      0.49329290585734      0.07210593993596
  C          6.67817926403030      0.24657783555862     -1.84758959154562
  C          7.98557084073219      0.44522665119998     -1.85215804061620
  C          3.97164306114772      0.43027428481469      0.64414792712088
  C          5.55757801430499      0.21454085591409     -1.27258617126823
  C          7.61129932857347      0.91777743565982      0.34069895743849
  C          8.50622096875835      0.81979384191152     -0.62941534498994
  O          1.66144394564191      0.29351381013270      1.73076627724065
  H          1.00998936217999      0.03366200414508      1.07526344271625
```

```
Structure: C9OH_d.xyz
11
Coordinates from ORCA-job 9_5 E -418.335148366596
  C          4.27109008913737      1.02200557904891      0.53730504599310
  C          3.88173740000197      0.26445577180646     -0.52066651596205
  C          5.48918635740891     -1.29723024748853     -1.80309248125772
  C          6.58755691658982     -0.92532241209587     -1.11343684033128
  C          7.31835673127455     -0.51645239487301     -0.16619589446520
  C          4.62208881353260     -0.45543174095630     -1.28483154234780
  C          6.86858880530544      0.68757409428423      0.40241552276806
  C          5.69217180828587      1.28814361081509      0.71567984121418
  C          6.84263984669182      2.07012579676478      0.71284448323110
  O          3.44023428212859      1.60469848662501      1.44270418468845
  H          2.54228895472124      1.33875345140360      1.23159419277195
```
